# Supplementary material for: Microkinetics of alcohol reforming for H2 production from a FAIR density functional theory database
Source: Nat Commun. 2018 Feb 6;9:526. doi: 10.1038/s41467-018-02884-y (PMC5802771; doi:10.1038/s41467-018-02884-y)
Supplement: Supplementary file 1 — Supplementary Information [file 41467_2018_2884_MOESM1_ESM.pdf]

Supplementary Note 1: Linear scaling relationships depend on energy references

When a potential energy is reported, different states can be used as energy references. For energies obtained from periodic DFT, it is convenient to use the metal slabs (Cu, Pd, and Pt(111), and Ru(0001)) and closed-shell molecules as references, as it was done in the present study. However, there is arbitrariness in the molecules that can be selected as references. For instance, a study on the water-gas shift reaction can use as references {CO, H_2O, H_2, and *}, but also {CH_4, O_2, H_2, and *}, and even {CO, O_2, H*, and *}, as long as the stoichiometry of all species involved can be generated from the references.[1] Finally, some authors report their energies as obtained from the DFT packages.[2] As the selection of energy references is a source of arbitrariness, a good linear-scaling relationship should be independent from the energy references used to derive them. Changing references is equivalent to put a constant offset h in the energies of the initial, transition, and final states, as for each reactions the three states share the same stoichiometry, Supplementary Equations 1-3.

$$\text{Initial State:} \quad E'_{\text{IS}} = E_{\text{IS}} + h \tag{1}$$

$$\text{Transition State:} \quad E'_{\text{TS}} = E_{\text{TS}} + h \tag{2}$$

$$\text{Final State:} \quad E'_{\text{FS}} = E_{\text{FS}} + h \tag{3}$$

In the Brønsted-Evans-Polanyi relationships, the contributions of offset h are cancelled during the computation of the reaction and activation energies, as shown in Supplementary Equations 4-5. This means that the BEP relationships are insensitive to the references used.

For Reference 1:

$$\begin{aligned} E_{\text{a}} &= \alpha\Delta E + \beta \\ E_{TS} - E_{IS} &= \alpha(E_{FS} - E_{IS}) + \beta \end{aligned} \tag{4}$$

For Reference 2:

$$\begin{aligned} E'_a &= \alpha\Delta E' + \beta \\ E_{TS} + h - E_{IS} - h &= \alpha(E_{FS} + h - E_{IS} - h) + \beta \\ E_{TS} - E_{IS} &= \alpha(E_{FS} - E_{IS}) + \beta \end{aligned} \tag{5}$$

However, in initial and final state scaling relationships, the offset is not cancelled, as shown in Supplementary Equations 6-7. This means that ISS and FSS are sensitive to the references unless $\alpha = 1$.

For Reference 1:

$$E_{TS} = \alpha E_{IS} + \beta \tag{6}$$

For Reference 2:

$$\begin{aligned} E'_{TS} &= \alpha E'_{IS} + \beta \\ E_{TS} + h &= \alpha(E_{IS} + h) + \beta \\ E_{TS} &= \alpha E_{IS} + \beta + h(\alpha - 1) \end{aligned} \tag{7}$$

Supplementary Note 2: Linear scaling relationships hold in solvated environments Supplementary Figure 1 shows the superposition of the initial- and final state scaling relationships reported in the present study and the values when explicit or implicit solvation is used. These include the C–H breakings on Pt, Pd, and Ru and the O–H breakings on Pd and Pt. The O–H breakings on Ru(0001) were excluded, as these reactions are mediated by the coadsorbed OH* and are barrierless, Supplementary Equations 8-9.

$$CH_3OH^* + OH^* \xrightarrow{\text{Ru(0001)}} CH_3O^* + H_2O^* \quad E_a = 0.00 \text{ eV} \tag{8}$$

$$CH_2OH^* + OH^* \xrightarrow{\text{Ru(0001)}} CH_2O^* + H_2O^* \quad E_a = 0.00 \text{ eV} \tag{9}$$

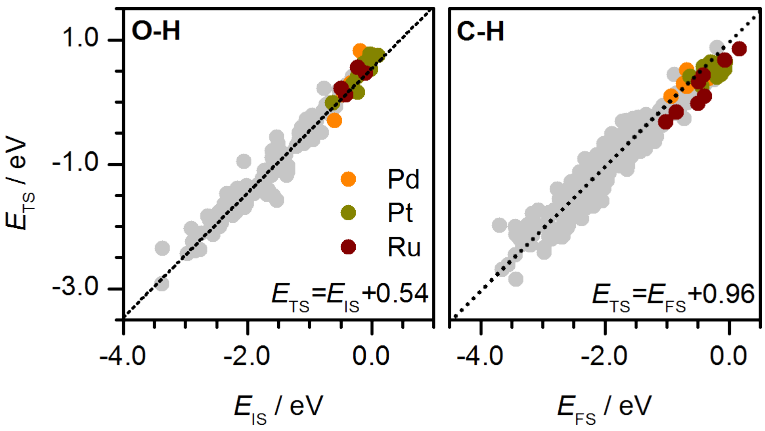

Supplementary Figure 1: **Linear Scaling Relationships under solvation**. Initial-state scaling for O–H and final-state scaling for C–H breaking under all solvation models superposed to the data of Supplementary Figure 3 in the main text (grey) and its scaling line for Pd, Pt, and Ru. The data for solvated environments is shown in colour and it was taken from our results for methanol reported in Ref. [3].

Supplementary Note 3: Pseudo-stationary states in differential reactors Complex reaction networks may have several stationary states depending on the time scale. To exemplify this, let us consider a system where three isomers interconvert with two consecutive reactions, Supplementary Equation 10. R is the reactant, P the desired product, and U an undesired by-product. We used the transition state theory and the Knudsen Supplementary Equations to describe the kinetics of reactions and adsorptions, Supplementary Equation 34 and 32. Their energy profile is defined by Supplementary Figure 2 (a). The formation of U, r_{2d}, is exothermic, but it has an activation barrier is higher than r_{1i} or the desorption of P.

The desorption energy of U is larger that R and P, thus U will be formed at a rate much lower that P and it will poison the surface once formed. Supplementary Figure 2 (b). If this system were tested experimentally, very different behaviours would be seen according to the time scale. Once the reactor is turned on, the desorption rate of P reaches a plateau between 10^1 and 10^5 s, roughly one day. After that, U poisoning is not longer negligible and the desorption rate of P starts do decrease over time. In four months (aprox. 10^7 s) the production of P decreases by two orders of magnitude. Finally, the true stationary state is reached at 10^{10} s, or 320 years! In these conditions, the desorption rate of P is 9 orders of magnitude lower than during the first day. If the steady-state approximation had been included in this microkinetic model, the desorption rate of P would describe a poisoned surface instead of the values observed experimentally, provided that they are done within the 10^1 to 10^5 s time range.

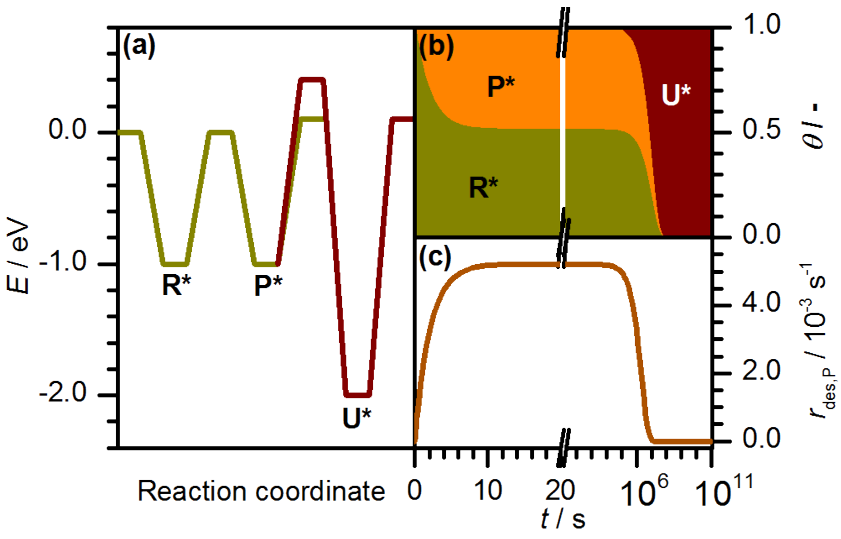

Supplementary Figure 2: **Pseudo-stationary states in a model system**. (a) Energy profile for the model system, Supplementary Equation 10 (a). (b) Surface coverages of R*, P*, and U* as a function of time. (d) Desorption rate of P as a function of time. Reaction conditions: $P_R = 1.0$ atm; $T = 373$ K.

$$R^* \underset{r_{1i}}{\overset{r_{1d}}{\rightleftharpoons}} P^* \underset{r_{2i}}{\overset{r_{2d}}{\rightleftharpoons}} U^* \tag{10}$$

$$r_1 = k_{1d}\theta_R - k_{1i}\theta_P \tag{11}$$

$$r_2 = k_{2d}\theta_P - k_{2i}\theta_U \tag{12}$$

Along the manuscript, it was considered that a system entered in a relevant stationary state if the relative variation of all desorption rates, Supplementary Equation 13, were lower than 10^{-4} s^{-1}.

$$v_i = 2\frac{r_{i,(t+\Delta t)} - r_{i,(t)}}{(r_{i,(t+\Delta t)} + r_{i,(t)})\Delta t} \quad (13)$$

There is few experimental data measuring the catalyst activity over long times. However, it has been found that for the APR of ethylene glycol,[4] after 60 h the reactions are still not in their final stationary state, as the hydrogen production is still slowly going down. The results of our microkinetic model seems to be faster in the initial decay, but still agrees that after 60 h the system has not reached its final stationary state, Supplementary Figure 3.

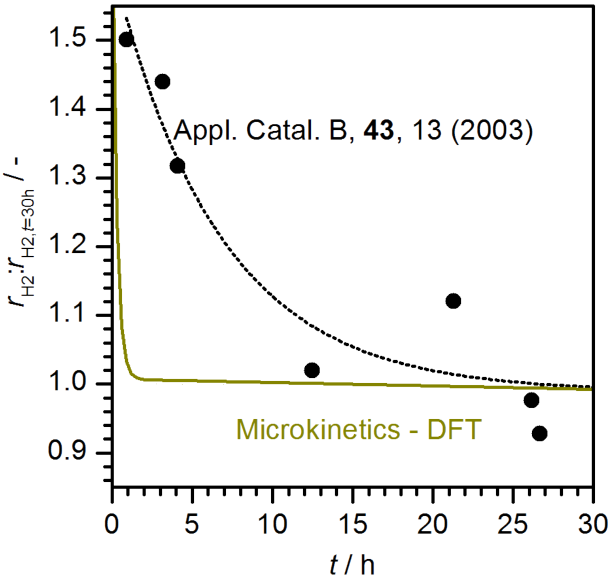

Supplementary Figure 3: **Long equilibration times: Experiments vs. Microkinetic models**. The experimental data, taken from Ref. [4], and the microkinetic model are based on the same conditions: APR of ethylene glycol on Pt at 483 K.

Supplementary Note 4: Coverage effects influences the pseudostationary states

We tested if the long equilibration times we have found, up to two years, may be an artifact from not considering the lateral interactions. To this end, the effect of lateral interactions was quantified following a model analogue to Ref. [5,6], Supplementary Equation 14, applying them on the the direct decomposition of ethanol on Pd(111), Supplementary Figure 4 in the main text. We selected the most abundant reaction intermediates according to Supplementary Figure 4(b) in the main text: CO, CH, CCH_2, and CCH_3. Then, we recalculated their adsorption energies for higher coverages, using supercells with periodicity $\sqrt{3}\times\sqrt{3}-R30°$, 2×2, 3×3, and $2\sqrt{3}\times2\sqrt{3}-R30°$. We considered that their maximum coverage (For $\theta_j = 1$) is around one molecule per three metal atoms. The resulting coefficients for the adsorption energy shift, in eV ML^{-1}, are: κ_{CO} =0.21, κ_{CH} =0.34, κ_{CCH2} =0.36, and

κ_{CCH3} =0.37. These values are in good agreement with a previous study: κ_{CH} =0.35 and κ_{CCH3} =0.43 eV ML^{-1}.[5] As shown in Supplementary Figure 4, shorter times are required to reach the pseudo-stationary states and the final stationary state. However, the times required are within the same order of magnitude. Besides, the surface coverages and the relative desorption rates for the products are not significantly affected.

$$E_j(\theta_j) = E_j(\theta_j = 0) + \kappa_j(\theta_j - \theta_{j0}) \tag{14}$$

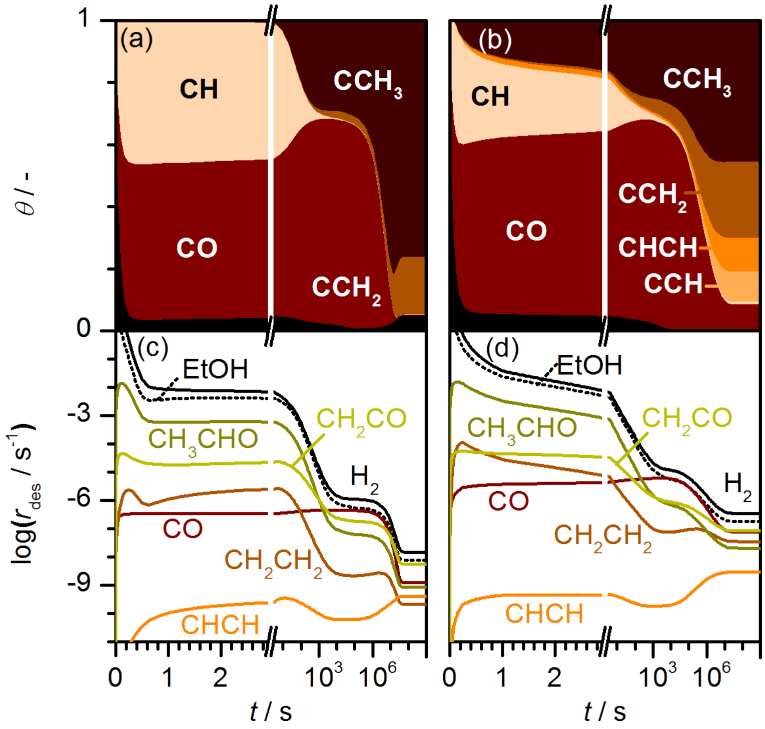

Supplementary Figure 4: **Coverage effects in microkinetic modeling**. (a-b) Surface coverages and (c-d) main desorption products as a function of time. Values on the left panels were obtained through the mean-field approximation and are equivalent to Figure 4. Values on the right panels include coverage effects for CO, CH, CCH_2, and CCH_3.

Supplementary Note 5: ATR is sensible to oxygen pressure

Autothermal reforming may occur on three different regimes depending on the oxygen pressure, as exemplified in Supplementary Figure 5. In the first regime, shown in yellow, the reaction is controlled by O_2, which removes the carbonaceous and CO* poisons. Therefore, the reaction follows a first order kinetics with respect to the oxygen pressure. The productivity keeps increasing until $P_{O_2} \approx 0.060$ atm, at which the system reaches a zeroth-order dependence on P_{O_2}. Above that point, any additional hydrogen produced will be consumed by the excess of oxygen. Finally, at $P_{O_2} \gtrsim 0.095$ atm, oxygen adsorbs faster that it can react.

In this regime, all the adsorption sites are blocked and the surface is not longer active.

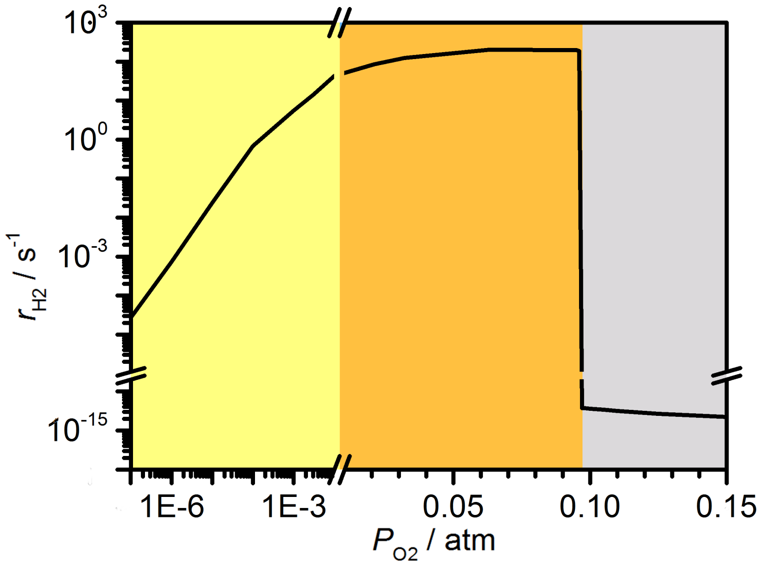

Supplementary Figure 5: **Dependence of ATR on the oxygen pressure**. Hydrogen desorption rate for autothermal reforming of ethanol on Ru(0001) as a function of oxygen pressure. T = 950 K; P_{etOH} = 0.25 atm; $P_{\mathrm{H_2O}} = 1.00 - P_{\mathrm{etOH}} - P_{\mathrm{O_2}}$ [atm]. Yellow and orange regions correspond to first order and zeroth order kinetics. Gray region represents a catalyst poisoned by adsorbed oxygen.

Supplementary Discussion

In the following paragraphs, we deepen the discussion on several topics. First, we describe the C_2 decomposition intermediates and their stabilities. Then, details on the linear scaling relationships for O–H, C–H, C–C, C–O, and C–OH bond scissions are discussed. Finally, the direct decomposition of ethanol and ethylene glycol on Cu, Ru, Pd, and Pt is analysed from the energy profiles.

Adsorption of C_2 molecular fragments on Cu, Ru, Pd, and Pt The complete C_2 dehydrogenation network on Cu, Ru, Pd, and Pt contains 55 species with the stoichiometry $C_2H_yO_z$, y=0-6, z=0-2. The C_1 species are discussed in our previous work.[7] The most stable adsorption structures as well as related transition states are uploaded to our database.[8] Adsorption energies and zero-point energies of intermediates are shown in Supplementary Table 4 and 6. For clarity, these species are classified by the number of oxygen (from 2 to 0) and hydrogen (from 6 to 0) atoms in the structures.

For H saturated structures, they adsorb on the surface weakly with energies of ethylene glycol (-0.56, -0.79, -0.70, and -0.68 eV), ethanol (-0.43, -0.63, -0.52, and -0.51 eV) and ethane (-0.18, -0.18, -0.20, and -0.17 eV) on these four metals (Cu, Ru, Pd, Pt), respectively. On Cu(111), Pd(111) and Pt(111), ethylene glycol adsorbs on the top site with one O atom via lone pair electrons and forms intra-molecular hydrogen bond with the H from the other OH group, the formation of hydrogen bond decreases system energies by 0.14, 0.14 and 0.16 eV, compared with those structures without hydrogen bonds (last two lines in Supplementary Table 6). On Ru(0001), the H-bonded structures are almost degenerate in energy with the one having two OH groups on top sites without intra-molecular hydrogen bonding which is slightly more stable (-0.02 eV). This can be interpreted from the O affinity of Ru, which is higher than those of Cu, Pd, and Pt, Supplementary Table 4. Therefore, the second Ru–O formation energy is nearly equal than the energy to break the intra-molecular hydrogen bond. The interaction between other three metals and O atoms are not strong enough to meet the energies lowered by the hydrogen bond, hence these non hydrogen bonded structures are not energetically favoured on these surfaces.

Upon abstraction of an H from $C_2H_6O_2$, C_2H_5OH and C_2H_6, several intermediates would be obtained. Generally speaking, for isomers with specific H and O atoms, the energy variations from lowest to highest geometries are much smaller on Ru than those on other three metals (6). For example, the variations for $C_2H_4O_2$ ($C_2H_3O_2$) isomers are 1.01, 0.25,

1.38, and 1.57 eV (0.73, 0.42, 1.20, 1.46 eV) on Cu, Ru, Pd, and Pt, respectively, showing the balance between the intra-molecular structure stability and different adsorption ability of these metals towards the intermediates via the atomic adsorption energies (C, H, O) and sites (top, bridge, fcc, and hcp).

For species with five H and two O atoms, OCH_2-CH_2OH is more stable on Cu and Ru while $HOCH-CH_2OH$ is lower in energy while on Pd(111) and Pt(111). The opposite properties between Cu, Ru and Pd, Pt surfaces might also due to the different C and O affinities as stated before. On the surface with stronger affinity towards O atoms, intermediate species prefer the alkoxy structures while on the surfaces with weaker O affinity, the structures with OH groups are energetically preferred. For example, CH_3-CH_2O, CH_3-CHOH and CH_2-CH_2OH contain five H atoms and only one O atom. On Cu and Ru, CH_3-CH_2O and CH_3-CHOH are the most stable and unstable structures, the opposite of Pd and Pt surfaces.

Five $C_2H_4O_2$, five C_2H_4O and two C_2H_4 isomers are obtained. On Cu(111) and Ru(0001), di-O terminal structure OCH_2-CH_2O is the most stable while $HOCH-CHOH$ has the highest energy, showing the opposite sequence on Pd(111) and Pt(111). For C_2H_4O species, the lowest are O terminated CH_3-CHO on Cu(111) and Ru(0001) and OH terminated CH_3-COH on Pd(111) and Pt(111). The highest intermediates have OH terminal, $CH-CH_2OH$ for Cu and CH_3-COH for Ru. The energy between $CH-CH_2OH$ and CH_3-COH is only 0.07 eV, and the energy span is small (0.30 eV) from lowest (CH_3-CHO) to highest (CH_3-COH) structures on Ru(0001), showing the strong adsorption of Ru towards the adsorbates. Pd and Pt share the same highest O terminal structure, CH_2-CH_2O. Binding energies of closed-shell molecules (products in our case) are -0.22, -1.05, -0.67, and -0.50 eV for CH_3-CHO and -0.45, -1.35, -1.23, -1.39 eV for $CH_2{=}CH_2$ ethylene, respectively. On Cu, Pd and Pt, ethylene is more stable than $CH-CH_3$ (ethylidene) and slightly (0.05 eV) lower than it on Ru.

For intermediates with three and two H atoms, because of the low coordination of C atoms in these species, both inter-molecular factors such as forming C–C and C–O double bonds and molecular-surface interaction have significant effects on the system energies. The detail balance of these competing interactions is difficult to disentangle. The ultimate consequence is the lack of energy ordering in the adsorption energy sequence observed for these metals which makes it difficult to locate the most stable and unstable isomers. For example, the lowest $C_2H_3O_2$ structures are $OCH-CH_2O$, $OC-CH_2OH$, $OC-CH_2OH$, and $HOC-CHOH$, while the highest are $HOC-CH_2O$, $HOC-CH_2O$, $OCH-CH_2O$, and $OCH-CH_2O$ on these

metals. On Cu and Pt, di-O and di-OH isomers are the lowest or highest in energy. However, for Ru and Pd, they do not fully follow the empirical rules as the lowest and highest energy isomers are the combination of O and OH terminations. It is interesting to find that the empirical rule can be used again for these highest energy isomers. As they all have the same CH_2O moiety, the other parts, COH and CHO determine the stability, showing that OH terminal species are not favoured on Cu and Ru but favoured on Pd and Pt surfaces. For $C_2H_2O_2$ isomers, they follows the empirical rules well as Cu and Ru prefer the di-O terminated while Pd and Pt prefer the di-OH terminated. For C_2HO_2, O terminated OC−CHO is more stable than OC−COH on Cu and Ru but not on Pd and Pt.

For C_2H_3O, Cu and Pt follow the empirical rules, but all stable and unstable isomers are O-terminated on Ru and Pd. For C_2H_2O, all metal share the same highest energy isomer, C−CH_2O. The most stable one on Cu is CH−CHO. Since the close energies (less than 0.05 eV) between C−CHOH and CH_2−CO, determining the lowest structures on Ru, Pd and Pt is difficult. For C_2HO, CH−CO and C−COH are the most stable and unstable isomers on these metals. Hence, for structures with less H atoms, the empirical rule might not be fully applied to determine the stability of various intermediates.

For C_2H_3 intermediates, C−CH_3 (ethylidyne) is 0.31, 0.68, and 0.56 eV lower in energies than CH−CH_2 (vinyl) on Ru, Pd and Pt but 0.07 eV slightly higher on Cu. Acetylene adsorbs on these metals strongly with binding energies of -1.51, -3.13,-2.41, and -2.58 eV, respectively. However, another C_2H_2 species, C−CH_2 (vinylidene) is 0.14 and 0.21 eV lower than it on Pd and Pt.

Linear scaling relationships for O–H bond scission

For transition states scaling about O–H breaking (34 reactions on each metal, concluding C_1 and C_2), excellent linear scaling relationships are obtained for Cu(111) (FS-like), Ru(0001), Pd(111), and Pt(111) (IS-like), with small mean absolute errors (MAE) of 0.19, 0.12, 0.12 and 0.14 eV, representing the different O–H breaking catalytic abilities of these metals, especially the inert ability of Cu(111) surface.

The overall TSS scaling contains all metals based on IS-like shows a better performance with R^2 and MAE values of 0.96 and 0.17 eV than the FS-like one (0.90 and 0.28 eV), slightly lower (by 0.03 eV) than the reported TSS fitted values of 0.20 eV for dehydrogenation reactions of O–H scissions on various metal surfaces.[9] As O–H breaking has more FS-like character on Cu(111) surface, it deteriorates whole FS-like TSS relations with larger deviation points. For example, there are totally 26 reactions above the scaling line larger than

0.20 eV, among them, 15 reactions are on Cu(111) surface. H abstraction from C−CH_2OH, OC−CHOH, HOC−COH, COH, HOCH−CH_2OH, CH−COH, EG, CH_3OH and OC−COH are 0.61, 0.54, -0.50, -0.42, -0.34, -0.31, -0.27, -0.26, and -0.25 above the scaling lines.

A similar behavior for Cu is also reported by Liu and Greeley[10] when they extrapolate the binding energies of glycol decomposition species from Pt(111) to those on Cu(111). Therefore, the accuracy would be improved by isolating Cu from other three metals. To verify our assumption, only reactions on Ru, Pd, and Pt surfaces were considered and ideal R^2 and much lower MAE values of 0.94 and 0.14 eV were obtained. Among the total 102 reactions, 11 and 10 reactions are above and below the scaling line with deviation energies larger than 0.20 eV. For points above the line, adsorption structures (in the form of C-CH_xOH and CH-CH_xOH which are similar to some points on Cu(111)) and hydrogen bonds have large effects on increasing the TSs' energies. For example, H abstractions from C−COH on Ru, C−CH_2OH on Pd and Pt, and CH−CHOH on Pt are above the scaling line with respective energies of 0.25, 0.54, 0.41 eV, and 0.27 eV. It is the upright adsorption ISs' geometries on surfaces that makes it difficult for CHOH and CH_2OH moieties to bend to interact with surface.

Also, we observed the large structure reorganization from IS to TS in reaction OC−COH → OC−CO on Ru(0001). To fulfill the interaction between hydroxy group and the surface, intra-molecular hydrogen bond elimination by the rotation of COH moiety is necessary and herein increases the TS's energy to 1.01 eV, much higher than the average O–H breaking barrier of 0.51 eV on Ru(0001) and this reaction is 0.45 eV above the line. The distortion or reorganization from IS to TS greatly affects their similarity which TSS relations depend on within one specific reaction type.

H bonds would also increase the TSs' energies by another way, the abstraction of H between two O atoms which hydrogen bond forms normally needs more higher barrier (0.00-0.30 eV) than those H abstractions from terminal OH groups without forming hydrogen bonds. For instance, when hydrogen bonds are formed between two O atoms in OCH−CHOH on Pt and OCH_2−CH_2OH on Pd, the H abstractions are 0.25 and 0.43 eV above the scaling line. Furthermore, hydrogen bond formed far from the surface might increase the transition energies by preventing the bending of those intermediates. For example, H abstraction from HOC−COH on Ru are 0.39 eV above the scaling line.

For the 10 points below the scaling line, two of them (EG and HOCH−CHOH on Ru) are because of the intramolecular hydrogen bonds and the strong O–H catalytic activity of Ru. The others are the H abstraction from COH moiety, such as O–H breaking from CH_3−COH

on Ru (0.24 eV) and Pt (0.60 eV), HOC−CH_2O on Pd (0.30 eV) and Pt (0.33 eV), and HOC−CH_2OH on Pt (0.48 eV), demonstrating that O–H preferably breaks from R−COH species with lower barriers.

On Cu(111), the MAE for IS-like model is 0.22 eV. There are a few points leading to the larger MAE than other surfaces. H abstractions from OH groups in C−CH_2OH, OC−CHOH, and HOC−COH are 0.63, 0.45, and 0.42 eV above the scaling line. For C−CH_2OH, it corresponds to the higher barrier of 1.41 eV. As with the later two reactions, the hydrogen bond increases the TS energies. As we discussed before, the abstraction of H between two O atoms usually needs more energy than side H atoms. And the energy difference varies up to around 0.30 eV. Both OC−CHOH and HOC−COH have intra-molecular hydrogen bonds when they bind to the surface. When O–H bond breaks in the transition state, additional energy is needed to break the hydrogen bond, increasing the activation energies to 1.28 and 1.25 eV, respectively. As a consequence, they are above the scaling line by 0.44 and 0.42 eV.

On the contrary, reaction barriers can also be reduced by adding an intra-molecular hydrogen bond in the transition state. For example, barrier for O–H scission from HOCH−CHOH is 0.48 eV and below the regression line by 0.37 eV. Again, it is interesting to find that those points below scaling line with large energies are R−COH species. For example, O–H from breaking from CH_3−COH, HOC−CH_2OH, C−COH, and HOC−CH_2O are 0.56, 0.49, 0.32, and 0.32 eV lower than predicted energies. All these reactions have relative lower barriers and exothermic energies of 0.43 (-1.10), 0.24 (-0.51), 0.32 (-0.55), 0.50 (-0.50) eV.

Linear scaling relationships for C–H bond scission

For C–H bond cleavages (80 reactions on each metal), From Supplementary Table 8, it is clear to see that final state scaling exhibits better behaviours than ISS both for the overall and individual surfaces. The overall MAE of FS-like relations is 0.18 eV, and 0.18, 0.17, 0.12, and 0.15 eV for Cu, Ru, Pd, and Pt separately, much lower than reported average value of 0.29 eV.[9] H abstraction from C would enhance the interaction between C and the surface and affect the intermediate adsorption geometries and intra-molecular structures on the other hand. Therefore, unlike IS-like results of H abstraction from OH, C–H scission generally has more FS-like character than IS-like due to the larger reorganization effects, even though the average activation energies of C–H (0.94, 0.37, 0.49, 0.56 eV) and O–H (0.82, 0.52, 0.59, 0.54 eV) are close to each other on these metals.

Two main factors: unbalance adsorption structures of IS and re-optimization during the reaction lead to the deviation points. There are 25 dots above the line by more than 0.30

eV, we found 13 C–H scission reactions have IS adsorption structures in the form of CH_x-CH_y-OH_z, x=0,1, y=1,2, z=0,1. When there are few H atoms binding to C, geometries of these species are predominately determined by the CH_x parts which usually bind to the fcc or hcp sites. Hence, these species will undergo different mechanisms to proceed the C–H breaking from the other side moiety via tilted or rotated TS configurations. For example, C–H scissions from CH_y-OH_z have deviation energies of 0.68 (C−CHO), 0.61 ($CH-CH_2O$), 0.44 ($C-CH_2O$), and 0.33 eV ($C-CH_2OH$) on Cu(111), 0.40 eV ($CH-CH_2O$) on Pd(111), 0.34 eV (CH−CHO) on Ru(111), 0.44 ($C-CH_2O$), 0.44 (CH−CHO), 0.38 ($CH-CH_2O$), and 0.33 eV ($CH-CH_2OH$) on Pt(111). Deviations from Cu and Pt are more larger than those from Ru and Pd.

Re-optimization cases usually goes along with the increasing or changing binding atoms between two C sides as well as the eliminating intra-molecular hydrogen bonds, that is the structure unbalance brought by the reaction process. For instance, OCH−CHO binds on Pd in a planar way with C–C bond center and two O atoms on the top sites. During the H abstraction on OCH−CHO, the distance between the non-reacting CHO moiety and the surface increases, leaving C–O bond in the reacting side parallel to the Pd–Pd bond. Then, after the C–H breaks, the CHO moiety will go back to interact with the surface with O atom after a C–C rotation. This reaction is 0.30 eV above the scaling line. In reaction $OCH-CH_2OH \rightarrow OC-CH_2OH$ (3-12) on Pd, we observed the elimination of hydrogen bond from IS to TS and FS. In IS, it adsorbs on Pd surface with C and O from CHO, and the other OH on three top sites. The hydrogen bond length is elongated from 2.258 in IS to 2.886 Å in TS, after TS, structure re-optimized to a linear FS structure and eliminated the hydrogen bond. H bond elimination are also exist in reactions $OCH-CHOH \rightarrow OCH-COH$ and $HOCH-CH_2O \rightarrow HOC-CH_2OH$.

There are 52 calculations below the scaling line larger than 0.20 eV, they are 30, 16, 3, and 2 reactions on Ru, Cu, Pt, and Pd, respectively, showing that the scaling exist an obvious metal dependence. It is because of the much lower reaction barriers of these reactions on Ru, compared with those on other surfaces. Ideal scaling relations are obtained with fewer number of large deviations points on Pd and Pt surfaces. Therefore, the intra-molecular unbalance brought by the differences of coordination numbers or binding atoms (whether there is O atom or not) from each C side would have significant effects on the scaling behaviours as the reactions from species containing one O atom contribute to the deviations significantly. And reasonably grouping the reactions based on the initial structures would improve the accuracy of TSS.

Linear scaling relationships for C–C bond scission

As shown in Supplementary Table 8, the overall correlation for C–C cleavage shows more FS-like character with MAE of 0.27 eV and R^2 value of 0.95, lower than the previously reported 0.35 eV.[11] Separately, on Cu, Pd, and Pt, FS based scalings are much better than IS based while on Ru, results are improved slightly from IS to FS (MAE: from 0.23 to 0.22 eV, R^2: from 0.87 to 0.90). The MAEs are 0.19, 0.22, 0.21, and 0.26 on Cu, Ru, Pd, and Pt, respectively. Compared with the overall scalings, lower MAE and close R^2 values are obtained on individual metal surfaces, illustrating the metal dependence of TSS correlations. When Cu was removed from the data list, all relationships (BEP, ISS and FSS) become slightly less accurate that those from the full reaction list. As can be seen from Supplementary Table 12, the overall scaling deviations are also metal dependence. Points above the scaling line are related with reactions on Pd and Pt while the dots below line are mainly about reactions on Cu and Ru. For C-C breaking on these four metals, it usually proceeds with lower barriers when there has few H atoms (1,2 and 3) in the species (late dehydrogenation levels). Among these species, R−CO intermediates have lowest barriers and high exothermic energies, followed by the R−COH, R−CH, and R−CHO species. In the TSS relations about C–C cleavage, there are three kinds of species which deteriorate the scalings, saturated molecules at the beginning dehydrogenation level such as CH_3-CH_3, CH_3-CH_2OH, and EG, C−R species in the middle and final levels, and the species in final dehydrogenation steps species, $C_2H_xO_y$ (x=0,1 and y=1,2). For example, C–C scissions from CH_3-CH_3 on Cu, Ru, Pd, and Pt are 0.78, 0.93, 0.95 and 1.43 eV above the scaling line, along with other large deviation C–C breaking reactions, CH_3-CH_2OH (0.26, 1.50, 0.76, and 0.92 eV), C−C (0.63, 0.58, 0.79, 0.59 eV) and ethylene glycol (0.72 and 1.18 eV on Pd and Pt). Those huge deviations enlarge the mean absolute errors and decrease the precision of TSS relations. As C–C bond breaks in the late steps easily with H stoichiometry of two and three, C–C scission from the first and last steps can be ignored to improve the scaling accuracy. In the middle levels, because of the strong binding between C and the surface, C−R species usually take the upright structures, resulting in high barriers. Therefore, C–C breaking reactions at the beginning and last steps are not included in scaling results. And the MAEs (R^2) are improved from 0.39 (0.90) to 0.34 eV (0.91) for ISS and from 0.33 (0.93) to 0.27 eV (0.95). For ethanol decomposition on Ru(0001), Ferrin *et al.*[12] obtained the best scaling relationship for C–C and C–O dissociation on Ru(0001) with Supplementary Equation of $E_{\mathrm{TS}} = 0.88 * E_{\mathrm{FS}} + 1.07$ with R^2 of 0.98. Our results have the Supplementary Equation of $E_{\mathrm{TS}} = 0.96 * E_{\mathrm{FS}} + 1.12$ for C–C scission in ethanol and ethylene glycol decomposition sets on Ru(0001). This can

be interpreted by the different data sets used in the scaling. However, on these four metals, the overall results are $E_{\text{TS}} = 0.79E_{\text{FS}} + 0.96$. As these four metals have different catalytic properties on C–C bond scission, scalings separated by the metals are necessary to achieve better results and accuracy.

Linear scaling relationships for C–O bond scission

For C–O scission, the remaining CH_x (x=0,1,2) side after C–O breaking interacts strongly with the surface, while the interaction of the another moiety with the surface is reduced, forcing the other part move far away from the surface by weakening the C–surface or O–surface bonds. On the other hand, C–O involves a strong rearrangement from IS to TS, either by tilting or bending. Although these distortions facilitate the C–O breaking, they also increase the TS energies dramatically. Herein, structure reorganizations would be significant in the deviations points. For example, OCH−CHO → CH−CHO on Cu(111), the IS has a planar geometry and is slightly tilted parallel with the surface. In TS, one CHO is prone to the surface to achieve the C–O bond breaking and the other part goes into the gas phase. After C–O bond scission, the product CH−CHO adsorbs on the surface with C atom from CH moiety. On Pd(111), CH_2−CO adsorbs on the surface with two C atoms. After C–O bond scission, C−CH_2 adsorbs with only one C atom, pushing the CH_2 part into the gas phase. The other factor that leads to the higher barrier is the upright adsorption structures, such as C–O breaking reactions from C−CO and C−CHO. In addition, the C–OH bond rotation also can not be ignored in the transition calculations as it is the first step to facilitate the OH interaction with surface which is followed by the C–OH breaking. From Supplementary Table 7, C–O(terminal) breaking has higher average activation energy than C–OH with barriers of 1.65 vs 1.16 eV (Cu(111)), 1.11 vs 0.83 eV (Ru(0001)), 1.82 vs 1.43 eV (Pd(111)), and 1.95 vs 1.50 eV (Pt(111)), reflecting the C–O activation sequence of Ru(0001) > Cu(111) > Pd(111) > Pt(111) and the large difference of breaking C–O(terminal) and C–OH bonds. On the thermodynamic aspect, C–OH breaking is more endothermic than C–O in alkoxy on Ru, Pd, and Pt surfaces. On Cu, C–OH is 0.66 eV exothermic more than C–O scission. Different behaviours from R−CH_xO to R−CH_xOH originate in the changing terminal O to OH groups. For all of the reactions from R−CH_xO to R−CH_xOH, the decreased average activation barriers on these metals confirm that C–OH breaking is more easier to take place on these metals than C–O(terminal) breaking, especially on Ru and Cu. In other words, the C–O bonds could be activated by changing the terminal O into hydroxyl groups. Moreover, much lower barriers and exothermic energies on Ru(0001) shows that Ru has a superior

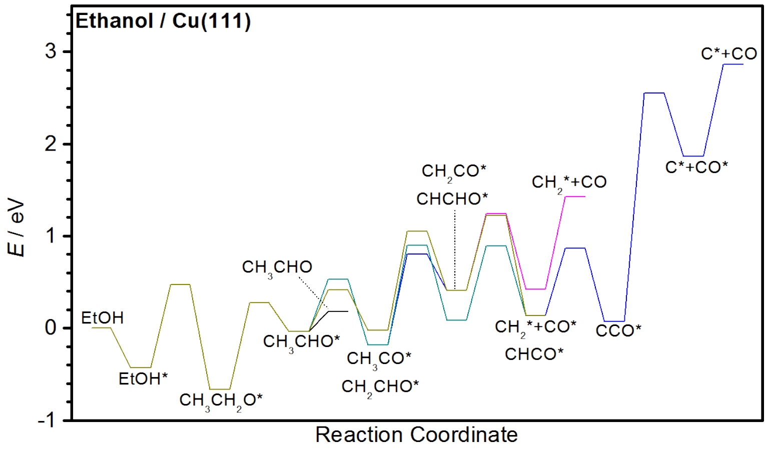

Supplementary Figure 6: **Energy profile for ethanol decomposition on Cu.**

performance in catalyzing C–O bond scission than other metals, followed by Cu, Pd and Pt.

Therefore, the scaling relations would be improved if these reactions are effectively classified or grouped based on some specific reaction types or adsorption structures. In this study, C–O scissions are divided into two groups, C–O (terminal) and C–OH breaking. Results show that scaling of C–O (terminal) scission from alkoxy groups based on FS energies have been significantly improved with with higher R^2 and lower MAE values. However, only a little improvement for C–OH breakings are observed, demonstrating the difficulty in predicting C–OH bond scissions.

Ethanol decomposition on Cu(111)

The energy profiles of ethanol decompositions on Cu, Ru, Pd, and Pt on closed-packed surfaces are shown in Supplementary Figure 6. Only the most energetically feasible reactions in each step, those within 0.30 eV higher than the reaction with lowest activation energy, are shown. Reaction numbers in the Supplementary Table 7 are used for discussion and the geometries can be found in accordance with the reactions uploaded in ioChem-BD database.[13] On clean Cu(111), ethanol decomposition starts by the O–H bond breaking (reaction 1-07 in Supplementary Table 7) to produce ethoxy, which has an energy barrier (reaction energy) of 0.90 (-0.24) eV. However, provided that its adsorption energy is just -0.43 eV, ethanol would prefer to desorb rather than react. The other possible reactions (from 1-08 to 1-11), α-H, β-H abstraction, C–OH, and C–C scissions, have much higher activation energies, 1.23, 1.34, 1.45, and 2.46 eV, which corresponds to reaction energies 0.72, 0.62, 0.11, and 1.29 eV. respectively. From ethoxy, the α-H abstraction (2-23) to produce acetaldehyde has the lowest activation barrier, 0.94, and is endothermic by 0.63 eV. Acetaldehyde easily desorb to the gas phase, because its binding energy (0.22 eV) is lower than the barriers of further α-H

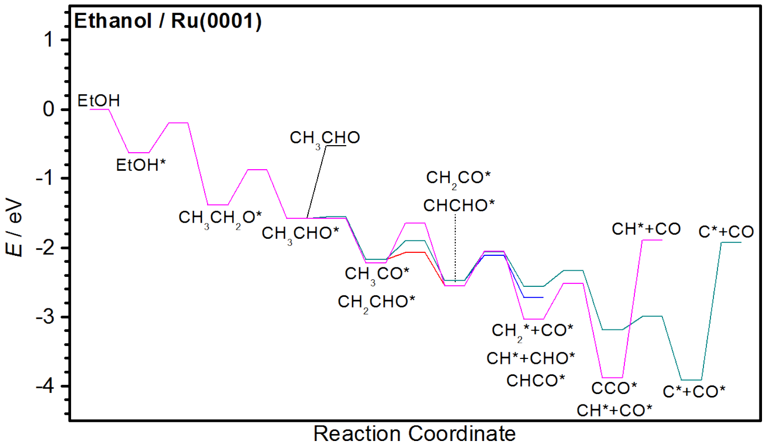

Supplementary Figure 7: **Energy profile for ethanol decomposition on Ru.**

(3-46) and β-H (3-45) abstractions, or rehydrogenation to ethoxy, 0.45, 0.56, and 0.31 eV respectively. This behaviour is similar to methanol decomposition on Cu(111)[7] that leads to formaldehyde as main desorption product, and agrees with a previous theoretical study.[14] Therefore, ethanol decomposition on Cu(111) follows: $CH_3{-}CH_2OH \rightarrow CH_3{-}CH_2O \rightarrow CH_3{-}CHO$. If the first dehydrogenation occurs on the β-H (1-08), it may be followed by an α-H (2-16) or a C–OH (2-18) bond breaking, to form either ethenol, or ethene and OH, with activation (reaction) energies of 0.68 (-0.08) and 0.42 (-0.51) eV respectively. After two further H abstraction steps from acetaldehyde, two intermediates are produced, $CH_2{-}CO$, from one β-H and one α-H abstractions, and CH$-$CHO from two consecutive C-H_β scissions. From $CH_2{-}CO$, C–C breaking (E_a = 0.83 eV in 5-26) competes with C–H scission (0.81 eV in 5-27), however, it presents a thermoneutral reaction energy of 0.02 eV, compared with -0.27 eV for C–H. From CH$-$CHO, α-H abstraction is both thermodynamically (0.05 eV) and kinetically (0.80 eV) preferred over the other reactions. Hence, CH$-$CHO and $CH_2{-}CO$ share the same product from C–H dissociation. Thereafter, the reaction pathway goes as CH$-$CO $\rightarrow$ C$-$CO $\rightarrow$ C + CO (6-13 and 7-03), with barrier and reaction energies of 0.73 (-0.06) and 2.48 (1.79) eV, respectively. The highest C–C bond breaking barrier lies in the upright adsorption structure of C$-$CO intermediate in which there is no interactions between CO and the surface. Due to this, C$-$CO stays adsorbed without reacting. The intermediate species with most possibility of breaking C–C bond is $CH_2{-}CO$. However, it competes with C–H scission and other reactions which lead to C$-$CO.

Ethanol decomposition on Ru(0001)

At the beginning of ethanol decomposition on Ru(0001), β-H abstraction (1-09) and O–H scission (1-07) complete with similar barriers, 0.42 and 0.43 eV. However, O–H breaking is

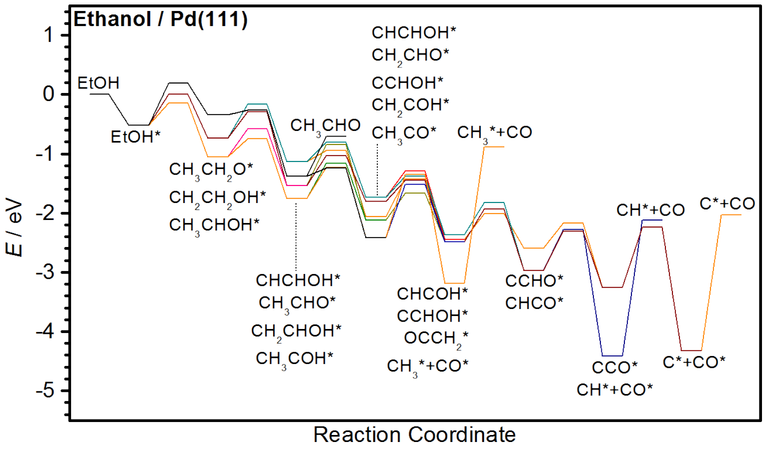

Supplementary Figure 8: **Energy profile for ethanol decomposition on Pd.**

preferred thermodynamically, with a reaction energy of -0.76 eV, compared to -0.40 of β-H abstraction. According to previous studies on the competition of O–H and C–H scission,[7,15] O–H scission is assisted by H-bonds, either intermolecular or intra-molecular, therefore, the whole reaction starts from the ethoxy formation, Supplementary Figure 7, where it becomes a common on-surface species. From ethoxy, C–O and C–C breakings are more energy demanding than α-H and β-H abstraction (Supplementary Table 7), and α-H (2-23) is preferred over β-H (2-22) abstraction both kinetically (0.51 vs. 0.64 eV) and thermodynamically (-0.19 vs -0.01 eV), in good agreement with Chiu *et al.*[16] Unlike Cu(111), acetaldehyde binds strongly on Ru(0001), ($E_{ads} = -1.05$ eV). The further α-H (3-46) and β-H (3-45) dehydrogenation, to produce CH_3-CO and CH_2-CHO, are exothermic and barrierless. However, the following decompositions proceed too fast, and the surface becomes rapidly covered by a plethora of species. Because of the lack of empty spaces, acetaldehyde desorbs rather than decompose. From CH_2-CHO, α-H abstraction (4-42) to CH_2-CO is slightly preferable over β-H abstraction (4-43) both kinetically (0.10 vs. 0.27 eV) and thermodynamically (-0.41 vs. -0.33 eV), sharing the same product of β-H abstraction from CH_3-CO. From CH_2-CO the reaction follows mainly as: $CH_2-CO \rightarrow CH-CO \rightarrow CH + CO$ with energies of 0.51 (-0.50) in 5-27 and 0.52 (-0.87) eV in 6-12, respectively. C–C breaking (5-38) also occurs easily from $CH-CHO$ with activation (reaction) energies of 0.41 (-0.10) eV.

Ethanol decomposition on Pd(111)

The ethanol decomposition on Pd has a complex reaction pathway, because it has many possible intermediate species coming from different dehydrogenation routes that have low activation energy, Supplementary Figure 8. When decomposition starts, both α and β C–H dehydrogenations are preferred over O–H, to yield CH_3-CHOH (1-08), CH_2-CH_2OH (1-09),

and $CH_3{-}CH_2O$ (1-07) with activation (reaction) energies of 0.37 (-0.47), 0.52 (-0.16), and 0.71 (0.24) eV, respectively. On the second step, α-H abstractions are thermodynamically and kinetically preferred to produce $CH_3{-}COH$ (2-27), $CH_2{-}CHOH$ (2-16), and $CH_3{-}CHO$ (2-23). After the third dehydrogenation, the most stable intermediate is acetyl $CH_3{-}CO$. The activation energies for further C–H, C–C, and C–O breakings (from 4-54 to 4-56) to produce $CH_2{-}CO$, $CH_3{-}C{+}O$ and $CH3{+}CO$, are 0.90, 1.85, and 1.07 eV respectively. However, the C–C breaking is more exothermic (-0.77 eV) than the C–H breaking (-0.07 eV). The C–C breaking can easily take place on the middle and highly dehydrogenated $CH{-}CH_2O$ and $CH{-}CO$, where the barriers are just 0.63 (4-45) and 0.69 eV (6-12) respectively. For $CH{-}CO$, C–H and C–C scissions have similar activation energies (0.66 vs. 0.69 eV), but a huge difference in reaction energies (-0.23 vs -1.36 eV), so the C–C breaking will be thermodynamically preferred. C–C breaking from $C{-}CO$ is also highly exothermic (-1.00 eV) but has a high barrier of 1.02 eV. As H-bonds may affect the O–H and C–H bond breaking mechanism, O–H competes with C–H breaking in the early dehydrogenation steps.[7,15] If the ethanol decomposition starts by the dehydrogenation from OH to for an alkoxy intermediates (1-07), the downstream C–H breakings will be significantly affected in ethanol decomposition pathways. For example, for ethoxy ($CH_3{-}CH_2O$), the α–H abstraction (2-23) has the smallest barrier (0.08 eV) and is the most exothermic (-0.99 eV) among all the C–H breaking reactions from C_2H_5O species. For C_2H_4O intermediates, α–H abstraction from $CH_2{-}CH_2O$ (3-38) is barrierless and highly exothermic (-1.06 eV). On $CH_3{-}CHO$ (acetaldehyde), α–H abstraction has a small barrier of 0.14 eV, and releases -0.98 eV. If ethanol decomposition starts by the O–H bond breaking, the reaction path becomes much simpler: $CH_3{-}CH_2O \rightarrow CH_3{-}CHO \rightarrow CH_3{-}CO \rightarrow CH_2{-}CO \rightarrow CH{-}CO \rightarrow CH + CO$.

Ethanol decomposition on Pt(111) The reaction profile for ethanol and ethylene glycol decomposition on Pt(111) is similar to that on Pd(111). Again, C–H bond breaking is accelerated by the formation of alkoxy intermediates. α C–H abstraction from $CH_3{-}CH_2O$ (2-23), $CH_2{-}CH_2O$ (3-38), and $CH_3{-}CHO$ (3-46), and O–H breaking from $CH_3{-}COH$ (3-50) are barrierless and exothermic reactions, $\Delta E = -0.72$, -0.64, -0.90, and -0.27 eV respectively. Two reaction paths can be envisaged from Supplementary Figure 9, $CH_3{-}CH_2OH \rightarrow CH_3{-}CHOH \rightarrow CH_3{-}COH \rightarrow CH_3{-}CO \rightarrow CH_2{-}CO \rightarrow CH{-}CO$ and $CH_3{-}CH_2OH \rightarrow CH_3{-}CH_2O \rightarrow CH_3{-}CHO \rightarrow CH_3{-}CO \rightarrow CH_2{-}CO \rightarrow CH{-}CO$. Both go through the acetyl intermediate, $CH_3{-}CO$, which can be form after three dehydrogenations with low barriers. A significant difference with Pd is that C–C scission is only favoured in later de-

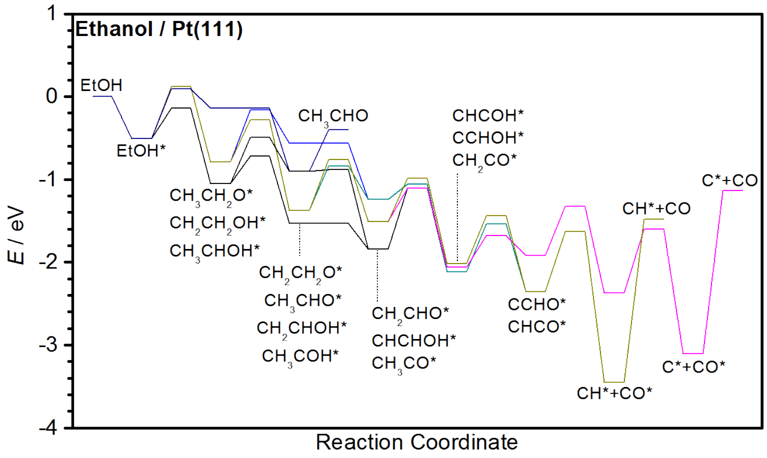

Supplementary Figure 9: **Energy profile for ethanol decomposition on Pt.**

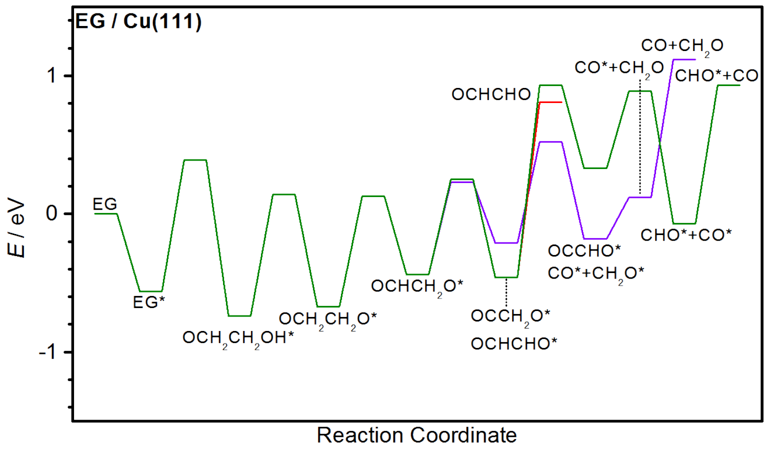

Supplementary Figure 10: **Energy profile for ethylene glycol decomposition on Cu.**

hydrogenation stagesfrom CH$-$CO, but not from CH_3-CO. CH$-$CO has the lowest barrier for which C–C breaking (0.73 eV for 6-12) can compete to C–H scission (1.04 eV for 6-13).

Ethylene glycol decomposition on Cu(111) The energy profiles of ethylene glycol decompositions on Cu, Ru, Pd, and Pt on closed-packed surfaces are shown in Supplementary Figure 10. Only the most energetically feasible reactions in each step, that are within 0.30 eV the reaction with lowest activation energy. For ethylene glycol on Cu(111), energy barriers (reaction energies) for O–H (1-03), C–H (1-02), and C–O cleavage (1-04) are 0.95 (-0.18), 1.29 (0.58), and 1.63 (-0.04) eV, respectively, showing than an O–H bond breaking is kinetically and thermodynamical preferred. However, the adsorption energy of ethylene glycol is only -0.56 eV, so most of the adsorbed molecules will prefer to desorb rather than react. At this early stage, C–C bond cleavage is unlikely to proceed as no strong interaction between two C sides and the metal surface to weaken the C–C bond. The second decomposition step is another O–H bond breaking (2-11) which produces OCH_2-CH_2O (1,2-dioxyethylene).

$OCH_2{-}CH_2O$ binds to the surface with two O atoms firmly attached to fcc sites, leaving the two C atoms far from the surface. When taking CH_2O gas phase energy as the reference, the formation energy of $OCH_2{-}CH_2O$ is -2.60 eV. This is equivalent to -1.30 eV per CH_2O, which is much larger than the weak formaldehyde adsorption energy of -0.36 eV. Because of the stability of $OCH_2{-}CH_2O$ and the hindered interactions between the C and surfaces, breaking the C–C bond (3-33) is highly energy demanding, with a barrier of 2.37 eV. Compared with C–C cleavage, C–H bond scission (3-34) to form $OCH{-}CH_2O$ is less energy demanding, 0.80 (0.23) eV, followed by the H abstraction from either the CH_2O moiety to produce $OCH{-}CHO$ (4-38), 0.69 (-0.02) eV, or the H abstraction from OCH to produce $OC{-}CH_2O$ (4-37), 0.67 (0.23) eV. The first possible product, $OCH{-}CHO$ (glyoxal), adsorbs on Cu(111) maintaining a planar structure typical of gas, but is slightly tilted with the two O atoms bound to the surface. Only the cis isomer is stable, so the interactions between H and surface are unlikely, resulting in a high C–H bond scission (5-35) barrier of 1.39 eV. The C–C bond breaking (5-34) is unlikely, with and activation energy of 1.56 eV. However, the adsorption energy of glyoxal on Cu(111) is -1.27 eV, so glyoxal desorption and further dehydrogenation are competitive processes. Indeed, glyoxal is produced in industry by ethylene glycol oxidation on Cu-based catalysts.[17] For the other $C_2H_2O_2$ isomer, $OC{-}CH_2O$, C–C cleavage (5-22) to produce CO and CH_2O has the lowest activation barrier, 0.73 eV, and competes the C–H dissociation (5-23) to produce $OC{-}CHO$, 0.87 eV. For the $OC{-}CHO$, C–C bond breaking has a lower barrier (0.56 eV) than C–H (0.64 eV) and C–O (1.31 and 1.60 eV from OCH in 6-11 and CO moieties in 6-10), and the further OCH dehydrogenation to CO (9-10) has a very low a barrier, 0.11 eV, therefore, the decomposition of $OC{-}CHO^* \longrightarrow CO^* + CHO^* \longrightarrow 2\,CO^* + H^*$. An important lateral path, even if kinetically unfavoured, comes from C–O and C–OH breaking. The O^* hydrogenation to OH^* is exothermic (-0.49 eV) and has a low barrier (0.90 eV). However, the rehydrogenation of OH to form water (8-08), $OH^* + H^* \longrightarrow H_2O^*$ presents a barrier of 1.21 eV and is slightly endothermic (0.18 eV).

Thus, ethylene glycol decomposition on Cu(111) starts by two O–H bond cleavage to produce $OCH_2{-}CH_2O$, followed by two C–H bond scissions to produce $OCH{-}CHO$ or $OC{-}CH_2O$. While glyoxal prefers to desorb rather than react, $OC{-}CH_2O$ mainly experiences a C–C breaking followed by the codesorption of CO and CH_2O.

Ethylene glycol decomposition on Ru(0001) The energy profile for ethylene glycol decomposition on Ru(0001) is shown in Supplementary Figure 11 There are two possible conformations for ethylene glycol on Ru(0001) that are degenerated in energy, with a differ-

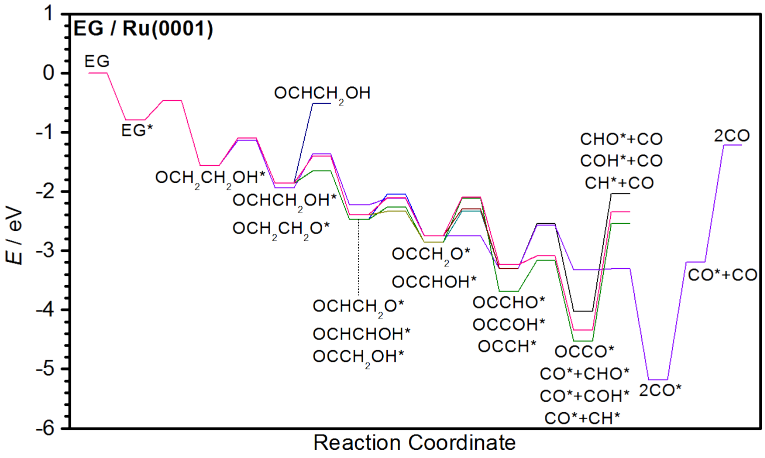

Supplementary Figure 11: **Energy profile for ethylene glycol decomposition on Ru.**

ence of just 0.02 eV, with and without intramolecular H-bond respectively. We calculated the reactions starting from these two structures to ensure that the reaction is correctly described. Our results show that whether the intramolecular hydrogen bond is present or not, O–H bond scission is preferred over C–H and C–O cleavages (Supplementary Table 7). The activation (reaction) energies for the conformations with and without H-bond are 0.28 (-0.81) in 1-03' and 0.32 (-0.80) eV in 1-03, respectively. After the O–H breaking, the O that lost its H goes to an fcc site, and both initial structures produce the same product, $OCH_2{-}CH_2OH$. In the second decomposition step, two H abstractions from O–H group (2-11) to produce $OCH_2{-}CH_2O$ (1,2-dioxyethylene)and from the CH_2O moiety (2-10) to produce $OCH{-}CH_2OH$ (glycolaldehyde), are competitive both kinetically and thermodynamically, with barriers (reaction energies) of 0.43 (-0.39) and 0.47 (-0.31) eV, respectively. Compared with them, C–H cleavage from CH_2OH moiety (2-09) in $OCH_2{-}CH_2OH$ is unlikely due to its higher barrier, 0.66 eV, and its less exothermic reaction energy, -0.20 eV. C–C (2-08), C–O (2-12), and C–OH (2-13) cleavages from $OCH_2{-}CH_2OH$ has high barriers of 1.92, 1.74, and 1.78 eV and therefore are unlikely to happen. $OCH_2{-}CH_2O$ is very stable on Ru(0001). The formation of 1,2-dioxyethylene is also reported from DFT calculation of ethylene glycol decomposition on Ni/Pt bimetallic catalyst[18] and on a Mo_2C surface.[19] The $OCH_2{-}CH_2O$ decomposition continues by an C–H abstraction (3-34) to produce $OCH{-}CH_2O$, $E_a = 0.58$ eV, which is preferred over C–C (3-33) and C–O (3-35) decompositions, $E_a = 1.65$ and 1.00 eV, respectively. Then, the $OCH{-}CH_2O$ further experiences two barrierless C–H breakings (4-37 and 5-23), $OCH{-}CH_2O \longrightarrow OC{-}CH_2O \longrightarrow OC{-}CHO$ with 0.11 (-0.54) and 0.01 (-0.58) eV. After that, $OC{-}CHO$ undergoes a C–C bond breaking with energies of 0.73 (-0.77) eV and C–H dissociation 0.70 (0.07) eV. After H

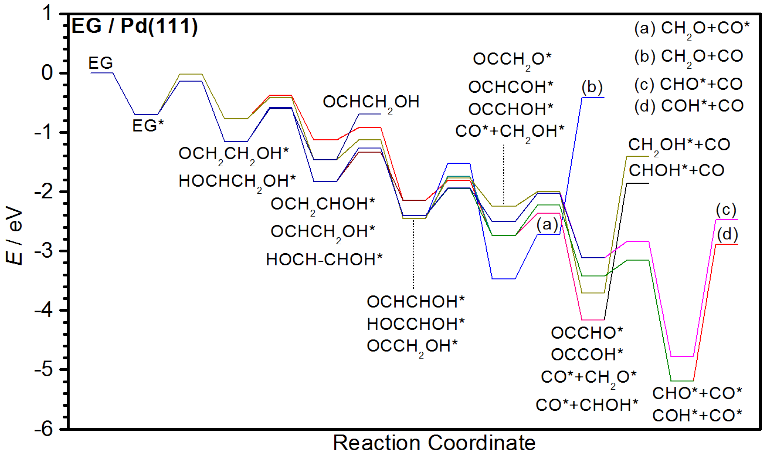

Supplementary Figure 12: **Energy profile for ethylene glycol decomposition on Pd.**

abstraction from OC−CHO, the products, OC−CO, would be followed by the C–C scission spontaneously to produce two CO. For OCH−CH_2OH from 2-10, both C–H scission from OCH (3-13) and CH_2OH moiety (3-12) need low barriers of 0.21 (-0.64) eV and 0.45 (-0.56) eV to overcome. It is expected that the reaction of C–C cleavage from OC−CH_2OH to produce CO (4-07) should take place easily. However, this reaction requires high energy barrier 0.73 (-0.50) eV while the C–H (4-08) and O–H (4-09) bond scissions have much lower barrier of 0.21 (-0.41) and 0.43 (-0.30) eV, respectively. Since then, OC−CH_2OH undergoes exclusively C–H cleaving reaction, OC−CH_2OH→ OC−CHOH (4-26), sharing the same product from C–H scission from the product of 3-13, OCH−CHOH, then followed by next O–H (5-06) or C–H scissions (5-05) to produce OC−CHO and OC−COH, in both structures C–C bond scissions are favoured with lower activation and larger exothermic energies (6-08 and 6-01). Moreover, in OC−CHO, C–C breaking are competitive to C–H scission (6-09), 0.77 (-0.74) eV vs. 0.74 (-0.21) eV. We also observed the C–OH bond scission reaction (5-07) from OC−CHOH with higher barrier of 0.74 eV but larger exothermic energy, -0.84 eV, comparable to C–H breaking (5-06) with energy of 0.53 (-0.46) eV. The product, CH−CO, is an important intermediate for C–C breaking in ethanol decomposition pathway.

Ethylene glycol decomposition on Pd(111) Unlike Ru(0001), C–H breaking on Pd normally are more kinetically and thermodynamically favoured. For ethylene glycol decomposition, C–H (1-02) and O–H scissions (1-03) are kinetically competitive (0.56 vs 0.68 eV), but C–H breaking is thermodynamically more favorable (-0.41 eV) than O–H scission (-0.01 eV). As shown in Supplementary Figure 12, di-OH terminated structures have lower potential energies than O terminated ones species in the energy profiles. The decomposition path of ethylene glycol on Pd(111) is complex because of the coexistence of many plausible

intermediates from combination of O–H and C–H breaking reactions.

From the product of 1-02, HOCH$-$CH$_2$OH, C–H breaking from CH$_2$OH (2-03) and O–H scission from HOCH (2-04) proceed with energies of 0.41 (-0.61) and 0.56 (-0.25) eV. From the product of HOCH$-$CHOH, O–H scission (3-09) would compete with C–H breakings (3-08) with close barriers (0.49 vs 0.56 eV) and slightly higher reaction energies (-0.27 vs -0.53). After C–H breaks, O–H breaking from both sides HOC (4-03) and CHOH (4-04) have lower barriers than C–H from CHOH side (4-02) in HOC$-$CHOH, and the reaction energies are still less exothermic than C–H breaking. Step 4-03 with product of OC$-$CHOH is followed by C–C bond breaking (5-04). In the meanwhile, C–H (5-05) and O–H breaking (5-06) from CHOH side from OC$-$CHOH again compete with each other. From the products above, OC$-$CHO and OC$-$COH, C–C breaks more easily than other reactions (6-01 and 6-08). Step 4-04 with product of OCH$-$COH is followed by C–H (5-10) and O–H (5-11) breaking reactions, which generate the same products as 5-05 and 5-06. After O–H breaks from HOCH$-$CHOH in step 3-09, C–H breaking from OCH$-$CHOH to product OC$-$CHOH (4-26) beats other rest reactions, and share the same products from reaction 4-03.

For the product of 1-03, OCH$_2$$-CH_2$OH, two C–H breakings from both sides occur at the same time (2-09 and 2-10). From step 2-09's product, OCH$_2$$-$CHOH, most favoured step is C–H breaking (3-18) which produces OCH$-$CHOH, having the same product from 3-09 step. From step 2-10's product, OCH$-$CH$_2$OH, one C–H breaking on the CH$_2$OH side in 3-13 has the same product with steps of 3-18 and 3-09. Another C–H breaking from 3-12 produces OC$-$CH$_2$OH, then C–H breaking from right side produces OC$-$CHOH (4-08), connects to the previous steps 4-03 and 4-26. O–H breaking (4-09) would also compete the above reaction to produce OC$-$CH$_2$O. Then C–C breaks (5-22) and C–H scission occurs and produces OC$-$CHO, followed by C–C breaking.

From above analysis, it is obvious that O–H and C–H alternatively break and share the specific products, therefore, the reaction network can be connected via these species such as OC$-$CHOH, OC$-$CHO, OC$-$COH from which C–C breaking would compete other reactions.

Ethylene glycol decomposition on Pt(111) Similar to the little differences of ethanol decompositions on Pd(111) and Pt(111), ethylene glycol decomposition on Pt(111) also exists the similarity to that on Pd(111), as shown in Supplementary Figure 13. It may start by either O–H or C–H dehydrogenations to form OCH$_2$$-CH_2$OH and HOCH$-CH_2$OH respectively. From OCH$_2$$-CH_2$OH, H abstracts from OCH$_2$ side (2-10) is almost barrier-less

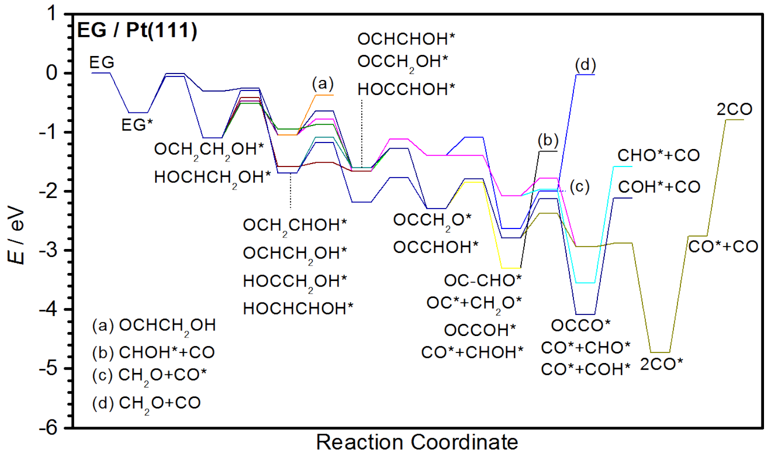

Supplementary Figure 13: **Energy profile for ethylene glycol decomposition on Pt.**

with energy of 0.05 (-070) eV. Then followed the same reaction steps on Pd(111). from HOCH$-$CH$_2$OH, O–H breaking from step 2-04 has the same product with step 2-10. Another O–H breaking produces OCH$_2$$-$CHOH, and then the reactions are very similar to those on Pd(111). Compared with Pd(111), reactions on Pt(111) proceed with more barrier-less reactions. Again, C–C breaks at high dehydrogenated intermediates such as OC$-$CH$_2$O, OC$-$CHOH, OC$-$CHO and OC$-$COH.

Supplementary Methods

Reaction network The full decomposition network of water, methane, methanol, and ethane are shown in Supplementary Figure 14. Additional reactions coming from the O_2 and CO_2 decompositions and the water-gas shift reaction are shown as Supplementary Equations 15–21.[20,21] The decomposition networks of ethanol and ethylene glycol are developed in Supplementary Figure 15-16. We also included reactions assisted by coadsorbed O* and OH* to strip the hydrogen from the hydroxyl group of methanol, ethanol, and ethylene glycol, Supplementary Equation 22-27. The C–H breakings assisted by O* and OH* were not considered in the microkinetic analysis as they have large activation barriers, as exemplified by Supplementary Equation 28-29, Supplementary Table 7.

$$H_2O^* + O^* \rightleftharpoons 2\,OH^* \tag{15}$$

$$O_2{}^* \rightleftharpoons 2\,O^* \tag{16}$$

$$CO_2{}^* \rightleftharpoons CO^* + O\cdot \tag{17}$$

$$COOH^* \rightleftharpoons CO^* + OH^* \tag{18}$$

$$COOH^* \rightleftharpoons CO_2{}^* + H^* \tag{19}$$

$$COOH^* + O^* \rightleftharpoons CO_2{}^* + OH^* \tag{20}$$

$$COOH^* + OH^* \rightleftharpoons CO_2{}^* + H_2O^* \tag{21}$$

$$CH_3OH^* + OH^* \rightleftharpoons CH_3O^* + H_2O^* \tag{22}$$

$$CH_3OH^* + O^* \rightleftharpoons CH_3O^* + OH^* \tag{23}$$

$$CH_3CH_2OH^* + OH^* \rightleftharpoons CH_3CH_2O^* + H_2O^* \tag{24}$$

$$CH_3CH_2OH^* + O^* \rightleftharpoons CH_3CH_2O^* + OH^* \tag{25}$$

$$CH_2OHCH_2OH^* + OH^* \rightleftharpoons CH_2OHCH_2O^* + H_2O^* \tag{26}$$

$$CH_2OHCH_2OH^* + O^* \rightleftharpoons CH_2OHCH_2O^* + OH^* \tag{27}$$

$$CH_3CH_2OH^* + O^* \rightleftharpoons HOCHCH_3{}^* + OH^* \tag{28}$$

$$CH_3CH_2OH^* + O^* \rightleftharpoons CH_2CH_2OH^* + OH^* \tag{29}$$

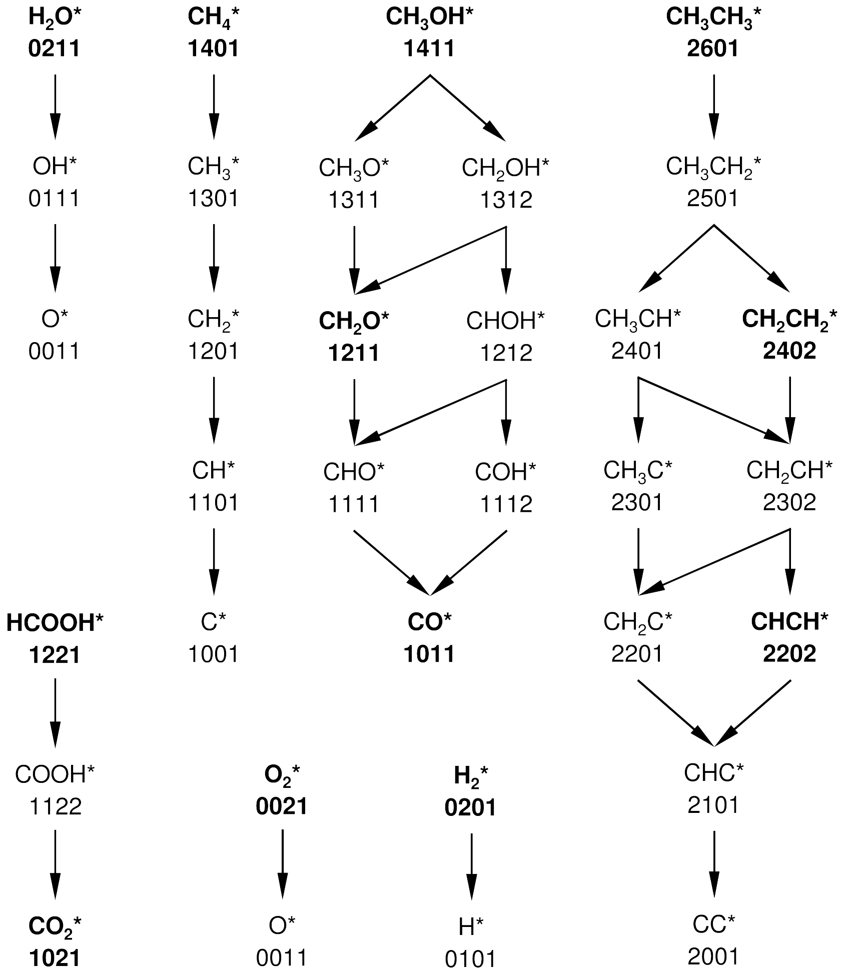

Supplementary Figure 14: **Reaction network for the dehydrogenation of small fragments**. Includes water, methane, methanol, ethane, and formic acid as starting points. Additional reactions come from C–O breaking for methanol and its by-products, and C–C bond breaking for ethane and its derivatives. Results for the reaction and activation energies for water, methane, and methanol can be found in our previous study,[7] including 16 dehydrogenations and 8 C–O breakings. Ethane decomposition comprises 12 dehydrogenations and 10 C–C breakings. Species in bold are stable in gas-phase and may adsorb/desorb. Decomposition of H_2* and O_2* were included for completeness.

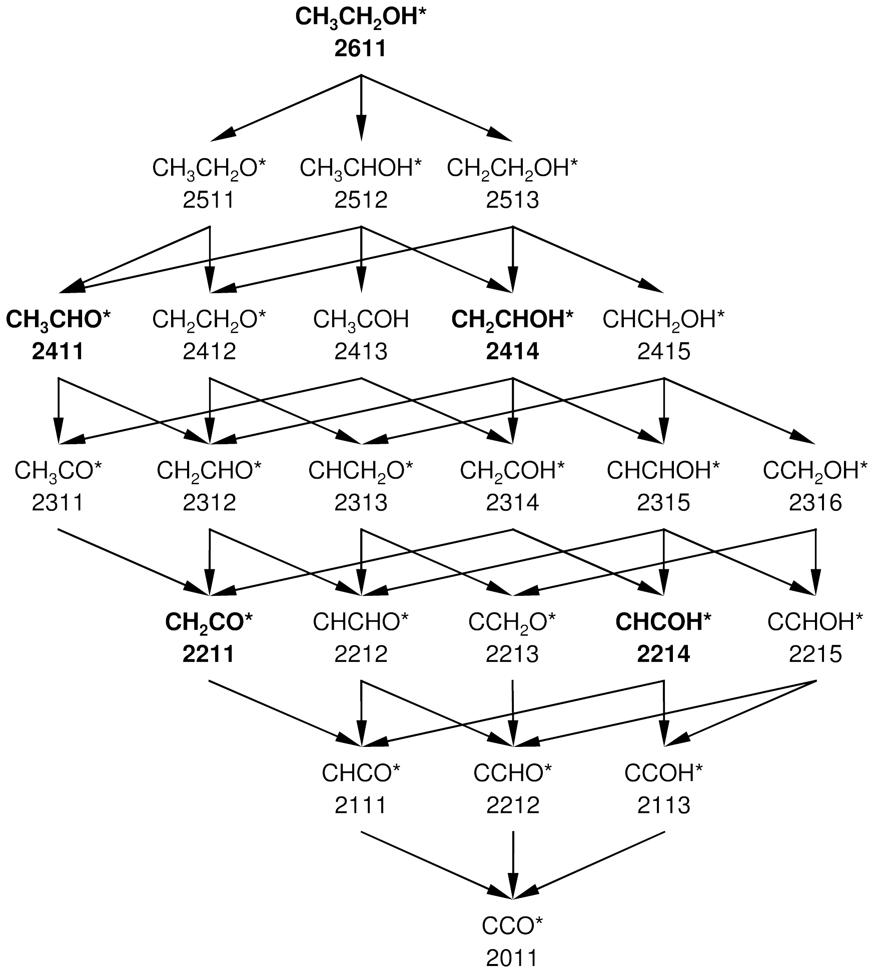

Supplementary Figure 15: **Reaction network for ethanol dehydrogenation**. Additional reactions come from C–O and C–C bond breaking on ethanol and its by-products. The full network comprises 46 dehydrogenations, 24 C–C and 24 C–O bond breakings. Species in bold are stable in gas-phase and may adsorb/desorb.

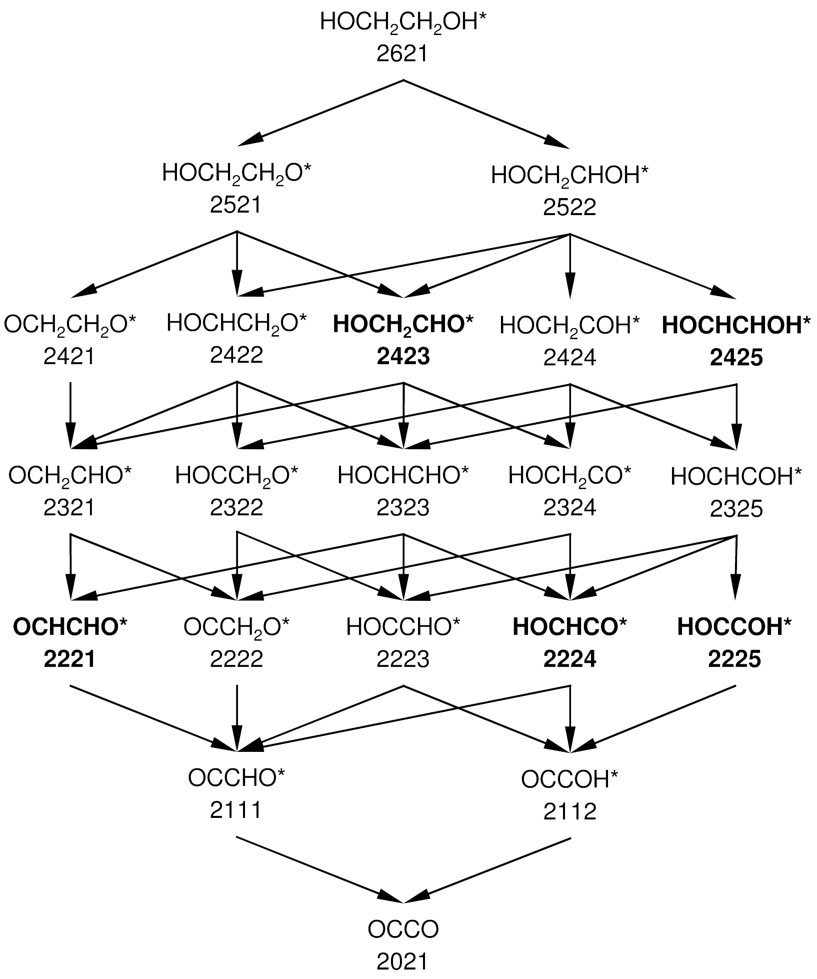

Supplementary Figure 16: **Reaction network for ethylene glycol dehydrogenation**. Additional reactions come from C–O and C–C bond breaking on ethylene glycol and its by-products. The full network comprises 42 dehydrogenations, 21 C–C and 36 C–O bond breakings. Species in bold are stable in gas-phase and may adsorb/desorb.

Energy references The energies reported along the manuscript for any given species $C_xH_yO_z{}^*$ adsorbed on the metal surface, $E_{C_xH_yO_z^*}$, were computed from Supplementary Equation 30-31, taking as reference: the energy of the clean metal surfaces, E_*, the energy of a hydrogen atom adsorbed on the metal surface, E_{H^*}, and a fully hydrogenated molecule in gas phase, $E_{C_2H_6O_z}$. The reference molecules are ethane, ethanol, and ethylene glycol, corresponding to the number of oxygens with z=0, 1, 2, respectively. The variables on the right side of Supplementary Equation 31, with the tag VASP, are used as obtained from the VASP package, adding the zero-point vibrational energies obtained from the frequency calculations. The zero-point energies for all C_2 species along the manuscript are shown in Supplementary Table 6. The values for C_1 can be found in our previous work.[7]

$$C_2H_6O_z(g) + (7-y)^* \longrightarrow C_2H_yO_z{}^* + (6-y)H^* \tag{30}$$

$$E_{\mathrm{C_xH_yO_z}*} = E^{\mathrm{VASP}}_{\mathrm{C_xH_yO_z}*} + (6-y) * E^{\mathrm{VASP}}_{H*} - (7-y) * E^{\mathrm{VASP}}_{*} - E^{\mathrm{VASP}}_{\mathrm{C_2H_6O_z}} \tag{31}$$

The adsorption energy for species "A", $E_{\mathrm{ads,A}}$ is calculated according to Supplementary Equation 32-33. The full list of stable gas-phase molecules and their adsorption energies is shown in Supplementary Table 4.

$$\mathrm{A(g)} + {}^{*} \rightleftharpoons \mathrm{A}^{*} \tag{32}$$

$$E_{\mathrm{ads,A}} = E_{\mathrm{A}*} - E_{\mathrm{A(g)}} - E_{*} \tag{33}$$

For any generic decomposition reaction, Supplementary Equation 34, the reaction and activation energies, ΔE and E_{a}, were calculated using Supplementary Equation 35-36. E_{IS}, E_{TS}, and E_{FS} are the energies of the initial, transition and final states respectively. All reactions with their corresponding ΔE and E_{a} are presented in Supplementary Table 7.

$$\underbrace{\mathrm{AB}^{*} + {}^{*}}_{\mathrm{IS}} \rightleftharpoons \underbrace{[\mathrm{A}^{*} \cdots \mathrm{B}^{*}]^{\dagger}}_{\mathrm{TS}} \rightleftharpoons \underbrace{\mathrm{A}^{*} + \mathrm{B}^{*}}_{\mathrm{FS}} \tag{34}$$

$$\Delta E = E_{\mathrm{FS}} - E_{\mathrm{IS}} = E_{\mathrm{A}^{*}} + E_{\mathrm{B}^{*}} - E_{\mathrm{AB}^{*}} - E_{*} \tag{35}$$

$$E_{\mathrm{a}} = \max\left(E_{\mathrm{TS}} - E_{\mathrm{IS}}; \Delta E; 0\right) \tag{36}$$

Equations of linear scaling relationships

The detailed calculations on all O–H, C–H, C–C, C–O, and C–OH cleavages in the decomposition network supplied us with sufficient data to analyze the Brønsted-Evans-Polanyi (BEP) relationships between the energy barriers and reaction energies, Supplementary Equation 37. We also tested transition state scaling correlations, which correlates the energies of the transition states whith the ones of the initial (ISS) or final (FSS) states, Supplementary Equation 38-39. Both methods are useful for the fast prediction of energy barriers from the energies of the initial and final states, which are easier to be obtained computationally.

$$\tilde{E}_{\mathrm{a}} = \alpha \Delta E + \beta \tag{37}$$

$$\tilde{E}_{TS} = \alpha E_{IS} + \beta \tag{38}$$

$$\tilde{E}_{TS} = \alpha E_{FS} + \beta \tag{39}$$

The BEP, ISS, and final state scaling relationship are shown in Supplementary Table 8. Compared with the good scaling observed on ISS and FSS methods, the traditional BEP relationships show larger deviations that may render the method unsuitable for reliable predictions of energy barriers. An important source of deviation is the large structural variation of some intermediates while the reaction is taking place. In the following sections, ISS and FSS correlations of O–H, C–H, C–C, and C–O cleavage reactions are discussed. We also detail about the factors that lead to deviation from the scaling lines.

Microkinetic model details

For simplicity, our microkinetic model describes the behavior of a differential reactor, as shown in Supplementary Figure 17. This is a particular case of a packed-bed reactor where the catalyst has differential thickness.

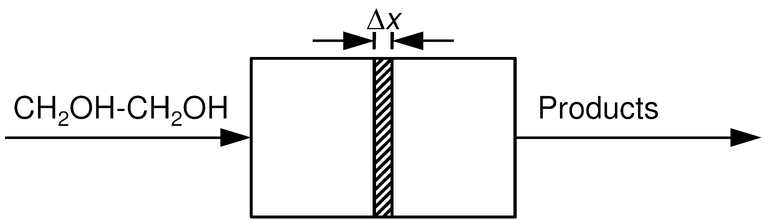

Supplementary Figure 17: **Differential reactor used in the microkinetic model.**

The total adsorption/desorption rate for a species j (Supplementary Equation 32) was modeled using the Knudsen equation:

$$r_{ads_j} = \frac{P_j \; S_0(T)}{N_0\sqrt{2\pi m_j k_\mathrm{B} T}}\theta_* - \frac{k_\mathrm{B}T}{h}\mathrm{exp}\left(\frac{E_{ads_j}}{k_\mathrm{B}T}\right)\theta_j \tag{40}$$

Where:

r_{ads_A}	Rate of adsorption (+) or desorption (–) of j
P_j	Pressure of j in gas phase
$S_0(T)$	Sticking coefficient
N_0	Density of sites per area
m_j	Molecular mass of j
E_{ads_j}	Adsorption energy of j, from 33

The rate for the i-th reaction (Supplementary Equation 34) is given by:

$$r_i = \frac{k_\mathrm{B}T}{h}\; exp\left(-\frac{E_\mathrm{a}}{k_\mathrm{B}T}\right)\theta_{\mathrm{AB}^*}\theta_* - \frac{k_\mathrm{B}T}{h}\; exp\left(-\frac{E'_\mathrm{a}}{k_\mathrm{B}T}\right)\theta_{\mathrm{A}^*}\theta_{\mathrm{B}^*} \tag{41}$$

Where:

r_i	Rate of i-th reaction
E_a, E'_a	Activation energy for direct and inverse i-th semireactions
θ_j	Surface concentration of species "j"
k_B	Boltzmann constant
T	Temperature

The variation of the surface concentration of species "j" was modeled by a System of "n" Ordinary Differential Equations (SODE), being "n" the total number of species. These ODE consist in the algebraic sum of the adsorption and reaction rates in which a given surface species "j" participates, as shown in Supplementary Equation 42.

$$\frac{d\theta_j}{dt} = \sum_{i=1}^{n} r_{ads_j} + s_{ij} r_i \tag{42}$$

Where:

s_{ij}	Stoichiometric coefficient of species "j" on reaction "i".
t	Time

For each time "t", the number of empty sites was calculated with the site balance equation:

$$\theta_* = 1 - \sum_{j=1}^{n} \theta_j \tag{43}$$

The general procedure can be summarized as follows: First, define the working conditions, adjusting the temperature and reactant pressures according to Supplementary Table 1. Then, calculate the adsorption and reaction constants from Supplementary Equations 40 and 41. For simplicity, the initial concentrations may correspond to a clean surface: $\theta_*(t = 0) = 1$. The concentrations and reaction rates for a given time t can then be obtained by using the Rosenbrock method, which is suitable for solving systems of differential equations which expand over several time scales (i.e. stiffness). While the concentration of all intermediates j is in a differential form, Supplementary Equation 42, the fraction of empty sites can be obtained from the site balance, which is algebraic, Supplementary Equation 43.

For simplicity, several assumptions were imposed in the derivation of the microkinetic model. Firstly, the reactor is isothermic. Also, in a differential reactor, the residence time in the reactor is negligible compared to the reaction time scales. As a consequence, the conversion of the reactants is negligible and the pressures of reactants are constant. This means that the partial pressures for all products in the outlet stream are negligible and can be set to zero. The fluid phase was assumed homogeneous, with no diffusion limitations. For simplicity, we also assumed that each intermediate occupies only one reaction site. Thus, all

reaction rates r_{ij} were written per unit of active sites on the surface. The sticking coefficients for adsorption were set to 1, as all adsorptions were found exothermic and unactivated. Our model neglects adsorbate-adsorbate interactions and the contributions of the partition functions in the prefactor coefficients.

Several strategies were put forward to improve the reliability, stabilily, and efficiency of the microkinetic model. The system of ordinary differential equations (SODEs) was solved numerically in Maple 13 using the Rosenbrock method. The floating-point precission was set to 64 digits. Increasing it to 100 digits gave no significant difference in the final results. The absolute error tolerance was adjusted to a tight value ($abserr = 1E - 16$). Besides, A damping function was added to the pressure of reactants, Supplementary Equation 44, to ensure that the pressure for each reactant "j" increases gently in the early stages, thus avoiding numerical instabilities. We set $\tau_{\mathrm{P}} = 1$ s. The reactant pressures stabilize to the set-point values P_{j0} in less than $10\tau_P$, as shown in Supplementary Figure 18.

$$P_j = \left(1 - exp\left(-\frac{t}{\tau_{\mathrm{P}}}\right)\right)^2 P_{j0} \tag{44}$$

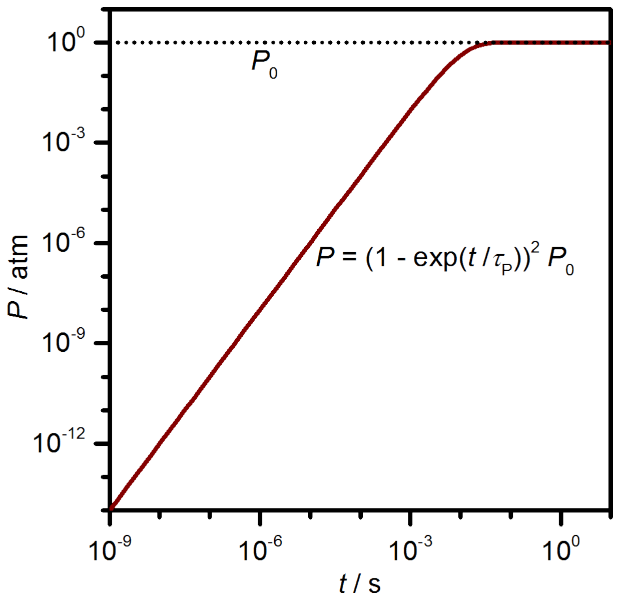

Supplementary Figure 18: **Pressure damping**. Total reactant pressure when a damping function is applied, according to Supplementary Equation 44.

Getting data from ioChem-BD

To check the structure and energy on any intermediate or transition state in ioChem-BD,[8] first the user has to ented into the desired collection (Ref. [13] in the present article). A tutorial is available in the first visit. The structure, energy, and other related metadata

is available upon a clic on the title of each calculation. The label format depend on the species. For gas-phase molecules, the tags have the format "`gas-XXXX`", where `XXXX` is the code shown in Supplementary Figure 14-16 (for species in bold only). For adsorbed C_0-C_2 species, the tags have the format `MM-XXXX`, where `Mm` is the metal {Cu, Ru, Pd, Pt} and `XXXX` is the code shown in Supplementary Figure 14-16. For adsorbed C_3 species on Ru, the labels have the format `Ru-XXXXX`, where `XXXXX` is the code shown in Supplementary Table 17. For transition states, tags have the format `MM-X-XX`, where `Mm` is the metal and `X-XX` is the reaction label from Supplementary Table 7. A `.csv` file, containing the full set of energies with their tags, is available for download upon a clic on "`Export .csv`" on the top right corner. The `.csv` file should be opened using the `UTF-8` encoding. In LibreOffice Calc, `UTF-8` is the default encoding. Commas should be used as delimiters when importing the data. No further actions are requested. In Microsoft Excel, the procedure is more elaborate: First a new spreadsheet should be opened. Then, in the "Data" menu, "From Text" button, the `.csv` file con be selected. On "File Origin", the encoding "65001 Unicode UTF-8" should be chosen, and commas should be used as delimiters.

Supplementary Tables

Supplementary Table 1: **Reaction conditions for the microkinetic model**: temperature (T, in K), partial pressures of alcohol, water, and oxygen in the inlet (P_a, P_w, P_o, in atm), and total reaction time (t_x, in s).

Process	Alcohol	T	P_a	P_w	P_o	t_x
Direct decomposition	All	600	1.000	0.000	0.000	10800
Autothermal reforming	Ethanol	950	0.250	0.625	0.125	10800
	Ethylene glycol	950	0.300	0.500	0.050	10800
	Glycerol	950	0.250	0.680	0.070	10800
Steam reforming	Ethanol	800	0.200	0.800	0.000	10800
	Ethylene glycol	800	0.250	0.750	0.000	10800
	Glycerol	800	0.100	0.900	0.000	10800
Aqueous phase reforming	Ethanol	550	20.00	80.00	0.000	10800
	Ethylene glycol	550	25.00	75.00	0.000	10800
	Glycerol	550	10.00	90.00	0.000	10800

Supplementary Table 2: **Previous experimental studies**. This table shows typical reaction conditions for decomposition, ATR, SR, and APR of alcohols on transition metals found in previous experimental studies: temperature (T, in K), pressure (P, in atm). The composition as molar ratios is shown for ATR (alcohol:H_2O:O_2) and SR (alcohol:H_2O).

Ref.	Alcohol	Metal	T	P	Ratios
		Direct decomposition			
22	MeOH	Ru	85–400	10^{-13}	–
23	MeOH	Pd	140–300	10^{-13}	–
24	MeOH	Pd	225–800	10^{-13}	–
25	MeOH	Au	77–650	10^{-13}	–
26	MeOH	Pt	90–350	10^{-13}	–
27	MeOH	Rh	100–600	10^{-13}	–
28	MeOH	Pd	100–550	10^{-12}	–
29	MeOH	Pd	90–500	10^{-12}	–
30	MeOH	Ru	80–600	10^{-13}	–
31	MeOH	Pt	100–600	10^{-12}	–
32	C_1–C_4	Pt	100–600	10^{-12}	–
33	EtOH	Pd	150–450	10^{-13}	–
34	EtOH	Pt	150–850	10^{-13}	–
35	EtOH, ethylene glycol	Pd	100–800	10^{-12}	–
36	EtOH	Pd	200–600	10^{-12}	–
37	C_1–C_3	Pd	160–700	10^{-12}	–
		Autothermal reforming			
38	MeOH	Cu, Pd	573–873	1.0	2:0:1
39	MeOH	Cu, Pd	273–900	1.0	10:0:5–10:15:2
40	MeOH	Cu, CuO	283–603	1.0	8:6:1
41	MeOH	Cu	470–590	1.5	1:0:0–1:0.75:0.8
42	MeOH, EtOH, Glycerol	Pt, Rh	673–1273	3.0	1:0:0–1:9:1.5
43	EtOH	Rh	873–1096	1.0	3:0:1–3:12:2
44	EtOH	Rh, Pt, RhPt	673–1173	1.0	5:20:1
45	EtOH	Cu,Ag,Au,Co,Ni Pd,Pt,Ru,Rh,Ir	673–873	1.0	1:0:0.03–1:10:0.3
46	bio-BuOH	{Ru,Rh,Ir,Pd}Co	773	1.0	1:10:1.6
47	BuOH	Rh	773–973	1.0	1:16:0.4

Supplementary Table 2 – continues from previous page

Ref.	Alcohol	Metal	T	P	Ratios
		Steam reforming			
48	MeOH	Pd	493	1.0	1:1
49	MeOH	Cu	523	1.0	1:2
50	MeOH	Cu	453–573	1.0	10:13
51	EtOH	Rh	573–923	1.0	10:84
52	EtOH	Co	573–723	1.0	1:13
53	EtOH	Ru	873–973	0.5	1:10
54	EtOH	CuNi	673–923	1.0	1:3.7
55	EtOH	Pd, Pt	575–723	0.5	1:3
56	EtOH	Pt	573	0.02	1:3
57	EtOH	Co	623–773	1.0	1:10
58	EtOH	Rh	773–1073	1.0	1:3–1:10
59	EtOH	Ru, Pd, Ag	373–873	1.0	1:5
60	EtOH	Ni	773–1023	1.0	1:3
61	EtOH	RhPt	723–923	1.0	1:3.4–1:8.0
62	EtOH	Pt	573–675	1.0	1:3:4
63	Glycerol	Ru, Fe, Ir, Rh Co, Pt, Pd, Ni	873	1.0	1:9.9
64	Glycerol	Ni,Pd,Pt,Rh,Ir,Ru	873–1173	1.00	1:3-1:9
65	BuOH	Rh	773–973	1.0	1:16
		Aqueous phase reforming			
66	MeOH	Pt/α-MoC	423–463	< 60.0	–
67	MeOH, ethylene glycol	Pt	523	22.1	–
68	EtOH	Ni	473–523	15.0–39.0	–
4	EG	Ni, Pd, Pt, Ru, Rh	483–498	21.7	–
69	EG	120 bimetallic catalysts	483	4.8–25.5	–
70	EG	Pd	498	25.5	–
71	EG	Pt	548–723	200–250	–
72	EG	Pt{Re,Mn,Fe,Cs,Ba, Ba,Ga,Ag,Mo}	523	45.0	–
73	EG, Glycerol, Sorbitol	$Ni_{37}Sn_3$	498–538	25.8–51.4	–
74	EG, Glycerol, Sorbitol	Pt, Pd	498–538	25.8–59.9	–
75	EG, Glycerol, Sorbitol	Pt, Ni, SnNi	498–538	25.8–55.3	–
76	Glycerol	Pt, PtRe	498	29.9	–
77	Sorbitol, Galactitol	Pt	495	29.3	–

Supplementary Table 3: **Previous theoretical studies**. This table lists previous theoreticas studies on the decomposition of C_1-C_3 alcohols on transition metal surfaces. The number of reactions (NR), the use of zero-point energies (ZPE), the presence of a microkinetic model (MK), and the obtention of (Extended) Brønsted-Evans-Polanyi relationships (BEP) is indicated. The most stable terminations for fcc and hcp metals, (111) and (0001), are ommited for simplicity.

Ref.	Metal	Alcohol	NR	Method	ZPE	MK	BEP	Year
78	Cu	MeOH	6	B3LYP				2001
79	Cu	MeOH	4	PW91	✓			2002
80	Cu	MeOH	38	PW91	✓	✓		2011
81	Cu	MeOH	-	optB88-vdW		✓		2012
82	Cu	MeOH	34	PW91		✓		2014
83	Cu	MeOH	14	PW91	✓	✓		2014
84	Cu(100)	MeOH	10	PW91				2017
85	Cu(110)	MeOH	11	PBE			✓	2016
86	Cu(110)	MeOH	13	PBE		✓		2016
87	Cu(110)	MeOH	-	PBE				2008
88	Cu(100), Cu(110)	MeOH	4	PW91				2005
89	Cu(110)	MeOH	4	PW91		✓		2006
90	Cu(110)	MeOH	9	PBE		✓		2007

Supplementary Table 3 – Continues from previous page

Ref.	Metal	Alcohol	NR	Method	ZPE	MK	BEP	Year
91	Cu(221), PdZn(100), Ir(100)	MeOH	9	PBE				2012
92	Cu, Cu_2O/Cu	MeOH	6	PW91				2016
93	Cu, Pd	MeOH	13	PW91				2010
94	Cu, Au, Pt	MeOH	-	optB88-vdW				2014
95	Cu, Co, Ni	MeOH	24	PBE			✓	2016
96	Cu, Pd, PdZn	MeOH	3	PW91				2004
97	Ni, Pd, Pt, Rh, Au doped Cu	MeOH	15	PW91		✓		2012
98	ZnCu	MeOH	52	PW91		✓		2016
99	Pd	MeOH	3	PW91				2003
100	Pd	MeOH	13	BP				2008
101	Pd	MeOH	10	PW91	✓			2009
102)	Pd	MeOH	4	PW91	✓		✓	2010
103	Pd	MeOH	4	PW91	✓			2011
104	Pd	MeOH	19	PW91	✓	✓		2013
105	Pd(100)	MeOH	10	PW91				2016
106	Pd(211)	MeOH	12	PW91	✓	✓		2013
107	PdIn(110)	MeOH	4	PBE, vdw-DF	✓			2012
108	PdZn	MeOH	5	PW91	✓			2011
109	PdZn	MeOH	11	PW91	✓			2012
110	PdZn	MeOH	7	PW91	✓			2011
111	PdZn	MeOH	7	PW91	✓			2013
112	PdZn	MeOH	8	PW91				2015
113	PdZn	MeOH	4	PW91	✓		✓	2016
114	Pt	MeOH	4	PW91				2002
115	Pt	MeOH	10	PW91			✓	2004
116	Pt	MeSH	12	PW91		✓	✓	2010
117	PtRu	MeOH	16	PW91	✓			2015
118	PtAu	MeOH	4	PW91				2008
119	PtAu	MeOH	7	PBE				2012
120	PtRu/Pt	MeOH	12	PW91	✓			2016
121	Pt_3Sn	MeOH	11	PW91	✓	✓	✓	2016
122	Pt_3Ni	MeOH	-	PBE-D3				2015
123	$PtPd_3$	MeOH	10	PBE				2015
124	Pt, Pd, Ni	MeOH	10	PW91		✓		2014
125	NiZn, PdZn, PtZn	MeOH	2	PBE	✓			2015
7	Cu, Ru, Pt, Pd	MeOH	18	PBE-D2	✓		✓	2015
126	Au, Ag, Cu, Pt, Pd, Ni, Ir, Rh	MeOH	26	PW91	✓			2008
127	Co,Ni,Ru,Rh,Pd,Pt-doped Cu	EtOH	4	PBE	✓	✓	✓	2017
128	Ni(100)	MeOH	13	PBE			✓	2006
129	Ni	MeOH	13	PBE			✓	2005
130	Ni	MeOH	10	RPBE			✓	2004
131	Au	MeOH	5	PW91				2006
132	Au	MeOH	33	PW91				2014
133	Ru	MeOH	14	PW91	✓	✓		2015
134	Ru	MeOH	20	PW91	✓			2016
135	V(100)	MeOH	12	PBE	✓	✓		2012
136	Ir	MeOH	13	PW91	✓			2013
137	Co,	MeOH	18	PBE	✓	✓		2014
138	ZnO(001)	MeOH	3	PW91	✓			2013
139	Co	EtOH	29	PW91	✓			2015
140	Co	EtOH	37	PW91				2016
14	Cu	EtOH	5	RPBE				2012
141	Cu(100)	EtOH	13	PW91	✓			2015
142	Cu(111),(100),(110)	EtOH	2	PBE	✓			2012
143	Pt	EtOH	24	PW91		✓	✓	2003
144	Pt(111),(211),(100)	EtOH	23	PBE	✓			2008
62	Pt	EtOH	18	PBE		✓	✓	2013
145	Pt	EtOH	160	PBE		✓	✓	2016
146	Pt	EtOH	-	PBE-D3				2014
33	Pd	EtOH	2	RPBE-D2				2014
147	Pd	EtOH	33	PW91				2010
148	Pd	EtOH	10	PBE				2014
149	Pd(110)	EtOH	22	PW91				2010
150	Rh	EtOH	35	PW91		✓		2010
151	Rh	EtOH	24	PW91		✓		2013
152	Rh(211)	EtOH	35	PW91				2011

Supplementary Table 3 – Continues from previous page

Ref.	Metal	Alcohol	NR	Method	ZPE	MK	BEP	Year
153	Cu(211)	EtOH	22	PW91				2013
154	Rh(211)	EtOH	20	PW91				2014
155	Rh	EtOH	-	PW91				2007
156	MnCu(211)	EtOH	24	PW91	✓			2014
157	Pt	EtOH	-	PBE-D3			✓	2013
158	$Mo_2C(100)$	EtOH	44	PW91	✓		✓	2013
16	Ru	EtOH	47	PW91				2013
159	Rh	EtOH	15	PW91	✓	✓		2009
160	Rh	EtOH	40	PW91		✓		2011
15	Rh	EtOH	10	PW91				2011
161	Rh(111),(211)	EtOH	17	PW91	✓	✓		2010
162	Rh(111),(553)	EtOH	1	PW91				2008
163	Rh(211)	EtOH	33	PW91		✓		2014
164	α-Al_2O_3	EtOH	12	PW91				2016
165	Co	EtOH	10	PBE		✓	✓	2012
166	Co, Ni, Pd, Pt, Rh, Ru	EtOH	18	PBE	✓	✓	✓	2015
167	Co, Ni, Pd, Pt, Rh, Ru	EtOH	18	PBE		✓	✓	2016
168	Co, Ni, Cu, Rh, Pd, Ag, Ir, Pt, Au	EtOH	12	PW91				2009
12	Cu, Pt, Pd, Ni, Ir,Rh, Co, Os, Ru, Re	EtOH	24	PW91	✓		✓	2009
18	Pt, Ni-Pt	EG	63	PBE	✓			2011
19	{Ni,Au,Cu,Pt}-Mo_2C	EG	15	PW91, PBE		✓		2014
169	{Rh,Ni,Co}-$MgAl_2O_4$	EG	63	PBE	✓			2016
170	Pt	EG	250	PBE	✓	✓	✓	2011
171	Pt	EG	63	PW91		✓	✓	2011
172	Pt(111),(211)	EG	63	PW91			✓	2015
2	Rh	Glycerol	29	PW91			✓	2014
15	Rh	Glycerol	6	PW91				2011
173	Rh	Glycerol	10	PW91				2011
10	Pd, Rh, Cu, Ni	Glycerol	36	PW91			✓	2013
174	Pt(111),(110),(100)	Glycerol	-	PBE-D3				2014
175	Pt	Glycerol	21	PW91			✓	2011
176	Pt	Glycerol	7	PW91			✓	2012
177	Pt	Glycerol	204	PBE			✓	2011

Supplementary Table 4: **Adsorption energies in gas phase**. Adsorption energies, in eV, for molecules that are stable in gas-phase on Cu, Pd, Pt(111), and Ru(0001). For the dissociative adsorption of H_2, O_2, and CH_4, the corresponding reactions are included.

Formula	Name	Cu	Ru	Pd	Pt
$\frac{1}{2}H_2 + * \longrightarrow H*$	Hydrogen	-0.30	-0.66	-0.75	-0.59
$\frac{1}{2}O_2 + * \longrightarrow O*$	Oxygen	-1.73	-2.93	-1.63	-1.27
$CH_4 + * \longrightarrow C* + 2\,H_2$	Carbon	3.72	1.06	1.50	1.43
O_2	Oxygen	-1.02	-2.11	-1.13	-0.73
CH_4	Methane	-0.09	-0.09	-0.14	-0.12
CH_3OH	Methanol	-0.35	-0.55	-0.46	-0.44
CH_2O	Formaldehyde	-0.30	-1.20	-0.75	-0.63
CO	Carbon monoxide	-1.00	-1.99	-2.30	-1.97
CO_2	Carbon dioxide	-0.10	-0.43	-0.02	0.14
H_2O	Water	-0.26	-0.47	-0.33	-0.31
$HOCH_2{-}CH_2OH$	Ethylene glycol	-0.56	-0.79	-0.70	-0.68
$CH_3{-}CH_2OH$	Ethanol	-0.43	-0.63	-0.52	-0.51
$CH_3{-}CH_3$	Ethane	-0.18	-0.18	-0.20	-0.17
$CH_3{-}CHO$	Acetaldehyde	-0.22	-1.05	-0.67	-0.50
$CH_2{-}CH_2$	Ethene	-0.45	-1.35	-1.23	-1.39
CH−CH	Ethyne	-1.51	-3.13	-2.41	-2.58
$CH_2{-}CHOH$	Ethenol	-0.54	-1.42	-1.30	-1.45
$CH_2{-}CO$	Ketene	-0.37	-1.90	-1.48	-1.74
HC−COH	Ethynol	-1.93	-3.23	-2.98	-3.25
HOC−COH	Acetylenediol	-2.47	-4.04	-3.76	-3.95
$CHOCH_2OH$	Glycolaldehyde	-0.33	-1.34	-0.77	-0.67
HOCH−CHOH	Ethene-1,2-diol	-0.60	-1.38	-1.41	-1.58
OCH−CHO	Glyoxal	-1.27	-2.40	-1.34	-1.04
OC−CHOH	2-hydroxyethenone	-1.25	-2.78	-2.31	-2.49

Supplementary Table 5: **Adsorption energies in liquid phase**. Adsorption energy, in eV, for water-solvated molecules on Cu, Ru, Pd and Pt, calculated following the methodology in Ref. [3, 178] and Supplementary Equation 33.

Formula	Name	Cu	Ru	Pd	Pt
H_2O	Water	0.11	-0.05	0.10	0.22
CO	Carbon monoxide	-0.72	-1.60	-1.94	-1.51
OCH−CHO	Glyoxal	-0.90	-2.03	-0.82	-0.44
$CH_3{-}CHO$	Acetaldehyde	0.07	-0.85	-0.45	-0.16
$CHOCH_2OH$	Glycolaldehyde	0.11	-0.39	-0.38	-0.21
$CH_3{-}CH_2OH$	Ethanol	-0.32	-0.47	-0.21	-0.10
$HOCH_2{-}CH_2OH$	Ethylene glycol	-0.11	-0.26	-0.27	-0.13

Supplementary Table 6: **Referenced energies**. Referenced energies, in eV, for C_2 species on Cu, Ru, Pd, and Pt, including zero-point energy corrections.

Formula	Name	Cu	Ru	Pd	Pt
	C_2H_x, x=0..2				
C−C	Carbon dimer	1.88	-1.93	-1.37	-0.23
C−CH	Ethynyl	1.38	-2.31	-1.49	-0.76
C−CH_2	Vinylidene	1.23	-2.08	-1.95	-1.56
CH−CH	Acetylene	0.88	-2.17	-1.80	-1.35
	C_2H_3				
C−CH_3	Ethylidyne	1.07	-1.88	-2.06	-1.74
CH−CH_2	Vinyl	1.00	-1.56	-1.38	-1.18
	C_2H_4				
CH−CH_3	Ethylidene	1.01	-1.16	-0.93	-0.77
CH_2−CH_2	Ethene	0.51	-1.10	-1.16	-1.01
	C_2H_x, x=5..6				
CH_2−CH_3	Ethyl	0.51	-0.55	-0.52	-0.56
CH_3−CH_3	Ethane	-0.18	-0.18	-0.20	-0.17
	C_2H_xO, x=0..1				
C−CO	Ketenylidene	0.07	-3.29	-3.25	-2.37
C−CHO		0.87	-2.56	-2.60	-1.92
C−COH		1.17	-2.52	-2.02	-1.52
CH−CO	Ketenyl	0.13	-3.04	-2.97	-2.36
	C_2H_2O				
CH_2−CO	Ketene	0.41	-2.56	-2.49	-2.12
CH−CHO	Formylmethylene	0.08	-2.48	-2.06	-1.59
C−CH_2O		1.63	-2.11	-1.35	-0.88
C−CHOH		0.54	-2.50	-2.45	-2.06
CH−COH	Ethynol	0.46	-2.27	-2.37	-2.02
	C_2H_3O				
CH_3−CO	Acetyl	-0.03	-2.22	-2.42	-1.84
CH_2−CHO	Formylmethyl	-0.18	-2.17	-1.80	-1.24
CH−CH_2O		0.83	-1.58	-0.97	-0.73
CH_2−COH	1-Hydroxy-vinyl	0.53	-1.92	-2.12	-1.90
CH−CHOH		0.29	-1.97	-1.73	-1.51
C−CH_2OH		0.97	-1.93	-2.05	-1.52
	C_2H_4O				
CH_3−CHO	Ethanal	-0.03	-1.58	-1.38	-0.90
CH_2−CH_2O		0.27	-1.40	-0.69	-0.56
CH_3−COH	1-Hydroxy-ethylidene	0.49	-1.28	-1.75	-1.53
CH_2−CHOH	Ethenol	0.11	-1.48	-1.54	-1.38
CH−CH_2OH	2-Hydroxy-ethylidene	0.69	-1.35	-1.13	-0.98
	C_2H_5O				
CH_3−CH_2O	Ethoxide	-0.66	-1.39	-0.34	-0.14
CH_2−CH_2OH	2-Hydroxy-ethyl	0.19	-1.03	-0.73	-0.79
CH_3−CHOH	1-Hydroxy-ethyl	0.29	-0.89	-1.04	-1.05
	C_2H_6O				
CH_3−CH_2OH	Ethanol	-0.43	-0.63	-0.52	-0.51
	C_2H_xO2, x=0..2				
OC−CO	Ethylene dione	0.53	-3.50	-3.61	-2.94
OC−CHO		0.33	-3.31	-3.12	-2.08
OC−COH		0.37	-3.24	-3.38	-2.77
OC−CH_2O		-0.21	-2.75	-2.25	-1.75
OC−CHOH		0.10	-2.87	-2.74	-2.30
OCH−CHO	Glyoxal	-0.46	-3.02	-2.31	-1.38
OCH−COH		0.03	-2.75	-2.50	-2.04
HOC−COH	Acetylenediol	0.19	-2.80	-2.88	-2.43

Continues on next page

Supplementary Table 6 – continues from previous page

Formula	Name	Cu	Ru	Pd	Pt
		$C_2H_3O_2$			
$OCH{-}CH_2O$		-0.44	-2.23	-1.26	-0.72
$OCH{-}CHOH$		-0.36	-2.40	-2.15	-1.60
$OC{-}CH_2OH$	2-Hydroxyacetyl	-0.19	-2.47	-2.46	-1.66
$HOC{-}CH_2O$		0.29	-2.25	-1.66	-1.34
$HOC{-}CHOH$		0.26	-2.15	-2.41	-2.19
		$C_2H_4O_2$			
$OCH{-}CH_2OH$	Glycolaldehyde	-0.14	-1.86	-1.47	-1.05
$HOC{-}CH_2OH$	1,2-Dihydroxyvinyl	0.36	-1.69	-1.60	-1.58
$OCH_2{-}CH_2O$	1,2-Dioxyethylene	-0.67	-1.94	-0.44	-0.12
$OCH_2{-}CHOH$		0.12	-1.75	-1.13	-0.95
$HOCH{-}CHOH$	Dihydroxyethene	-0.13	-1.63	-1.83	-1.69
		$C_2H_5O_2$			
$OCH_2{-}CH_2OH$	2-Hydroxyethoxy	-0.74	-1.57	-0.77	-0.31
$HOCH{-}CH_2OH$	1,2-Dihydroxyetghyl	0.02	-1.17	-1.16	-1.10
		$C_2H_6O_2$			
$HOCH_2{-}CH_2OH$	Ethylene glycol	-0.42	-0.79	-0.56	-0.52
$HOCH_2{-}CH_2OH^H$	Ethylene glycol	-0.56	-0.77	-0.70	-0.68

$HOCH_2{-}CH_2OH^H$ has an intramolecular hydrogen bond

Supplementary Table 7: **List of activation and reaction energies**. Activation and reaction energies, E_a and ΔE, for the full decomposition network of methanol, methane, ethane, ethanol, ethylene glycol and WGS reactions on Cu, Ru, Pd, and Pt. All energies are in eV and include zero-point vibrational contributions. For few reactions we were unable to find the transition states, the corresponding E_a are reported as "-".

			E_a				ΔE			
Tag	Reaction	Type	Cu	Ru	Pd	Pt	Cu	Ru	Pd	Pt
	Average-O–H		0.82	0.52	0.59	0.54	-0.17	-0.58	-0.21	0.06
	Average-C–H		0.94	0.37	0.49	0.56	0.21	-0.46	-0.50	-0.39
	Average-C–C		1.78	0.99	1.20	1.20	1.16	-0.31	-0.46	-0.41
	Average-C–O(terminal)		1.65	1.11	1.82	1.95	0.44	-1.07	0.13	0.31
	Average-C–OH		1.16	0.83	1.43	1.50	-0.22	-0.77	0.22	0.50
1-01	$HOCH_2CH_2OH \rightarrow CH_2OH+CH_2OH$	C-C	-	-	-	-	1.59	0.04	0.21	-0.19
1-02	$HOCH_2CH_2OH \rightarrow HOCHCH_2OH+H$	C-H	1.29	0.52	0.56	0.62	0.58	-0.40	-0.41	-0.38
1-03	$HOCH_2CH_2OH \rightarrow OCH_2CH_2OH+H$	O-H	0.95	0.32	0.68	0.67	-0.18	-0.80	-0.01	0.41
1-04	$HOCH_2CH_2OH \rightarrow CH_2CH_2OH+OH$	C-OH	1.63	1.09	1.94	1.80	-0.03	-0.91	0.14	0.23
1-05	$CH_3CH_3 \rightarrow CH_2CH_3+H$	C-H	1.21	0.41	0.35	0.39	0.69	-0.39	-0.26	-0.35
1-06	$CH_3CH_3 \rightarrow CH_3+CH_3$	C-C	-	-	-	-	0.91	0.05	0.32	-0.16
1-07	$CH_3CH_2OH \rightarrow OCH_2CH_3+H$	O-H	0.90	0.43	0.71	0.60	-0.24	-0.78	0.24	0.41
1-08	$CH_3CH_2OH \rightarrow HOCHCH_3+H$	C-H	1.23	0.62	0.37	0.37	0.72	-0.28	-0.47	-0.50
1-09	$CH_3CH_2OH \rightarrow CH_2CH_2OH+H$	C-H	1.34	0.42	0.52	0.62	0.62	-0.42	-0.16	-0.24
1-10	$CH_3CH_2OH \rightarrow CH_2CH_3+OH$	C-OH	1.45	1.44	1.72	2.06	0.11	-0.63	0.14	0.24
1-11	$CH_3CH_2OH \rightarrow CH_3+CH_2OH$	C-C	-	-	-	-	1.29	0.17	0.31	-0.11
2-01	$HOCHCH_2OH \rightarrow CHOH+CH_2OH$	C-C	2.08	1.31	1.66	1.21	1.36	-0.16	-0.03	-0.24
2-02	$HOCHCH_2OH \rightarrow HOCCH_2OH+H$	C-H	0.79	0.17	0.78	0.67	0.34	-0.54	-0.38	-0.45
2-03	$HOCHCH_2OH \rightarrow HOCHCHOH+H$	C-H	0.77	0.37	0.41	0.80	-0.15	-0.48	-0.61	-0.55
2-04	$HOCHCH_2OH \rightarrow OCHCH_2OH+H$	O-H	0.78	0.50	0.56	0.43	-0.16	-0.71	-0.25	0.08
2-05	$HOCHCH_2OH \rightarrow OCH_2CHOH+H$	O-H	1.07	0.51	0.66	0.58	0.09	-0.60	0.09	0.19

Supplementary Table 7 – continues from previous page

Tag	Reaction	Type	E_a Cu	E_a Ru	E_a Pd	E_a Pt	ΔE Cu	ΔE Ru	ΔE Pd	ΔE Pt
2-06	$HOCHCH_2OH \rightarrow CHCH_2OH+OH$	C-OH	1.06	0.92	1.21	0.97	-0.12	-0.85	0.22	0.45
2-07	$HOCHCH_2OH \rightarrow CH_2CHOH+OH$	C-OH	0.62	0.32	1.23	1.28	-0.69	-0.98	-0.20	0.06
2-08	$OCH_2CH_2OH \rightarrow CH_2O+CH_2OH$	C-C	1.92	2.04	1.59	1.44	1.61	0.28	-0.07	-0.37
2-09	$OCH_2CH_2OH \rightarrow OCH_2CHOH+H$	C-H	1.05	0.66	0.39	0.40	0.86	-0.20	-0.30	-0.60
2-10	$OCH_2CH_2OH \rightarrow OCHCH_2OH+H$	C-H	1.06	0.47	0.35	0.05	0.60	-0.31	-0.65	-0.70
2-11	$OCH_2CH_2OH \rightarrow OCH_2CH_2O+H$	O-H	0.88	0.43	0.98	0.72	0.07	-0.39	0.38	0.23
2-12	$OCH_2CH_2OH \rightarrow CH_2CH_2OH+O$	C-O	1.74	1.36	1.58	1.68	0.63	-0.99	-0.15	-0.30
2-13	$OCH_2CH_2OH \rightarrow CH_2CH_2O+OH$	C-OH	1.78	1.48	1.78	1.40	0.22	-0.50	0.26	0.09
2-14	$CH_2CH_2OH \rightarrow CH_2+CH_2OH$	C-C	1.83	1.20	1.91	1.76	1.28	-0.07	0.16	0.11
2-15	$CH_2CH_2OH \rightarrow CHCH_2OH+H$	C-H	1.06	0.38	0.57	0.79	0.50	-0.34	-0.34	-0.15
2-16	$CH_2CH_2OH \rightarrow CH_2CHOH+H$	C-H	0.68	0.34	0.40	0.51	-0.08	-0.47	-0.75	-0.55
2-17	$CH_2CH_2OH \rightarrow CH_2CH_2O+H$	O-H	0.97	0.54	0.69	0.63	0.07	-0.39	0.10	0.27
2-18	$CH_2CH_2OH \rightarrow CH_2CH_2+OH$	C-OH	0.42	0.83	1.05	1.20	-0.51	-0.78	-0.29	0.08
2-19	$CH_2CH_3 \rightarrow CHCH_3+H$	C-H	1.04	0.19	0.96	0.76	0.50	-0.63	-0.36	-0.17
2-20	$CH_2CH_3 \rightarrow CH_2CH_2+H$	C-H	0.54	0.04	0.34	0.52	0.00	-0.57	-0.59	-0.40
2-21	$CH_2CH_3 \rightarrow CH_2+CH_3$	C-C	1.44	0.87	1.22	1.55	0.83	-0.22	0.27	0.18
2-22	$OCH_2CH_3 \rightarrow CH_2CH_2O+H$	C-H	1.45	0.64	0.43	0.53	0.93	-0.03	-0.30	-0.38
2-23	$OCH_2CH_3 \rightarrow OCHCH_3+H$	C-H	0.94	0.51	0.08	0.01	0.63	-0.21	-0.99	-0.72
2-24	$OCH_2CH_3 \rightarrow CH_2CH_3+O$	C-O	1.68	1.03	1.42	1.78	0.84	-0.73	-0.40	-0.29
2-25	$OCH_2CH_3 \rightarrow CH_2O+CH_3$	C-C	2.36	2.16	1.90	1.43	1.36	0.39	-0.21	-0.29
2-26	$HOCHCH_3 \rightarrow CH_2CHOH+H$	C-H	0.60	0.36	0.46	0.85	-0.17	-0.61	-0.44	-0.29
2-27	$HOCHCH_3 \rightarrow HOCCH_3+H$	C-H	0.87	0.18	0.30	0.32	0.20	-0.41	-0.66	-0.44
2-28	$HOCHCH_3 \rightarrow OCHCH_3+H$	O-H	0.54	0.46	0.52	0.56	-0.32	-0.71	-0.28	0.19
2-29	$HOCHCH_3 \rightarrow CHCH_3+OH$	C-OH	1.00	0.47	1.37	1.17	-0.10	-0.98	0.25	0.58
2-30	$HOCHCH_3 \rightarrow CHOH+CH_3$	C-C	1.35	1.11	1.55	1.45	0.93	-0.15	0.14	-0.03
3-01	$HOCCH_2OH \rightarrow COH+CH_2OH$	C-C	1.57	0.85	1.11	1.45	0.84	-0.45	-0.63	-0.54
3-02	$HOCCH_2OH \rightarrow HOCCHOH+H$	C-H	0.79	0.38	0.41	0.56	-0.10	-0.49	-0.76	-0.56
3-03	$HOCCH_2OH \rightarrow OCCH_2OH+H$	O-H	0.32	0.40	0.44	0.07	-0.55	-0.81	-0.80	-0.04
3-04	$HOCCH_2OH \rightarrow HOCCH_2O+H$	O-H	0.75	0.40	0.71	0.50	-0.07	-0.59	-0.01	0.28
3-05	$HOCCH_2OH \rightarrow CCH_2OH+OH$	C-OH	0.91	0.61	0.81	1.00	-0.17	-0.92	-0.28	0.40
3-06	$HOCCH_2OH \rightarrow HOCCH_2+OH$	C-OH	1.19	0.84	1.65	2.06	-0.62	-0.91	-0.34	0.02
3-07	$HOCHCHOH \rightarrow CHOH+CHOH$	C-C	1.99	0.89	1.38	1.01	1.87	-0.28	-0.07	-0.11
3-08	$HOCHCHOH \rightarrow HOCCHOH+H$	C-H	0.97	0.17	0.56	0.51	0.39	-0.55	-0.53	-0.46
3-09	$HOCHCHOH \rightarrow OCHCHOH+H$	O-H	0.48	0.20	0.49	0.59	-0.22	-0.80	-0.27	0.12
3-10	$HOCHCHOH \rightarrow CHCHOH+OH$	C-OH	1.48	0.65	1.19	1.04	-0.37	-1.01	0.28	0.52
3-11	$OCHCH_2OH \rightarrow CHO+CH_2OH$	C-C	1.41	0.95	0.99	1.16	1.12	0.04	-0.34	-0.53
3-12	$OCHCH_2OH \rightarrow OCCH_2OH+H$	C-H	0.71	0.21	0.34	0.27	-0.04	-0.64	-0.93	-0.56
3-13	$OCHCH_2OH \rightarrow OCHCHOH+H$	C-H	0.51	0.45	0.25	0.41	-0.21	-0.56	-0.62	-0.51
3-14	$OCHCH_2OH \rightarrow OCHCH_2O+H$	O-H	0.94	0.50	0.68	0.77	-0.30	-0.39	0.27	0.37
3-15	$OCHCH_2OH \rightarrow CHCH_2OH+O$	C-O	1.43	0.81	1.92	1.67	0.53	-1.02	0.16	0.24
3-16	$OCHCH_2OH \rightarrow OCHCH_2+OH$	C-OH	0.52	0.67	1.15	1.86	-0.82	-0.98	-0.15	0.15
3-17	$OCH_2CHOH \rightarrow CH_2O+CHOH$	C-C	1.42	1.31	0.95	0.80	1.11	-0.12	-0.41	-0.19
3-18	$OCH_2CHOH \rightarrow OCHCHOH+H$	C-H	0.76	0.44	0.21	0.08	-0.47	-0.67	-0.97	-0.61
3-19	$OCH_2CHOH \rightarrow HOCCH_2O+H$	C-H	0.76	0.65	0.47	0.58	0.17	-0.52	-0.48	-0.35
3-20	$OCH_2CHOH \rightarrow OCHCH_2O+H$	O-H	0.69	0.45	0.48	0.74	-0.56	-0.50	-0.08	0.27
3-21	$OCH_2CHOH \rightarrow CHCH_2O+OH$	C-OH	1.11	0.48	1.36	1.13	-0.07	-0.50	0.33	0.56
3-22	$OCH_2CHOH \rightarrow CH_2CHOH+O$	C-O	0.60	0.66	1.14	1.31	-0.30	-1.26	-0.60	-0.25

			E_a				ΔE			
Tag	Reaction	Type	Cu	Ru	Pd	Pt	Cu	Ru	Pd	Pt
3-23	$CHCH_2OH \rightarrow CH+CH_2OH$	C-C	1.60	0.82	1.27	1.32	1.09	-0.32	-0.32	-0.50
3-24	$CHCH_2OH \rightarrow CCH_2OH+H$	C-H	0.93	0.02	0.18	0.36	0.28	-0.60	-0.87	-0.50
3-25	$CHCH_2OH \rightarrow CHCHOH+H$	C-H	0.63	0.10	0.32	0.73	-0.40	-0.63	-0.55	-0.48
3-26	$CHCH_2OH \rightarrow CHCH_2O+H$	O-H	0.93	0.50	0.72	0.56	0.14	-0.25	0.21	0.29
3-27	$CHCH_2OH \rightarrow CHCH_2+OH$	C-OH	0.61	0.76	1.22	1.53	-0.52	-0.92	-0.12	0.10
3-28	$CH_2CHOH \rightarrow CH_2+CHOH$	C-C	1.85	1.41	1.49	1.35	1.71	-0.20	0.26	0.24
3-29	$CH_2CHOH \rightarrow CHCHOH+H$	C-H	1.18	0.52	0.75	0.61	0.18	-0.51	-0.14	-0.09
3-30	$CH_2CHOH \rightarrow HOCCH_2+H$	C-H	1.08	0.53	0.69	0.81	0.41	-0.46	-0.52	-0.48
3-31	$CH_2CHOH \rightarrow OCHCH_2+H$	O-H	0.65	0.59	0.51	0.54	-0.29	-0.71	-0.21	0.18
3-32	$CH_2CHOH \rightarrow CHCH_2+OH$	C-OH	1.57	1.13	1.30	1.49	0.06	-0.79	0.30	0.49
3-33	$OCH_2CH_2O \rightarrow CH_2O+CH_2O$	C-C	2.37	1.65	0.54	0.67	1.38	0.11	-0.74	-0.36
3-34	$OCH_2CH_2O \rightarrow OCHCH_2O+H$	C-H	0.80	0.58	0.01	0.21	0.23	-0.31	-0.76	-0.56
3-35	$OCH_2CH_2O \rightarrow CH_2CH_2O+O$	C-O	1.65	1.00	1.35	1.71	0.63	-0.99	-0.43	-0.27
3-36	$CH_2CH_2O \rightarrow CH_2+CH_2O$	C-C	1.31	0.93	1.01	1.41	1.05	-0.24	-0.23	0.08
3-37	$CH_2CH_2O \rightarrow CHCH_2O+H$	C-H	1.09	0.52	0.58	0.54	0.56	-0.20	-0.23	-0.13
3-38	$CH_2CH_2O \rightarrow OCHCH_2+H$	C-H	0.46	0.41	0.01	0.01	-0.45	-0.79	-1.06	-0.64
3-39	$CH_2CH_2O \rightarrow CH_2CH_2+O$	C-O	0.63	0.70	0.75	1.31	-0.09	-1.27	-0.69	-0.31
3-40	$CH_2CH_2 \rightarrow CH_2+CH_2$	C-C	1.77	0.96	1.72	1.97	1.45	-0.31	0.54	0.56
3-41	$CH_2CH_2 \rightarrow CHCH_2+H$	C-H	1.26	0.30	0.73	0.65	0.49	-0.48	-0.17	-0.14
3-42	$CHCH_3 \rightarrow CCH_3+H$	C-H	0.81	0.01	0.06	0.26	0.06	-0.74	-1.08	-0.93
3-43	$CHCH_3 \rightarrow CHCH_2+H$	C-H	0.65	0.13	0.46	0.44	-0.01	-0.42	-0.40	-0.37
3-44	$CHCH_3 \rightarrow CH+CH_3$	C-C	1.29	0.85	1.33	1.53	0.64	-0.19	-0.19	-0.42
3-45	$OCHCH_3 \rightarrow OCHCH_2+H$	C-H	0.56	0.03	0.66	0.64	-0.15	-0.61	-0.37	-0.30
3-46	$OCHCH_3 \rightarrow OCCH_3+H$	C-H	0.45	0.01	0.14	0.01	0.01	-0.66	-0.98	-0.90
3-47	$OCHCH_3 \rightarrow CHCH_3+O$	C-O	1.37	0.79	1.63	1.48	0.70	-1.14	0.23	0.26
3-48	$OCHCH_3 \rightarrow CHO+CH_3$	C-C	1.41	0.77	1.48	1.31	0.85	0.05	-0.15	-0.43
3-49	$HOCCH_3 \rightarrow HOCCH_2+H$	C-H	0.75	0.17	0.60	0.59	0.04	-0.66	-0.31	-0.33
3-50	$HOCCH_3 \rightarrow OCCH_3+H$	O-H	0.24	0.31	0.52	0.01	-0.51	-0.96	-0.60	-0.27
3-51	$HOCCH_3 \rightarrow CCH_3+OH$	C-OH	0.29	0.26	1.04	0.99	-0.24	-1.30	-0.17	0.09
3-52	$HOCCH_3 \rightarrow COH+CH_3$	C-C	1.69	0.99	1.51	1.57	0.54	-0.56	-0.18	-0.34
4-01	$HOCCHOH \rightarrow COH+CHOH$	C-C	1.77	0.74	1.59	1.85	1.29	-0.56	-0.51	-0.40
4-02	$HOCCHOH \rightarrow HOC-COH+H$	C-H	0.97	0.07	0.67	0.91	-0.07	-0.67	-0.41	-0.21
4-03	$HOCCHOH \rightarrow OCCHOH+H$	O-H	0.94	0.48	0.47	0.41	-0.16	-0.73	-0.27	-0.07
4-04	$HOCCHOH \rightarrow OCHCOH+H$	O-H	0.98	0.40	0.47	0.71	-0.23	-0.62	-0.03	0.19
4-05	$HOCCHOH \rightarrow CCHOH+OH$	C-OH	1.13	0.69	1.35	1.47	-0.51	-1.02	0.14	0.47
4-06	$HOCCHOH \rightarrow CHCOH+OH$	C-OH	1.29	0.67	1.28	1.28	-0.59	-0.79	0.22	0.51
4-07	$OCCH_2OH \rightarrow CO+CH_2OH$	C-C	1.19	0.73	0.93	0.81	0.16	-0.50	-0.91	-1.11
4-08	$OCCH_2OH \rightarrow OCCHOH+H$	C-H	1.04	0.21	0.72	0.38	0.28	-0.41	-0.23	-0.60
4-09	$OCCH_2OH \rightarrow OCCH_2O+H$	O-H	0.86	0.43	0.69	0.53	-0.02	-0.30	0.26	-0.05
4-10	$OCCH_2OH \rightarrow CCH_2OH+O$	C-O	2.09	1.09	1.88	1.78	0.86	-0.98	0.22	0.31
4-11	$OCCH_2OH \rightarrow OCCH_2+OH$	C-OH	1.18	0.97	1.71	1.51	-0.19	-0.76	0.15	-0.12
4-12	$HOCCH_2O \rightarrow COH+CH_2O$	C-C	1.20	0.92	1.00	1.02	0.75	-0.42	-0.91	-0.59
4-13	$HOCCH_2O \rightarrow OCHCOH+H$	C-H	0.62	0.25	0.25	0.14	-0.26	-0.52	-0.78	-0.65
4-14	$HOCCH_2O \rightarrow OCCH_2O+H$	O-H	0.50	0.86	0.25	0.22	-0.50	-0.52	-0.53	-0.37
4-15	$HOCCH_2O \rightarrow CCH_2O+OH$	C-OH	1.70	0.94	1.26	1.24	0.56	-0.53	0.50	0.80
4-16	$HOCCH_2O \rightarrow HOCCH_2+O$	C-O	1.21	1.22	1.34	1.86	-0.06	-1.20	-0.64	-0.39
4-17	$CCH_2OH \rightarrow C+CH_2OH$	C-C	2.36	1.04	1.54	1.29	1.92	0.22	0.68	0.35

			E_a				ΔE			
Tag	Reaction	Type	Cu	Ru	Pd	Pt	Cu	Ru	Pd	Pt
4-18	CCH_2OH→CCHOH+H	C-H	0.63	0.01	0.62	0.61	-0.44	-0.59	-0.34	-0.49
4-19	CCH_2OH→CCH_2O+H	O-H	1.41	0.66	1.10	0.96	0.66	-0.20	0.76	0.68
4-20	CCH_2OH→CCH_2+OH	C-OH	0.53	0.60	1.39	1.43	-0.57	-0.86	0.25	0.26
4-21	$HOCCH_2$→COH+CH_2	C-C	1.75	0.96	1.86	2.04	1.12	-0.56	-0.19	-0.03
4-22	$HOCCH_2$→CHCOH+H	C-H	0.85	0.12	0.73	1.00	-0.07	-0.37	-0.19	-0.08
4-23	$HOCCH_2$→$OCCH_2$+H	O-H	0.72	0.53	0.45	0.42	-0.12	-0.66	-0.31	-0.18
4-24	$HOCCH_2$→CCH_2+OH	C-OH	1.19	0.71	1.40	1.45	-0.13	-0.87	0.31	0.64
4-25	OCHCHOH→CHO+CHOH	C-C	2.00	1.20	1.05	0.73	1.69	0.00	-0.37	-0.44
4-26	OCHCHOH→OCCHOH+H	C-H	1.23	0.07	0.34	0.32	0.45	-0.49	-0.53	-0.65
4-27	OCHCHOH→OCHCOH+H	C-H	1.24	0.29	0.91	0.82	0.38	-0.37	-0.29	-0.39
4-28	OCHCHOH→OCHCHO+H	O-H	0.69	0.46	0.69	0.81	-0.11	-0.64	-0.10	0.26
4-29	OCHCHOH→CHCHOH+O	C-O	1.79	1.44	1.76	1.68	0.34	-1.09	0.24	0.27
4-30	OCHCHOH→CHCHO+OH	C-OH	1.31	0.75	1.67	1.41	-0.35	-0.75	0.27	0.35
4-31	CHCHOH→CH+CHOH	C-C	2.07	0.67	1.43	1.25	1.84	-0.29	-0.42	-0.44
4-32	CHCHOH→CCHOH+H	C-H	0.96	0.10	0.44	0.40	0.25	-0.56	-0.66	-0.51
4-33	CHCHOH→CHCOH+H	C-H	1.16	0.61	0.35	0.52	0.17	-0.32	-0.58	-0.47
4-34	CHCHOH→CHCHO+H	O-H	0.62	0.35	0.63	0.83	-0.21	-0.54	-0.28	-0.04
4-35	CHCHOH→CHCH+OH	C-OH	1.48	0.70	0.89	0.89	-0.23	-0.92	0.07	0.46
4-36	$OCHCH_2O$→CHO+CH_2O	C-C	1.66	0.84	0.49	0.48	1.26	-0.13	-0.90	-0.66
4-37	$OCHCH_2O$→$OCCH_2O$+H	C-H	0.67	0.11	0.20	0.41	0.23	-0.54	-0.94	-0.99
4-38	$OCHCH_2O$→OCHCHO+H	C-H	0.69	0.53	0.13	0.11	-0.02	-0.81	-0.99	-0.62
4-39	$OCHCH_2O$→$CHCH_2O$+O	C-O	2.04	0.94	1.39	1.34	0.97	-0.88	0.10	0.16
4-40	$OCHCH_2O$→$OCHCH_2$+O	C-O	0.93	0.55	0.73	1.30	-0.04	-1.47	-0.73	-0.35
4-41	$OCHCH_2$→CHO+CH_2	C-C	1.91	1.06	1.59	1.28	1.61	0.00	-0.10	-0.15
4-42	$OCHCH_2$→$OCCH_2$+H	C-H	0.98	0.10	0.36	0.18	0.59	-0.41	-0.63	-0.84
4-43	$OCHCH_2$→CHCHO+H	C-H	1.08	0.27	0.82	0.90	0.27	-0.33	-0.20	-0.31
4-44	$OCHCH_2$→$CHCH_2$+O	C-O	2.09	1.27	1.81	1.61	0.84	-0.96	0.20	0.19
4-45	$CHCH_2O$→CH+CH_2O	C-C	1.10	0.80	0.63	1.00	0.79	-0.63	-0.82	-0.55
4-46	$CHCH_2O$→CCH_2O+H	C-H	1.57	0.22	0.53	0.88	0.80	-0.55	-0.32	-0.10
4-47	$CHCH_2O$→CHCHO+H	C-H	0.64	0.01	0.30	0.47	-0.74	-0.92	-1.03	-0.81
4-48	$CHCH_2O$→$CHCH_2$+O	C-O	0.76	0.49	0.86	1.38	-0.17	-1.55	-0.63	-0.32
4-49	$CHCH_2$→CH+CH_2	C-C	1.70	0.99	1.16	1.47	1.26	-0.42	-0.11	-0.07
4-50	$CHCH_2$→CCH_2+H	C-H	0.87	0.04	0.40	0.73	0.23	-0.54	-0.51	-0.34
4-51	$CHCH_2$→CHCH+H	C-H	0.77	0.56	0.59	0.81	-0.12	-0.63	-0.37	-0.12
4-52	CCH_3→CCH_2+H	C-H	0.98	0.28	1.06	0.96	0.16	-0.22	0.17	0.22
4-53	CCH_3→C+CH_3	C-C	2.27	1.30	1.85	1.77	1.69	0.50	1.01	0.87
4-54	$OCCH_3$→$OCCH_2$+H	C-H	1.07	0.32	0.90	0.73	0.43	-0.36	-0.02	-0.24
4-55	$OCCH_3$→CCH_3+O	C-O	1.56	0.77	1.85	1.95	0.76	-1.22	0.13	0.22
4-56	$OCCH_3$→CO+CH_3	C-C	1.00	0.86	1.07	1.28	-0.17	-0.47	-0.66	-0.68
5-01	HOC−COH→COH+COH	C-C	1.75	0.45	0.94	1.03	1.17	-0.72	-1.08	-0.95
5-02	HOC−COH→OC−COH+H	O-H	1.25	0.86	0.63	0.49	0.18	-0.46	-0.45	-0.29
5-03	HOC−COH→C−COH+OH	C-OH	1.60	1.10	1.91	1.88	0.19	-0.39	1.04	1.25
5-04	OCCHOH→CO+CHOH	C-C	1.02	0.97	0.37	0.45	0.23	-0.69	-1.33	-0.93
5-05	OCCHOH→OC−COH+H	C-H	1.09	0.89	0.51	0.51	0.27	-0.40	-0.58	-0.43
5-06	OCCHOH→OCCHO+H	O-H	1.28	0.53	0.63	0.63	0.24	-0.46	-0.32	0.25
5-07	OCCHOH→OCCH+OH	C-OH	1.26	0.74	1.05	1.44	-0.75	-0.84	-0.05	0.28
5-08	OCCHOH→CCHOH+O	C-O	1.63	1.33	1.73	1.96	0.14	-1.16	0.11	0.41

			E_a				ΔE			
Tag	Reaction	Type	Cu	Ru	Pd	Pt	Cu	Ru	Pd	Pt
5-09	OCHCOH→CHO+COH	C-C	1.55	0.61	0.62	0.88	1.12	-0.45	-1.05	-0.80
5-10	OCHCOH→OC−COH+H	C-H	0.99	0.65	0.21	0.48	0.34	-0.51	-0.82	-0.69
5-11	OCHCOH→OCCHO+H	O-H	0.93	0.46	0.47	0.56	0.31	-0.58	-0.56	-0.01
5-12	OCHCOH→CHCOH+O	C-O	1.77	1.06	1.83	1.97	0.13	-1.04	-0.05	0.19
5-13	OCHCOH→CCHO+OH	C-OH	1.20	0.71	1.18	1.30	0.06	-0.48	0.08	0.45
5-14	CCHOH→C+CHOH	C-C	2.94	1.09	1.77	1.52	2.71	0.21	0.37	0.42
5-15	CCHOH→C−COH+H	C-H	1.46	0.99	1.30	1.43	0.63	-0.03	0.49	0.58
5-16	CCHOH→CCHO+H	O-H	0.89	0.55	0.44	0.37	0.33	-0.08	-0.10	0.18
5-17	CCHOH→CCH+OH	C-OH	1.05	0.74	2.05	1.61	0.01	-0.51	1.10	1.60
5-18	CHCOH→CH+COH	C-C	1.99	0.34	1.06	1.20	1.49	-0.79	-0.81	-0.72
5-19	CHCOH→C−COH+H	C-H	1.67	0.46	1.21	1.41	0.71	-0.27	0.41	0.54
5-20	CHCOH→OCCH+H	O-H	1.08	0.62	0.54	0.58	-0.32	-0.79	-0.54	-0.30
5-21	CHCOH→CCH+OH	C-OH	1.36	1.24	1.63	1.80	0.10	-0.75	1.02	1.57
5-22	$OCCH_2O$→CO+CH_2O	C-C	0.73	0.70	0.26	0.66	0.03	-0.77	-1.46	-0.82
5-23	$OCCH_2O$→OCCHO+H	C-H	0.87	0.01	0.26	0.16	0.55	-0.58	-0.81	-0.29
5-24	$OCCH_2O$→$OCCH_2$+O	C-O	1.45	0.80	1.35	2.02	0.32	-1.34	-0.42	-0.19
5-25	$OCCH_2O$→CCH_2O+O	C-O	2.72	1.35	2.18	2.39	1.54	-0.89	0.72	1.04
5-26	$OCCH_2$→CO+CH_2	C-C	0.83	0.82	1.11	0.99	0.02	-0.77	-0.97	-0.45
5-27	$OCCH_2$→OCCH+H	C-H	0.81	0.51	0.55	0.57	-0.27	-0.50	-0.42	-0.20
5-28	$OCCH_2$→CCH_2+O	C-O	1.61	1.16	2.86	2.28	0.48	-1.09	0.32	0.68
5-29	CCH_2O→C+CH_2O	C-C	1.67	0.96	0.81	1.05	1.10	-0.14	-0.38	-0.10
5-30	CCH_2O→CCHO+H	C-H	0.38	0.19	0.06	0.37	-0.76	-0.47	-1.20	-1.00
5-31	CCH_2O→CCH_2+O	C-O	0.44	0.44	0.59	1.00	-0.75	-1.53	-0.82	-0.55
5-32	CCH_2→C+CH_2	C-C	2.62	1.46	2.35	2.36	2.15	0.06	0.52	0.63
5-33	CCH_2→CCH+H	C-H	0.97	0.63	1.16	1.29	0.15	-0.25	0.52	0.85
5-34	OCHCHO→CHO+CHO	C-C	1.56	0.93	0.79	0.80	1.39	0.12	-0.83	-0.90
5-35	OCHCHO→OCCHO+H	C-H	1.39	0.99	0.56	0.32	0.80	-0.31	-0.76	-0.66
5-36	OCHCHO→CHCHO+O	C-O	1.80	1.25	1.51	1.31	0.25	-0.99	0.06	-0.03
5-37	CHCHO→CH+CHO	C-C	1.92	0.37	0.69	0.86	1.65	-0.27	-0.71	-0.60
5-38	CHCHO→CCHO+H	C-H	1.56	0.41	0.58	0.77	0.79	-0.10	-0.48	-0.29
5-39	CHCHO→OCCH+H	C-H	0.80	0.79	0.26	0.67	0.05	-0.58	-0.85	-0.73
5-40	CHCHO→CHCH+O	C-O	1.83	0.97	1.98	1.78	0.46	-1.26	0.04	0.37
5-41	CHCH→CH+CH	C-C	2.23	0.88	1.12	1.30	1.69	-0.39	-0.57	-0.71
5-42	CHCH→CCH+H	C-H	1.52	0.70	1.01	1.32	0.49	-0.16	0.37	0.63
6-01	OCCOH→CO+COH	C-C	0.47	0.16	0.22	0.63	-0.22	-1.12	-1.72	-1.25
6-02	OCCOH→OC−CO+H	O-H	1.01	1.01	0.45	0.39	0.16	-0.28	-0.18	-0.13
6-03	OCCOH→C−COH+O	C-O	2.29	1.90	2.90	2.89	0.50	-0.80	1.18	1.42
6-04	OCCOH→C−CO+OH	C-OH	0.76	1.39	1.28	1.82	-1.08	-0.72	0.30	0.74
6-05	CCOH→C+COH	C-C	2.51	0.69	0.83	0.95	1.89	-0.58	-1.09	-0.91
6-06	CCOH→CCO+H	O-H	0.43	0.81	0.38	0.48	-1.10	-0.79	-1.18	-0.81
6-07	CCOH→CC+OH	C-OH	1.84	1.86	2.65	3.13	-0.11	-0.12	0.79	1.59
6-08	OCCHO→CO+CHO	C-C	0.56	0.77	0.28	0.11	-0.41	-0.74	-1.57	-1.39
6-09	OCCHO→OC−CO+H	C-H	0.64	0.74	0.50	0.30	0.20	-0.21	-0.44	-0.81
6-10	OCCHO→CCHO+O	C-O	1.60	1.63	2.00	2.02	0.24	-0.78	0.33	0.33
6-11	OCCHO→OCCH+O	C-O	1.31	1.22	2.18	2.28	-0.50	-1.25	-0.03	-0.11
6-12	OCCH→CO+CH	C-C	1.43	0.52	0.69	0.73	0.60	-0.87	-1.36	-1.02
6-13	OCCH→C−CO+H	C-H	0.73	1.15	0.66	1.04	-0.06	-0.27	-0.23	0.03

Tag	Reaction	Type	E_a Cu	E_a Ru	E_a Pd	E_a Pt	ΔE Cu	ΔE Ru	ΔE Pd	ΔE Pt
6-14	OCCH→CCH+O	C-O	2.62	1.61	2.72	3.11	0.90	-0.84	1.26	1.74
6-15	CCHO→C+CHO	C-C	2.42	0.51	1.09	0.97	1.97	-0.22	-0.10	0.04
6-16	CCHO→C−CO+H	C-H	0.65	0.22	0.43	0.59	-0.80	-0.75	-0.60	-0.41
6-17	CCHO→CCH+O	C-O	1.41	0.74	2.02	2.38	0.17	-1.31	0.89	1.30
6-18	CCH→C+CH	C-C	3.00	1.19	1.13	0.71	2.31	-0.29	-0.81	-0.99
6-19	CCH→C−C+H	C-H	1.38	1.31	0.92	1.63	0.51	0.36	0.17	0.56
7-01	C−C→C+C	C-C	3.72	1.46	1.42	0.73	2.91	-0.70	-0.86	-1.20
7-02	C−CO→C−C+O	C-O	3.05	2.09	4.36	4.45	1.47	-0.20	1.66	2.27
7-03	C−CO→C+CO	C-C	2.48	0.30	1.02	0.77	1.77	-0.65	-1.00	-0.69
7-04	OC−CO→CO+CO	C-C	0.06	0.02	0.00	0.05	-1.61	-1.71	-2.63	-1.72
7-05	OC−CO→C−CO+O	C-O	0.83	1.22	2.01	2.01	-0.76	-1.32	0.18	0.74
	O_2 and CO_2 decompositions and water-gas shift reaction									
8-01	H_2O + O →OH + OH	O-H	0.01	0.26	0.37	0.57	-0.66	0.30	0.30	0.57
8-02	O_2 → 2O	O-O	0.15	0.21	0.55	0.46	-2.45	-3.64	-2.17	-1.84
8-03	CO_2 → CO + O	C-O	1.44	0.64	0.95	0.96	0.49	-1.24	-0.80	-0.26
8-04	COOH → CO + OH	C-OH	0.42	0.62	0.74	0.65	-0.52	-0.70	-0.76	0.03
8-05	COOH →CO_2 + H	O-H	1.05	0.82	0.55	0.55	-0.52	-0.25	-0.32	0.12
8-06	COOH + O →CO_2+OH	O-H	-0.21	0.67	0.59	0.89	-1.00	0.54	0.04	0.29
8-07	COOH + OH →CO_2+H_2O	O-H	-0.07	0.24	0.36	0.55	-0.34	0.24	-0.26	-0.29
8-08	H_2O →OH + H	O-H	1.03	0.58	0.81	0.69	-0.18	-0.57	-0.02	0.43
8-09	OH →O + H	O-H	1.39	0.66	0.78	1.37	0.48	-0.88	-0.31	-0.13
	Methanol and methane decomposition reactions on Cu, Ru, Pd, and Pt surfaces									
9-01	CH_3OH→CH_3O+H	O-H	0.96	0.50	0.67	0.59	-0.22	-0.71	-0.13	0.35
9-02	CH_2OH→CH_2O+H	O-H	0.68	0.34	0.58	0.62	-0.16	-0.56	-0.29	0.23
9-03	CHOH→CHO+H	O-H	0.44	0.57	0.49	0.19	-0.40	-0.51	-0.57	-0.21
9-04	COH→CO+H	O-H	1.18	0.73	0.71	0.78	-1.22	-0.87	-1.09	-0.60
9-05	CH_3OH→CH_2OH+H	C-H	1.13	0.47	0.41	0.53	0.67	-0.37	-0.42	-0.47
9-06	CH_3O→CH_2O+H	C-H	1.04	0.71	0.38	0.08	0.73	-0.22	-0.58	-0.58
9-07	CH_2OH→CHOH+H	C-H	0.99	0.18	0.37	0.40	0.35	-0.60	-0.65	-0.42
9-08	CH_2O→CHO+H	C-H	0.54	0.09	0.22	0.14	0.11	-0.55	-0.92	-0.86
9-09	CHOH→COH+H	C-H	0.52	0.01	0.23	0.52	-0.18	-0.82	-0.98	-0.75
9-10	CHO→CO+H	C-H	0.11	0.09	0.22	0.22	-1.00	-1.18	-1.50	-1.15
9-11	CH_4→CH_3+H	C-H	1.36	0.61	0.43	0.50	0.52	-0.27	-0.17	-0.27
9-12	CH_3→CH_2+H	C-H	1.16	0.07	0.54	0.64	0.61	-0.66	-0.32	-0.02
9-13	CH_2→CH+H	C-H	0.84	0.00	0.17	0.05	0.31	-0.59	-0.82	-0.77
9-14	CH→C+H	C-H	1.74	0.69	1.30	1.24	1.11	-0.06	0.13	0.35
9-15	CH_3OH→CH_3+OH	C-OH	1.73	0.31	1.48	1.61	-0.28	-0.73	-0.01	0.09
9-16	CH_3O→CH_3+O	C-O	1.68	1.45	1.23	1.38	0.42	-0.90	-0.19	-0.39
9-17	CH_2OH→CH_2+OH	C-OH	0.91	0.64	1.15	1.36	-0.34	-1.02	0.09	0.53
9-18	CH_2O→CH_2+O	C-O	1.26	0.74	1.62	1.53	0.30	-1.34	0.08	0.17
9-19	CHOH→CH+OH	C-OH	0.25	0.50	1.04	1.09	-0.39	-1.02	-0.08	0.19
9-20	CHO→CH+O	C-O	1.45	0.84	1.80	1.92	0.50	-1.38	0.18	0.27
9-21	COH→C+OH	C-OH	2.15	1.11	2.19	2.47	0.91	-0.25	1.03	1.29
9-22	CO→C+O	C-O	3.65	1.92	3.64	3.65	2.62	-0.26	1.80	1.76
	Reactions from intramolecular H–bonded ethylene glycol (EG^H on Ru(0001):									
1-01†	EG^H →2 CH_2OH	C-C		-				0.04		
1-02†	EG^H →$HOCHCH_2OH$+H	C-H		0.51				-0.41		

Supplementary Table 7 – continues from previous page

			E_a				ΔE			
Tag	Reaction	Type	Cu	Ru	Pd	Pt	Cu	Ru	Pd	Pt
1-03†	$EG^H \rightarrow OCH_2CH_2OH+H$	O-H		0.28				-0.81		
1-04†	$EG^H \rightarrow CH_2CH_2OH+OH$	C-OH		1.13				-0.92		
	C–H breakings from ethanol assisted by coadsorbed O									
1-08'	$EtOH+O \rightarrow HOCHCH_3+OH$	C-H	1.37	0.33	1.45	0.73	1.22	-0.13	1.08	-0.33
1-09'	$EtOH+O \rightarrow CH_2CH_2OH+OH$	C-H	1.20	0.34	1.44	0.56	1.36	0.22	1.25	-0.05
	O–H breakings assisted by coadsorbed O and OH									
9-01'	$CH_3OH+OH \rightarrow CH_3O+H_2O$	O-H	0.10	0.10	0.10	0.10	-0.04	-0.16	-0.11	-0.09
9-01"	$CH_3OH+O \rightarrow CH_3O+OH$	O-H	0.36	0.17	0.30	0.48	-0.74	0.17	0.19	0.48
1-07'	$EtOH+OH \rightarrow CH_3CH_2O+H_2O$	O-H	0.10	0.10	0.10	0.10	-0.08	-0.20	0.01	-0.05
1-07"	$EtOH+O \rightarrow CH_3CH_2O+OH$	O-H	0.46	0.22	0.35	0.52	-0.77	0.13	0.31	0.52
1-03'	$EG+OH \rightarrow CH_2OHCH_2O+H_2O$	O-H	0.10	0.10	0.13	0.10	-0.04	-0.19	0.13	-0.08
1-03"	$EG+O \rightarrow CH_2OHCH_2O+OH$	O-H	0.48	0.17	0.44	0.49	-0.74	0.14	0.44	0.49

Supplementary Table 8: **Initial and final state scaling relationships as linear functions** Initial and final state scaling relationships for O–H, C–H, C–C, C–O, and C–OH bond breakings on Cu, Ru, Pd, and Pt closed-packed surfaces. The regression parameters: slope (α, dimensionless), and intercept (β, in eV), are defined by Supplementary Equations 37-39. The coefficient of determination R^2, dimensionless, and the mean absolute errors MAE, in eV, are included.

	BEP				ISS				FSS			
Metal	α	β	R^2	MAE	α	β	R^2	MAE	α	β	R^2	MAE
						O–H bonds						
Cu	0.39	0.89	0.27	0.18	0.93	0.84	0.66	0.22	0.72	1.00	0.60	0.19
Ru	0.23	0.65	0.08	0.12	0.87	0.28	0.95	0.12	0.89	0.83	0.88	0.18
Pd	0.27	0.65	0.43	0.09	1.06	0.69	0.96	0.12	0.80	0.41	0.90	0.19
Pt	0.32	0.52	0.21	0.13	1.06	0.64	0.90	0.14	0.79	0.19	0.88	0.16
M^1	0.18	0.60	0.16	0.12	0.99	0.54	0.94	0.14	0.73	0.27	0.85	0.23
M^2	0.24	0.67	0.16	0.16	1.08	0.72	0.96	0.17	0.92	0.73	0.90	0.28
						C–H bonds						
Cu	0.63	0.81	0.69	0.14	0.93	0.96	0.72	0.25	0.99	0.74	0.84	0.18
Ru	0.89	0.77	0.45	0.18	0.88	0.15	0.83	0.24	0.91	0.64	0.91	0.17
Pd	0.67	0.83	0.78	0.10	0.86	0.29	0.84	0.21	0.87	0.73	0.95	0.12
Pt	0.77	0.86	0.70	0.15	0.77	0.30	0.66	0.25	0.84	0.71	0.89	0.15
M^1	0.75	0.81	0.62	0.16	0.90	0.33	0.81	0.25	0.92	0.77	0.92	0.16
M^2	0.71	0.79	0.74	0.15	1.11	0.70	0.90	0.29	0.92	0.77	0.96	0.17
						C–C bonds						
Cu	0.78	0.88	0.80	0.23	1.31	1.59	0.63	0.43	0.89	0.70	0.93	0.19
Ru	0.84	1.25	0.46	0.26	1.22	1.40	0.87	0.23	0.96	1.12	0.90	0.22
Pd	0.69	1.51	0.71	0.24	1.18	1.46	0.77	0.35	0.81	1.16	0.93	0.21
Pt	0.73	1.50	0.53	0.27	1.11	1.32	0.69	0.34	0.82	1.23	0.84	0.26
M^1	0.70	1.40	0.52	0.29	1.20	1.43	0.81	0.31	0.89	1.22	0.87	0.28
M^2	0.56	1.30	0.64	0.30	1.27	1.56	0.91	0.34	0.79	0.96	0.95	0.27
						C–O bonds						
Cu	0.94	1.24	0.80	0.25	0.37	1.63	0.10	0.41	0.97	1.20	0.73	0.24
Ru	1.13	2.32	0.69	0.18	0.67	0.28	0.64	0.25	0.81	1.51	0.86	0.16
Pd	1.11	1.68	0.84	0.23	0.44	0.67	0.48	0.35	0.84	1.36	0.81	0.19
Pt	0.90	1.67	0.80	0.25	0.42	1.07	0.44	0.27	0.76	1.35	0.63	0.23
M^1	0.66	1.52	0.58	0.33	0.78	1.13	0.55	0.48	0.77	1.32	0.92	0.20
M^2	0.66	1.41	0.56	0.32	0.89	1.42	0.72	0.47	0.77	1.31	0.95	0.22
						C–OH bonds						
Cu	0.77	1.33	0.42	0.29	0.66	1.23	0.27	0.34	0.73	1.38	0.58	0.27
Ru	0.92	1.55	0.41	0.21	0.85	0.55	0.76	0.27	0.99	1.58	0.84	0.21
Pd	0.70	1.27	0.46	0.22	0.92	1.29	0.75	0.31	1.10	1.36	0.85	0.23
Pt	0.46	1.27	0.22	0.31	0.90	1.35	0.59	0.33	0.95	0.96	0.55	0.38
M^1	0.57	1.27	0.56	0.25	0.98	1.22	0.65	0.38	0.81	0.94	0.78	0.33
M^2	0.58	1.27	0.52	0.27	0.95	1.17	0.80	0.38	0.91	1.19	0.86	0.33
						C–O + C–OH bonds						
Cu	0.85	1.31	0.72	0.27	0.41	1.45	0.11	0.39	0.80	1.33	0.66	0.26
Ru	0.96	1.46	0.62	0.31	0.74	0.40	0.71	0.27	0.73	1.05	0.74	0.25
Pd	0.69	1.45	0.42	0.38	0.63	0.90	0.55	0.36	0.90	1.25	0.72	0.30
Pt	0.59	1.52	0.20	0.30	0.63	1.16	0.45	0.37	0.78	1.07	0.41	0.41
M^1	0.62	1.43	0.55	0.33	0.78	1.13	0.55	0.48	0.77	1.32	0.92	0.20
M^2	0.66	1.41	0.56	0.32	0.91	1.27	0.74	0.45	0.81	1.21	0.88	0.31

M^1: Ru, Pd, Pt

M^2: Cu, Ru, Pd, Pt

Supplementary Table 9: **Initial and final state scaling relationships with slope** $\alpha = 1$. Initial and final state scaling relationships for O–H, C–H, C–C, C–O, and C–OH bond breakings on Cu, Ru, Pd, and Pt closed-packed surfaces for $\alpha = 1$ on Supplementary Equation 38-39. The parameter β, in eV, its mean average errors, MAE, in eV, and the coefficient of determination R^2, dimensionless, are included.

	ISS			FSS		
Metal	β	R^2	MAE	β	R^2	MAE
			O–H bonds			
Cu	0.85	0.89	0.21	1.09	0.82	0.27
Ru	0.50	0.86	0.15	1.07	0.77	0.19
Pd	0.58	0.95	0.11	0.90	0.74	0.24
Pt	0.55	0.89	0.17	0.56	0.82	0.23
Ru, Pd, Pt	0.54	0.91	0.14	0.85	0.83	0.28
			C–H bonds			
Cu	0.99	0.84	0.25	0.81	0.91	0.19
Ru	0.35	0.77	0.23	0.80	0.86	0.18
Pd	0.52	0.83	0.24	1.07	0.93	0.15
Pt	0.58	0.78	0.29	1.00	0.93	0.17
Ru, Pd, Pt	0.48	0.82	0.26	0.96	0.93	0.18
			C–C bonds			
Cu	1.77	0.52	0.45	0.64	0.91	0.19
Ru	0.91	0.28	0.30	1.19	0.56	0.24
Pd	1.18	0.07	0.38	1.66	0.51	0.26
Pt	1.17	0.09	0.35	1.58	0.49	0.26
Ru, Pd, Pt	1.09	0.46	0.36	1.47	0.68	0.30
			C–O bonds			
Cu	1.64	0.41	0.53	1.24	0.89	0.23
Ru	1.03	0.58	0.29	2.13	0.82	0.19
Pd	1.73	0.18	0.49	1.71	0.87	0.19
Pt	1.83	0.27	0.44	1.63	0.80	0.23
Ru, Pd, Pt	1.53	0.68	0.50	1.82	0.92	0.27
			C–OH bonds			
Cu	1.20	0.68	0.36	1.48	0.80	0.28
Ru	0.85	0.48	0.30	1.59	0.83	0.22
Pd	1.44	0.66	0.33	1.26	0.87	0.20
Pt	1.48	0.61	0.38	0.98	0.64	0.36
Ru, Pd, Pt	1.26	0.70	0.41	1.27	0.81	0.34

Supplementary Table 10: **Outliers for O–H LSR**. Reactions with activation energies deviated more than 0.20 eV of the initial state scaling line: O–H breaking on Cu, Ru, Pd, and Pt.

Above the scaling line			Below the scaling line		
Metal	Reaction	Deviation	Metal	Reaction	Deviation
Pd	4-19	0.54	Pd	4-14	0.30
Pd	2-11	0.43	Pt	3-03	0.48
Pt	4-19	0.41	Pt	3-50	0.60
Pt	4-34	0.27	Pt	4-14	0.33
Pt	4-28	0.25	Pt	9-03	0.36
Pt	3-14	0.22	Ru	3-09	0.35
Pt	9-04	0.22	Ru	4-34	0.20
Ru	6-02	0.45	Ru	1-03	0.22
Ru	5-02	0.30	Ru	9-02	0.21
Ru	4-14	0.30	Ru	3-50	0.24
Ru	6-06	0.25	Cu	3-03	0.49
Cu	4-19	0.65	Cu	2-28	0.28
Cu	2-05	0.23	Cu	3-50	0.56
Cu	5-20	0.27	Cu	4-14	0.32
Cu	9-04	0.38	Cu	6-06	0.32
Cu	5-06	0.44	Cu	3-09	0.37
Cu	5-02	0.42	Cu	9-03	0.34

Supplementary Table 11: **Outliers for C–H LSR**. Reactions with activation energies deviated more than 0.20 eV of the final state scaling line: C–H breaking on Cu, Ru, Pd, and Pt.

Above the scaling line			Below the scaling line		
Metal	Reaction	Deviation	Metal	Reaction	Deviation
Cu	6-16	0.68	Cu	2-09	0.57
Cu	4-47	0.61	Cu	2-23	0.46
Cu	5-30	0.44	Cu	9-06	0.46
Cu	3-18	0.43	Cu	5-23	0.41
Cu	5-42	0.36	Cu	4-37	0.34
Cu	4-18	0.33	Cu	4-42	0.34
Cu	5-27	0.32	Cu	3-46	0.33
Cu	3-25	0.28	Cu	2-10	0.32
Cu	5-19	0.28	Cu	9-08	0.32
Cu	9-10	0.28	Cu	2-02	0.29
Cu	4-02	0.27	Cu	9-05	0.29
Cu	4-33	0.26	Cu	6-09	0.29
Cu	3-29	0.25	Cu	2-22	0.23
Cu	6-19	0.24	Cu	1-08	0.23
Pd	9-10	0.65	Cu	3-34	0.23
Pd	2-19	0.47	Cu	1-05	0.21
Pd	4-47	0.40	Pd	5-33	0.24
Pd	3-12	0.30	Pd	5-42	0.25
Pd	5-35	0.30	Pt	3-18	0.20
Pd	3-30	0.28	Pt	3-38	0.31
Pd	5-30	0.28	Pt	5-33	0.38
Pd	9-14	0.28	Pt	5-23	0.48
Pd	2-02	0.26	Ru	4-42	0.48
Pd	2-16	0.26	Ru	4-22	0.47
Pd	3-18	0.24	Ru	5-38	0.47
Pd	4-27	0.23	Ru	5-23	0.46
Pd	9-09	0.23	Ru	4-26	0.45
Pd	3-02	0.21	Ru	4-52	0.44
Pd	3-42	0.20	Ru	4-18	0.40
Pd	9-08	0.20	Ru	4-08	0.39
Pt	4-37	0.49	Ru	4-43	0.38
Pt	2-03	0.44	Ru	4-50	0.37
Pt	5-30	0.44	Ru	3-46	0.35
Pt	5-39	0.44	Ru	4-37	0.35
Pt	4-47	0.38	Ru	3-43	0.34
Pt	3-30	0.37	Ru	4-27	0.33
Pt	9-10	0.37	Ru	5-30	0.32
Pt	3-25	0.33	Ru	4-32	0.31
Pt	9-09	0.33	Ru	3-45	0.31
Pt	4-43	0.32	Ru	9-13	0.31
Pt	3-42	0.28	Ru	3-24	0.31
Pt	4-27	0.28	Ru	4-54	0.30
Pt	6-19	0.28	Ru	9-08	0.29
Pt	2-26	0.25	Ru	2-27	0.28
Pt	2-02	0.22	Ru	9-09	0.28
Ru	6-13	0.38	Ru	4-02	0.26
Ru	5-39	0.34	Ru	5-19	0.25
Ru	4-38	0.32	Ru	2-20	0.25
Ru	3-38	0.25	Ru	3-08	0.23
Ru	5-35	0.25	Ru	3-42	0.23
Ru	4-51	0.24	Ru	4-13	0.23
Ru	5-05	0.24	Ru	2-22	0.21
Ru	9-10	0.24			
Ru	3-19	0.21			

Supplementary Table 12: **Outliers for the C–C LSR**. Reactions with activation energies deviated more than 0.20 eV of the final state scaling line: C–C breaking on Cu, Ru, Pd, and Pt.

Above the scaling line			Below the scaling line		
Metal	Reaction	Deviation	Metal	Reaction	Deviation
Cu	6-18	0.51	Cu	5-34	0.60
Cu	3-52	0.40	Cu	3-07	0.46
Cu	6-05	0.30	Cu	3-11	0.46
Cu	5-32	0.22	Cu	2-08	0.46
Cu	4-53	0.20	Cu	3-28	0.43
Pd	4-21	0.60	Cu	3-36	0.42
Pd	5-32	0.56	Cu	3-17	0.39
Pd	4-01	0.52	Cu	4-36	0.39
Pd	2-08	0.52	Cu	4-25	0.36
Pd	6-18	0.49	Cu	4-41	0.35
Pd	2-01	0.47	Cu	5-37	0.32
Pd	4-31	0.43	Cu	4-45	0.30
Pd	4-12	0.40	Cu	5-22	0.29
Pd	5-26	0.38	Cu	4-12	0.29
Pd	3-48	0.34	Cu	5-09	0.29
Pd	3-23	0.33	Cu	2-30	0.28
Pd	3-44	0.32	Cu	4-31	0.28
Pd	3-52	0.32	Cu	6-01	0.23
Pd	4-41	0.32	Cu	3-48	0.23
Pd	3-01	0.31	Cu	3-40	0.22
Pd	6-05	0.30	Pd	4-17	0.38
Pd	2-30	0.26	Pd	4-53	0.34
Pd	5-18	0.24	Pd	6-15	0.34
Pd	5-41	0.22	Pt	6-15	0.42
Pd	5-01	0.22	Pt	4-17	0.27
Pt	3-44	0.74	Pt	5-04	0.26
Pt	4-01	0.74	Pt	4-53	0.24
Pt	4-21	0.70	Pt	3-07	0.22
Pt	2-08	0.70	Pt	4-25	0.22
Pt	5-41	0.62	Pt	3-17	0.21
Pt	3-01	0.59	Pt	5-14	0.21
Pt	5-32	0.57	Ru	5-37	0.93
Pt	3-52	0.55	Ru	6-15	0.84
Pt	3-23	0.55	Ru	5-34	0.79
Pt	3-48	0.50	Ru	6-01	0.63
Pt	4-56	0.46	Ru	5-14	0.59
Pt	3-11	0.39	Ru	5-09	0.59
Pt	6-05	0.38	Ru	3-48	0.58
Pt	5-18	0.38	Ru	5-01	0.55
Pt	6-18	0.37	Ru	4-17	0.52
Pt	4-07	0.37	Ru	4-36	0.51
Pt	3-40	0.35	Ru	4-31	0.50
Pt	4-31	0.32	Ru	5-18	0.49
Pt	4-45	0.32	Ru	4-53	0.46
Pt	2-21	0.32	Ru	3-11	0.44
Pt	4-49	0.31	Ru	6-12	0.43
Pt	5-01	0.30	Ru	6-05	0.38
Pt	2-30	0.29	Ru	4-07	0.37
Pt	3-36	0.27	Ru	4-41	0.37
Pt	5-34	0.26	Ru	5-29	0.36
Pt	4-12	0.24	Ru	6-08	0.33
Pt	2-01	0.20	Ru	4-25	0.28
Ru	2-08	0.52	Ru	5-22	0.26
Ru	3-28	0.28	Ru	5-41	0.26
Ru	2-01	0.22	Ru	4-01	0.27
			Ru	3-44	0.22
			Ru	4-56	0.22
			Ru	3-07	0.20

Supplementary Table 13: **Outliers for C–O and C–OH LSR**. Reactions with activation energies deviated more than 0.30 eV of the final state scaling line: C–O and C–OH breaking on Cu, Ru, Pd, and Pt. M, R, and D stand for Metal, Reaction, and Deviation, in eV.

Above (C–O)			Below (C–O)			Above (C–OH)			Below (C–OH)		
M	R	D	M	R	D	M	R	D	M	R	D
Cu	6-03	0.68	Cu	3-39	0.55	Cu	6-07	0.85	Cu	3-51	0.64
Cu	6-14	0.64	Cu	3-47	0.49	Cu	5-07	0.76	Cu	9-19	0.52
Cu	6-11	0.46	Cu	4-40	0.46	Cu	9-15	0.76	Cu	2-18	0.29
Cu	5-12	0.37	Cu	3-22	0.45	Cu	4-06	0.65	Pd	5-21	0.70
Pd	5-28	0.72	Cu	2-24	0.42	Cu	3-10	0.61	Pd	4-15	0.53
Pd	2-24	0.33	Cu	4-55	0.33	Cu	3-06	0.59	Pd	4-35	0.51
Pt	2-24	0.66	Cu	3-15	0.32	Cu	6-04	0.59	Pd	6-04	0.48
Pt	3-35	0.57	Cu	3-35	0.31	Cu	4-35	0.52	Pd	5-03	0.47
Pt	6-11	0.56	Pd	6-17	0.58	Cu	4-05	0.42	Pd	3-10	0.41
Pt	2-12	0.52	Pd	5-31	0.41	Cu	1-04	0.41	Pd	5-17	0.35
Pt	4-16	0.52	Pd	5-36	0.39	Cu	4-30	0.41	Pd	5-07	0.33
Pt	5-24	0.44	Pd	4-40	0.31	Cu	3-32	0.33	Pd	4-06	0.32
Pt	9-16	0.34	Pd	5-08	0.30	Cu	2-13	0.32	Pd	3-32	0.30
Pt	9-22	0.33	Pd	4-39	0.30	Pd	3-06	0.63	Pd	5-13	0.30
Ru	9-16	0.53	Pd	6-10	0.29	Pd	1-04	0.56	Pt	5-17	1.22
Ru	2-12	0.43	Pt	6-17	0.38	Pd	6-07	0.56	Pt	5-21	0.99
Ru	6-03	0.41	Pt	5-36	0.23	Pd	1-10	0.36	Pt	4-35	0.85
Ru	4-29	0.39				Pt	3-06	0.71	Pt	4-15	0.80
						Pt	1-10	0.60	Pt	3-10	0.77
						Pt	3-16	0.43	Pt	2-06	0.73
						Pt	6-07	0.35	Pt	3-05	0.69
						Pt	1-04	0.34	Pt	5-03	0.66
						Pt	9-15	0.31	Pt	3-21	0.65
						Ru	1-10	0.77	Pt	2-29	0.64
						Ru	1-04	0.66	Pt	4-06	0.56
						Ru	2-13	0.61	Pt	4-24	0.49
						Ru	6-04	0.56	Pt	5-13	0.48
						Ru	6-07	0.55	Pt	3-51	0.41
						Ru	3-32	0.52	Pt	9-17	0.40
						Ru	5-21	0.52	Pt	9-19	0.39
						Ru	2-06	0.40	Pt	4-05	0.33
						Ru	3-06	0.32	Ru	3-21	0.41
						Ru	9-17	0.30			

Supplementary Table 14: **Surface coverages**.

M	*	O*	OH*	C_xH_y*	CO*	CCO*	Others
Ethanol direct decomposition							
Cu	0.004	0.000	0.000	0.000	0.000	0.996	0.001
Ru	0.000	0.000	0.000	0.763	0.164	0.072	0.001
Pd	0.000	0.000	0.000	0.338	0.662	0.000	0.000
Pt	0.000	0.000	0.000	0.862	0.136	0.001	0.001
Ethanol autothermal reforming							
Cu	0.000	0.999	0.001	0.000	0.000	0.000	0.000
Ru	0.000	1.000	0.000	0.000	0.000	0.000	0.000
Pd	0.000	0.037	0.000	0.131	0.825	0.004	0.003
Pt	0.002	0.007	0.000	0.970	0.020	0.000	0.001
Ethanol steam reforming							
Cu	0.150	0.000	0.000	0.001	0.000	0.848	0.001
Ru	0.000	0.000	0.000	0.993	0.002	0.006	0.000
Pd	0.000	0.000	0.000	0.987	0.013	0.000	0.000
Pt	0.000	0.000	0.000	1.000	0.000	0.000	0.000
Ethanol aqueous phase reforming							
Cu	0.000	0.486	0.514	0.000	0.000	0.000	0.000
Ru	0.000	0.000	0.000	0.331	0.582	0.086	0.001
Pd	0.000	0.000	0.000	0.354	0.645	0.001	0.000
Pt	0.000	0.000	0.000	0.524	0.473	0.003	0.001
Ethylene glycol direct decomposition							
Cu	0.006	0.014	0.000	0.000	0.000	0.922	0.058
Ru	0.000	0.000	0.000	0.002	0.979	0.018	0.001
Pd	0.000	0.000	0.000	0.000	1.000	0.000	0.000
Pt	0.000	0.000	0.000	0.001	0.999	0.000	0.000
Ethylene glycol autothermal reforming							
Cu	0.018	0.101	0.028	0.000	0.002	0.438	0.413
Ru	0.000	0.024	0.000	0.372	0.419	0.165	0.020
Pd	0.000	0.000	0.000	0.000	1.000	0.000	0.000
Pt	0.003	0.000	0.000	0.009	0.987	0.000	0.001
Ethylene glycol steam reforming							
Cu	0.002	0.000	0.000	0.000	0.000	0.998	0.000
Ru	0.000	0.000	0.000	0.993	0.002	0.006	0.000
Pd	0.000	0.000	0.000	0.002	0.998	0.000	0.000
Pt	0.000	0.000	0.000	0.145	0.853	0.001	0.000
Ethylene glycol aqueous phase reforming							
Cu	0.001	0.000	0.000	0.000	0.000	0.996	0.003
Ru	0.000	0.000	0.000	0.000	0.997	0.003	0.000
Pd	0.000	0.000	0.000	0.000	1.000	0.000	0.000
Pt	0.000	0.000	0.000	0.000	1.000	0.000	0.000

Supplementary Table 15: **Main desorption products**. Fraction of desorbed products at $t = 10800$ s. AA stands for acetaldehyde and GA for glycolaldehyde.

M	H_2	H_2O	CO	CO_2	CH_2O	AA	GA	Others
Ethanol direct decomposition								
Cu	0.500	0.000	0.000	0.000	0.000	0.500	0.000	0.000
Ru	0.635	0.002	0.358	0.000	0.000	0.005	0.000	0.000
Pd	0.645	0.000	0.198	0.000	0.000	0.042	0.000	0.115
Pt	0.624	0.000	0.364	0.000	0.000	0.008	0.000	0.005
Ethanol autothermal reforming								
Cu	0.001	0.500	0.001	0.001	0.001	0.491	0.000	0.004
Ru	0.000	0.521	0.060	0.112	0.000	0.304	0.000	0.003
Pd	0.304	0.238	0.005	0.159	0.000	0.287	0.000	0.006
Pt	0.590	0.000	0.005	0.346	0.000	0.058	0.000	0.001
Ethanol steam reforming								
Cu	0.500	0.001	0.001	0.000	0.000	0.495	0.000	0.003
Ru	0.597	0.002	0.398	0.000	0.000	0.000	0.001	0.001
Pd	0.607	0.000	0.176	0.000	0.000	0.065	0.000	0.153
Pt	0.595	0.000	0.247	0.000	0.000	0.023	0.000	0.135
Ethanol aqueous phase reforming								
Cu	0.715	0.000	0.000	0.000	0.000	0.285	0.000	0.000
Ru	0.654	0.000	0.344	0.000	0.000	0.001	0.000	0.000
Pd	0.911	0.000	0.003	0.000	0.000	0.061	0.000	0.024
Pt	0.589	0.000	0.406	0.000	0.000	0.005	0.000	0.001
Ethylene glycol direct decomposition								
Cu	0.520	0.001	0.229	0.004	0.206	0.000	0.008	0.032
Ru	0.599	0.002	0.399	0.000	0.000	0.000	0.000	0.000
Pd	0.580	0.000	0.319	0.000	0.000	0.000	0.028	0.073
Pt	0.568	0.000	0.272	0.000	0.000	0.000	0.160	0.000
Ethylene glycol autothermal reforming								
Cu	0.400	0.143	0.178	0.026	0.154	0.002	0.028	0.069
Ru	0.497	0.103	0.378	0.019	0.000	0.000	0.001	0.002
Pd	0.430	0.141	0.254	0.033	0.004	0.000	0.137	0.001
Pt	0.582	0.011	0.103	0.271	0.000	0.000	0.033	0.000
Ethylene glycol steam reforming								
Cu	0.546	0.020	0.159	0.000	0.124	0.001	0.019	0.130
Ru	0.597	0.002	0.398	0.000	0.000	0.000	0.001	0.001
Pd	0.593	0.000	0.373	0.000	0.001	0.000	0.023	0.010
Pt	0.589	0.000	0.353	0.001	0.000	0.000	0.057	0.000
Ethylene glycol aqueous phase reforming								
Cu	0.547	0.006	0.178	0.000	0.161	0.000	0.006	0.101
Ru	0.600	0.001	0.399	0.000	0.000	0.000	0.000	0.000
Pd	0.582	0.000	0.327	0.000	0.000	0.000	0.023	0.068
Pt	0.567	0.000	0.267	0.000	0.000	0.000	0.166	0.000

Supplementary Table 16: **Predicted activation energies for glycerol decomposition on Ru(0001)**. Complete list of elementary steps for C_3 (glycerol), also containing those from C_2 (ethylene glycol, ethanol, and ethane), C_1 (methanol and methane) and water-gas shift reaction. The intermediates were obtained by DFT and the kinetic parameters E_a and E'_a for the direct and inverse reactions were obtained from linear scaling relationships. The DFT values were used for C_1 and C_2 transition states. All energies are in eV.

Tag	Reaction	Type	E_a	E'_a
r001	H_2(g)+*→H_2*	Ads	0.000	-0.682
r002	O_2(g)+*→O_2*	Ads	0.000	-2.186
r003	H_2O(g)+*→H_2O*	Ads	0.000	-0.486
r004	CH_4(g)+*→CH_4*	Ads	0.000	-0.097
r005	CH_3OH(g)+*→CH_3OH*	Ads	0.000	-0.570
r006	CH_3−CH_3(g)+*→CH_3−CH_3*	Ads	0.000	-0.196
r007	CH_3−CH_2OH(g)+*→CH_3−CH_2OH*	Ads	0.000	-0.647
r008	$HOCH_2$−CH_2OH(g)+*→$HOCH_2$−CH_2OH*	Ads	0.000	-0.813
r009	CO(g)+*→CO*	Ads	0.000	-2.007
r010	CO_2(g)+*→CO_2*	Ads	0.000	-0.507
r011	CH_2O(g)+*→CH_2O*	Ads	0.000	-1.218
r012	HCOOH(g)+*→HCOOH*	Ads	0.000	-1.613
r013	CH_2−CH_2(g)+*→CH_2−CH_2*	Ads	0.000	-1.371
r014	CH−CH(g)+*→CH−CH*	Ads	0.000	-3.161
r015	OCH−CH_3(g)+*→OCH−CH_3*	Ads	0.000	-1.064
r016	CH_2−CHOH(g)+*→CH_2−CHOH*	Ads	0.000	-1.381
r017	OC−CH_2(g)+*→OC−CH_2*	Ads	0.000	-1.929
r018	CH−COH(g)+*→CH−COH*	Ads	0.000	-3.247
r019	OCH−CH_2OH(g)+*→OCH−CH_2OH*	Ads	0.000	-1.531
r020	HOCH−CHOH(g)+*→HOCH−CHOH*	Ads	0.000	-1.405
r021	OCH−CHO(g)+*→OCH−CHO*	Ads	0.000	-2.596
r022	OC−CHOH(g)+*→OC−CHOH*	Ads	0.000	-2.739
r023	HOC−COH(g)+*→HOC−COH*	Ads	0.000	-4.052
r024	$HOCH_2$−CHOH−CH_2OH(g)+*→$HOCH_2$−CHOH−CH_2OH*	Ads	0.000	-1.067
r025	OCH−CO−CHO(g)+*→OCH−CO−CHO*	Ads	0.000	-3.512
r026	OCH−CH−CH_2(g)+*→OCH−CH−CH_2*	Ads	0.000	-2.054
r027	OCH−CHOH−CHO(g)+*→OCH−CHOH−CHO*	Ads	0.000	-1.952
r028	OCH−CO−CH_2OH(g)+*→OCH−CO−CH_2OH*	Ads	0.000	-2.717
r029	CH_2−CH−CH_2OH(g)+*→CH_2−CH−CH_2OH*	Ads	0.000	-1.802
r030	$HOCH_2$−CO−CH_2OH(g)+*→$HOCH_2$−CO−CH_2OH*	Ads	0.000	-1.620
r031	OCH−CHOH−CH_2OH(g)+*→OCH−CHOH−CH_2OH*	Ads	0.000	-1.761
r101	H_2*+*→H*+H*	H-H	0.000	0.000
r102	O_2*+*→O*+O*	O-O	0.209	3.853
r103	H_2O*+*→OH*+H*	O-H	0.577	1.150
r104	H_2O*+O*→OH*+OH*	O-H	0.304	0.000
r105	OH*+*→H*+O*	O-H	0.660	1.537
r106	CH_4*+*→CH_3*+H*	C-H	0.614	0.887
r107	CH_3*+*→CH_2*+H*	C-H	0.069	0.731
r108	CH_2*+*→CH*+H*	C-H	0.000	0.593
r109	CH*+*→C*+H*	C-H	0.694	0.750
r110	CH_3OH*+*→CH_3O*+H*	O-H	0.505	1.215
r111	CH_2OH*+*→CH_2O*+H*	O-H	0.338	0.899
r112	CHOH*+*→CHO*+H*	O-H	0.573	1.087
r113	COH*+*→CO*+H*	O-H	0.729	1.595
r114	CH_3OH*+*→CH_2OH*+H*	C-H	0.468	0.841
r115	CH_3O*+*→CH_2O*+H*	C-H	0.705	0.929
r116	CH_2OH*+*→CHOH*+H*	C-H	0.185	0.786
r117	CH_2O*+*→CHO*+H*	C-H	0.090	0.644
r118	CHOH*+*→COH*+H*	C-H	0.000	0.824
r119	CHO*+*→CO*+H*	C-H	0.093	1.270
r120	CH_3OH*+*→CH_3*+OH*	C-OH	0.315	1.049
r121	CH_3O*+*→CH_3*+O*	C-O	1.454	2.354
r122	CH_2OH*+*→CH_2*+OH*	C-OH	0.641	1.665

Tag	Reaction	Type	E_a	E'_a
r123	CH_2O*+*→CH_2*+O*	C-O	0.742	2.081
r124	CHOH*+*→CH*+OH*	C-OH	0.505	1.520
r125	CHO*+*→CH*+O*	C-O	0.835	2.214
r126	COH*+*→C*+OH*	C-OH	1.113	1.361
r127	CO*+*→C*+O*	C-O	1.919	2.176
r128	CO_2*+*→O*+CO*	C-O	0.635	1.879
r129	COOH*+*→OH*+CO*	C-OH	0.622	1.322
r134	COOH*+*→H*+CO_2*	O-H	0.826	1.079
r135	COOH*+O*→OH*+CO_2*	O-H	0.672	0.128
r136	COOH*+OH*→H_2O*+CO_2*	O-H	0.240	0.000
r201	CH_3−CH_3*+*→CH_2−CH_3*+H*	C-H	0.413	0.807
r202	CH_2−CH_3*+*→CH−CH_3*+H*	C-H	0.194	0.821
r203	CH_2−CH_3*+*→CH_2−CH_2*+H*	C-H	0.041	0.612
r204	CH−CH_3*+*→C−CH_3*+H*	C-H	0.000	0.739
r205	CH−CH_3*+*→CH−CH_2*+H*	C-H	0.132	0.557
r206	CH_2−CH_2*+*→CH−CH_2*+H*	C-H	0.304	0.784
r207	C−CH_3*+*→C−CH_2*+H*	C-H	0.281	0.505
r208	CH−CH_2*+*→C−CH_2*+H*	C-H	0.038	0.576
r209	CH−CH_2*+*→CH−CH*+H*	C-H	0.562	1.192
r210	C−CH_2*+*→C−CH*+H*	C-H	0.626	0.874
r211	CH−CH*+*→C−CH*+H*	C-H	0.704	0.861
r212	C−CH*+*→C−C*+H*	C-H	1.314	0.955
r213	CH_3−CH_2OH*+*→OCH_2−CH_3*+H*	O-H	0.433	1.212
r214	CH_3−CH_2OH*+*→HOCH−CH_3*+H*	C-H	0.620	0.901
r215	CH_3−CH_2OH*+*→CH_2−CH_2OH*+H*	C-H	0.424	0.846
r216	OCH_2−CH_3*+*→OCH−CH_3*+H*	C-H	0.515	0.728
r217	OCH_2−CH_3*+*→CH_2−CH_2O*+H*	C-H	0.642	0.674
r218	HOCH−CH_3*+*→OCH−CH_3*+H*	O-H	0.464	1.176
r219	HOCH−CH_3*+*→HOC−CH_3*+H*	C-H	0.178	0.592
r220	HOCH−CH_3*+*→CH_2−CHOH*+H*	C-H	0.363	0.974
r221	CH_2−CH_2OH*+*→CH_2−CH_2O*+H*	O-H	0.538	0.927
r222	CH_2−CH_2OH*+*→CH_2−CHOH*+H*	C-H	0.337	0.807
r223	CH_2−CH_2OH*+*→CH−CH_2OH*+H*	C-H	0.381	0.722
r224	OCH−CH_3*+*→OC−CH_3*+H*	C-H	0.000	0.662
r225	OCH−CH_3*+*→OCH−CH_2*+H*	C-H	0.026	0.636
r226	CH_2−CH_2O*+*→OCH−CH_2*+H*	C-H	0.408	1.199
r227	CH_2−CH_2O*+*→CH−CH_2O*+H*	C-H	0.524	0.726
r228	HOC−CH_3*+*→OC−CH_3*+H*	O-H	0.310	1.269
r229	HOC−CH_3*+*→HOC−CH_2*+H*	C-H	0.172	0.831
r230	CH_2−CHOH*+*→OCH−CH_2*+H*	O-H	0.588	1.298
r231	CH_2−CHOH*+*→HOC−CH_2*+H*	C-H	0.529	0.991
r232	CH_2−CHOH*+*→CH−CHOH*+H*	C-H	0.520	1.026
r233	CH−CH_2OH*+*→CH−CH_2O*+H*	O-H	0.496	0.746
r234	CH−CH_2OH*+*→CH−CHOH*+H*	C-H	0.100	0.735
r235	CH−CH_2OH*+*→C−CH_2OH*+H*	C-H	0.023	0.625
r236	OC−CH_3*+*→OC−CH_2*+H*	C-H	0.325	0.682
r237	OCH−CH_2*+*→OC−CH_2*+H*	C-H	0.096	0.506
r238	OCH−CH_2*+*→CH−CHO*+H*	C-H	0.266	0.598
r239	CH−CH_2O*+*→CH−CHO*+H*	C-H	0.000	0.920
r240	CH−CH_2O*+*→C−CH_2O*+H*	C-H	0.219	0.771
r241	HOC−CH_2*+*→OC−CH_2*+H*	O-H	0.533	1.191
r242	HOC−CH_2*+*→CH−COH*+H*	C-H	0.120	0.488
r243	CH−CHOH*+*→CH−CHO*+H*	O-H	0.351	0.887
r244	CH−CHOH*+*→CH−COH*+H*	C-H	0.612	0.936
r245	CH−CHOH*+*→C−CHOH*+H*	C-H	0.104	0.662
r246	C−CH_2OH*+*→C−CH_2O*+H*	O-H	0.663	0.863
r247	C−CH_2OH*+*→C−CHOH*+H*	C-H	0.000	0.591
r248	OC−CH_2*+*→OC−CH*+H*	C-H	0.506	1.005
r249	CH−CHO*+*→OC−CH*+H*	C-H	0.789	1.366
r250	CH−CHO*+*→C−CHO*+H*	C-H	0.412	0.513
r251	C−CH_2O*+*→C−CHO*+H*	C-H	0.192	0.661
r252	CH−COH*+*→OC−CH*+H*	O-H	0.625	1.414
r253	CH−COH*+*→C−COH*+H*	C-H	0.459	0.727

Tag	Reaction	Type	E_a	E'_a
r254	C−CHOH*+*→C−CHO*+H*	O-H	0.546	0.624
r255	C−CHOH*+*→C−COH*+H*	C-H	0.987	1.021
r256	OC−CH*+*→C−CO*+H*	C-H	1.150	1.424
r257	C−CHO*+*→C−CO*+H*	C-H	0.225	0.975
r258	C−COH*+*→C−CO*+H*	O-H	0.808	1.603
r259	$HOCH_2$−CH_2OH*+*→OCH_2−CH_2OH*+H*	O-H	0.321	1.121
r260	$HOCH_2$−CH_2OH*+*→HOCH−CH_2OH*+H*	C-H	0.522	0.920
r261	OCH_2−CH_2OH*+*→OCH_2−CH_2O*+H*	O-H	0.433	0.825
r262	OCH_2−CH_2OH*+*→OCH_2−CHOH*+H*	C-H	0.659	0.858
r263	OCH_2−CH_2OH*+*→OCH−CH_2OH*+H*	C-H	0.472	0.783
r264	HOCH−CH_2OH*+*→OCH_2−CHOH*+H*	O-H	0.506	1.107
r265	HOCH−CH_2OH*+*→OCH−CH_2OH*+H*	O-H	0.499	1.212
r266	HOCH−CH_2OH*+*→HOC−CH_2OH*+H*	C-H	0.173	0.712
r267	HOCH−CH_2OH*+*→HOCH−CHOH*+H*	C-H	0.372	0.851
r268	OCH_2−CH_2O*+*→OCH−CH_2O*+H*	C-H	0.576	0.885
r269	OCH_2−CHOH*+*→OCH−CH_2O*+H*	O-H	0.455	0.957
r270	OCH_2−CHOH*+*→HOC−CH_2O*+H*	C-H	0.647	1.172
r271	OCH_2−CHOH*+*→OCH−CHOH*+H*	C-H	0.445	1.118
r272	OCH−CH_2OH*+*→OCH−CH_2O*+H*	O-H	0.503	0.893
r273	OCH−CH_2OH*+*→OCH−CHOH*+H*	C-H	0.449	1.011
r274	OCH−CH_2OH*+*→OC−CH_2OH*+H*	C-H	0.208	0.844
r275	HOC−CH_2OH*+*→HOC−CH_2O*+H*	O-H	0.405	0.992
r276	HOC−CH_2OH*+*→OC−CH_2OH*+H*	O-H	0.403	1.213
r277	HOC−CH_2OH*+*→HOC−CHOH*+H*	C-H	0.381	0.869
r278	HOCH−CHOH*+*→OCH−CHOH*+H*	O-H	0.203	0.999
r279	HOCH−CHOH*+*→HOC−CHOH*+H*	C-H	0.169	0.715
r280	OCH−CH_2O*+*→OCH−CHO*+H*	C-H	0.530	1.340
r281	OCH−CH_2O*+*→OC−CH_2O*+H*	C-H	0.106	0.647
r282	HOC−CH_2O*+*→OC−CH_2O*+H*	O-H	0.858	1.377
r283	HOC−CH_2O*+*→OCH−COH*+H*	C-H	0.251	0.770
r284	OCH−CHOH*+*→OCH−CHO*+H*	O-H	0.464	1.103
r285	OCH−CHOH*+*→OCH−COH*+H*	C-H	0.292	0.662
r286	OCH−CHOH*+*→OC−CHOH*+H*	C-H	0.065	0.550
r287	OC−CH_2OH*+*→OC−CH_2O*+H*	O-H	0.429	0.725
r288	OC−CH_2OH*+*→OC−CHOH*+H*	C-H	0.208	0.620
r289	HOC−CHOH*+*→OCH−COH*+H*	O-H	0.404	1.023
r290	HOC−CHOH*+*→OC−CHOH*+H*	O-H	0.478	1.212
r291	HOC−CHOH*+*→HOC−COH*+H*	C-H	0.068	0.736
r292	OCH−CHO*+*→OC−CHO*+H*	C-H	0.989	1.300
r293	OC−CH_2O*+*→OC−CHO*+H*	C-H	0.001	0.581
r294	OCH−COH*+*→OC−CHO*+H*	O-H	0.456	1.035
r295	OCH−COH*+*→OC−COH*+H*	C-H	0.650	1.161
r296	OC−CHOH*+*→OC−CHO*+H*	O-H	0.527	0.991
r297	OC−CHOH*+*→OC−COH*+H*	C-H	0.888	1.284
r298	HOC−COH*+*→OC−COH*+H*	O-H	0.863	1.324
r299	OC−CHO*+*→OC−CO*+H*	C-H	0.736	0.948
r300	OC−COH*+*→OC−CO*+H*	O-H	1.013	1.295
r301	CH_3−CH_3*+*→CH_3*+CH_3*	C-C	2.083	2.034
r302	CH_2−CH_3*+*→CH_2*+CH_3*	C-C	0.866	1.086
r303	CH−CH_3*+*→CH*+CH_3*	C-C	0.851	1.037
r304	CH_2−CH_2*+*→CH_2*+CH_2*	C-C	0.958	1.268
r305	C−CH_3*+*→C*+CH_3*	C-C	1.301	0.805
r306	CH−CH_2*+*→CH*+CH_2*	C-C	0.988	1.411
r307	C−CH_2*+*→C*+CH_2*	C-C	1.455	1.397
r308	CH−CH*+*→CH*+CH*	C-C	0.877	1.265
r309	C−CH*+*→C*+CH*	C-C	1.190	1.477
r310	C−C*+*→C*+C*	C-C	1.463	2.166
r311	CH_3−CH_2OH*+*→CH_3*+CH_2OH*	C-C	2.830	2.659
r312	OCH_2−CH_3*+*→CH_2O*+CH_3*	C-C	2.159	1.769
r313	HOCH−CH_3*+*→CHOH*+CH_3*	C-C	1.108	1.257
r314	CH_2−CH_2OH*+*→CH_2*+CH_2OH*	C-C	1.198	1.267
r315	OCH−CH_3*+*→CHO*+CH_3*	C-C	0.766	0.718
r316	CH_2−CH_2O*+*→CH_2*+CH_2O*	C-C	0.932	1.173

Supplementary Table 16 – continues from previous page

Tag	Reaction	Type	E_a	E'_a
r317	$HOC-CH_3$*+*→COH*+CH_3*	C-C	0.994	1.554
r318	CH_2-CHOH*+*→CH_2*+CHOH*	C-C	1.414	1.615
r319	$CH-CH_2OH$*+*→CH*+CH_2OH*	C-C	0.821	1.142
r320	$OC-CH_3$*+*→CO*+CH_3*	C-C	0.860	1.327
r321	$OCH-CH_2$*+*→CHO*+CH_2*	C-C	1.064	1.068
r322	$CH-CH_2O$*+*→CH*+CH_2O*	C-C	0.803	1.435
r323	$HOC-CH_2$*+*→COH*+CH_2*	C-C	0.960	1.523
r324	CH−CHOH*+*→CH*+CHOH*	C-C	0.666	0.953
r325	$C-CH_2OH$*+*→C*+CH_2OH*	C-C	1.040	0.816
r326	$OC-CH_2$*+*→CO*+CH_2*	C-C	0.824	1.595
r327	CH−CHO*+*→CH*+CHO*	C-C	0.368	0.634
r328	$C-CH_2O$*+*→C*+CH_2O*	C-C	0.955	1.092
r329	CH−COH*+*→CH*+COH*	C-C	0.343	1.131
r330	C−CHOH*+*→C*+CHOH*	C-C	1.088	0.873
r331	OC−CH*+*→CO*+CH*	C-C	0.517	1.382
r332	C−CHO*+*→C*+CHO*	C-C	0.508	0.729
r333	C−COH*+*→C*+COH*	C-C	0.686	1.262
r334	C−CO*+*→C*+CO*	C-C	0.296	0.943
r335	$HOCH_2-CH_2OH$*+*→CH_2OH*+CH_2OH*	C-C	1.303	1.260
r336	OCH_2-CH_2OH*+*→CH_2O*+CH_2OH*	C-C	2.042	1.760
r337	$HOCH-CH_2OH$*+*→CHOH*+CH_2OH*	C-C	1.312	1.472
r338	OCH_2-CH_2O*+*→CH_2O*+CH_2O*	C-C	1.650	1.537
r339	OCH_2-CHOH*+*→CH_2O*+CHOH*	C-C	1.310	1.430
r340	$OCH-CH_2OH$*+*→CHO*+CH_2OH*	C-C	0.949	0.910
r341	$HOC-CH_2OH$*+*→COH*+CH_2OH*	C-C	0.854	1.300
r342	HOCH−CHOH*+*→CHOH*+CHOH*	C-C	0.891	1.173
r343	$OCH-CH_2O$*+*→CHO*+CH_2O*	C-C	0.836	0.967
r344	$HOC-CH_2O$*+*→COH*+CH_2O*	C-C	0.921	1.340
r345	OCH−CHOH*+*→CHO*+CHOH*	C-C	1.202	1.202
r346	$OC-CH_2OH$*+*→CO*+CH_2OH*	C-C	0.734	1.237
r347	HOC−CHOH*+*→COH*+CHOH*	C-C	0.741	1.301
r348	OCH−CHO*+*→CHO*+CHO*	C-C	0.929	0.805
r349	$OC-CH_2O$*+*→CO*+CH_2O*	C-C	0.698	1.466
r350	OCH−COH*+*→CHO*+COH*	C-C	0.614	1.069
r351	OC−CHOH*+*→CO*+CHOH*	C-C	0.974	1.666
r352	HOC−COH*+*→COH*+COH*	C-C	0.452	1.168
r353	OC−CHO*+*→CO*+CHO*	C-C	0.769	1.510
r354	OC−COH*+*→CO*+COH*	C-C	0.157	1.278
r355	OC−CO*+*→CO*+CO*	C-C	0.019	1.724
r356	CH_3-CH_2OH*+*→CH_2-CH_3*+OH*	C-OH	1.441	2.074
r357	OCH_2-CH_3*+*→CH_2-CH_3*+O*	C-O	1.029	1.759
r358	$HOCH-CH_3$*+*→$CH-CH_3$*+OH*	C-OH	0.472	1.450
r359	CH_2-CH_2OH*+*→CH_2-CH_2*+OH*	C-OH	0.831	1.613
r360	$OCH-CH_3$*+*→$CH-CH_3$*+O*	C-O	0.787	1.930
r361	CH_2-CH_2O*+*→CH_2-CH_2*+O*	C-O	0.698	1.967
r362	$HOC-CH_3$*+*→$C-CH_3$*+OH*	C-OH	0.262	1.565
r363	CH_2-CHOH*+*→$CH-CH_2$*+OH*	C-OH	1.126	1.918
r364	$CH-CH_2OH$*+*→$CH-CH_2$*+OH*	C-OH	0.764	1.685
r365	$OC-CH_3$*+*→$C-CH_3$*+O*	C-O	0.767	1.987
r366	$OCH-CH_2$*+*→$CH-CH_2$*+O*	C-O	1.268	2.226
r367	$CH-CH_2O$*+*→$CH-CH_2$*+O*	C-O	0.486	2.033
r368	$HOC-CH_2$*+*→$C-CH_2$*+OH*	C-OH	0.712	1.580
r369	CH−CHOH*+*→CH−CH*+OH*	C-OH	0.697	1.612
r370	$C-CH_2OH$*+*→$C-CH_2$*+OH*	C-OH	0.603	1.459
r371	$OC-CH_2$*+*→$C-CH_2$*+O*	C-O	1.162	2.249
r372	CH−CHO*+*→CH−CH*+O*	C-O	0.966	2.223
r373	$C-CH_2O$*+*→$C-CH_2$*+O*	C-O	0.436	1.969
r374	CH−COH*+*→C−CH*+OH*	C-OH	1.239	1.987
r375	C−CHOH*+*→C−CH*+OH*	C-OH	0.744	1.258
r376	OC−CH*+*→C−CH*+O*	C-O	1.608	2.444
r377	C−CHO*+*→C−CH*+O*	C-O	0.740	2.052
r378	C−COH*+*→C−C*+OH*	C-OH	1.860	1.981
r379	C−CO*+*→C−C*+O*	C-O	2.094	2.297

Supplementary Table 16 – continues from previous page

Tag	Reaction	Type	E_a	E'_a
r380	$HOCH_2-CH_2OH$*+*→CH_2-CH_2OH*+OH*	C-OH	1.091	2.003
r381	OCH_2-CH_2OH*+*→CH_2-CH_2O*+OH*	C-OH	1.482	1.983
r382	OCH_2-CH_2OH*+*→CH_2-CH_2OH*+O*	C-O	1.364	2.352
r383	$HOCH-CH_2OH$*+*→CH_2-CHOH*+OH*	C-OH	0.316	1.299
r384	$HOCH-CH_2OH$*+*→$CH-CH_2OH$*+OH*	C-OH	0.918	1.772
r385	OCH_2-CH_2O*+*→CH_2-CH_2O*+O*	C-O	1.001	1.986
r386	OCH_2-CHOH*+*→$CH-CH_2O$*+OH*	C-OH	0.481	0.984
r387	OCH_2-CHOH*+*→CH_2-CHOH*+O*	C-O	0.660	1.918
r388	$OCH-CH_2OH$*+*→$OCH-CH_2$*+OH*	C-OH	0.671	1.651
r389	$OCH-CH_2OH$*+*→$CH-CH_2OH$*+O*	C-O	0.811	1.829
r390	$HOC-CH_2OH$*+*→$HOC-CH_2$*+OH*	C-OH	0.836	1.743
r391	$HOC-CH_2OH$*+*→$C-CH_2OH$*+OH*	C-OH	0.608	1.526
r392	HOCH−CHOH*+*→CH−CHOH*+OH*	C-OH	0.653	1.663
r393	$OCH-CH_2O$*+*→$OCH-CH_2$*+O*	C-O	0.549	2.016
r394	$OCH-CH_2O$*+*→$CH-CH_2O$*+O*	C-O	0.939	1.817
r395	$HOC-CH_2O$*+*→$C-CH_2O$*+OH*	C-OH	0.944	1.474
r396	$HOC-CH_2O$*+*→$HOC-CH_2$*+O*	C-O	1.219	2.415
r397	OCH−CHOH*+*→CH−CHO*+OH*	C-OH	0.747	1.498
r398	OCH−CHOH*+*→CH−CHOH*+O*	C-O	1.445	2.536
r399	$OC-CH_2OH$*+*→$OC-CH_2$*+OH*	C-OH	0.967	1.722
r400	$OC-CH_2OH$*+*→$C-CH_2OH$*+O*	C-O	1.092	2.076
r401	HOC−CHOH*+*→CH−COH*+OH*	C-OH	0.673	1.461
r402	HOC−CHOH*+*→C−CHOH*+OH*	C-OH	0.692	1.713
r403	OCH−CHO*+*→CH−CHO*+O*	C-O	1.245	2.234
r404	$OC-CH_2O$*+*→$OC-CH_2$*+O*	C-O	0.800	2.135
r405	$OC-CH_2O$*+*→$C-CH_2O$*+O*	C-O	1.350	2.238
r406	OCH−COH*+*→C−CHO*+OH*	C-OH	0.712	1.193
r407	OCH−COH*+*→CH−COH*+O*	C-O	1.058	2.102
r408	OC−CHOH*+*→OC−CH*+OH*	C-OH	0.742	1.584
r409	OC−CHOH*+*→C−CHOH*+O*	C-O	1.334	2.498
r410	HOC−COH*+*→C−COH*+OH*	C-OH	1.098	1.485
r411	OC−CHO*+*→OC−CH*+O*	C-O	1.218	2.472
r412	OC−CHO*+*→C−CHO*+O*	C-O	1.630	2.407
r413	OC−COH*+*→C−CO*+OH*	C-OH	1.388	2.109
r414	OC−COH*+*→C−COH*+O*	C-O	1.896	2.698
r415	OC−CO*+*→C−CO*+O*	C-O	1.221	2.536
r500	$HOCH_2-CHOH-CH_2OH$*+*→$OCH_2-CHOH-CH_2OH$*+H*	O-H	0.406	1.098
r501	$HOCH_2-CHOH-CH_2OH$*+*→$HOCH_2-CHO-CH_2OH$*+H*	O-H	0.406	1.117
r502	$HOCH_2-CHOH-CH_2OH$*+*→$HOCH-CHOH-CH_2OH$*+H*	C-H	0.359	0.733
r503	$HOCH_2-CHOH-CH_2OH$*+*→$HOCH_2-COH-CH_2OH$*+H*	C-H	0.215	0.749
r504	$HOCH_2-CHOH-CH_2OH$*+*→$CH_2-CHOH-CH_2OH$*+OH*	C-OH	0.895	1.662
r505	$HOCH_2-CHOH-CH_2OH$*+*→$HOCH_2-CH-CH_2OH$*+OH*	C-OH	0.708	1.651
r506	$HOCH_2-CHOH-CH_2OH$*+*→$HOCH-CH_2OH$*+CH_2OH*	C-C	1.217	1.144
r507	$HOCH_2-COH-CH_2OH$*+*→$OCH_2-COH-CH_2OH$*+H*	O-H	0.482	1.086
r508	$HOCH_2-COH-CH_2OH$*+*→$HOCH_2-CO-CH_2OH$*+H*	O-H	0.482	1.244
r509	$HOCH_2-COH-CH_2OH$*+*→$HOCH-COH-CH_2OH$*+H*	C-H	0.298	0.797
r510	$HOCH_2-COH-CH_2OH$*+*→$CH_2-COH-CH_2OH$*+OH*	C-OH	0.651	1.617
r511	$HOCH_2-COH-CH_2OH$*+*→$HOCH_2-C-CH_2OH$*+OH*	C-OH	1.143	1.646
r512	$HOCH_2-COH-CH_2OH$*+*→$HOC-CH_2OH$*+CH_2OH*	C-C	1.237	1.169
r513	$HOCH_2-CHO-CH_2OH$*+*→$OCH_2-CHO-CH_2OH$*+H*	O-H	0.508	1.079
r514	$HOCH_2-CHO-CH_2OH$*+*→$HOCH-CHO-CH_2OH$*+H*	C-H	0.556	0.789
r515	$HOCH_2-CHO-CH_2OH$*+*→$HOCH_2-CO-CH_2OH$*+H*	C-H	0.238	0.823
r516	$HOCH_2-CHO-CH_2OH$*+*→$HOCH_2-CH-CH_2OH$*+O*	C-O	0.896	2.005
r517	$HOCH_2-CHO-CH_2OH$*+*→$CH_2-CHO-CH_2OH$*+OH*	C-OH	1.092	1.632
r518	$HOCH_2-CHO-CH_2OH$*+*→$OCH-CH_2OH$*+CH_2OH*	C-C	1.248	1.176
r519	$HOCH_2-CH-CH_2OH$*+*→$OCH_2-CH-CH_2OH$*+H*	O-H	0.541	1.108
r520	$HOCH_2-CH-CH_2OH$*+*→$HOCH-CH-CH_2OH$*+H*	C-H	0.440	0.826
r521	$HOCH_2-CH-CH_2OH$*+*→$HOCH_2-C-CH_2OH$*+H*	C-H	0.704	0.797
r522	$HOCH_2-CH-CH_2OH$*+*→$CH_2-CH-CH_2OH$*+OH*	C-OH	0.814	1.603
r523	$HOCH_2-CH-CH_2OH$*+*→$CH-CH_2OH$*+CH_2OH*	C-C	1.345	1.183
r524	$HOCH_2-CO-CH_2OH$*+*→$OCH_2-CO-CH_2OH$*+H*	O-H	0.591	0.859
r525	$HOCH_2-CO-CH_2OH$*+*→$HOCH-CO-CH_2OH$*+H*	C-H	0.324	0.876
r526	$HOCH_2-CO-CH_2OH$*+*→$HOCH_2-C-CH_2OH$*+O*	C-O	1.413	2.030

Supplementary Table 16 – continues from previous page

Tag	Reaction	Type	E_a	E'_a
r527	HOCH$_2$−CO−CH$_2$OH*+*→CH$_2$−CO−CH$_2$OH*+OH*	C-OH	0.789	1.580
r528	HOCH$_2$−CO−CH$_2$OH*+*→OC−CH$_2$OH*+CH$_2$OH*	C-C	1.226	1.205
r529	HOCH$_2$−C−CH$_2$OH*+*→OCH$_2$−C−CH$_2$OH*+H*	O-H	0.554	0.945
r530	HOCH$_2$−C−CH$_2$OH*+*→HOCH−C−CH$_2$OH*+H*	C-H	0.247	0.857
r531	HOCH$_2$−C−CH$_2$OH*+*→CH$_2$−C−CH$_2$OH*+OH*	C-OH	0.557	1.582
r532	HOCH$_2$−C−CH$_2$OH*+*→C−CH$_2$OH*+CH$_2$OH*	C-C	0.864	1.210
r533	HOCH−CHOH−CH$_2$OH*+*→OCH−CHOH−CH$_2$OH*+H*	O-H	0.459	1.217
r534	HOCH−CHOH−CH$_2$OH*+*→HOCH−CHO−CH$_2$OH*+H*	O-H	0.459	1.029
r535	HOCH−CHOH−CH$_2$OH*+*→OCH$_2$−CHOH−CHOH*+H*	O-H	0.459	1.075
r536	HOCH−CHOH−CH$_2$OH*+*→HOC−CHOH−CH$_2$OH*+H*	C-H	0.301	0.780
r537	HOCH−CHOH−CH$_2$OH*+*→HOCH−COH−CH$_2$OH*+H*	C-H	0.138	0.797
r538	HOCH−CHOH−CH$_2$OH*+*→HOCH−CHOH−CHOH*+H*	C-H	0.341	0.775
r539	HOCH−CHOH−CH$_2$OH*+*→CH−CHOH−CH$_2$OH*+OH*	C-OH	0.776	1.634
r540	HOCH−CHOH−CH$_2$OH*+*→HOCH−CH−CH$_2$OH*+OH*	C-OH	0.672	1.627
r541	HOCH−CHOH−CH$_2$OH*+*→CH$_2$−CHOH−CHOH*+OH*	C-OH	0.777	1.634
r542	HOCH−CHOH−CH$_2$OH*+*→HOCH−CHOH*+CH$_2$OH*	C-C	1.134	1.166
r543	HOCH−CHOH−CH$_2$OH*+*→HOCH−CH$_2$OH*+CHOH*	C-C	1.017	1.171
r544	HOCH−CHOH−CHOH*+*→OCH−CHOH−CHOH*+H*	O-H	0.521	1.001
r545	HOCH−CHOH−CHOH*+*→HOCH−CHO−CHOH*+H*	O-H	0.521	1.010
r546	HOCH−CHOH−CHOH*+*→HOC−CHOH−CHOH*+H*	C-H	0.456	0.810
r547	HOCH−CHOH−CHOH*+*→HOCH−COH−CHOH*+H*	C-H	0.234	0.834
r548	HOCH−CHOH−CHOH*+*→CH−CHOH−CHOH*+OH*	C-OH	1.109	1.628
r549	HOCH−CHOH−CHOH*+*→HOCH−CH−CHOH*+OH*	C-OH	0.754	1.607
r550	HOCH−CHOH−CHOH*+*→HOCH−CHOH*+CHOH*	C-C	0.994	1.193
r551	HOCH−COH−CH$_2$OH*+*→OCH−COH−CH$_2$OH*+H*	O-H	0.554	1.339
r552	HOCH−COH−CH$_2$OH*+*→HOCH−CO−CH$_2$OH*+H*	O-H	0.554	1.369
r553	HOCH−COH−CH$_2$OH*+*→OCH$_2$−COH−CHOH*+H*	O-H	0.554	1.400
r554	HOCH−COH−CH$_2$OH*+*→HOC−COH−CH$_2$OH*+H*	C-H	0.517	0.827
r555	HOCH−COH−CH$_2$OH*+*→HOCH−COH−CHOH*+H*	C-H	0.460	0.834
r556	HOCH−COH−CH$_2$OH*+*→CH−COH−CH$_2$OH*+OH*	C-OH	0.682	1.589
r557	HOCH−COH−CH$_2$OH*+*→HOCH−C−CH$_2$OH*+OH*	C-OH	0.994	1.608
r558	HOCH−COH−CH$_2$OH*+*→CH$_2$−COH−CHOH*+OH*	C-OH	0.676	1.589
r559	HOCH−COH−CH$_2$OH*+*→HOC−CHOH*+CH$_2$OH*	C-C	1.271	1.191
r560	HOCH−COH−CH$_2$OH*+*→HOC−CH$_2$OH*+CHOH*	C-C	1.163	1.196
r561	HOCH−COH−CHOH*+*→OCH−COH−CHOH*+H*	O-H	0.607	1.325
r562	HOCH−COH−CHOH*+*→HOCH−CO−CHOH*+H*	O-H	0.607	1.560
r563	HOCH−COH−CHOH*+*→HOC−COH−CHOH*+H*	C-H	0.492	0.870
r564	HOCH−COH−CHOH*+*→CH−COH−CHOH*+OH*	C-OH	0.910	1.581
r565	HOCH−COH−CHOH*+*→HOCH−C−CHOH*+OH*	C-OH	0.697	1.568
r566	HOCH−COH−CHOH*+*→HOC−CHOH*+CHOH*	C-C	1.071	1.218
r567	HOCH−CHO−CH$_2$OH*+*→OCH−CHO−CH$_2$OH*+H*	O-H	0.541	1.152
r568	HOCH−CHO−CH$_2$OH*+*→OCH$_2$−CHO−CHOH*+H*	O-H	0.541	0.818
r569	HOCH−CHO−CH$_2$OH*+*→HOC−CHO−CH$_2$OH*+H*	C-H	0.378	0.833
r570	HOCH−CHO−CH$_2$OH*+*→HOCH−CO−CH$_2$OH*+H*	C-H	0.000	0.905
r571	HOCH−CHO−CH$_2$OH*+*→HOCH−CHO−CHOH*+H*	C-H	0.470	0.823
r572	HOCH−CHO−CH$_2$OH*+*→HOCH−CH−CH$_2$OH*+O*	C-O	0.846	2.108
r573	HOCH−CHO−CH$_2$OH*+*→CH−CHO−CH$_2$OH*+OH*	C-OH	1.198	1.625
r574	HOCH−CHO−CH$_2$OH*+*→CH$_2$−CHO−CHOH*+OH*	C-OH	0.911	1.608
r575	HOCH−CHO−CH$_2$OH*+*→OCH−CHOH*+CH$_2$OH*	C-C	0.944	1.202
r576	HOCH−CHO−CH$_2$OH*+*→OCH−CH$_2$OH*+CHOH*	C-C	0.906	1.204
r577	HOCH−CHO−CHOH*+*→OCH−CHO−CHOH*+H*	O-H	0.591	1.088
r578	HOCH−CHO−CHOH*+*→HOC−CHO−CHOH*+H*	C-H	0.356	0.873
r579	HOCH−CHO−CHOH*+*→HOCH−CO−CHOH*+H*	C-H	0.000	1.064
r580	HOCH−CHO−CHOH*+*→HOCH−CH−CHOH*+O*	C-O	0.957	2.198
r581	HOCH−CHO−CHOH*+*→CH−CHO−CHOH*+OH*	C-OH	1.035	1.595
r582	HOCH−CHO−CHOH*+*→OCH−CHOH*+CHOH*	C-C	0.723	1.229
r583	HOCH−CH−CH$_2$OH*+*→OCH−CH−CH$_2$OH*+H*	O-H	0.596	1.365
r584	HOCH−CH−CH$_2$OH*+*→OCH$_2$−CH−CHOH*+H*	O-H	0.596	1.066
r585	HOCH−CH−CH$_2$OH*+*→HOC−CH−CH$_2$OH*+H*	C-H	0.433	0.868
r586	HOCH−CH−CH$_2$OH*+*→HOCH−C−CH$_2$OH*+H*	C-H	0.539	0.857
r587	HOCH−CH−CH$_2$OH*+*→HOCH−CH−CHOH*+H*	C-H	0.526	0.858
r588	HOCH−CH−CH$_2$OH*+*→CH−CH−CH$_2$OH*+OH*	C-OH	0.871	1.583
r589	HOCH−CH−CH$_2$OH*+*→CH$_2$−CH−CHOH*+OH*	C-OH	1.902	1.643

Supplementary Table 16 – continues from previous page

Tag	Reaction	Type	E_a	E'_a
r590	HOCH−CH−CH$_2$OH*+*→CH−CHOH*+CH$_2$OH*	C-C	1.124	1.211
r591	HOCH−CH−CH$_2$OH*+*→CH−CH$_2$OH*+CHOH*	C-C	1.157	1.210
r592	HOCH−CH−CHOH*+*→OCH−CH−CHOH*+H*	O-H	0.643	1.631
r593	HOCH−CH−CHOH*+*→HOC−CH−CHOH*+H*	C-H	0.585	0.887
r594	HOCH−CH−CHOH*+*→HOCH−C−CHOH*+H*	C-H	0.300	0.918
r595	HOCH−CH−CHOH*+*→CH−CH−CHOH*+OH*	C-OH	1.013	1.572
r596	HOCH−CH−CHOH*+*→CH−CHOH*+CHOH*	C-C	0.882	1.239
r597	HOCH−CO−CH$_2$OH*+*→OCH−CO−CH$_2$OH*+H*	O-H	0.670	1.380
r598	HOCH−CO−CH$_2$OH*+*→OCH$_2$−CO−CHOH*+H*	O-H	0.670	1.284
r599	HOCH−CO−CH$_2$OH*+*→HOC−CO−CH$_2$OH*+H*	C-H	0.616	0.905
r600	HOCH−CO−CH$_2$OH*+*→HOCH−CO−CHOH*+H*	C-H	0.415	0.926
r601	HOCH−CO−CH$_2$OH*+*→HOCH−C−CH$_2$OH*+O*	C-O	1.519	2.194
r602	HOCH−CO−CH$_2$OH*+*→CH−CO−CH$_2$OH*+OH*	C-OH	1.095	1.566
r603	HOCH−CO−CH$_2$OH*+*→CH$_2$−CO−CHOH*+OH*	C-OH	0.593	1.537
r604	HOCH−CO−CH$_2$OH*+*→OC−CHOH*+CH$_2$OH*	C-C	1.385	1.224
r605	HOCH−CO−CH$_2$OH*+*→OC−CH$_2$OH*+CHOH*	C-C	1.205	1.232
r606	HOCH−CO−CHOH*+*→OCH−CO−CHOH*+H*	O-H	0.744	1.124
r607	HOCH−CO−CHOH*+*→HOC−CO−CHOH*+H*	C-H	0.754	0.945
r608	HOCH−CO−CHOH*+*→HOCH−C−CHOH*+O*	C-O	1.569	2.363
r609	HOCH−CO−CHOH*+*→CH−CO−CHOH*+OH*	C-OH	1.037	1.533
r610	HOCH−CO−CHOH*+*→OC−CHOH*+CHOH*	C-C	1.323	1.251
r611	HOCH−C−CH$_2$OH*+*→OCH−C−CH$_2$OH*+H*	O-H	0.641	1.311
r612	HOCH−C−CH$_2$OH*+*→OCH$_2$−C−CHOH*+H*	O-H	0.641	1.221
r613	HOCH−C−CH$_2$OH*+*→HOC−C−CH$_2$OH*+H*	C-H	0.297	0.917
r614	HOCH−C−CH$_2$OH*+*→HOCH−C−CHOH*+H*	C-H	0.286	0.918
r615	HOCH−C−CH$_2$OH*+*→CH−C−CH$_2$OH*+OH*	C-OH	0.633	1.551
r616	HOCH−C−CH$_2$OH*+*→CH$_2$−C−CHOH*+OH*	C-OH	0.680	1.554
r617	HOCH−C−CH$_2$OH*+*→C−CHOH*+CH$_2$OH*	C-C	0.909	1.237
r618	HOCH−C−CH$_2$OH*+*→C−CH$_2$OH*+CHOH*	C-C	0.900	1.237
r619	HOCH−C−CHOH*+*→OCH−C−CHOH*+H*	O-H	0.732	1.176
r620	HOCH−C−CHOH*+*→HOC−C−CHOH*+H*	C-H	0.591	0.953
r621	HOCH−C−CHOH*+*→CH−C−CHOH*+OH*	C-OH	0.911	1.530
r622	HOCH−C−CHOH*+*→C−CHOH*+CHOH*	C-C	0.967	1.264
r623	OCH$_2$−CHOH−CH$_2$OH*+*→OCH$_2$−CHO−CH$_2$OH*+H*	O-H	0.505	1.095
r624	OCH$_2$−CHOH−CH$_2$OH*+*→OCH$_2$−CHOH−CH$_2$O*+H*	O-H	0.505	1.098
r625	OCH$_2$−CHOH−CH$_2$OH*+*→OCH−CHOH−CH$_2$OH*+H*	C-H	0.367	0.807
r626	OCH$_2$−CHOH−CH$_2$OH*+*→OCH$_2$−COH−CH$_2$OH*+H*	C-H	0.362	0.807
r627	OCH$_2$−CHOH−CH$_2$OH*+*→OCH$_2$−CHOH−CHOH*+H*	C-H	0.495	0.793
r628	OCH$_2$−CHOH−CH$_2$OH*+*→CH$_2$−CHOH−CH$_2$OH*+O*	C-O	1.006	1.958
r629	OCH$_2$−CHOH−CH$_2$OH*+*→OCH$_2$−CH−CH$_2$OH*+OH*	C-OH	0.798	1.616
r630	OCH$_2$−CHOH−CH$_2$OH*+*→CH$_2$−CHOH−CH$_2$O*+OH*	C-OH	0.992	1.628
r631	OCH$_2$−CHOH−CH$_2$OH*+*→OCH$_2$−CHOH*+CH$_2$OH*	C-C	1.336	1.171
r632	OCH$_2$−CHOH−CH$_2$OH*+*→HOCH−CH$_2$OH*+CH$_2$O*	C-C	1.374	1.170
r633	OCH$_2$−CHOH−CHOH*+*→OCH$_2$−CHO−CHOH*+H*	O-H	0.548	0.778
r634	OCH$_2$−CHOH−CHOH*+*→OCH−CHOH−CH$_2$O*+H*	O-H	0.548	1.132
r635	OCH$_2$−CHOH−CHOH*+*→OCH−CHOH−CHOH*+H*	C-H	0.525	0.822
r636	OCH$_2$−CHOH−CHOH*+*→OCH$_2$−COH−CHOH*+H*	C-H	0.000	0.890
r637	OCH$_2$−CHOH−CHOH*+*→HOC−CHOH−CH$_2$O*+H*	C-H	0.316	0.844
r638	OCH$_2$−CHOH−CHOH*+*→CH$_2$−CHOH−CHOH*+O*	C-O	0.965	2.082
r639	OCH$_2$−CHOH−CHOH*+*→OCH$_2$−CH−CHOH*+OH*	C-OH	0.789	1.598
r640	OCH$_2$−CHOH−CHOH*+*→CH−CHOH−CH$_2$O*+OH*	C-OH	0.677	1.592
r641	OCH$_2$−CHOH−CHOH*+*→OCH$_2$−CHOH*+CHOH*	C-C	1.059	1.199
r642	OCH$_2$−CHOH−CHOH*+*→HOCH−CHOH*+CH$_2$O*	C-C	1.214	1.191
r643	OCH$_2$−CHOH−CH$_2$O*+*→OCH$_2$−CHO−CH$_2$O*+H*	O-H	0.590	1.086
r644	OCH$_2$−CHOH−CH$_2$O*+*→OCH−CHOH−CH$_2$O*+H*	C-H	0.561	0.850
r645	OCH$_2$−CHOH−CH$_2$O*+*→OCH$_2$−COH−CH$_2$O*+H*	C-H	0.473	0.859
r646	OCH$_2$−CHOH−CH$_2$O*+*→CH$_2$−CHOH−CH$_2$O*+O*	C-O	1.189	2.108
r647	OCH$_2$−CHOH−CH$_2$O*+*→OCH$_2$−CH−CH$_2$O*+OH*	C-OH	1.054	1.596
r648	OCH$_2$−CHOH−CH$_2$O*+*→OCH$_2$−CHOH*+CH$_2$O*	C-C	1.393	1.197
r649	OCH$_2$−COH−CH$_2$OH*+*→OCH$_2$−CO−CH$_2$OH*+H*	O-H	0.569	0.994
r650	OCH$_2$−COH−CH$_2$OH*+*→OCH$_2$−COH−CH$_2$O*+H*	O-H	0.569	1.103
r651	OCH$_2$−COH−CH$_2$OH*+*→OCH−COH−CH$_2$OH*+H*	C-H	0.192	0.874
r652	OCH$_2$−COH−CH$_2$OH*+*→OCH$_2$−COH−CHOH*+H*	C-H	0.137	0.879

Supplementary Table 16 – continues from previous page

Tag	Reaction	Type	E_a	E'_a
r653	$OCH_2-COH-CH_2OH$*+*→$CH_2-COH-CH_2OH$*+O*	C-O	0.916	2.154
r654	$OCH_2-COH-CH_2OH$*+*→OCH_2-C-CH_2OH*+OH*	C-OH	1.332	1.621
r655	$OCH_2-COH-CH_2OH$*+*→$CH_2-COH-CH_2O$*+OH*	C-OH	0.819	1.591
r656	$OCH_2-COH-CH_2OH$*+*→$HOC-CH_2O$*+CH_2OH*	C-C	1.280	1.195
r657	$OCH_2-COH-CH_2OH$*+*→$HOC-CH_2OH$*+CH_2O*	C-C	1.305	1.194
r658	$OCH_2-COH-CHOH$*+*→$OCH_2-CO-CHOH$*+H*	O-H	0.675	1.257
r659	$OCH_2-COH-CHOH$*+*→$OCH-COH-CH_2O$*+H*	O-H	0.675	1.150
r660	$OCH_2-COH-CHOH$*+*→$OCH-COH-CHOH$*+H*	C-H	0.659	0.903
r661	$OCH_2-COH-CHOH$*+*→$HOC-COH-CH_2O$*+H*	C-H	0.579	0.912
r662	$OCH_2-COH-CHOH$*+*→$CH_2-COH-CHOH$*+O*	C-O	1.332	2.274
r663	$OCH_2-COH-CHOH$*+*→$OCH_2-C-CHOH$*+OH*	C-OH	1.225	1.572
r664	$OCH_2-COH-CHOH$*+*→$CH-COH-CH_2O$*+OH*	C-OH	1.256	1.574
r665	$OCH_2-COH-CHOH$*+*→$HOC-CH_2O$*+CHOH*	C-C	1.448	1.222
r666	$OCH_2-COH-CHOH$*+*→$HOC-CHOH$*+CH_2O*	C-C	1.582	1.216
r667	$OCH_2-COH-CH_2O$*+*→$OCH_2-CO-CH_2O$*+H*	O-H	0.645	1.009
r668	$OCH_2-COH-CH_2O$*+*→$OCH-COH-CH_2O$*+H*	C-H	0.243	0.926
r669	$OCH_2-COH-CH_2O$*+*→$CH_2-COH-CH_2O$*+O*	C-O	1.150	2.264
r670	$OCH_2-COH-CH_2O$*+*→OCH_2-C-CH_2O*+OH*	C-OH	1.492	1.599
r671	$OCH_2-COH-CH_2O$*+*→$HOC-CH_2O$*+CH_2O*	C-C	1.279	1.220
r672	$OCH_2-CHO-CH_2OH$*+*→$OCH_2-CHO-CH_2O$*+H*	O-H	0.589	1.089
r673	$OCH_2-CHO-CH_2OH$*+*→$OCH-CHO-CH_2OH$*+H*	C-H	0.576	0.848
r674	$OCH_2-CHO-CH_2OH$*+*→$OCH_2-CO-CH_2OH$*+H*	C-H	0.568	0.849
r675	$OCH_2-CHO-CH_2OH$*+*→$OCH_2-CHO-CHOH$*+H*	C-H	0.877	0.816
r676	$OCH_2-CHO-CH_2OH$*+*→$CH_2-CHO-CH_2OH$*+O*	C-O	1.242	2.088
r677	$OCH_2-CHO-CH_2OH$*+*→$OCH_2-CH-CH_2OH$*+O*	C-O	1.052	2.157
r678	$OCH_2-CHO-CH_2OH$*+*→$CH_2-CHO-CH_2O$*+OH*	C-OH	0.818	1.583
r679	$OCH_2-CHO-CH_2OH$*+*→$OCH-CH_2O$*+CH_2OH*	C-C	1.446	1.194
r680	$OCH_2-CHO-CH_2OH$*+*→$OCH-CH_2OH$*+CH_2O*	C-C	1.284	1.202
r681	$OCH_2-CHO-CHOH$*+*→$OCH-CHO-CH_2O$*+H*	O-H	0.581	1.641
r682	$OCH_2-CHO-CHOH$*+*→$OCH-CHO-CHOH$*+H*	C-H	0.299	0.871
r683	$OCH_2-CHO-CHOH$*+*→$OCH_2-CO-CHOH$*+H*	C-H	0.000	1.241
r684	$OCH_2-CHO-CHOH$*+*→$HOC-CHO-CH_2O$*+H*	C-H	0.104	0.892
r685	$OCH_2-CHO-CHOH$*+*→$CH_2-CHO-CHOH$*+O*	C-O	0.896	2.192
r686	$OCH_2-CHO-CHOH$*+*→$OCH_2-CH-CHOH$*+O*	C-O	0.779	2.235
r687	$OCH_2-CHO-CHOH$*+*→$CH-CHO-CH_2O$*+OH*	C-OH	0.627	1.575
r688	$OCH_2-CHO-CHOH$*+*→$OCH-CH_2O$*+CHOH*	C-C	0.811	1.221
r689	$OCH_2-CHO-CHOH$*+*→$OCH-CHOH$*+CH_2O*	C-C	0.685	1.227
r690	$OCH_2-CHO-CH_2O$*+*→$OCH-CHO-CH_2O$*+H*	C-H	0.419	0.918
r691	$OCH_2-CHO-CH_2O$*+*→$OCH_2-CO-CH_2O$*+H*	C-H	0.641	0.895
r692	$OCH_2-CHO-CH_2O$*+*→$CH_2-CHO-CH_2O$*+O*	C-O	1.159	2.301
r693	$OCH_2-CHO-CH_2O$*+*→$OCH_2-CH-CH_2O$*+O*	C-O	1.320	2.242
r694	$OCH_2-CHO-CH_2O$*+*→$OCH-CH_2O$*+CH_2O*	C-C	1.410	1.219
r695	$OCH_2-CH-CH_2OH$*+*→$OCH_2-CH-CH_2O$*+H*	O-H	0.622	0.939
r696	$OCH_2-CH-CH_2OH$*+*→$OCH-CH-CH_2OH$*+H*	C-H	0.314	0.900
r697	$OCH_2-CH-CH_2OH$*+*→OCH_2-C-CH_2OH*+H*	C-H	0.919	0.835
r698	$OCH_2-CH-CH_2OH$*+*→$OCH_2-CH-CHOH$*+H*	C-H	0.583	0.872
r699	$OCH_2-CH-CH_2OH$*+*→$CH_2-CH-CH_2OH$*+O*	C-O	1.119	2.216
r700	$OCH_2-CH-CH_2OH$*+*→$CH_2-CH-CH_2O$*+OH*	C-OH	2.073	1.643
r701	$OCH_2-CH-CH_2OH$*+*→$CH-CH_2O$*+CH_2OH*	C-C	1.674	1.194
r702	$OCH_2-CH-CH_2OH$*+*→$CH-CH_2OH$*+CH_2O*	C-C	1.377	1.208
r703	$OCH_2-CH-CHOH$*+*→$OCH-CH-CH_2O$*+H*	O-H	0.663	1.507
r704	$OCH_2-CH-CHOH$*+*→$OCH-CH-CHOH$*+H*	C-H	0.105	0.954
r705	$OCH_2-CH-CHOH$*+*→$OCH_2-C-CHOH$*+H*	C-H	0.486	0.913
r706	$OCH_2-CH-CHOH$*+*→$HOC-CH-CH_2O$*+H*	C-H	0.147	0.950
r707	$OCH_2-CH-CHOH$*+*→$CH_2-CH-CHOH$*+O*	C-O	1.892	2.039
r708	$OCH_2-CH-CHOH$*+*→$CH-CH-CH_2O$*+OH*	C-OH	0.643	1.542
r709	$OCH_2-CH-CHOH$*+*→$CH-CH_2O$*+CHOH*	C-C	1.388	1.221
r710	$OCH_2-CH-CHOH$*+*→$CH-CHOH$*+CH_2O*	C-C	1.059	1.237
r711	$OCH_2-CH-CH_2O$*+*→$OCH-CH-CH_2O$*+H*	C-H	0.138	0.954
r712	$OCH_2-CH-CH_2O$*+*→OCH_2-C-CH_2O*+H*	C-H	0.918	0.870
r713	$OCH_2-CH-CH_2O$*+*→$CH_2-CH-CH_2O$*+O*	C-O	1.913	2.042
r714	$OCH_2-CH-CH_2O$*+*→$CH-CH_2O$*+CH_2O*	C-C	1.455	1.219
r715	$OCH_2-CO-CH_2OH$*+*→$OCH_2-CO-CH_2O$*+H*	O-H	0.630	1.102

Supplementary Table 16 – continues from previous page

Tag	Reaction	Type	E_a	E'_a
r716	OCH$_2$−CO−CH$_2$OH*+*→OCH−CO−CH$_2$OH*+H*	C-H	0.000	0.994
r717	OCH$_2$−CO−CH$_2$OH*+*→OCH$_2$−CO−CHOH*+H*	C-H	0.038	0.936
r718	OCH$_2$−CO−CH$_2$OH*+*→CH$_2$−CO−CH$_2$OH*+O*	C-O	0.912	2.312
r719	OCH$_2$−CO−CH$_2$OH*+*→OCH$_2$−C−CH$_2$OH*+O*	C-O	1.395	2.135
r720	OCH$_2$−CO−CH$_2$OH*+*→CH$_2$−CO−CH$_2$O*+OH*	C-OH	0.518	1.549
r721	OCH$_2$−CO−CH$_2$OH*+*→OC−CH$_2$O*+CH$_2$OH*	C-C	1.211	1.219
r722	OCH$_2$−CO−CH$_2$OH*+*→OC−CH$_2$OH*+CH$_2$O*	C-C	0.958	1.230
r723	OCH$_2$−CO−CHOH*+*→OCH−CO−CH$_2$O*+H*	O-H	0.758	1.123
r724	OCH$_2$−CO−CHOH*+*→OCH−CO−CHOH*+H*	C-H	0.685	0.963
r725	OCH$_2$−CO−CHOH*+*→HOC−CO−CH$_2$O*+H*	C-H	1.101	0.918
r726	OCH$_2$−CO−CHOH*+*→CH$_2$−CO−CHOH*+O*	C-O	1.294	2.501
r727	OCH$_2$−CO−CHOH*+*→OCH$_2$−C−CHOH*+O*	C-O	1.708	2.350
r728	OCH$_2$−CO−CHOH*+*→CH−CO−CH$_2$O*+OH*	C-OH	1.220	1.537
r729	OCH$_2$−CO−CHOH*+*→OC−CH$_2$O*+CHOH*	C-C	1.535	1.246
r730	OCH$_2$−CO−CHOH*+*→OC−CHOH*+CH$_2$O*	C-C	1.463	1.249
r731	OCH$_2$−CO−CH$_2$O*+*→OCH−CO−CH$_2$O*+H*	C-H	0.181	0.971
r732	OCH$_2$−CO−CH$_2$O*+*→CH$_2$−CO−CH$_2$O*+O*	C-O	1.013	2.448
r733	OCH$_2$−CO−CH$_2$O*+*→OCH$_2$−C−CH$_2$O*+O*	C-O	1.609	2.230
r734	OCH$_2$−CO−CH$_2$O*+*→OC−CH$_2$O*+CH$_2$O*	C-C	1.147	1.244
r735	OCH$_2$−C−CH$_2$OH*+*→OCH$_2$−C−CH$_2$O*+H*	O-H	0.610	0.963
r736	OCH$_2$−C−CH$_2$OH*+*→OCH−C−CH$_2$OH*+H*	C-H	0.033	0.922
r737	OCH$_2$−C−CH$_2$OH*+*→OCH$_2$−C−CHOH*+H*	C-H	0.114	0.913
r738	OCH$_2$−C−CH$_2$OH*+*→CH$_2$−C−CH$_2$OH*+O*	C-O	0.794	2.305
r739	OCH$_2$−C−CH$_2$OH*+*→CH$_2$−C−CH$_2$O*+OH*	C-OH	0.619	1.563
r740	OCH$_2$−C−CH$_2$OH*+*→C−CH$_2$O*+CH$_2$OH*	C-C	1.063	1.219
r741	OCH$_2$−C−CH$_2$OH*+*→C−CH$_2$OH*+CH$_2$O*	C-C	0.719	1.235
r742	OCH$_2$−C−CHOH*+*→OCH−C−CH$_2$O*+H*	O-H	0.724	1.294
r743	OCH$_2$−C−CHOH*+*→OCH−C−CHOH*+H*	C-H	0.465	0.961
r744	OCH$_2$−C−CHOH*+*→HOC−C−CH$_2$O*+H*	C-H	0.440	0.964
r745	OCH$_2$−C−CHOH*+*→CH$_2$−C−CHOH*+O*	C-O	1.257	2.428
r746	OCH$_2$−C−CHOH*+*→CH−C−CH$_2$O*+OH*	C-OH	0.828	1.528
r747	OCH$_2$−C−CHOH*+*→C−CH$_2$O*+CHOH*	C-C	1.288	1.246
r748	OCH$_2$−C−CHOH*+*→C−CHOH*+CH$_2$O*	C-C	0.953	1.262
r749	OCH$_2$−C−CH$_2$O*+*→OCH−C−CH$_2$O*+H*	C-H	0.000	1.016
r750	OCH$_2$−C−CH$_2$O*+*→CH$_2$−C−CH$_2$O*+O*	C-O	0.921	2.388
r751	OCH$_2$−C−CH$_2$O*+*→C−CH$_2$O*+CH$_2$O*	C-C	0.880	1.244
r752	CH$_2$−CHOH−CH$_2$OH*+*→CH$_2$−CHO−CH$_2$OH*+H*	O-H	0.515	0.999
r753	CH$_2$−CHOH−CH$_2$OH*+*→CH$_2$−CHOH−CH$_2$O*+H*	O-H	0.515	1.076
r754	CH$_2$−CHOH−CH$_2$OH*+*→CH−CHOH−CH$_2$OH*+H*	C-H	0.353	0.816
r755	CH$_2$−CHOH−CH$_2$OH*+*→CH$_2$−COH−CH$_2$OH*+H*	C-H	0.110	0.842
r756	CH$_2$−CHOH−CH$_2$OH*+*→CH$_2$−CHOH−CHOH*+H*	C-H	0.353	0.816
r757	CH$_2$−CHOH−CH$_2$OH*+*→CH$_2$−CH−CH$_2$OH*+OH*	C-OH	0.638	1.603
r758	CH$_2$−CHOH−CH$_2$OH*+*→CH$_2$−CHOH−CH$_2$*+OH*	C-OH	0.893	1.617
r759	CH$_2$−CHOH−CH$_2$OH*+*→CH$_2$−CHOH*+CH$_2$OH*	C-C	1.046	1.189
r760	CH$_2$−CHOH−CH$_2$OH*+*→HOCH−CH$_2$OH*+CH$_2$*	C-C	1.007	1.190
r761	CH$_2$−CHOH−CHOH*+*→CH$_2$−CHO−CHOH*+H*	O-H	0.582	0.992
r762	CH$_2$−CHOH−CHOH*+*→OCH−CHOH−CH$_2$*+H*	O-H	0.582	1.086
r763	CH$_2$−CHOH−CHOH*+*→CH−CHOH−CHOH*+H*	C-H	0.730	0.826
r764	CH$_2$−CHOH−CHOH*+*→CH$_2$−COH−CHOH*+H*	C-H	0.170	0.886
r765	CH$_2$−CHOH−CHOH*+*→HOC−CHOH−CH$_2$*+H*	C-H	0.502	0.850
r766	CH$_2$−CHOH−CHOH*+*→CH$_2$−CH−CHOH*+OH*	C-OH	1.803	1.643
r767	CH$_2$−CHOH−CHOH*+*→CH−CHOH−CH$_2$*+OH*	C-OH	1.834	1.645
r768	CH$_2$−CHOH−CHOH*+*→CH$_2$−CHOH*+CHOH*	C-C	0.935	1.216
r769	CH$_2$−CHOH−CHOH*+*→HOCH−CHOH*+CH$_2$*	C-C	1.013	1.212
r770	CH$_2$−CHOH−CH$_2$O*+*→CH$_2$−CHO−CH$_2$O*+H*	O-H	0.596	1.315
r771	CH$_2$−CHOH−CH$_2$O*+*→CH−CHOH−CH$_2$O*+H*	C-H	0.305	0.882
r772	CH$_2$−CHOH−CH$_2$O*+*→CH$_2$−COH−CH$_2$O*+H*	C-H	0.300	0.882
r773	CH$_2$−CHOH−CH$_2$O*+*→OCH−CHOH−CH$_2$*+H*	C-H	0.458	0.865
r774	CH$_2$−CHOH−CH$_2$O*+*→CH$_2$−CHOH−CH$_2$*+O*	C-O	1.112	2.152
r775	CH$_2$−CHOH−CH$_2$O*+*→CH$_2$−CH−CH$_2$O*+OH*	C-OH	1.891	1.643
r776	CH$_2$−CHOH−CH$_2$O*+*→CH$_2$−CHOH*+CH$_2$O*	C-C	1.071	1.214
r777	CH$_2$−CHOH−CH$_2$O*+*→OCH$_2$−CHOH*+CH$_2$*	C-C	0.994	1.218
r778	CH$_2$−CHOH−CH$_2$*+*→CH$_2$−CHO−CH$_2$*+H*	O-H	0.666	0.000

Supplementary Table 16 – continues from previous page

Tag	Reaction	Type	E_a	E'_a
r779	CH_2−CHOH−CH_2*+*→CH−CHOH−CH_2*+H*	C-H	1.247	0.798
r780	CH_2−CHOH−CH_2*+*→CH_2−COH−CH_2*+H*	C-H	0.138	0.917
r781	CH_2−CHOH−CH_2*+*→CH_2−CH−CH_2*+OH*	C-OH	0.565	1.556
r782	CH_2−CHOH−CH_2*+*→CH_2−CHOH*+CH_2*	C-C	0.793	1.235
r783	CH_2−COH−CH_2OH*+*→CH_2−CO−CH_2OH*+H*	O-H	0.620	1.208
r784	CH_2−COH−CH_2OH*+*→CH_2−COH−CH_2O*+H*	O-H	0.620	1.031
r785	CH_2−COH−CH_2OH*+*→CH−COH−CH_2OH*+H*	C-H	0.444	0.885
r786	CH_2−COH−CH_2OH*+*→CH_2−COH−CHOH*+H*	C-H	0.439	0.886
r787	CH_2−COH−CH_2OH*+*→CH_2−C−CH_2OH*+OH*	C-OH	1.020	1.582
r788	CH_2−COH−CH_2OH*+*→CH_2−COH−CH_2*+OH*	C-OH	0.798	1.569
r789	CH_2−COH−CH_2OH*+*→HOC−CH_2*+CH_2OH*	C-C	1.337	1.209
r790	CH_2−COH−CH_2OH*+*→HOC−CH_2OH*+CH_2*	C-C	1.226	1.215
r791	CH_2−COH−CHOH*+*→CH_2−CO−CHOH*+H*	O-H	0.684	1.530
r792	CH_2−COH−CHOH*+*→OCH−COH−CH_2*+H*	O-H	0.684	1.279
r793	CH_2−COH−CHOH*+*→CH−COH−CHOH*+H*	C-H	0.767	0.899
r794	CH_2−COH−CHOH*+*→HOC−COH−CH_2*+H*	C-H	0.474	0.930
r795	CH_2−COH−CHOH*+*→CH_2−C−CHOH*+OH*	C-OH	0.979	1.554
r796	CH_2−COH−CHOH*+*→CH−COH−CH_2*+OH*	C-OH	0.875	1.547
r797	CH_2−COH−CHOH*+*→HOC−CH_2*+CHOH*	C-C	1.210	1.237
r798	CH_2−COH−CHOH*+*→HOC−CHOH*+CH_2*	C-C	1.207	1.237
r799	CH_2−COH−CH_2O*+*→CH_2−CO−CH_2O*+H*	O-H	0.679	1.363
r800	CH_2−COH−CH_2O*+*→CH−COH−CH_2O*+H*	C-H	0.623	0.910
r801	CH_2−COH−CH_2O*+*→OCH−COH−CH_2*+H*	C-H	0.313	0.944
r802	CH_2−COH−CH_2O*+*→CH_2−COH−CH_2*+O*	C-O	1.123	2.361
r803	CH_2−COH−CH_2O*+*→CH_2−C−CH_2O*+OH*	C-OH	1.102	1.563
r804	CH_2−COH−CH_2O*+*→HOC−CH_2*+CH_2O*	C-C	1.212	1.235
r805	CH_2−COH−CH_2O*+*→HOC−CH_2O*+CH_2*	C-C	1.075	1.241
r806	CH_2−COH−CH_2*+*→CH_2−CO−CH_2*+H*	O-H	0.731	1.534
r807	CH_2−COH−CH_2*+*→CH−COH−CH_2*+H*	C-H	0.604	0.951
r808	CH_2−COH−CH_2*+*→CH_2−C−CH_2*+OH*	C-OH	1.084	1.541
r809	CH_2−COH−CH_2*+*→HOC−CH_2*+CH_2*	C-C	1.131	1.256
r810	CH_2−CHO−CH_2OH*+*→CH_2−CHO−CH_2O*+H*	O-H	0.585	1.381
r811	CH_2−CHO−CH_2OH*+*→CH−CHO−CH_2OH*+H*	C-H	0.711	0.830
r812	CH_2−CHO−CH_2OH*+*→CH_2−CO−CH_2OH*+H*	C-H	0.064	0.899
r813	CH_2−CHO−CH_2OH*+*→CH_2−CHO−CHOH*+H*	C-H	0.467	0.856
r814	CH_2−CHO−CH_2OH*+*→CH_2−CH−CH_2OH*+O*	C-O	0.859	2.216
r815	CH_2−CHO−CH_2OH*+*→CH_2−CHO−CH_2*+OH*	C-OH	2.084	1.659
r816	CH_2−CHO−CH_2OH*+*→OCH−CH_2*+CH_2OH*	C-C	0.852	1.221
r817	CH_2−CHO−CH_2OH*+*→OCH−CH_2OH*+CH_2*	C-C	0.811	1.223
r818	CH_2−CHO−CHOH*+*→OCH−CHO−CH_2*+H*	O-H	0.641	0.935
r819	CH_2−CHO−CHOH*+*→CH−CHO−CHOH*+H*	C-H	0.662	0.877
r820	CH_2−CHO−CHOH*+*→CH_2−CO−CHOH*+H*	C-H	0.000	1.152
r821	CH_2−CHO−CHOH*+*→HOC−CHO−CH_2*+H*	C-H	0.595	0.884
r822	CH_2−CHO−CHOH*+*→CH_2−CH−CHOH*+O*	C-O	1.733	2.039
r823	CH_2−CHO−CHOH*+*→CH−CHO−CH_2*+OH*	C-OH	0.485	1.543
r824	CH_2−CHO−CHOH*+*→OCH−CH_2*+CHOH*	C-C	0.667	1.248
r825	CH_2−CHO−CHOH*+*→OCH−CHOH*+CH_2*	C-C	0.664	1.248
r826	CH_2−CHO−CH_2O*+*→CH−CHO−CH_2O*+H*	C-H	0.786	0.907
r827	CH_2−CHO−CH_2O*+*→CH_2−CO−CH_2O*+H*	C-H	0.402	0.949
r828	CH_2−CHO−CH_2O*+*→OCH−CHO−CH_2*+H*	C-H	0.997	0.885
r829	CH_2−CHO−CH_2O*+*→CH_2−CH−CH_2O*+O*	C-O	2.132	2.042
r830	CH_2−CHO−CH_2O*+*→CH_2−CHO−CH_2*+O*	C-O	2.318	1.974
r831	CH_2−CHO−CH_2O*+*→OCH−CH_2*+CH_2O*	C-C	1.112	1.246
r832	CH_2−CHO−CH_2O*+*→OCH−CH_2O*+CH_2*	C-C	1.234	1.240
r833	CH_2−CHO−CH_2*+*→CH−CHO−CH_2*+H*	C-H	0.000	1.873
r834	CH_2−CHO−CH_2*+*→CH_2−CO−CH_2*+H*	C-H	0.000	2.249
r835	CH_2−CHO−CH_2*+*→CH_2−CH−CH_2*+O*	C-O	0.000	2.533
r836	CH_2−CHO−CH_2*+*→OCH−CH_2*+CH_2*	C-C	0.000	1.818
r837	CH_2−CH−CH_2OH*+*→CH_2−CH−CH_2O*+H*	O-H	0.653	0.002
r838	CH_2−CH−CH_2OH*+*→CH−CH−CH_2OH*+H*	C-H	0.586	0.895
r839	CH_2−CH−CH_2OH*+*→CH_2−C−CH_2OH*+H*	C-H	0.567	0.897
r840	CH_2−CH−CH_2OH*+*→CH_2−CH−CHOH*+H*	C-H	1.462	0.801
r841	CH_2−CH−CH_2OH*+*→CH_2−CH−CH_2*+OH*	C-OH	0.805	1.556

Tag	Reaction	Type	E_a	E'_a
r842	CH_2−CH−CH_2OH*+*→CH−CH_2*+CH_2OH*	C-C	1.254	1.224
r843	CH_2−CH−CH_2OH*+*→CH−CH_2OH*+CH_2*	C-C	1.156	1.229
r844	CH_2−CH−CHOH*+*→OCH−CH−CH_2*+H*	O-H	0.559	2.187
r845	CH_2−CH−CHOH*+*→CH−CH−CHOH*+H*	C-H	0.000	1.149
r846	CH_2−CH−CHOH*+*→CH_2−C−CHOH*+H*	C-H	0.000	1.450
r847	CH_2−CH−CHOH*+*→HOC−CH−CH_2*+H*	C-H	0.000	1.657
r848	CH_2−CH−CHOH*+*→CH−CH−CH_2*+OH*	C-OH	0.000	1.871
r849	CH_2−CH−CHOH*+*→CH−CH_2*+CHOH*	C-C	0.019	1.251
r850	CH_2−CH−CHOH*+*→CH−CHOH*+CH_2*	C-C	0.000	1.369
r851	CH_2−CH−CH_2O*+*→CH−CH−CH_2O*+H*	C-H	0.000	1.619
r852	CH_2−CH−CH_2O*+*→CH_2−C−CH_2O*+H*	C-H	0.000	1.290
r853	CH_2−CH−CH_2O*+*→OCH−CH−CH_2*+H*	C-H	0.000	1.618
r854	CH_2−CH−CH_2O*+*→CH_2−CH−CH_2*+O*	C-O	0.139	2.417
r855	CH_2−CH−CH_2O*+*→CH−CH_2*+CH_2O*	C-C	0.067	1.250
r856	CH_2−CH−CH_2O*+*→CH−CH_2O*+CH_2*	C-C	0.266	1.240
r857	CH_2−CH−CH_2*+*→CH−CH−CH_2*+H*	C-H	0.523	0.982
r858	CH_2−CH−CH_2*+*→CH_2−C−CH_2*+H*	C-H	0.716	0.961
r859	CH_2−CH−CH_2*+*→CH−CH_2*+CH_2*	C-C	1.027	1.270
r860	CH_2−CO−CH_2OH*+*→CH_2−CO−CH_2O*+H*	O-H	0.704	1.211
r861	CH_2−CO−CH_2OH*+*→CH−CO−CH_2OH*+H*	C-H	0.690	0.922
r862	CH_2−CO−CH_2OH*+*→CH_2−CO−CHOH*+H*	C-H	0.263	0.968
r863	CH_2−CO−CH_2OH*+*→CH_2−C−CH_2OH*+O*	C-O	1.454	2.305
r864	CH_2−CO−CH_2OH*+*→CH_2−CO−CH_2*+OH*	C-OH	0.532	1.519
r865	CH_2−CO−CH_2OH*+*→OC−CH_2*+CH_2OH*	C-C	1.296	1.239
r866	CH_2−CO−CH_2OH*+*→OC−CH_2OH*+CH_2*	C-C	1.040	1.251
r867	CH_2−CO−CHOH*+*→OCH−CO−CH_2*+H*	O-H	0.805	1.134
r868	CH_2−CO−CHOH*+*→CH−CO−CHOH*+H*	C-H	0.911	0.974
r869	CH_2−CO−CHOH*+*→HOC−CO−CH_2*+H*	C-H	1.027	0.962
r870	CH_2−CO−CHOH*+*→CH_2−C−CHOH*+O*	C-O	1.823	2.428
r871	CH_2−CO−CHOH*+*→CH−CO−CH_2*+OH*	C-OH	1.246	1.520
r872	CH_2−CO−CHOH*+*→OC−CH_2*+CHOH*	C-C	1.427	1.266
r873	CH_2−CO−CHOH*+*→OC−CHOH*+CH_2*	C-C	1.352	1.270
r874	CH_2−CO−CH_2O*+*→CH−CO−CH_2O*+H*	C-H	0.782	0.967
r875	CH_2−CO−CH_2O*+*→OCH−CO−CH_2*+H*	C-H	0.473	1.000
r876	CH_2−CO−CH_2O*+*→CH_2−C−CH_2O*+O*	C-O	1.735	2.388
r877	CH_2−CO−CH_2O*+*→CH_2−CO−CH_2*+O*	C-O	1.220	2.577
r878	CH_2−CO−CH_2O*+*→OC−CH_2*+CH_2O*	C-C	1.267	1.264
r879	CH_2−CO−CH_2O*+*→OC−CH_2O*+CH_2*	C-C	1.264	1.265
r880	CH_2−CO−CH_2*+*→CH−CO−CH_2*+H*	C-H	1.003	0.995
r881	CH_2−CO−CH_2*+*→CH_2−C−CH_2*+O*	C-O	1.953	2.483
r882	CH_2−CO−CH_2*+*→OC−CH_2*+CH_2*	C-C	1.306	1.285
r883	CH_2−C−CH_2OH*+*→CH_2−C−CH_2O*+H*	O-H	0.701	1.010
r884	CH_2−C−CH_2OH*+*→CH−C−CH_2OH*+H*	C-H	0.443	0.946
r885	CH_2−C−CH_2OH*+*→CH_2−C−CHOH*+H*	C-H	0.483	0.941
r886	CH_2−C−CH_2OH*+*→CH_2−C−CH_2*+OH*	C-OH	0.875	1.541
r887	CH_2−C−CH_2OH*+*→C−CH_2*+CH_2OH*	C-C	1.070	1.249
r888	CH_2−C−CH_2OH*+*→C−CH_2OH*+CH_2*	C-C	0.911	1.256
r889	CH_2−C−CHOH*+*→OCH−C−CH_2*+H*	O-H	0.766	1.532
r890	CH_2−C−CHOH*+*→CH−C−CHOH*+H*	C-H	0.601	0.978
r891	CH_2−C−CHOH*+*→HOC−C−CH_2*+H*	C-H	0.486	0.990
r892	CH_2−C−CHOH*+*→CH−C−CH_2*+OH*	C-OH	0.655	1.501
r893	CH_2−C−CHOH*+*→C−CH_2*+CHOH*	C-C	0.955	1.276
r894	CH_2−C−CHOH*+*→C−CHOH*+CH_2*	C-C	0.806	1.283
r895	CH_2−C−CH_2O*+*→CH−C−CH_2O*+H*	C-H	0.425	0.981
r896	CH_2−C−CH_2O*+*→OCH−C−CH_2*+H*	C-H	0.100	1.016
r897	CH_2−C−CH_2O*+*→CH_2−C−CH_2*+O*	C-O	1.250	2.483
r898	CH_2−C−CH_2O*+*→C−CH_2*+CH_2O*	C-C	0.844	1.274
r899	CH_2−C−CH_2O*+*→C−CH_2O*+CH_2*	C-C	1.030	1.265
r900	CH_2−C−CH_2*+*→CH−C−CH_2*+H*	C-H	0.384	1.024
r901	CH_2−C−CH_2*+*→C−CH_2*+CH_2*	C-C	0.759	1.295
r902	HOC−CHOH−CH_2OH*+*→OC−CHOH−CH_2OH*+H*	O-H	0.528	1.411
r903	HOC−CHOH−CH_2OH*+*→HOC−CHO−CH_2OH*+H*	O-H	0.528	1.074
r904	HOC−CHOH−CH_2OH*+*→HOC−CHOH−CH_2O*+H*	O-H	0.528	1.194

Supplementary Table 16 – continues from previous page

Tag	Reaction	Type	E_a	E'_a
r905	$HOC-CHOH-CH_2OH^*+^*\to HOC-COH-CH_2OH^*+H^*$	C-H	0.336	0.827
r906	$HOC-CHOH-CH_2OH^*+^*\to HOC-CHOH-CHOH^*+H^*$	C-H	0.500	0.810
r907	$HOC-CHOH-CH_2OH^*+^*\to C-CHOH-CH_2OH^*+OH^*$	C-OH	0.467	1.588
r908	$HOC-CHOH-CH_2OH^*+^*\to HOC-CH-CH_2OH^*+OH^*$	C-OH	0.688	1.600
r909	$HOC-CHOH-CH_2OH^*+^*\to HOC-CHOH-CH_2^*+OH^*$	C-OH	0.886	1.612
r910	$HOC-CHOH-CH_2OH^*+^*\to HOC-CHOH^*+CH_2OH^*$	C-C	1.090	1.191
r911	$HOC-CHOH-CH_2OH^*+^*\to HOCH-CH_2OH^*+COH^*$	C-C	0.708	1.209
r912	$HOC-CHOH-CHOH^*+^*\to OC-CHOH-CHOH^*+H^*$	O-H	0.572	1.446
r913	$HOC-CHOH-CHOH^*+^*\to HOC-CHO-CHOH^*+H^*$	O-H	0.572	1.224
r914	$HOC-CHOH-CHOH^*+^*\to OCH-CHOH-COH^*+H^*$	O-H	0.572	1.237
r915	$HOC-CHOH-CHOH^*+^*\to HOC-COH-CHOH^*+H^*$	C-H	0.246	0.870
r916	$HOC-CHOH-CHOH^*+^*\to HOC-CHOH-COH^*+H^*$	C-H	0.436	0.850
r917	$HOC-CHOH-CHOH^*+^*\to C-CHOH-CHOH^*+OH^*$	C-OH	0.645	1.580
r918	$HOC-CHOH-CHOH^*+^*\to HOC-CH-CHOH^*+OH^*$	C-OH	0.787	1.588
r919	$HOC-CHOH-CHOH^*+^*\to CH-CHOH-COH^*+OH^*$	C-OH	1.088	1.606
r920	$HOC-CHOH-CHOH^*+^*\to HOC-CHOH^*+CHOH^*$	C-C	0.826	1.218
r921	$HOC-CHOH-CHOH^*+^*\to HOCH-CHOH^*+COH^*$	C-C	0.561	1.230
r922	$HOC-CHOH-CH_2O^*+^*\to OC-CHOH-CH_2O^*+H^*$	O-H	0.623	1.496
r923	$HOC-CHOH-CH_2O^*+^*\to HOC-CHO-CH_2O^*+H^*$	O-H	0.623	1.114
r924	$HOC-CHOH-CH_2O^*+^*\to HOC-COH-CH_2O^*+H^*$	C-H	0.217	0.912
r925	$HOC-CHOH-CH_2O^*+^*\to OCH-CHOH-COH^*+H^*$	C-H	0.566	0.874
r926	$HOC-CHOH-CH_2O^*+^*\to HOC-CHOH-CH_2^*+O^*$	C-O	1.239	2.176
r927	$HOC-CHOH-CH_2O^*+^*\to C-CHOH-CH_2O^*+OH^*$	C-OH	0.652	1.559
r928	$HOC-CHOH-CH_2O^*+^*\to HOC-CH-CH_2O^*+OH^*$	C-OH	0.464	1.548
r929	$HOC-CHOH-CH_2O^*+^*\to HOC-CHOH^*+CH_2O^*$	C-C	1.221	1.216
r930	$HOC-CHOH-CH_2O^*+^*\to OCH_2-CHOH^*+COH^*$	C-C	0.800	1.236
r931	$HOC-CHOH-CH_2^*+^*\to OC-CHOH-CH_2^*+H^*$	O-H	0.632	1.424
r932	$HOC-CHOH-CH_2^*+^*\to HOC-CHO-CH_2^*+H^*$	O-H	0.632	0.983
r933	$HOC-CHOH-CH_2^*+^*\to HOC-COH-CH_2^*+H^*$	C-H	0.106	0.930
r934	$HOC-CHOH-CH_2^*+^*\to CH-CHOH-COH^*+H^*$	C-H	0.759	0.860
r935	$HOC-CHOH-CH_2^*+^*\to C-CHOH-CH_2^*+OH^*$	C-OH	0.807	1.565
r936	$HOC-CHOH-CH_2^*+^*\to HOC-CH-CH_2^*+OH^*$	C-OH	0.392	1.541
r937	$HOC-CHOH-CH_2^*+^*\to HOC-CHOH^*+CH_2^*$	C-C	0.839	1.237
r938	$HOC-CHOH-CH_2^*+^*\to CH_2-CHOH^*+COH^*$	C-C	0.496	1.253
r939	$HOC-CHOH-COH^*+^*\to OC-CHOH-COH^*+H^*$	O-H	0.632	1.157
r940	$HOC-CHOH-COH^*+^*\to HOC-CHO-COH^*+H^*$	O-H	0.632	1.098
r941	$HOC-CHOH-COH^*+^*\to HOC-COH-COH^*+H^*$	C-H	0.000	1.156
r942	$HOC-CHOH-COH^*+^*\to C-CHOH-COH^*+OH^*$	C-OH	0.879	1.569
r943	$HOC-CHOH-COH^*+^*\to HOC-CH-COH^*+OH^*$	C-OH	0.286	1.535
r944	$HOC-CHOH-COH^*+^*\to HOC-CHOH^*+COH^*$	C-C	0.453	1.255
r945	$HOC-COH-CH_2OH^*+^*\to OC-COH-CH_2OH^*+H^*$	O-H	0.598	1.542
r946	$HOC-COH-CH_2OH^*+^*\to HOC-CO-CH_2OH^*+H^*$	O-H	0.598	1.392
r947	$HOC-COH-CH_2OH^*+^*\to HOC-COH-CH_2O^*+H^*$	O-H	0.598	1.467
r948	$HOC-COH-CH_2OH^*+^*\to HOC-COH-CHOH^*+H^*$	C-H	0.428	0.870
r949	$HOC-COH-CH_2OH^*+^*\to C-COH-CH_2OH^*+OH^*$	C-OH	0.560	1.564
r950	$HOC-COH-CH_2OH^*+^*\to HOC-C-CH_2OH^*+OH^*$	C-OH	0.646	1.569
r951	$HOC-COH-CH_2OH^*+^*\to HOC-COH-CH_2^*+OH^*$	C-OH	0.502	1.561
r952	$HOC-COH-CH_2OH^*+^*\to HOC-COH^*+CH_2OH^*$	C-C	0.943	1.221
r953	$HOC-COH-CH_2OH^*+^*\to HOC-CH_2OH^*+COH^*$	C-C	0.686	1.233
r954	$HOC-COH-CHOH^*+^*\to OC-COH-CHOH^*+H^*$	O-H	0.662	1.425
r955	$HOC-COH-CHOH^*+^*\to HOC-CO-CHOH^*+H^*$	O-H	0.662	1.427
r956	$HOC-COH-CHOH^*+^*\to OCH-COH-COH^*+H^*$	O-H	0.662	1.714
r957	$HOC-COH-CHOH^*+^*\to HOC-COH-COH^*+H^*$	C-H	0.016	0.962
r958	$HOC-COH-CHOH^*+^*\to C-COH-CHOH^*+OH^*$	C-OH	0.518	1.536
r959	$HOC-COH-CHOH^*+^*\to HOC-C-CHOH^*+OH^*$	C-OH	0.691	1.546
r960	$HOC-COH-CHOH^*+^*\to CH-COH-COH^*+OH^*$	C-OH	0.347	1.526
r961	$HOC-COH-CHOH^*+^*\to HOC-COH^*+CHOH^*$	C-C	0.812	1.248
r962	$HOC-COH-CHOH^*+^*\to HOC-CHOH^*+COH^*$	C-C	0.663	1.255
r963	$HOC-COH-CH_2O^*+^*\to OC-COH-CH_2O^*+H^*$	O-H	0.723	1.314
r964	$HOC-COH-CH_2O^*+^*\to HOC-CO-CH_2O^*+H^*$	O-H	0.723	0.789
r965	$HOC-COH-CH_2O^*+^*\to OCH-COH-COH^*+H^*$	C-H	0.347	0.973
r966	$HOC-COH-CH_2O^*+^*\to HOC-COH-CH_2^*+O^*$	C-O	1.330	2.397
r967	$HOC-COH-CH_2O^*+^*\to C-COH-CH_2O^*+OH^*$	C-OH	0.754	1.525

Supplementary Table 16 – continues from previous page

Tag	Reaction	Type	E_a	E'_a
r968	HOC−COH−CH$_2$O*+*→HOC−C−CH$_2$O*+OH*	C-OH	1.002	1.539
r969	HOC−COH−CH$_2$O*+*→HOC−COH*+CH$_2$O*	C-C	1.277	1.246
r970	HOC−COH−CH$_2$O*+*→HOC−CH$_2$O*+COH*	C-C	0.994	1.259
r971	HOC−COH−CH$_2$*+*→OC−COH−CH$_2$*+H*	O-H	0.750	1.534
r972	HOC−COH−CH$_2$*+*→HOC−CO−CH$_2$*+H*	O-H	0.750	1.074
r973	HOC−COH−CH$_2$*+*→CH−COH−COH*+H*	C-H	0.422	0.985
r974	HOC−COH−CH$_2$*+*→C−COH−CH$_2$*+OH*	C-OH	0.712	1.511
r975	HOC−COH−CH$_2$*+*→HOC−C−CH$_2$*+OH*	C-OH	0.900	1.522
r976	HOC−COH−CH$_2$*+*→HOC−COH*+CH$_2$*	C-C	1.025	1.267
r977	HOC−COH−CH$_2$*+*→HOC−CH$_2$*+COH*	C-C	0.879	1.274
r978	HOC−COH−COH*+*→OC−COH−COH*+H*	O-H	0.797	0.645
r979	HOC−COH−COH*+*→HOC−CO−COH*+H*	O-H	0.797	1.081
r980	HOC−COH−COH*+*→C−COH−COH*+OH*	C-OH	0.801	1.497
r981	HOC−COH−COH*+*→HOC−C−COH*+OH*	C-OH	1.303	1.526
r982	HOC−COH−COH*+*→HOC−COH*+COH*	C-C	0.971	1.285
r983	HOC−CHO−CH$_2$OH*+*→OC−CHO−CH$_2$OH*+H*	O-H	0.606	1.277
r984	HOC−CHO−CH$_2$OH*+*→HOC−CHO−CH$_2$O*+H*	O-H	0.606	1.217
r985	HOC−CHO−CH$_2$OH*+*→HOC−CO−CH$_2$OH*+H*	C-H	0.165	0.905
r986	HOC−CHO−CH$_2$OH*+*→HOC−CHO−CHOH*+H*	C-H	0.457	0.873
r987	HOC−CHO−CH$_2$OH*+*→HOC−CH−CH$_2$OH*+O*	C-O	0.983	2.225
r988	HOC−CHO−CH$_2$OH*+*→C−CHO−CH$_2$OH*+OH*	C-OH	0.982	1.586
r989	HOC−CHO−CH$_2$OH*+*→HOC−CHO−CH$_2$*+OH*	C-OH	1.059	1.590
r990	HOC−CHO−CH$_2$OH*+*→OCH−COH*+CH$_2$OH*	C-C	1.045	1.219
r991	HOC−CHO−CH$_2$OH*+*→OCH−CH$_2$OH*+COH*	C-C	0.574	1.241
r992	HOC−CHO−CHOH*+*→OC−CHO−CHOH*+H*	O-H	0.666	1.376
r993	HOC−CHO−CHOH*+*→OCH−CHO−COH*+H*	O-H	0.666	0.937
r994	HOC−CHO−CHOH*+*→HOC−CO−CHOH*+H*	C-H	0.207	0.945
r995	HOC−CHO−CHOH*+*→HOC−CHO−COH*+H*	C-H	0.666	0.895
r996	HOC−CHO−CHOH*+*→HOC−CH−CHOH*+O*	C-O	1.253	2.279
r997	HOC−CHO−CHOH*+*→C−CHO−CHOH*+OH*	C-OH	1.110	1.569
r998	HOC−CHO−CHOH*+*→CH−CHO−COH*+OH*	C-OH	1.498	1.591
r999	HOC−CHO−CHOH*+*→OCH−COH*+CHOH*	C-C	0.887	1.246
r1000	HOC−CHO−CHOH*+*→OCH−CHOH*+COH*	C-C	0.453	1.266
r1001	HOC−CHO−CH$_2$O*+*→OC−CHO−CH$_2$O*+H*	O-H	0.694	1.220
r1002	HOC−CHO−CH$_2$O*+*→HOC−CO−CH$_2$O*+H*	C-H	0.648	0.918
r1003	HOC−CHO−CH$_2$O*+*→OCH−CHO−COH*+H*	C-H	0.823	0.900
r1004	HOC−CHO−CH$_2$O*+*→HOC−CH−CH$_2$O*+O*	C-O	0.980	2.450
r1005	HOC−CHO−CH$_2$O*+*→HOC−CHO−CH$_2$*+O*	C-O	1.473	2.270
r1006	HOC−CHO−CH$_2$O*+*→C−CHO−CH$_2$O*+OH*	C-OH	0.801	1.539
r1007	HOC−CHO−CH$_2$O*+*→OCH−COH*+CH$_2$O*	C-C	1.120	1.244
r1008	HOC−CHO−CH$_2$O*+*→OCH−CH$_2$O*+COH*	C-C	0.812	1.258
r1009	HOC−CHO−CH$_2$*+*→OC−CHO−CH$_2$*+H*	O-H	0.682	1.383
r1010	HOC−CHO−CH$_2$*+*→HOC−CO−CH$_2$*+H*	C-H	0.165	0.962
r1011	HOC−CHO−CH$_2$*+*→CH−CHO−COH*+H*	C-H	0.905	0.882
r1012	HOC−CHO−CH$_2$*+*→HOC−CH−CH$_2$*+O*	C-O	0.809	2.484
r1013	HOC−CHO−CH$_2$*+*→C−CHO−CH$_2$*+OH*	C-OH	1.230	1.569
r1014	HOC−CHO−CH$_2$*+*→OCH−COH*+CH$_2$*	C-C	0.599	1.265
r1015	HOC−CHO−CH$_2$*+*→OCH−CH$_2$*+COH*	C-C	0.169	1.285
r1016	HOC−CHO−COH*+*→OC−CHO−COH*+H*	O-H	0.699	1.073
r1017	HOC−CHO−COH*+*→HOC−CO−COH*+H*	C-H	0.016	0.990
r1018	HOC−CHO−COH*+*→HOC−CH−COH*+O*	C-O	0.852	2.510
r1019	HOC−CHO−COH*+*→C−CHO−COH*+OH*	C-OH	1.369	1.570
r1020	HOC−CHO−COH*+*→OCH−COH*+COH*	C-C	0.328	1.283
r1021	HOC−CH−CH$_2$OH*+*→OC−CH−CH$_2$OH*+H*	O-H	0.658	1.498
r1022	HOC−CH−CH$_2$OH*+*→HOC−CH−CH$_2$O*+H*	O-H	0.658	1.497
r1023	HOC−CH−CH$_2$OH*+*→HOC−C−CH$_2$OH*+H*	C-H	0.414	0.917
r1024	HOC−CH−CH$_2$OH*+*→HOC−CH−CHOH*+H*	C-H	0.689	0.887
r1025	HOC−CH−CH$_2$OH*+*→C−CH−CH$_2$OH*+OH*	C-OH	0.949	1.562
r1026	HOC−CH−CH$_2$OH*+*→HOC−CH−CH$_2$*+OH*	C-OH	0.577	1.541
r1027	HOC−CH−CH$_2$OH*+*→CH−COH*+CH$_2$OH*	C-C	1.250	1.226
r1028	HOC−CH−CH$_2$OH*+*→CH−CH$_2$OH*+COH*	C-C	0.804	1.247
r1029	HOC−CH−CHOH*+*→OC−CH−CHOH*+H*	O-H	0.687	1.755
r1030	HOC−CH−CHOH*+*→OCH−CH−COH*+H*	O-H	0.687	1.528

Supplementary Table 16 – continues from previous page

Tag	Reaction	Type	E_a	E'_a
r1031	HOC−CH−CHOH*+*→HOC−C−CHOH*+H*	C-H	0.275	0.953
r1032	HOC−CH−CHOH*+*→HOC−CH−COH*+H*	C-H	0.110	0.971
r1033	HOC−CH−CHOH*+*→C−CH−CHOH*+OH*	C-OH	0.532	1.526
r1034	HOC−CH−CHOH*+*→CH−CH−COH*+OH*	C-OH	0.201	1.507
r1035	HOC−CH−CHOH*+*→CH−COH*+CHOH*	C-C	0.875	1.253
r1036	HOC−CH−CHOH*+*→CH−CHOH*+COH*	C-C	0.397	1.276
r1037	HOC−CH−CH_2O*+*→OC−CH−CH_2O*+H*	O-H	0.778	1.221
r1038	HOC−CH−CH_2O*+*→HOC−C−CH_2O*+H*	C-H	0.816	0.964
r1039	HOC−CH−CH_2O*+*→OCH−CH−COH*+H*	C-H	0.768	0.969
r1040	HOC−CH−CH_2O*+*→HOC−CH−CH_2*+O*	C-O	1.482	2.484
r1041	HOC−CH−CH_2O*+*→C−CH−CH_2O*+OH*	C-OH	0.855	1.508
r1042	HOC−CH−CH_2O*+*→CH−COH*+CH_2O*	C-C	1.553	1.251
r1043	HOC−CH−CH_2O*+*→CH−CH_2O*+COH*	C-C	1.404	1.258
r1044	HOC−CH−CH_2*+*→OC−CH−CH_2*+H*	O-H	0.796	1.468
r1045	HOC−CH−CH_2*+*→HOC−C−CH_2*+H*	C-H	0.693	0.990
r1046	HOC−CH−CH_2*+*→CH−CH−COH*+H*	C-H	0.473	1.014
r1047	HOC−CH−CH_2*+*→C−CH−CH_2*+OH*	C-OH	1.071	1.513
r1048	HOC−CH−CH_2*+*→CH−COH*+CH_2*	C-C	1.236	1.272
r1049	HOC−CH−CH_2*+*→CH−CH_2*+COH*	C-C	0.888	1.289
r1050	HOC−CH−COH*+*→OC−CH−COH*+H*	O-H	0.810	1.663
r1051	HOC−CH−COH*+*→HOC−C−COH*+H*	C-H	0.852	0.984
r1052	HOC−CH−COH*+*→C−CH−COH*+OH*	C-OH	0.642	1.482
r1053	HOC−CH−COH*+*→CH−COH*+COH*	C-C	0.949	1.290
r1054	HOC−CO−CH_2OH*+*→OC−CO−CH_2OH*+H*	O-H	0.712	1.299
r1055	HOC−CO−CH_2OH*+*→HOC−CO−CH_2O*+H*	O-H	0.712	0.854
r1056	HOC−CO−CH_2OH*+*→HOC−CO−CHOH*+H*	C-H	0.531	0.945
r1057	HOC−CO−CH_2OH*+*→HOC−C−CH_2OH*+O*	C-O	1.354	2.360
r1058	HOC−CO−CH_2OH*+*→C−CO−CH_2OH*+OH*	C-OH	1.111	1.550
r1059	HOC−CO−CH_2OH*+*→HOC−CO−CH_2*+OH*	C-OH	0.951	1.541
r1060	HOC−CO−CH_2OH*+*→OC−COH*+CH_2OH*	C-C	1.296	1.242
r1061	HOC−CO−CH_2OH*+*→OC−CH_2OH*+COH*	C-C	0.706	1.269
r1062	HOC−CO−CHOH*+*→OC−CO−CHOH*+H*	O-H	0.771	1.519
r1063	HOC−CO−CHOH*+*→OCH−CO−COH*+H*	O-H	0.771	1.305
r1064	HOC−CO−CHOH*+*→HOC−CO−COH*+H*	C-H	0.525	0.990
r1065	HOC−CO−CHOH*+*→HOC−C−CHOH*+O*	C-O	1.495	2.461
r1066	HOC−CO−CHOH*+*→C−CO−CHOH*+OH*	C-OH	1.140	1.527
r1067	HOC−CO−CHOH*+*→CH−CO−COH*+OH*	C-OH	1.463	1.546
r1068	HOC−CO−CHOH*+*→OC−COH*+CHOH*	C-C	1.137	1.269
r1069	HOC−CO−CHOH*+*→OC−CHOH*+COH*	C-C	0.727	1.288
r1070	HOC−CO−CH_2O*+*→OC−CO−CH_2O*+H*	O-H	0.732	1.591
r1071	HOC−CO−CH_2O*+*→OCH−CO−COH*+H*	C-H	0.191	0.997
r1072	HOC−CO−CH_2O*+*→HOC−C−CH_2O*+O*	C-O	1.143	2.490
r1073	HOC−CO−CH_2O*+*→HOC−CO−CH_2*+O*	C-O	1.160	2.484
r1074	HOC−CO−CH_2O*+*→C−CO−CH_2O*+OH*	C-OH	0.845	1.526
r1075	HOC−CO−CH_2O*+*→OC−COH*+CH_2O*	C-C	0.903	1.267
r1076	HOC−CO−CH_2O*+*→OC−CH_2O*+COH*	C-C	0.565	1.283
r1077	HOC−CO−CH_2*+*→OC−CO−CH_2*+H*	O-H	0.796	1.592
r1078	HOC−CO−CH_2*+*→CH−CO−COH*+H*	C-H	1.045	0.953
r1079	HOC−CO−CH_2*+*→HOC−C−CH_2*+O*	C-O	1.389	2.563
r1080	HOC−CO−CH_2*+*→C−CO−CH_2*+OH*	C-OH	0.809	1.498
r1081	HOC−CO−CH_2*+*→OC−COH*+CH_2*	C-C	0.908	1.288
r1082	HOC−CO−CH_2*+*→OC−CH_2*+COH*	C-C	0.574	1.303
r1083	HOC−CO−COH*+*→OC−CO−COH*+H*	O-H	0.838	0.768
r1084	HOC−CO−COH*+*→HOC−C−COH*+O*	C-O	1.729	2.546
r1085	HOC−CO−COH*+*→C−CO−COH*+OH*	C-OH	1.090	1.497
r1086	HOC−CO−COH*+*→OC−COH*+COH*	C-C	0.815	1.306
r1087	HOC−C−CH_2OH*+*→OC−C−CH_2OH*+H*	O-H	0.730	1.231
r1088	HOC−C−CH_2OH*+*→HOC−C−CH_2O*+H*	O-H	0.730	1.213
r1089	HOC−C−CH_2OH*+*→HOC−C−CHOH*+H*	C-H	0.579	0.953
r1090	HOC−C−CH_2OH*+*→C−C−CH_2OH*+OH*	C-OH	1.062	1.540
r1091	HOC−C−CH_2OH*+*→HOC−C−CH_2*+OH*	C-OH	0.765	1.522
r1092	HOC−C−CH_2OH*+*→C−COH*+CH_2OH*	C-C	1.497	1.238
r1093	HOC−C−CH_2OH*+*→C−CH_2OH*+COH*	C-C	0.733	1.274

Supplementary Table 16 – continues from previous page

Tag	Reaction	Type	E_a	E'_a
r1094	HOC−C−CHOH*+*→OC−C−CHOH*+H*	O-H	0.784	1.015
r1095	HOC−C−CHOH*+*→OCH−C−COH*+H*	O-H	0.784	1.535
r1096	HOC−C−CHOH*+*→HOC−C−COH*+H*	C-H	0.669	0.984
r1097	HOC−C−CHOH*+*→C−C−CHOH*+OH*	C-OH	1.149	1.523
r1098	HOC−C−CHOH*+*→CH−C−COH*+OH*	C-OH	1.014	1.515
r1099	HOC−C−CHOH*+*→C−COH*+CHOH*	C-C	1.297	1.265
r1100	HOC−C−CHOH*+*→C−CHOH*+COH*	C-C	0.542	1.301
r1101	HOC−C−CH_2O*+*→OC−C−CH_2O*+H*	O-H	0.800	1.019
r1102	HOC−C−CH_2O*+*→OCH−C−COH*+H*	C-H	0.385	1.026
r1103	HOC−C−CH_2O*+*→HOC−C−CH_2*+O*	C-O	1.413	2.563
r1104	HOC−C−CH_2O*+*→C−C−CH_2O*+OH*	C-OH	1.236	1.521
r1105	HOC−C−CH_2O*+*→C−COH*+CH_2O*	C-C	1.445	1.263
r1106	HOC−C−CH_2O*+*→C−CH_2O*+COH*	C-C	1.025	1.283
r1107	HOC−C−CH_2*+*→OC−C−CH_2*+H*	O-H	0.839	0.931
r1108	HOC−C−CH_2*+*→CH−C−COH*+H*	C-H	0.885	1.002
r1109	HOC−C−CH_2*+*→C−C−CH_2*+OH*	C-OH	1.335	1.511
r1110	HOC−C−CH_2*+*→C−COH*+CH_2*	C-C	1.277	1.284
r1111	HOC−C−CH_2*+*→C−CH_2*+COH*	C-C	0.672	1.313
r1112	HOC−C−COH*+*→OC−C−COH*+H*	O-H	0.829	1.325
r1113	HOC−C−COH*+*→C−C−COH*+OH*	C-OH	1.223	1.509
r1114	HOC−C−COH*+*→C−COH*+COH*	C-C	0.825	1.302
r1115	OCH−CHOH−CH_2OH*+*→OCH−CHO−CH_2OH*+H*	O-H	0.568	0.991
r1116	OCH−CHOH−CH_2OH*+*→OCH−CHOH−CH_2O*+H*	O-H	0.568	1.010
r1117	OCH−CHOH−CH_2OH*+*→OC−CHOH−CH_2OH*+H*	C-H	0.262	0.865
r1118	OCH−CHOH−CH_2OH*+*→OCH−COH−CH_2OH*+H*	C-H	0.187	0.874
r1119	OCH−CHOH−CH_2OH*+*→OCH−CHOH−CHOH*+H*	C-H	0.667	0.822
r1120	OCH−CHOH−CH_2OH*+*→CH−CHOH−CH_2OH*+O*	C-O	1.107	2.082
r1121	OCH−CHOH−CH_2OH*+*→OCH−CH−CH_2OH*+OH*	C-OH	0.614	1.580
r1122	OCH−CHOH−CH_2OH*+*→OCH−CHOH−CH_2*+OH*	C-OH	0.999	1.602
r1123	OCH−CHOH−CH_2OH*+*→OCH−CHOH*+CH_2OH*	C-C	1.132	1.202
r1124	OCH−CHOH−CH_2OH*+*→HOCH−CH_2OH*+CHO*	C-C	1.285	1.195
r1125	OCH−CHOH−CHOH*+*→OCH−CHO−CHOH*+H*	O-H	0.590	1.097
r1126	OCH−CHOH−CHOH*+*→OCH−CHOH−CHO*+H*	O-H	0.590	1.127
r1127	OCH−CHOH−CHOH*+*→OC−CHOH−CHOH*+H*	C-H	0.146	0.895
r1128	OCH−CHOH−CHOH*+*→OCH−COH−CHOH*+H*	C-H	0.066	0.903
r1129	OCH−CHOH−CHOH*+*→OCH−CHOH−COH*+H*	C-H	0.335	0.874
r1130	OCH−CHOH−CHOH*+*→CH−CHOH−CHOH*+O*	C-O	1.192	2.108
r1131	OCH−CHOH−CHOH*+*→OCH−CH−CHOH*+OH*	C-OH	0.184	1.546
r1132	OCH−CHOH−CHOH*+*→CH−CHOH−CHO*+OH*	C-OH	0.799	1.581
r1133	OCH−CHOH−CHOH*+*→OCH−CHOH*+CHOH*	C-C	0.713	1.229
r1134	OCH−CHOH−CHOH*+*→HOCH−CHOH*+CHO*	C-C	0.983	1.216
r1135	OCH−CHOH−CH_2O*+*→OCH−CHO−CH_2O*+H*	O-H	0.631	1.338
r1136	OCH−CHOH−CH_2O*+*→OC−CHOH−CH_2O*+H*	C-H	0.112	0.929
r1137	OCH−CHOH−CH_2O*+*→OCH−COH−CH_2O*+H*	C-H	0.145	0.926
r1138	OCH−CHOH−CH_2O*+*→OCH−CHOH−CHO*+H*	C-H	0.625	0.874
r1139	OCH−CHOH−CH_2O*+*→CH−CHOH−CH_2O*+O*	C-O	1.056	2.263
r1140	OCH−CHOH−CH_2O*+*→OCH−CHOH−CH_2*+O*	C-O	1.181	2.218
r1141	OCH−CHOH−CH_2O*+*→OCH−CH−CH_2O*+OH*	C-OH	0.477	1.546
r1142	OCH−CHOH−CH_2O*+*→OCH−CHOH*+CH_2O*	C-C	1.039	1.227
r1143	OCH−CHOH−CH_2O*+*→OCH_2−CHOH*+CHO*	C-C	1.153	1.222
r1144	OCH−CHOH−CH_2*+*→OCH−CHO−CH_2*+H*	O-H	0.654	0.854
r1145	OCH−CHOH−CH_2*+*→OC−CHOH−CH_2*+H*	C-H	0.291	0.927
r1146	OCH−CHOH−CH_2*+*→OCH−COH−CH_2*+H*	C-H	0.138	0.944
r1147	OCH−CHOH−CH_2*+*→CH−CHOH−CHO*+H*	C-H	0.563	0.898
r1148	OCH−CHOH−CH_2*+*→CH−CHOH−CH_2*+O*	C-O	1.848	2.032
r1149	OCH−CHOH−CH_2*+*→OCH−CH−CH_2*+OH*	C-OH	0.579	1.543
r1150	OCH−CHOH−CH_2*+*→OCH−CHOH*+CH_2*	C-C	0.758	1.248
r1151	OCH−CHOH−CH_2*+*→CH_2−CHOH*+CHO*	C-C	0.949	1.239
r1152	OCH−CHOH−COH*+*→OCH−CHO−COH*+H*	O-H	0.667	0.926
r1153	OCH−CHOH−COH*+*→OC−CHOH−CHO*+H*	O-H	0.667	1.427
r1154	OCH−CHOH−COH*+*→OC−CHOH−COH*+H*	C-H	0.626	0.901
r1155	OCH−CHOH−COH*+*→OCH−COH−COH*+H*	C-H	0.000	1.012
r1156	OCH−CHOH−COH*+*→CH−CHOH−COH*+O*	C-O	1.474	2.203

Tag	Reaction	Type	E_a	E'_a
r1157	OCH−CHOH−COH*+*→OCH−CH−COH*+OH*	C-OH	0.559	1.536
r1158	OCH−CHOH−COH*+*→C−CHOH−CHO*+OH*	C-OH	0.939	1.558
r1159	OCH−CHOH−COH*+*→OCH−CHOH*+COH*	C-C	0.466	1.266
r1160	OCH−CHOH−COH*+*→HOC−CHOH*+CHO*	C-C	1.000	1.241
r1161	OCH−CHOH−CHO*+*→OCH−CHO−CHO*+H*	O-H	0.667	1.030
r1162	OCH−CHOH−CHO*+*→OC−CHOH−CHO*+H*	C-H	0.186	0.948
r1163	OCH−CHOH−CHO*+*→OCH−COH−CHO*+H*	C-H	0.000	1.034
r1164	OCH−CHOH−CHO*+*→CH−CHOH−CHO*+O*	C-O	1.186	2.307
r1165	OCH−CHOH−CHO*+*→OCH−CH−CHO*+OH*	C-OH	0.246	1.518
r1166	OCH−CHOH−CHO*+*→OCH−CHOH*+CHO*	C-C	0.760	1.252
r1167	OCH−COH−CH_2OH*+*→OCH−CO−CH_2OH*+H*	O-H	0.666	1.405
r1168	OCH−COH−CH_2OH*+*→OCH−COH−CH_2O*+H*	O-H	0.666	1.202
r1169	OCH−COH−CH_2OH*+*→OC−COH−CH_2OH*+H*	C-H	0.451	0.919
r1170	OCH−COH−CH_2OH*+*→OCH−COH−CHOH*+H*	C-H	0.598	0.903
r1171	OCH−COH−CH_2OH*+*→CH−COH−CH_2OH*+O*	C-O	1.274	2.273
r1172	OCH−COH−CH_2OH*+*→OCH−C−CH_2OH*+OH*	C-OH	1.069	1.566
r1173	OCH−COH−CH_2OH*+*→OCH−COH−CH_2*+OH*	C-OH	0.830	1.552
r1174	OCH−COH−CH_2OH*+*→OCH−COH*+CH_2OH*	C-C	1.466	1.219
r1175	OCH−COH−CH_2OH*+*→HOC−CH_2OH*+CHO*	C-C	1.458	1.219
r1176	OCH−COH−CHOH*+*→OCH−CO−CHOH*+H*	O-H	0.710	1.326
r1177	OCH−COH−CHOH*+*→OCH−COH−CHO*+H*	O-H	0.710	1.443
r1178	OCH−COH−CHOH*+*→OC−COH−CHOH*+H*	C-H	0.520	0.945
r1179	OCH−COH−CHOH*+*→OCH−COH−COH*+H*	C-H	0.259	0.973
r1180	OCH−COH−CHOH*+*→CH−COH−CHOH*+O*	C-O	1.480	2.310
r1181	OCH−COH−CHOH*+*→OCH−C−CHOH*+OH*	C-OH	0.943	1.541
r1182	OCH−COH−CHOH*+*→CH−COH−CHO*+OH*	C-OH	0.879	1.537
r1183	OCH−COH−CHOH*+*→OCH−COH*+CHOH*	C-C	1.197	1.246
r1184	OCH−COH−CHOH*+*→HOC−CHOH*+CHO*	C-C	1.298	1.241
r1185	OCH−COH−CH_2O*+*→OCH−CO−CH_2O*+H*	O-H	0.743	1.215
r1186	OCH−COH−CH_2O*+*→OC−COH−CH_2O*+H*	C-H	0.520	0.969
r1187	OCH−COH−CH_2O*+*→OCH−COH−CHO*+H*	C-H	0.472	0.974
r1188	OCH−COH−CH_2O*+*→CH−COH−CH_2O*+O*	C-O	1.623	2.342
r1189	OCH−COH−CH_2O*+*→OCH−COH−CH_2*+O*	C-O	1.371	2.434
r1190	OCH−COH−CH_2O*+*→OCH−C−CH_2O*+OH*	C-OH	1.096	1.536
r1191	OCH−COH−CH_2O*+*→OCH−COH*+CH_2O*	C-C	1.466	1.244
r1192	OCH−COH−CH_2O*+*→HOC−CH_2O*+CHO*	C-C	1.432	1.245
r1193	OCH−COH−CH_2*+*→OCH−CO−CH_2*+H*	O-H	0.769	1.350
r1194	OCH−COH−CH_2*+*→OC−COH−CH_2*+H*	C-H	0.360	1.006
r1195	OCH−COH−CH_2*+*→CH−COH−CHO*+H*	C-H	0.725	0.967
r1196	OCH−COH−CH_2*+*→CH−COH−CH_2*+O*	C-O	1.500	2.454
r1197	OCH−COH−CH_2*+*→OCH−C−CH_2*+OH*	C-OH	0.760	1.506
r1198	OCH−COH−CH_2*+*→OCH−COH*+CH_2*	C-C	1.210	1.265
r1199	OCH−COH−CH_2*+*→HOC−CH_2*+CHO*	C-C	1.314	1.260
r1200	OCH−COH−COH*+*→OCH−CO−COH*+H*	O-H	0.812	1.059
r1201	OCH−COH−COH*+*→OC−COH−CHO*+H*	O-H	0.812	1.369
r1202	OCH−COH−COH*+*→OC−COH−COH*+H*	C-H	1.206	0.948
r1203	OCH−COH−COH*+*→CH−COH−COH*+O*	C-O	1.544	2.548
r1204	OCH−COH−COH*+*→OCH−C−COH*+OH*	C-OH	0.946	1.499
r1205	OCH−COH−COH*+*→C−COH−CHO*+OH*	C-OH	0.835	1.493
r1206	OCH−COH−COH*+*→OCH−COH*+COH*	C-C	1.124	1.283
r1207	OCH−COH−COH*+*→HOC−COH*+CHO*	C-C	1.374	1.271
r1208	OCH−COH−CHO*+*→OCH−CO−CHO*+H*	O-H	0.815	1.304
r1209	OCH−COH−CHO*+*→OC−COH−CHO*+H*	C-H	0.489	1.027
r1210	OCH−COH−CHO*+*→CH−COH−CHO*+O*	C-O	1.697	2.499
r1211	OCH−COH−CHO*+*→OCH−C−CHO*+OH*	C-OH	0.759	1.487
r1212	OCH−COH−CHO*+*→OCH−COH*+CHO*	C-C	1.440	1.269
r1213	OCH−CHO−CH_2OH*+*→OCH−CHO−CH_2O*+H*	O-H	0.628	1.355
r1214	OCH−CHO−CH_2OH*+*→OC−CHO−CH_2OH*+H*	C-H	0.384	0.898
r1215	OCH−CHO−CH_2OH*+*→OCH−CO−CH_2OH*+H*	C-H	0.000	1.003
r1216	OCH−CHO−CH_2OH*+*→OCH−CHO−CHOH*+H*	C-H	0.633	0.871
r1217	OCH−CHO−CH_2OH*+*→CH−CHO−CH_2OH*+O*	C-O	1.428	2.120
r1218	OCH−CHO−CH_2OH*+*→OCH−CH−CH_2OH*+O*	C-O	0.895	2.315
r1219	OCH−CHO−CH_2OH*+*→OCH−CHO−CH_2*+OH*	C-OH	1.210	1.590

Supplementary Table 16 – continues from previous page

Tag	Reaction	Type	E_a	E'_a
r1220	$OCH-CHO-CH_2OH^{*}+^{*}\rightarrow OCH-CHO^{*}+CH_2OH^{*}$	C-C	0.945	1.231
r1221	$OCH-CHO-CH_2OH^{*}+^{*}\rightarrow OCH-CH_2OH^{*}+CHO^{*}$	C-C	1.027	1.227
r1222	$OCH-CHO-CHOH^{*}+^{*}\rightarrow OCH-CHO-CHO^{*}+H^{*}$	O-H	0.663	1.056
r1223	$OCH-CHO-CHOH^{*}+^{*}\rightarrow OC-CHO-CHOH^{*}+H^{*}$	C-H	0.211	0.942
r1224	$OCH-CHO-CHOH^{*}+^{*}\rightarrow OCH-CO-CHOH^{*}+H^{*}$	C-H	0.016	0.963
r1225	$OCH-CHO-CHOH^{*}+^{*}\rightarrow OCH-CHO-COH^{*}+H^{*}$	C-H	0.607	0.900
r1226	$OCH-CHO-CHOH^{*}+^{*}\rightarrow CH-CHO-CHOH^{*}+O^{*}$	C-O	1.311	2.250
r1227	$OCH-CHO-CHOH^{*}+^{*}\rightarrow OCH-CH-CHOH^{*}+O^{*}$	C-O	0.731	2.463
r1228	$OCH-CHO-CHOH^{*}+^{*}\rightarrow CH-CHO-CHO^{*}+OH^{*}$	C-OH	1.105	1.570
r1229	$OCH-CHO-CHOH^{*}+^{*}\rightarrow OCH-CHO^{*}+CHOH^{*}$	C-C	0.610	1.258
r1230	$OCH-CHO-CHOH^{*}+^{*}\rightarrow OCH-CHOH^{*}+CHO^{*}$	C-C	0.729	1.252
r1231	$OCH-CHO-CH_2O^{*}+^{*}\rightarrow OC-CHO-CH_2O^{*}+H^{*}$	C-H	0.689	0.943
r1232	$OCH-CHO-CH_2O^{*}+^{*}\rightarrow OCH-CO-CH_2O^{*}+H^{*}$	C-H	0.426	0.971
r1233	$OCH-CHO-CH_2O^{*}+^{*}\rightarrow OCH-CHO-CHO^{*}+H^{*}$	C-H	1.004	0.909
r1234	$OCH-CHO-CH_2O^{*}+^{*}\rightarrow CH-CHO-CH_2O^{*}+O^{*}$	C-O	1.570	2.334
r1235	$OCH-CHO-CH_2O^{*}+^{*}\rightarrow OCH-CH-CH_2O^{*}+O^{*}$	C-O	1.222	2.461
r1236	$OCH-CHO-CH_2O^{*}+^{*}\rightarrow OCH-CHO-CH_2^{*}+O^{*}$	C-O	1.741	2.271
r1237	$OCH-CHO-CH_2O^{*}+^{*}\rightarrow OCH-CHO^{*}+CH_2O^{*}$	C-C	1.136	1.256
r1238	$OCH-CHO-CH_2O^{*}+^{*}\rightarrow OCH-CH_2O^{*}+CHO^{*}$	C-C	1.381	1.244
r1239	$OCH-CHO-CH_2^{*}+^{*}\rightarrow OC-CHO-CH_2^{*}+H^{*}$	C-H	0.257	0.952
r1240	$OCH-CHO-CH_2^{*}+^{*}\rightarrow OCH-CO-CH_2^{*}+H^{*}$	C-H	0.000	1.186
r1241	$OCH-CHO-CH_2^{*}+^{*}\rightarrow CH-CHO-CHO^{*}+H^{*}$	C-H	0.593	0.916
r1242	$OCH-CHO-CH_2^{*}+^{*}\rightarrow CH-CHO-CH_2^{*}+O^{*}$	C-O	0.836	2.476
r1243	$OCH-CHO-CH_2^{*}+^{*}\rightarrow OCH-CH-CH_2^{*}+O^{*}$	C-O	0.836	2.476
r1244	$OCH-CHO-CH_2^{*}+^{*}\rightarrow OCH-CHO^{*}+CH_2^{*}$	C-C	0.348	1.277
r1245	$OCH-CHO-CH_2^{*}+^{*}\rightarrow OCH-CH_2^{*}+CHO^{*}$	C-C	0.471	1.271
r1246	$OCH-CHO-COH^{*}+^{*}\rightarrow OC-CHO-CHO^{*}+H^{*}$	O-H	0.705	1.228
r1247	$OCH-CHO-COH^{*}+^{*}\rightarrow OC-CHO-COH^{*}+H^{*}$	C-H	0.601	0.932
r1248	$OCH-CHO-COH^{*}+^{*}\rightarrow OCH-CO-COH^{*}+H^{*}$	C-H	0.000	1.000
r1249	$OCH-CHO-COH^{*}+^{*}\rightarrow CH-CHO-COH^{*}+O^{*}$	C-O	1.566	2.264
r1250	$OCH-CHO-COH^{*}+^{*}\rightarrow OCH-CH-COH^{*}+O^{*}$	C-O	0.910	2.505
r1251	$OCH-CHO-COH^{*}+^{*}\rightarrow C-CHO-CHO^{*}+OH^{*}$	C-OH	0.991	1.546
r1252	$OCH-CHO-COH^{*}+^{*}\rightarrow OCH-CHO^{*}+COH^{*}$	C-C	0.115	1.295
r1253	$OCH-CHO-COH^{*}+^{*}\rightarrow OCH-COH^{*}+CHO^{*}$	C-C	0.668	1.269
r1254	$OCH-CHO-CHO^{*}+^{*}\rightarrow OC-CHO-CHO^{*}+H^{*}$	C-H	0.528	0.950
r1255	$OCH-CHO-CHO^{*}+^{*}\rightarrow OCH-CO-CHO^{*}+H^{*}$	C-H	0.000	1.160
r1256	$OCH-CHO-CHO^{*}+^{*}\rightarrow CH-CHO-CHO^{*}+O^{*}$	C-O	1.411	2.358
r1257	$OCH-CHO-CHO^{*}+^{*}\rightarrow OCH-CH-CHO^{*}+O^{*}$	C-O	0.797	2.583
r1258	$OCH-CHO-CHO^{*}+^{*}\rightarrow OCH-CHO^{*}+CHO^{*}$	C-C	0.513	1.281
r1259	$OCH-CH-CH_2OH^{*}+^{*}\rightarrow OCH-CH-CH_2O^{*}+H^{*}$	O-H	0.706	1.252
r1260	$OCH-CH-CH_2OH^{*}+^{*}\rightarrow OC-CH-CH_2OH^{*}+H^{*}$	C-H	0.444	0.950
r1261	$OCH-CH-CH_2OH^{*}+^{*}\rightarrow OCH-C-CH_2OH^{*}+H^{*}$	C-H	0.703	0.922
r1262	$OCH-CH-CH_2OH^{*}+^{*}\rightarrow OCH-CH-CHOH^{*}+H^{*}$	C-H	0.404	0.954
r1263	$OCH-CH-CH_2OH^{*}+^{*}\rightarrow CH-CH-CH_2OH^{*}+O^{*}$	C-O	1.480	2.299
r1264	$OCH-CH-CH_2OH^{*}+^{*}\rightarrow OCH-CH-CH_2^{*}+OH^{*}$	C-OH	0.942	1.543
r1265	$OCH-CH-CH_2OH^{*}+^{*}\rightarrow CH-CHO^{*}+CH_2OH^{*}$	C-C	1.382	1.236
r1266	$OCH-CH-CH_2OH^{*}+^{*}\rightarrow CH-CH_2OH^{*}+CHO^{*}$	C-C	1.435	1.233
r1267	$OCH-CH-CHOH^{*}+^{*}\rightarrow OCH-CH-CHO^{*}+H^{*}$	O-H	0.785	1.232
r1268	$OCH-CH-CHOH^{*}+^{*}\rightarrow OC-CH-CHOH^{*}+H^{*}$	C-H	0.608	0.991
r1269	$OCH-CH-CHOH^{*}+^{*}\rightarrow OCH-C-CHOH^{*}+H^{*}$	C-H	0.887	0.961
r1270	$OCH-CH-CHOH^{*}+^{*}\rightarrow OCH-CH-COH^{*}+H^{*}$	C-H	0.813	0.969
r1271	$OCH-CH-CHOH^{*}+^{*}\rightarrow CH-CH-CHOH^{*}+O^{*}$	C-O	1.900	2.347
r1272	$OCH-CH-CHOH^{*}+^{*}\rightarrow CH-CH-CHO^{*}+OH^{*}$	C-OH	1.114	1.520
r1273	$OCH-CH-CHOH^{*}+^{*}\rightarrow CH-CHO^{*}+CHOH^{*}$	C-C	1.358	1.263
r1274	$OCH-CH-CHOH^{*}+^{*}\rightarrow CH-CHOH^{*}+CHO^{*}$	C-C	1.379	1.262
r1275	$OCH-CH-CH_2O^{*}+^{*}\rightarrow OC-CH-CH_2O^{*}+H^{*}$	C-H	0.591	0.993
r1276	$OCH-CH-CH_2O^{*}+^{*}\rightarrow OCH-C-CH_2O^{*}+H^{*}$	C-H	0.816	0.968
r1277	$OCH-CH-CH_2O^{*}+^{*}\rightarrow OCH-CH-CHO^{*}+H^{*}$	C-H	0.545	0.997
r1278	$OCH-CH-CH_2O^{*}+^{*}\rightarrow CH-CH-CH_2O^{*}+O^{*}$	C-O	1.544	2.476
r1279	$OCH-CH-CH_2O^{*}+^{*}\rightarrow OCH-CH-CH_2^{*}+O^{*}$	C-O	1.544	2.476
r1280	$OCH-CH-CH_2O^{*}+^{*}\rightarrow CH-CHO^{*}+CH_2O^{*}$	C-C	1.392	1.261
r1281	$OCH-CH-CH_2O^{*}+^{*}\rightarrow CH-CH_2O^{*}+CHO^{*}$	C-C	1.742	1.244
r1282	$OCH-CH-CH_2^{*}+^{*}\rightarrow OC-CH-CH_2^{*}+H^{*}$	C-H	0.326	1.027

Supplementary Table 16 – continues from previous page

Tag	Reaction	Type	E_a	E'_a
r1283	OCH−CH−CH_2*+*→OCH−C−CH_2*+H*	C-H	0.428	1.016
r1284	OCH−CH−CH_2*+*→CH−CH−CHO*+H*	C-H	0.637	0.993
r1285	OCH−CH−CH_2*+*→CH−CH−CH_2*+O*	C-O	1.421	2.541
r1286	OCH−CH−CH_2*+*→CH−CHO*+CH_2*	C-C	1.005	1.282
r1287	OCH−CH−CH_2*+*→CH−CH_2*+CHO*	C-C	1.156	1.275
r1288	OCH−CH−COH*+*→OC−CH−CHO*+H*	O-H	0.807	1.729
r1289	OCH−CH−COH*+*→OC−CH−COH*+H*	C-H	0.181	1.054
r1290	OCH−CH−COH*+*→OCH−C−COH*+H*	C-H	0.438	1.026
r1291	OCH−CH−COH*+*→CH−CH−COH*+O*	C-O	1.287	2.629
r1292	OCH−CH−COH*+*→C−CH−CHO*+OH*	C-OH	0.176	1.456
r1293	OCH−CH−COH*+*→CH−CHO*+COH*	C-C	0.727	1.300
r1294	OCH−CH−COH*+*→CH−COH*+CHO*	C-C	1.226	1.276
r1295	OCH−CH−CHO*+*→OC−CH−CHO*+H*	C-H	0.429	1.059
r1296	OCH−CH−CHO*+*→OCH−C−CHO*+H*	C-H	0.555	1.045
r1297	OCH−CH−CHO*+*→CH−CH−CHO*+O*	C-O	1.736	2.572
r1298	OCH−CH−CHO*+*→CH−CHO*+CHO*	C-C	1.315	1.286
r1299	OCH−CO−CH_2OH*+*→OCH−CO−CH_2O*+H*	O-H	0.772	1.041
r1300	OCH−CO−CH_2OH*+*→OC−CO−CH_2OH*+H*	C-H	0.795	0.962
r1301	OCH−CO−CH_2OH*+*→OCH−CO−CHOH*+H*	C-H	0.781	0.963
r1302	OCH−CO−CH_2OH*+*→CH−CO−CH_2OH*+O*	C-O	1.737	2.374
r1303	OCH−CO−CH_2OH*+*→OCH−C−CH_2OH*+O*	C-O	1.739	2.374
r1304	OCH−CO−CH_2OH*+*→OCH−CO−CH_2*+OH*	C-OH	0.953	1.516
r1305	OCH−CO−CH_2OH*+*→OC−CHO*+CH_2OH*	C-C	1.651	1.245
r1306	OCH−CO−CH_2OH*+*→OC−CH_2OH*+CHO*	C-C	1.424	1.255
r1307	OCH−CO−CHOH*+*→OCH−CO−CHO*+H*	O-H	0.798	1.404
r1308	OCH−CO−CHOH*+*→OC−CO−CHOH*+H*	C-H	0.458	1.017
r1309	OCH−CO−CHOH*+*→OCH−CO−COH*+H*	C-H	0.652	0.997
r1310	OCH−CO−CHOH*+*→CH−CO−CHOH*+O*	C-O	1.527	2.518
r1311	OCH−CO−CHOH*+*→OCH−C−CHOH*+O*	C-O	1.624	2.483
r1312	OCH−CO−CHOH*+*→CH−CO−CHO*+OH*	C-OH	0.997	1.508
r1313	OCH−CO−CHOH*+*→OC−CHO*+CHOH*	C-C	1.260	1.272
r1314	OCH−CO−CHOH*+*→OC−CHOH*+CHO*	C-C	1.213	1.274
r1315	OCH−CO−CH_2O*+*→OC−CO−CH_2O*+H*	C-H	0.691	1.002
r1316	OCH−CO−CH_2O*+*→OCH−CO−CHO*+H*	C-H	0.502	1.022
r1317	OCH−CO−CH_2O*+*→CH−CO−CH_2O*+O*	C-O	1.669	2.498
r1318	OCH−CO−CH_2O*+*→OCH−C−CH_2O*+O*	C-O	1.657	2.502
r1319	OCH−CO−CH_2O*+*→OCH−CO−CH_2*+O*	C-O	1.419	2.590
r1320	OCH−CO−CH_2O*+*→OC−CHO*+CH_2O*	C-C	1.384	1.270
r1321	OCH−CO−CH_2O*+*→OC−CH_2O*+CHO*	C-C	1.409	1.269
r1322	OCH−CO−CH_2*+*→OC−CO−CH_2*+H*	C-H	0.638	1.039
r1323	OCH−CO−CH_2*+*→CH−CO−CHO*+H*	C-H	0.882	1.013
r1324	OCH−CO−CH_2*+*→CH−CO−CH_2*+O*	C-O	1.753	2.575
r1325	OCH−CO−CH_2*+*→OCH−C−CH_2*+O*	C-O	1.592	2.634
r1326	OCH−CO−CH_2*+*→OC−CHO*+CH_2*	C-C	1.237	1.291
r1327	OCH−CO−CH_2*+*→OC−CH_2*+CHO*	C-C	1.266	1.289
r1328	OCH−CO−COH*+*→OC−CO−CHO*+H*	O-H	0.848	1.452
r1329	OCH−CO−COH*+*→OC−CO−COH*+H*	C-H	1.122	0.983
r1330	OCH−CO−COH*+*→CH−CO−COH*+O*	C-O	2.034	2.459
r1331	OCH−CO−COH*+*→OCH−C−COH*+O*	C-O	1.479	2.662
r1332	OCH−CO−COH*+*→C−CO−CHO*+OH*	C-OH	1.134	1.496
r1333	OCH−CO−COH*+*→OC−CHO*+COH*	C-C	0.817	1.309
r1334	OCH−CO−COH*+*→OC−COH*+CHO*	C-C	1.180	1.292
r1335	OCH−CO−CHO*+*→OC−CO−CHO*+H*	C-H	0.712	1.055
r1336	OCH−CO−CHO*+*→CH−CO−CHO*+O*	C-O	1.843	2.625
r1337	OCH−CO−CHO*+*→OCH−C−CHO*+O*	C-O	1.598	2.714
r1338	OCH−CO−CHO*+*→OC−CHO*+CHO*	C-C	1.375	1.295
r1339	OCH−C−CH_2OH*+*→OCH−C−CH_2O*+H*	O-H	0.737	1.216
r1340	OCH−C−CH_2OH*+*→OC−C−CH_2OH*+H*	C-H	0.514	0.966
r1341	OCH−C−CH_2OH*+*→OCH−C−CHOH*+H*	C-H	0.555	0.961
r1342	OCH−C−CH_2OH*+*→CH−C−CH_2OH*+O*	C-O	1.316	2.440
r1343	OCH−C−CH_2OH*+*→OCH−C−CH_2*+OH*	C-OH	0.536	1.506
r1344	OCH−C−CH_2OH*+*→C−CHO*+CH_2OH*	C-C	1.505	1.240
r1345	OCH−C−CH_2OH*+*→C−CH_2OH*+CHO*	C-C	1.079	1.260

Supplementary Table 16 – continues from previous page

Tag	Reaction	Type	E_a	E'_a
r1346	OCH−C−CHOH*+*→OCH−C−CHO*+H*	O-H	0.796	1.659
r1347	OCH−C−CHOH*+*→OC−C−CHOH*+H*	C-H	0.826	0.976
r1348	OCH−C−CHOH*+*→OCH−C−COH*+H*	C-H	0.357	1.026
r1349	OCH−C−CHOH*+*→CH−C−CHOH*+O*	C-O	1.478	2.529
r1350	OCH−C−CHOH*+*→CH−C−CHO*+OH*	C-OH	0.796	1.497
r1351	OCH−C−CHOH*+*→C−CHO*+CHOH*	C-C	1.337	1.267
r1352	OCH−C−CHOH*+*→C−CHOH*+CHO*	C-C	0.921	1.287
r1353	OCH−C−CH_2O*+*→OC−C−CH_2O*+H*	C-H	0.811	0.985
r1354	OCH−C−CH_2O*+*→OCH−C−CHO*+H*	C-H	0.255	1.045
r1355	OCH−C−CH_2O*+*→CH−C−CH_2O*+O*	C-O	1.529	2.537
r1356	OCH−C−CH_2O*+*→OCH−C−CH_2*+O*	C-O	1.266	2.634
r1357	OCH−C−CH_2O*+*→C−CHO*+CH_2O*	C-C	1.448	1.265
r1358	OCH−C−CH_2O*+*→C−CH_2O*+CHO*	C-C	1.367	1.269
r1359	OCH−C−CH_2*+*→OC−C−CH_2*+H*	C-H	1.169	1.000
r1360	OCH−C−CH_2*+*→CH−C−CHO*+H*	C-H	0.892	1.029
r1361	OCH−C−CH_2*+*→CH−C−CH_2*+O*	C-O	1.698	2.655
r1362	OCH−C−CH_2*+*→C−CHO*+CH_2*	C-C	1.497	1.286
r1363	OCH−C−CH_2*+*→C−CH_2*+CHO*	C-C	1.231	1.299
r1364	OCH−C−COH*+*→OC−C−CHO*+H*	O-H	0.891	1.174
r1365	OCH−C−COH*+*→OC−C−COH*+H*	C-H	0.972	1.032
r1366	OCH−C−COH*+*→CH−C−COH*+O*	C-O	1.969	2.595
r1367	OCH−C−COH*+*→C−C−CHO*+OH*	C-OH	1.502	1.500
r1368	OCH−C−COH*+*→C−CHO*+COH*	C-C	1.219	1.304
r1369	OCH−C−COH*+*→C−COH*+CHO*	C-C	1.558	1.288
r1370	OCH−C−CHO*+*→OC−C−CHO*+H*	C-H	0.965	1.054
r1371	OCH−C−CHO*+*→CH−C−CHO*+O*	C-O	1.956	2.671
r1372	OCH−C−CHO*+*→C−CHO*+CHO*	C-C	1.709	1.290
r1373	CH−CHOH−CH_2OH*+*→CH−CHO−CH_2OH*+H*	O-H	0.582	0.721
r1374	CH−CHOH−CH_2OH*+*→CH−CHOH−CH_2O*+H*	O-H	0.582	1.255
r1375	CH−CHOH−CH_2OH*+*→C−CHOH−CH_2OH*+H*	C-H	0.147	0.888
r1376	CH−CHOH−CH_2OH*+*→CH−COH−CH_2OH*+H*	C-H	0.176	0.885
r1377	CH−CHOH−CH_2OH*+*→CH−CHOH−CHOH*+H*	C-H	0.731	0.826
r1378	CH−CHOH−CH_2OH*+*→CH−CH−CH_2OH*+OH*	C-OH	0.774	1.583
r1379	CH−CHOH−CH_2OH*+*→CH−CHOH−CH_2*+OH*	C-OH	1.834	1.645
r1380	CH−CHOH−CH_2OH*+*→CH−CHOH*+CH_2OH*	C-C	1.026	1.211
r1381	CH−CHOH−CH_2OH*+*→HOCH−CH_2OH*+CH*	C-C	0.905	1.217
r1382	CH−CHOH−CHOH*+*→CH−CHO−CHOH*+H*	O-H	0.596	1.125
r1383	CH−CHOH−CHOH*+*→CH−CHOH−CHO*+H*	O-H	0.596	1.339
r1384	CH−CHOH−CHOH*+*→C−CHOH−CHOH*+H*	C-H	0.130	0.901
r1385	CH−CHOH−CHOH*+*→CH−COH−CHOH*+H*	C-H	0.147	0.899
r1386	CH−CHOH−CHOH*+*→CH−CHOH−COH*+H*	C-H	0.507	0.860
r1387	CH−CHOH−CHOH*+*→CH−CH−CHOH*+OH*	C-OH	0.679	1.572
r1388	CH−CHOH−CHOH*+*→CH−CHOH−CH*+OH*	C-OH	1.071	1.595
r1389	CH−CHOH−CHOH*+*→CH−CHOH*+CHOH*	C-C	0.547	1.239
r1390	CH−CHOH−CHOH*+*→HOCH−CHOH*+CH*	C-C	0.542	1.239
r1391	CH−CHOH−CH_2O*+*→CH−CHO−CH_2O*+H*	O-H	0.678	0.943
r1392	CH−CHOH−CH_2O*+*→C−CHOH−CH_2O*+H*	C-H	0.411	0.932
r1393	CH−CHOH−CH_2O*+*→CH−COH−CH_2O*+H*	C-H	0.618	0.910
r1394	CH−CHOH−CH_2O*+*→CH−CHOH−CHO*+H*	C-H	0.733	0.898
r1395	CH−CHOH−CH_2O*+*→CH−CHOH−CH_2*+O*	C-O	2.018	2.032
r1396	CH−CHOH−CH_2O*+*→CH−CH−CH_2O*+OH*	C-OH	0.748	1.542
r1397	CH−CHOH−CH_2O*+*→CH−CHOH*+CH_2O*	C-C	1.164	1.237
r1398	CH−CHOH−CH_2O*+*→OCH_2−CHOH*+CH*	C-C	1.004	1.244
r1399	CH−CHOH−CH_2*+*→CH−CHO−CH_2*+H*	O-H	0.555	2.211
r1400	CH−CHOH−CH_2*+*→C−CHOH−CH_2*+H*	C-H	0.000	1.294
r1401	CH−CHOH−CH_2*+*→CH−COH−CH_2*+H*	C-H	0.000	1.576
r1402	CH−CHOH−CH_2*+*→CH−CHOH−CH*+H*	C-H	0.068	0.876
r1403	CH−CHOH−CH_2*+*→CH−CH−CH_2*+OH*	C-OH	0.000	1.899
r1404	CH−CHOH−CH_2*+*→CH−CHOH*+CH_2*	C-C	0.000	1.398
r1405	CH−CHOH−CH_2*+*→CH_2−CHOH*+CH*	C-C	0.000	1.485
r1406	CH−CHOH−COH*+*→CH−CHO−COH*+H*	O-H	0.646	0.874
r1407	CH−CHOH−COH*+*→OC−CHOH−CH*+H*	O-H	0.646	1.310
r1408	CH−CHOH−COH*+*→C−CHOH−COH*+H*	C-H	0.331	0.917

Supplementary Table 16 – continues from previous page

Tag	Reaction	Type	E_a	E'_a
r1409	CH−CHOH−COH*+*→CH−COH−COH*+H*	C-H	0.000	1.286
r1410	CH−CHOH−COH*+*→CH−CH−COH*+OH*	C-OH	0.000	1.589
r1411	CH−CHOH−COH*+*→C−CHOH−CH*+OH*	C-OH	0.921	1.566
r1412	CH−CHOH−COH*+*→CH−CHOH*+COH*	C-C	0.113	1.276
r1413	CH−CHOH−COH*+*→HOC−CHOH*+CH*	C-C	0.374	1.263
r1414	CH−CHOH−CHO*+*→CH−CHO−CHO*+H*	O-H	0.702	0.891
r1415	CH−CHOH−CHO*+*→C−CHOH−CHO*+H*	C-H	0.558	0.934
r1416	CH−CHOH−CHO*+*→CH−COH−CHO*+H*	C-H	0.254	0.967
r1417	CH−CHOH−CHO*+*→OC−CHOH−CH*+H*	C-H	0.651	0.924
r1418	CH−CHOH−CHO*+*→CH−CHOH−CH*+O*	C-O	1.591	2.249
r1419	CH−CHOH−CHO*+*→CH−CH−CHO*+OH*	C-OH	0.535	1.520
r1420	CH−CHOH−CHO*+*→CH−CHOH*+CHO*	C-C	0.800	1.262
r1421	CH−CHOH−CHO*+*→OCH−CHOH*+CH*	C-C	0.526	1.275
r1422	CH−CHOH−CH*+*→CH−CHO−CH*+H*	O-H	0.671	1.141
r1423	CH−CHOH−CH*+*→C−CHOH−CH*+H*	C-H	0.449	0.923
r1424	CH−CHOH−CH*+*→CH−COH−CH*+H*	C-H	0.000	1.334
r1425	CH−CHOH−CH*+*→CH−CH−CH*+OH*	C-OH	0.817	1.550
r1426	CH−CHOH−CH*+*→CH−CHOH*+CH*	C-C	0.102	1.284
r1427	CH−COH−CH_2OH*+*→CH−CO−CH_2OH*+H*	O-H	0.683	1.062
r1428	CH−COH−CH_2OH*+*→CH−COH−CH_2O*+H*	O-H	0.683	0.940
r1429	CH−COH−CH_2OH*+*→C−COH−CH_2OH*+H*	C-H	0.518	0.925
r1430	CH−COH−CH_2OH*+*→CH−COH−CHOH*+H*	C-H	0.761	0.899
r1431	CH−COH−CH_2OH*+*→CH−C−CH_2OH*+OH*	C-OH	0.927	1.551
r1432	CH−COH−CH_2OH*+*→CH−COH−CH_2*+OH*	C-OH	0.870	1.547
r1433	CH−COH−CH_2OH*+*→CH−COH*+CH_2OH*	C-C	1.427	1.226
r1434	CH−COH−CH_2OH*+*→HOC−CH_2OH*+CH*	C-C	1.100	1.241
r1435	CH−COH−CHOH*+*→CH−CO−CHOH*+H*	O-H	0.703	1.481
r1436	CH−COH−CHOH*+*→CH−COH−CHO*+H*	O-H	0.703	1.408
r1437	CH−COH−CHOH*+*→C−COH−CHOH*+H*	C-H	0.244	0.969
r1438	CH−COH−CHOH*+*→CH−COH−COH*+H*	C-H	0.098	0.985
r1439	CH−COH−CHOH*+*→CH−C−CHOH*+OH*	C-OH	0.711	1.530
r1440	CH−COH−CHOH*+*→CH−COH−CH*+OH*	C-OH	0.406	1.512
r1441	CH−COH−CHOH*+*→CH−COH*+CHOH*	C-C	0.991	1.253
r1442	CH−COH−CHOH*+*→HOC−CHOH*+CH*	C-C	0.772	1.263
r1443	CH−COH−CH_2O*+*→CH−CO−CH_2O*+H*	O-H	0.720	1.302
r1444	CH−COH−CH_2O*+*→C−COH−CH_2O*+H*	C-H	0.201	0.987
r1445	CH−COH−CH_2O*+*→CH−COH−CHO*+H*	C-H	0.382	0.967
r1446	CH−COH−CH_2O*+*→CH−COH−CH_2*+O*	C-O	1.157	2.454
r1447	CH−COH−CH_2O*+*→CH−C−CH_2O*+OH*	C-OH	0.798	1.528
r1448	CH−COH−CH_2O*+*→CH−COH*+CH_2O*	C-C	1.148	1.251
r1449	CH−COH−CH_2O*+*→HOC−CH_2O*+CH*	C-C	0.796	1.268
r1450	CH−COH−CH_2*+*→CH−CO−CH_2*+H*	O-H	0.780	1.228
r1451	CH−COH−CH_2*+*→C−COH−CH_2*+H*	C-H	0.424	1.008
r1452	CH−COH−CH_2*+*→CH−COH−CH*+H*	C-H	0.440	1.006
r1453	CH−COH−CH_2*+*→CH−C−CH_2*+OH*	C-OH	0.752	1.501
r1454	CH−COH−CH_2*+*→CH−COH*+CH_2*	C-C	1.127	1.272
r1455	CH−COH−CH_2*+*→HOC−CH_2*+CH*	C-C	0.912	1.282
r1456	CH−COH−COH*+*→CH−CO−COH*+H*	O-H	0.830	0.499
r1457	CH−COH−COH*+*→OC−COH−CH*+H*	O-H	0.830	0.506
r1458	CH−COH−COH*+*→C−COH−COH*+H*	C-H	0.567	1.030
r1459	CH−COH−COH*+*→CH−C−COH*+OH*	C-OH	1.339	1.515
r1460	CH−COH−COH*+*→C−COH−CH*+OH*	C-OH	0.884	1.488
r1461	CH−COH−COH*+*→CH−COH*+COH*	C-C	1.091	1.290
r1462	CH−COH−COH*+*→HOC−COH*+CH*	C-C	1.021	1.294
r1463	CH−COH−CHO*+*→CH−CO−CHO*+H*	O-H	0.804	1.273
r1464	CH−COH−CHO*+*→C−COH−CHO*+H*	C-H	0.323	1.036
r1465	CH−COH−CHO*+*→OC−COH−CH*+H*	C-H	1.095	0.954
r1466	CH−COH−CHO*+*→CH−COH−CH*+O*	C-O	1.328	2.606
r1467	CH−COH−CHO*+*→CH−C−CHO*+OH*	C-OH	0.856	1.497
r1468	CH−COH−CHO*+*→CH−COH*+CHO*	C-C	1.205	1.276
r1469	CH−COH−CHO*+*→OCH−COH*+CH*	C-C	0.886	1.291
r1470	CH−COH−CH*+*→CH−CO−CH*+H*	O-H	0.861	0.554
r1471	CH−COH−CH*+*→C−COH−CH*+H*	C-H	0.658	1.044

Supplementary Table 16 – continues from previous page

Tag	Reaction	Type	E_a	E'_a
r1472	CH−COH−CH*+*→CH−C−CH*+OH*	C-OH	1.248	1.497
r1473	CH−COH−CH*+*→CH−COH*+CH*	C-C	1.127	1.299
r1474	CH−CHO−CH_2OH*+*→CH−CHO−CH_2O*+H*	O-H	0.602	1.400
r1475	CH−CHO−CH_2OH*+*→C−CHO−CH_2OH*+H*	C-H	0.260	0.891
r1476	CH−CHO−CH_2OH*+*→CH−CO−CH_2OH*+H*	C-H	0.000	0.949
r1477	CH−CHO−CH_2OH*+*→CH−CHO−CHOH*+H*	C-H	0.392	0.877
r1478	CH−CHO−CH_2OH*+*→CH−CH−CH_2OH*+O*	C-O	0.752	2.299
r1479	CH−CHO−CH_2OH*+*→CH−CHO−CH_2*+OH*	C-OH	0.214	1.543
r1480	CH−CHO−CH_2OH*+*→CH−CHO*+CH_2OH*	C-C	0.654	1.236
r1481	CH−CHO−CH_2OH*+*→OCH−CH_2OH*+CH*	C-C	0.363	1.249
r1482	CH−CHO−CHOH*+*→CH−CHO−CHO*+H*	O-H	0.671	1.074
r1483	CH−CHO−CHOH*+*→C−CHO−CHOH*+H*	C-H	0.500	0.918
r1484	CH−CHO−CHOH*+*→CH−CO−CHOH*+H*	C-H	0.000	1.000
r1485	CH−CHO−CHOH*+*→CH−CHO−COH*+H*	C-H	0.831	0.882
r1486	CH−CHO−CHOH*+*→CH−CH−CHOH*+O*	C-O	1.107	2.347
r1487	CH−CHO−CHOH*+*→CH−CHO−CH*+OH*	C-OH	1.101	1.566
r1488	CH−CHO−CHOH*+*→CH−CHO*+CHOH*	C-C	0.566	1.263
r1489	CH−CHO−CHOH*+*→OCH−CHOH*+CH*	C-C	0.312	1.275
r1490	CH−CHO−CH_2O*+*→C−CHO−CH_2O*+H*	C-H	0.385	0.964
r1491	CH−CHO−CH_2O*+*→CH−CO−CH_2O*+H*	C-H	0.357	0.967
r1492	CH−CHO−CH_2O*+*→CH−CHO−CHO*+H*	C-H	0.827	0.916
r1493	CH−CHO−CH_2O*+*→CH−CH−CH_2O*+O*	C-O	1.069	2.476
r1494	CH−CHO−CH_2O*+*→CH−CHO−CH_2*+O*	C-O	1.070	2.476
r1495	CH−CHO−CH_2O*+*→CH−CHO*+CH_2O*	C-C	0.917	1.261
r1496	CH−CHO−CH_2O*+*→OCH−CH_2O*+CH*	C-C	0.789	1.267
r1497	CH−CHO−CH_2*+*→C−CHO−CH_2*+H*	C-H	1.347	0.917
r1498	CH−CHO−CH_2*+*→CH−CO−CH_2*+H*	C-H	0.627	0.995
r1499	CH−CHO−CH_2*+*→CH−CHO−CH*+H*	C-H	1.300	0.922
r1500	CH−CHO−CH_2*+*→CH−CH−CH_2*+O*	C-O	1.421	2.541
r1501	CH−CHO−CH_2*+*→CH−CHO*+CH_2*	C-C	1.005	1.282
r1502	CH−CHO−CH_2*+*→OCH−CH_2*+CH*	C-C	0.755	1.293
r1503	CH−CHO−COH*+*→OC−CHO−CH*+H*	O-H	0.679	1.160
r1504	CH−CHO−COH*+*→C−CHO−COH*+H*	C-H	0.577	0.915
r1505	CH−CHO−COH*+*→CH−CO−COH*+H*	C-H	0.225	0.953
r1506	CH−CHO−COH*+*→CH−CH−COH*+O*	C-O	0.390	2.629
r1507	CH−CHO−COH*+*→C−CHO−CH*+OH*	C-OH	1.007	1.557
r1508	CH−CHO−COH*+*→CH−CHO*+COH*	C-C	0.000	1.470
r1509	CH−CHO−COH*+*→OCH−COH*+CH*	C-C	0.010	1.291
r1510	CH−CHO−CHO*+*→C−CHO−CHO*+H*	C-H	0.571	0.953
r1511	CH−CHO−CHO*+*→CH−CO−CHO*+H*	C-H	0.019	1.013
r1512	CH−CHO−CHO*+*→OC−CHO−CH*+H*	C-H	0.799	0.929
r1513	CH−CHO−CHO*+*→CH−CH−CHO*+O*	C-O	0.898	2.572
r1514	CH−CHO−CHO*+*→CH−CHO−CH*+O*	C-O	1.436	2.375
r1515	CH−CHO−CHO*+*→CH−CHO*+CHO*	C-C	0.477	1.286
r1516	CH−CHO−CHO*+*→OCH−CHO*+CH*	C-C	0.105	1.303
r1517	CH−CHO−CH*+*→C−CHO−CH*+H*	C-H	0.799	0.936
r1518	CH−CHO−CH*+*→CH−CO−CH*+H*	C-H	0.420	0.976
r1519	CH−CHO−CH*+*→CH−CH−CH*+O*	C-O	1.306	2.445
r1520	CH−CHO−CH*+*→CH−CHO*+CH*	C-C	0.060	1.308
r1521	CH−CH−CH_2OH*+*→CH−CH−CH_2O*+H*	O-H	0.698	1.356
r1522	CH−CH−CH_2OH*+*→C−CH−CH_2OH*+H*	C-H	0.591	0.928
r1523	CH−CH−CH_2OH*+*→CH−C−CH_2OH*+H*	C-H	0.422	0.946
r1524	CH−CH−CH_2OH*+*→CH−CH−CHOH*+H*	C-H	0.734	0.912
r1525	CH−CH−CH_2OH*+*→CH−CH−CH_2*+OH*	C-OH	0.627	1.528
r1526	CH−CH−CH_2OH*+*→CH−CH*+CH_2OH*	C-C	0.962	1.253
r1527	CH−CH−CH_2OH*+*→CH−CH_2OH*+CH*	C-C	0.899	1.256
r1528	CH−CH−CHOH*+*→CH−CH−CHO*+H*	O-H	0.723	1.559
r1529	CH−CH−CHOH*+*→C−CH−CHOH*+H*	C-H	0.246	0.984
r1530	CH−CH−CHOH*+*→CH−C−CHOH*+H*	C-H	0.300	0.978
r1531	CH−CH−CHOH*+*→CH−CH−COH*+H*	C-H	0.000	1.050
r1532	CH−CH−CHOH*+*→CH−CH−CH*+OH*	C-OH	1.186	1.550
r1533	CH−CH−CHOH*+*→CH−CH*+CHOH*	C-C	0.566	1.280
r1534	CH−CH−CHOH*+*→CH−CHOH*+CH*	C-C	0.471	1.284

Supplementary Table 16 – continues from previous page

Tag	Reaction	Type	E_a	E'_a
r1535	CH−CH−CH_2O*+*→C−CH−CH_2O*+H*	C-H	0.457	1.013
r1536	CH−CH−CH_2O*+*→CH−C−CH_2O*+H*	C-H	0.753	0.981
r1537	CH−CH−CH_2O*+*→CH−CH−CHO*+H*	C-H	0.638	0.993
r1538	CH−CH−CH_2O*+*→CH−CH−CH_2*+O*	C-O	1.422	2.541
r1539	CH−CH−CH_2O*+*→CH−CH*+CH_2O*	C-C	1.084	1.278
r1540	CH−CH−CH_2O*+*→CH−CH_2O*+CH*	C-C	1.318	1.267
r1541	CH−CH−CH_2*+*→C−CH−CH_2*+H*	C-H	0.776	1.004
r1542	CH−CH−CH_2*+*→CH−C−CH_2*+H*	C-H	0.598	1.024
r1543	CH−CH−CH_2*+*→CH−CH−CH*+H*	C-H	1.306	0.948
r1544	CH−CH−CH_2*+*→CH−CH*+CH_2*	C-C	0.885	1.299
r1545	CH−CH−CH_2*+*→CH−CH_2*+CH*	C-C	0.920	1.297
r1546	CH−CH−COH*+*→OC−CH−CH*+H*	O-H	0.874	1.508
r1547	CH−CH−COH*+*→C−CH−COH*+H*	C-H	0.657	1.053
r1548	CH−CH−COH*+*→CH−C−COH*+H*	C-H	1.129	1.002
r1549	CH−CH−COH*+*→C−CH−CH*+OH*	C-OH	1.341	1.497
r1550	CH−CH−COH*+*→CH−CH*+COH*	C-C	0.828	1.317
r1551	CH−CH−COH*+*→CH−COH*+CH*	C-C	1.211	1.299
r1552	CH−CH−CHO*+*→C−CH−CHO*+H*	C-H	0.064	1.093
r1553	CH−CH−CHO*+*→CH−C−CHO*+H*	C-H	0.660	1.029
r1554	CH−CH−CHO*+*→OC−CH−CH*+H*	C-H	0.228	1.076
r1555	CH−CH−CHO*+*→CH−CH−CH*+O*	C-O	2.040	2.445
r1556	CH−CH−CHO*+*→CH−CH*+CHO*	C-C	0.911	1.303
r1557	CH−CH−CHO*+*→CH−CHO*+CH*	C-C	0.795	1.308
r1558	CH−CH−CH*+*→C−CH−CH*+H*	C-H	0.187	1.029
r1559	CH−CH−CH*+*→CH−C−CH*+H*	C-H	0.179	1.030
r1560	CH−CH−CH*+*→CH−CH*+CH*	C-C	0.000	1.366
r1561	CH−CO−CH_2OH*+*→CH−CO−CH_2O*+H*	O-H	0.738	1.198
r1562	CH−CO−CH_2OH*+*→C−CO−CH_2OH*+H*	C-H	0.691	0.947
r1563	CH−CO−CH_2OH*+*→CH−CO−CHOH*+H*	C-H	0.438	0.974
r1564	CH−CO−CH_2OH*+*→CH−C−CH_2OH*+O*	C-O	1.318	2.440
r1565	CH−CO−CH_2OH*+*→CH−CO−CH_2*+OH*	C-OH	0.773	1.520
r1566	CH−CO−CH_2OH*+*→OC−CH*+CH_2OH*	C-C	1.052	1.262
r1567	CH−CO−CH_2OH*+*→OC−CH_2OH*+CH*	C-C	0.706	1.278
r1568	CH−CO−CHOH*+*→CH−CO−CHO*+H*	O-H	0.815	1.210
r1569	CH−CO−CHOH*+*→C−CO−CHOH*+H*	C-H	0.900	0.982
r1570	CH−CO−CHOH*+*→CH−CO−COH*+H*	C-H	1.174	0.953
r1571	CH−CO−CHOH*+*→CH−C−CHOH*+O*	C-O	1.611	2.529
r1572	CH−CO−CHOH*+*→CH−CO−CH*+OH*	C-OH	1.510	1.531
r1573	CH−CO−CHOH*+*→OC−CH*+CHOH*	C-C	1.014	1.289
r1574	CH−CO−CHOH*+*→OC−CHOH*+CH*	C-C	0.849	1.297
r1575	CH−CO−CH_2O*+*→C−CO−CH_2O*+H*	C-H	0.803	0.984
r1576	CH−CO−CH_2O*+*→CH−CO−CHO*+H*	C-H	0.540	1.013
r1577	CH−CO−CH_2O*+*→CH−C−CH_2O*+O*	C-O	1.513	2.537
r1578	CH−CO−CH_2O*+*→CH−CO−CH_2*+O*	C-O	1.411	2.575
r1579	CH−CO−CH_2O*+*→OC−CH*+CH_2O*	C-C	0.976	1.287
r1580	CH−CO−CH_2O*+*→OC−CH_2O*+CH*	C-C	0.882	1.291
r1581	CH−CO−CH_2*+*→C−CO−CH_2*+H*	C-H	0.679	1.028
r1582	CH−CO−CH_2*+*→CH−CO−CH*+H*	C-H	1.166	0.976
r1583	CH−CO−CH_2*+*→CH−C−CH_2*+O*	C-O	1.478	2.655
r1584	CH−CO−CH_2*+*→OC−CH*+CH_2*	C-C	0.821	1.308
r1585	CH−CO−CH_2*+*→OC−CH_2*+CH*	C-C	0.731	1.312
r1586	CH−CO−COH*+*→OC−CO−CH*+H*	O-H	0.783	1.309
r1587	CH−CO−COH*+*→C−CO−COH*+H*	C-H	0.240	1.029
r1588	CH−CO−COH*+*→CH−C−COH*+O*	C-O	1.211	2.595
r1589	CH−CO−COH*+*→C−CO−CH*+OH*	C-OH	1.291	1.531
r1590	CH−CO−COH*+*→OC−CH*+COH*	C-C	0.006	1.326
r1591	CH−CO−COH*+*→OC−COH*+CH*	C-C	0.250	1.314
r1592	CH−CO−CHO*+*→C−CO−CHO*+H*	C-H	0.836	1.032
r1593	CH−CO−CHO*+*→OC−CO−CH*+H*	C-H	1.095	1.004
r1594	CH−CO−CHO*+*→CH−C−CHO*+O*	C-O	1.622	2.671
r1595	CH−CO−CHO*+*→CH−CO−CH*+O*	C-O	2.022	2.524
r1596	CH−CO−CHO*+*→OC−CH*+CHO*	C-C	0.920	1.312
r1597	CH−CO−CHO*+*→OC−CHO*+CH*	C-C	0.801	1.317

Supplementary Table 16 – continues from previous page

Tag	Reaction	Type	E_a	E'_a
r1598	CH−CO−CH*+*→C−CO−CH*+H*	C-H	0.978	0.976
r1599	CH−CO−CH*+*→CH−C−CH*+O*	C-O	1.240	2.673
r1600	CH−CO−CH*+*→OC−CH*+CH*	C-C	0.066	1.334
r1601	CH−C−CH_2OH*+*→CH−C−CH_2O*+H*	O-H	0.773	1.136
r1602	CH−C−CH_2OH*+*→C−C−CH_2OH*+H*	C-H	0.783	0.963
r1603	CH−C−CH_2OH*+*→CH−C−CHOH*+H*	C-H	0.645	0.978
r1604	CH−C−CH_2OH*+*→CH−C−CH_2*+OH*	C-OH	0.698	1.501
r1605	CH−C−CH_2OH*+*→C−CH*+CH_2OH*	C-C	1.336	1.260
r1606	CH−C−CH_2OH*+*→C−CH_2OH*+CH*	C-C	0.847	1.283
r1607	CH−C−CHOH*+*→CH−C−CHO*+H*	O-H	0.820	1.347
r1608	CH−C−CHOH*+*→C−C−CHOH*+H*	C-H	0.872	0.990
r1609	CH−C−CHOH*+*→CH−C−COH*+H*	C-H	0.758	1.002
r1610	CH−C−CHOH*+*→CH−C−CH*+OH*	C-OH	0.961	1.497
r1611	CH−C−CHOH*+*→C−CH*+CHOH*	C-C	1.095	1.287
r1612	CH−C−CHOH*+*→C−CHOH*+CH*	C-C	0.616	1.309
r1613	CH−C−CH_2O*+*→C−C−CH_2O*+H*	C-H	0.883	0.992
r1614	CH−C−CH_2O*+*→CH−C−CHO*+H*	C-H	0.533	1.029
r1615	CH−C−CH_2O*+*→CH−C−CH_2*+O*	C-O	1.339	2.655
r1616	CH−C−CH_2O*+*→C−CH*+CH_2O*	C-C	1.163	1.285
r1617	CH−C−CH_2O*+*→C−CH_2O*+CH*	C-C	1.019	1.292
r1618	CH−C−CH_2*+*→C−C−CH_2*+H*	C-H	1.173	1.008
r1619	CH−C−CH_2*+*→CH−C−CH*+H*	C-H	0.964	1.030
r1620	CH−C−CH_2*+*→C−CH*+CH_2*	C-C	1.161	1.306
r1621	CH−C−CH_2*+*→C−CH_2*+CH*	C-C	0.832	1.321
r1622	CH−C−COH*+*→OC−C−CH*+H*	O-H	0.856	1.221
r1623	CH−C−COH*+*→C−C−COH*+H*	C-H	0.912	1.012
r1624	CH−C−COH*+*→C−C−CH*+OH*	C-OH	1.177	1.495
r1625	CH−C−COH*+*→C−CH*+COH*	C-C	0.552	1.324
r1626	CH−C−COH*+*→C−COH*+CH*	C-C	0.828	1.311
r1627	CH−C−CHO*+*→C−C−CHO*+H*	C-H	1.061	1.026
r1628	CH−C−CHO*+*→OC−C−CH*+H*	C-H	0.954	1.037
r1629	CH−C−CHO*+*→CH−C−CH*+O*	C-O	1.787	2.673
r1630	CH−C−CHO*+*→C−CH*+CHO*	C-C	1.131	1.310
r1631	CH−C−CHO*+*→C−CHO*+CH*	C-C	1.068	1.313
r1632	CH−C−CH*+*→C−C−CH*+H*	C-H	1.006	1.033
r1633	CH−C−CH*+*→C−CH*+CH*	C-C	0.661	1.333
r1634	OC−CHOH−CH_2OH*+*→OC−CHO−CH_2OH*+H*	O-H	0.654	0.988
r1635	OC−CHOH−CH_2OH*+*→OC−CHOH−CH_2O*+H*	O-H	0.654	1.311
r1636	OC−CHOH−CH_2OH*+*→OC−COH−CH_2OH*+H*	C-H	0.367	0.919
r1637	OC−CHOH−CH_2OH*+*→OC−CHOH−CHOH*+H*	C-H	0.594	0.895
r1638	OC−CHOH−CH_2OH*+*→C−CHOH−CH_2OH*+O*	C-O	1.167	2.281
r1639	OC−CHOH−CH_2OH*+*→OC−CH−CH_2OH*+OH*	C-OH	0.680	1.548
r1640	OC−CHOH−CH_2OH*+*→OC−CHOH−CH_2*+OH*	C-OH	0.927	1.563
r1641	OC−CHOH−CH_2OH*+*→OC−CHOH*+CH_2OH*	C-C	1.272	1.224
r1642	OC−CHOH−CH_2OH*+*→HOCH−CH_2OH*+CO*	C-C	0.764	1.248
r1643	OC−CHOH−CHOH*+*→OC−CHO−CHOH*+H*	O-H	0.697	1.186
r1644	OC−CHOH−CHOH*+*→OC−CHOH−CHO*+H*	O-H	0.697	1.248
r1645	OC−CHOH−CHOH*+*→OC−COH−CHOH*+H*	C-H	0.431	0.945
r1646	OC−CHOH−CHOH*+*→OC−CHOH−COH*+H*	C-H	0.835	0.901
r1647	OC−CHOH−CHOH*+*→C−CHOH−CHOH*+O*	C-O	1.377	2.315
r1648	OC−CHOH−CHOH*+*→OC−CH−CHOH*+OH*	C-OH	0.526	1.522
r1649	OC−CHOH−CHOH*+*→OC−CHOH−CH*+OH*	C-OH	1.257	1.564
r1650	OC−CHOH−CHOH*+*→OC−CHOH*+CHOH*	C-C	0.998	1.251
r1651	OC−CHOH−CHOH*+*→HOCH−CHOH*+CO*	C-C	0.607	1.269
r1652	OC−CHOH−CH_2O*+*→OC−CHO−CH_2O*+H*	O-H	0.748	0.893
r1653	OC−CHOH−CH_2O*+*→OC−COH−CH_2O*+H*	C-H	0.557	0.969
r1654	OC−CHOH−CH_2O*+*→OC−CHOH−CHO*+H*	C-H	0.753	0.948
r1655	OC−CHOH−CH_2O*+*→C−CHOH−CH_2O*+O*	C-O	1.492	2.403
r1656	OC−CHOH−CH_2O*+*→OC−CHOH−CH_2*+O*	C-O	1.532	2.388
r1657	OC−CHOH−CH_2O*+*→OC−CH−CH_2O*+OH*	C-OH	0.867	1.521
r1658	OC−CHOH−CH_2O*+*→OC−CHOH*+CH_2O*	C-C	1.392	1.249
r1659	OC−CHOH−CH_2O*+*→OCH_2−CHOH*+CO*	C-C	0.846	1.275
r1660	OC−CHOH−CH_2*+*→OC−CHO−CH_2*+H*	O-H	0.745	1.005

Supplementary Table 16 – continues from previous page

Tag	Reaction	Type	E_a	E'_a
r1661	OC−CHOH−CH_2*+*→OC−COH−CH_2*+H*	C-H	0.190	1.006
r1662	OC−CHOH−CH_2*+*→OC−CHOH−CH*+H*	C-H	0.952	0.924
r1663	OC−CHOH−CH_2*+*→C−CHOH−CH_2*+O*	C-O	1.536	2.379
r1664	OC−CHOH−CH_2*+*→OC−CH−CH_2*+OH*	C-OH	0.470	1.499
r1665	OC−CHOH−CH_2*+*→OC−CHOH*+CH_2*	C-C	0.930	1.270
r1666	OC−CHOH−CH_2*+*→CH_2−CHOH*+CO*	C-C	0.461	1.292
r1667	OC−CHOH−COH*+*→OC−CHO−COH*+H*	O-H	0.707	1.022
r1668	OC−CHOH−COH*+*→OC−CHOH−CO*+H*	O-H	0.707	1.452
r1669	OC−CHOH−COH*+*→OC−COH−COH*+H*	C-H	0.469	0.948
r1670	OC−CHOH−COH*+*→C−CHOH−COH*+O*	C-O	1.319	2.360
r1671	OC−CHOH−COH*+*→OC−CH−COH*+OH*	C-OH	0.000	1.576
r1672	OC−CHOH−COH*+*→C−CHOH−CO*+OH*	C-OH	0.719	1.529
r1673	OC−CHOH−COH*+*→OC−CHOH*+COH*	C-C	0.277	1.288
r1674	OC−CHOH−COH*+*→HOC−CHOH*+CO*	C-C	0.151	1.294
r1675	OC−CHOH−CHO*+*→OC−CHO−CHO*+H*	O-H	0.776	0.799
r1676	OC−CHOH−CHO*+*→OC−COH−CHO*+H*	C-H	0.218	1.027
r1677	OC−CHOH−CHO*+*→OC−CHOH−CO*+H*	C-H	0.713	0.974
r1678	OC−CHOH−CHO*+*→C−CHOH−CHO*+O*	C-O	1.673	2.409
r1679	OC−CHOH−CHO*+*→OC−CHOH−CH*+O*	C-O	1.748	2.381
r1680	OC−CHOH−CHO*+*→OC−CH−CHO*+OH*	C-OH	0.340	1.479
r1681	OC−CHOH−CHO*+*→OC−CHOH*+CHO*	C-C	1.059	1.274
r1682	OC−CHOH−CHO*+*→OCH−CHOH*+CO*	C-C	0.398	1.305
r1683	OC−CHOH−CH*+*→OC−CHO−CH*+H*	O-H	0.741	0.787
r1684	OC−CHOH−CH*+*→OC−COH−CH*+H*	C-H	0.655	0.954
r1685	OC−CHOH−CH*+*→C−CHOH−CO*+H*	C-H	0.411	0.980
r1686	OC−CHOH−CH*+*→C−CHOH−CH*+O*	C-O	1.518	2.376
r1687	OC−CHOH−CH*+*→OC−CH−CH*+OH*	C-OH	0.000	1.560
r1688	OC−CHOH−CH*+*→OC−CHOH*+CH*	C-C	0.336	1.297
r1689	OC−CHOH−CH*+*→CH−CHOH*+CO*	C-C	0.000	1.365
r1690	OC−CHOH−CO*+*→OC−CHO−CO*+H*	O-H	0.814	0.853
r1691	OC−CHOH−CO*+*→OC−COH−CO*+H*	C-H	0.000	1.099
r1692	OC−CHOH−CO*+*→C−CHOH−CO*+O*	C-O	1.592	2.534
r1693	OC−CHOH−CO*+*→OC−CH−CO*+OH*	C-OH	0.039	1.446
r1694	OC−CHOH−CO*+*→OC−CHOH*+CO*	C-C	0.195	1.327
r1695	OC−COH−CH_2OH*+*→OC−CO−CH_2OH*+H*	O-H	0.733	1.171
r1696	OC−COH−CH_2OH*+*→OC−COH−CH_2O*+H*	O-H	0.733	1.250
r1697	OC−COH−CH_2OH*+*→OC−COH−CHOH*+H*	C-H	0.682	0.945
r1698	OC−COH−CH_2OH*+*→C−COH−CH_2OH*+O*	C-O	1.444	2.382
r1699	OC−COH−CH_2OH*+*→OC−C−CH_2OH*+OH*	C-OH	1.057	1.538
r1700	OC−COH−CH_2OH*+*→OC−COH−CH_2*+OH*	C-OH	0.612	1.512
r1701	OC−COH−CH_2OH*+*→OC−COH*+CH_2OH*	C-C	1.446	1.242
r1702	OC−COH−CH_2OH*+*→HOC−CH_2OH*+CO*	C-C	0.802	1.272
r1703	OC−COH−CHOH*+*→OC−CO−CHOH*+H*	O-H	0.771	1.521
r1704	OC−COH−CHOH*+*→OC−COH−CHO*+H*	O-H	0.771	1.617
r1705	OC−COH−CHOH*+*→OC−COH−COH*+H*	C-H	0.917	0.948
r1706	OC−COH−CHOH*+*→C−COH−CHOH*+O*	C-O	1.374	2.505
r1707	OC−COH−CHOH*+*→OC−C−CHOH*+OH*	C-OH	1.209	1.532
r1708	OC−COH−CHOH*+*→OC−COH−CH*+OH*	C-OH	1.453	1.546
r1709	OC−COH−CHOH*+*→OC−COH*+CHOH*	C-C	1.135	1.269
r1710	OC−COH−CHOH*+*→HOC−CHOH*+CO*	C-C	0.599	1.294
r1711	OC−COH−CH_2O*+*→OC−CO−CH_2O*+H*	O-H	0.807	1.141
r1712	OC−COH−CH_2O*+*→OC−COH−CHO*+H*	C-H	0.436	1.027
r1713	OC−COH−CH_2O*+*→C−COH−CH_2O*+O*	C-O	1.497	2.553
r1714	OC−COH−CH_2O*+*→OC−COH−CH_2*+O*	C-O	1.347	2.607
r1715	OC−COH−CH_2O*+*→OC−C−CH_2O*+OH*	C-OH	1.359	1.525
r1716	OC−COH−CH_2O*+*→OC−COH*+CH_2O*	C-C	1.428	1.267
r1717	OC−COH−CH_2O*+*→HOC−CH_2O*+CO*	C-C	0.758	1.298
r1718	OC−COH−CH_2*+*→OC−CO−CH_2*+H*	O-H	0.862	1.197
r1719	OC−COH−CH_2*+*→OC−COH−CH*+H*	C-H	1.499	0.954
r1720	OC−COH−CH_2*+*→C−COH−CH_2*+O*	C-O	1.720	2.611
r1721	OC−COH−CH_2*+*→OC−C−CH_2*+OH*	C-OH	1.587	1.516
r1722	OC−COH−CH_2*+*→OC−COH*+CH_2*	C-C	1.369	1.288
r1723	OC−COH−CH_2*+*→HOC−CH_2*+CO*	C-C	0.836	1.313

Supplementary Table 16 – continues from previous page

Tag	Reaction	Type	E_a	E'_a
r1724	OC−COH−COH*+*→OC−CO−COH*+H*	O-H	0.775	1.142
r1725	OC−COH−COH*+*→OC−COH−CO*+H*	O-H	0.775	2.142
r1726	OC−COH−COH*+*→C−COH−COH*+O*	C-O	0.947	2.672
r1727	OC−COH−COH*+*→OC−C−COH*+OH*	C-OH	0.624	1.496
r1728	OC−COH−COH*+*→C−COH−CO*+OH*	C-OH	0.134	1.467
r1729	OC−COH−COH*+*→OC−COH*+COH*	C-C	0.378	1.306
r1730	OC−COH−COH*+*→HOC−COH*+CO*	C-C	0.000	1.333
r1731	OC−COH−CHO*+*→OC−CO−CHO*+H*	O-H	0.892	1.186
r1732	OC−COH−CHO*+*→OC−COH−CO*+H*	C-H	0.529	1.080
r1733	OC−COH−CHO*+*→C−COH−CHO*+O*	C-O	1.713	2.691
r1734	OC−COH−CHO*+*→OC−COH−CH*+O*	C-O	2.338	2.461
r1735	OC−COH−CHO*+*→OC−C−CHO*+OH*	C-OH	1.202	1.482
r1736	OC−COH−CHO*+*→OC−COH*+CHO*	C-C	1.490	1.292
r1737	OC−COH−CHO*+*→OCH−COH*+CO*	C-C	0.853	1.322
r1738	OC−COH−CH*+*→OC−CO−CH*+H*	O-H	0.784	1.303
r1739	OC−COH−CH*+*→C−COH−CO*+H*	C-H	0.000	1.272
r1740	OC−COH−CH*+*→C−COH−CH*+O*	C-O	0.905	2.710
r1741	OC−COH−CH*+*→OC−C−CH*+OH*	C-OH	0.627	1.492
r1742	OC−COH−CH*+*→OC−COH*+CH*	C-C	0.257	1.314
r1743	OC−COH−CH*+*→CH−COH*+CO*	C-C	0.000	1.390
r1744	OC−COH−CO*+*→OC−CO−CO*+H*	O-H	0.971	0.647
r1745	OC−COH−CO*+*→C−COH−CO*+O*	C-O	1.959	2.802
r1746	OC−COH−CO*+*→OC−C−CO*+OH*	C-OH	1.663	1.476
r1747	OC−COH−CO*+*→OC−COH*+CO*	C-C	0.917	1.345
r1748	OC−CHO−CH_2OH*+*→OC−CHO−CH_2O*+H*	O-H	0.702	1.169
r1749	OC−CHO−CH_2OH*+*→OC−CO−CH_2OH*+H*	C-H	0.306	0.962
r1750	OC−CHO−CH_2OH*+*→OC−CHO−CHOH*+H*	C-H	0.487	0.942
r1751	OC−CHO−CH_2OH*+*→C−CHO−CH_2OH*+O*	C-O	1.480	2.289
r1752	OC−CHO−CH_2OH*+*→OC−CH−CH_2OH*+O*	C-O	1.040	2.451
r1753	OC−CHO−CH_2OH*+*→OC−CHO−CH_2*+OH*	C-OH	0.986	1.547
r1754	OC−CHO−CH_2OH*+*→OC−CHO*+CH_2OH*	C-C	1.162	1.245
r1755	OC−CHO−CH_2OH*+*→OCH−CH_2OH*+CO*	C-C	0.417	1.280
r1756	OC−CHO−CHOH*+*→OC−CHO−CHO*+H*	O-H	0.767	0.852
r1757	OC−CHO−CHOH*+*→OC−CO−CHOH*+H*	C-H	0.242	1.017
r1758	OC−CHO−CHOH*+*→OC−CHO−COH*+H*	C-H	1.039	0.932
r1759	OC−CHO−CHOH*+*→C−CHO−CHOH*+O*	C-O	1.737	2.362
r1760	OC−CHO−CHOH*+*→OC−CH−CHOH*+O*	C-O	1.182	2.566
r1761	OC−CHO−CHOH*+*→OC−CHO−CH*+OH*	C-OH	1.698	1.562
r1762	OC−CHO−CHOH*+*→OC−CHO*+CHOH*	C-C	1.044	1.272
r1763	OC−CHO−CHOH*+*→OCH−CHOH*+CO*	C-C	0.336	1.305
r1764	OC−CHO−CH_2O*+*→OC−CO−CH_2O*+H*	C-H	0.400	1.002
r1765	OC−CHO−CH_2O*+*→OC−CHO−CHO*+H*	C-H	0.878	0.950
r1766	OC−CHO−CH_2O*+*→C−CHO−CH_2O*+O*	C-O	1.401	2.490
r1767	OC−CHO−CH_2O*+*→OC−CH−CH_2O*+O*	C-O	1.183	2.569
r1768	OC−CHO−CH_2O*+*→OC−CHO−CH_2*+O*	C-O	1.487	2.458
r1769	OC−CHO−CH_2O*+*→OC−CHO*+CH_2O*	C-C	1.093	1.270
r1770	OC−CHO−CH_2O*+*→OCH−CH_2O*+CO*	C-C	0.511	1.297
r1771	OC−CHO−CH_2*+*→OC−CO−CH_2*+H*	C-H	0.147	1.039
r1772	OC−CHO−CH_2*+*→OC−CHO−CH*+H*	C-H	1.171	0.929
r1773	OC−CHO−CH_2*+*→C−CHO−CH_2*+O*	C-O	1.846	2.361
r1774	OC−CHO−CH_2*+*→OC−CH−CH_2*+O*	C-O	1.018	2.664
r1775	OC−CHO−CH_2*+*→OC−CHO*+CH_2*	C-C	0.746	1.291
r1776	OC−CHO−CH_2*+*→OCH−CH_2*+CO*	C-C	0.042	1.324
r1777	OC−CHO−COH*+*→OC−CHO−CO*+H*	O-H	0.752	1.221
r1778	OC−CHO−COH*+*→OC−CO−COH*+H*	C-H	0.454	0.983
r1779	OC−CHO−COH*+*→C−CHO−COH*+O*	C-O	1.651	2.355
r1780	OC−CHO−COH*+*→OC−CH−COH*+O*	C-O	0.602	2.739
r1781	OC−CHO−COH*+*→C−CHO−CO*+OH*	C-OH	0.958	1.525
r1782	OC−CHO−COH*+*→OC−CHO*+COH*	C-C	0.149	1.309
r1783	OC−CHO−COH*+*→OCH−COH*+CO*	C-C	0.000	1.447
r1784	OC−CHO−CHO*+*→OC−CO−CHO*+H*	C-H	0.000	1.081
r1785	OC−CHO−CHO*+*→OC−CHO−CO*+H*	C-H	0.700	0.977
r1786	OC−CHO−CHO*+*→C−CHO−CHO*+O*	C-O	1.553	2.461

Supplementary Table 16 – continues from previous page

Tag	Reaction	Type	E_a	E'_a
r1787	OC−CHO−CHO*+*→OC−CH−CHO*+O*	C-O	0.759	2.752
r1788	OC−CHO−CHO*+*→OC−CHO−CH*+O*	C-O	1.737	2.393
r1789	OC−CHO−CHO*+*→OC−CHO*+CHO*	C-C	0.638	1.295
r1790	OC−CHO−CHO*+*→OCH−CHO*+CO*	C-C	0.000	1.523
r1791	OC−CHO−CH*+*→OC−CO−CH*+H*	C-H	0.232	1.004
r1792	OC−CHO−CH*+*→C−CHO−CO*+H*	C-H	0.391	0.987
r1793	OC−CHO−CH*+*→C−CHO−CH*+O*	C-O	1.466	2.412
r1794	OC−CHO−CH*+*→OC−CH−CH*+O*	C-O	0.408	2.799
r1795	OC−CHO−CH*+*→OC−CHO*+CH*	C-C	0.000	1.380
r1796	OC−CHO−CH*+*→CH−CHO*+CO*	C-C	0.000	1.855
r1797	OC−CHO−CO*+*→OC−CO−CO*+H*	C-H	0.312	1.049
r1798	OC−CHO−CO*+*→C−CHO−CO*+O*	C-O	1.579	2.553
r1799	OC−CHO−CO*+*→OC−CH−CO*+O*	C-O	0.649	2.894
r1800	OC−CHO−CO*+*→OC−CHO*+CO*	C-C	0.000	1.557
r1801	OC−CH−CH_2OH*+*→OC−CH−CH_2O*+H*	O-H	0.778	1.221
r1802	OC−CH−CH_2OH*+*→OC−C−CH_2OH*+H*	C-H	0.801	0.966
r1803	OC−CH−CH_2OH*+*→OC−CH−CHOH*+H*	C-H	0.564	0.991
r1804	OC−CH−CH_2OH*+*→C−CH−CH_2OH*+O*	C-O	1.739	2.390
r1805	OC−CH−CH_2OH*+*→OC−CH−CH_2*+OH*	C-OH	0.703	1.499
r1806	OC−CH−CH_2OH*+*→OC−CH*+CH_2OH*	C-C	1.336	1.262
r1807	OC−CH−CH_2OH*+*→CH−CH_2OH*+CO*	C-C	0.816	1.286
r1808	OC−CH−CHOH*+*→OC−CH−CHO*+H*	O-H	0.840	1.534
r1809	OC−CH−CHOH*+*→OC−C−CHOH*+H*	C-H	1.135	0.976
r1810	OC−CH−CHOH*+*→OC−CH−COH*+H*	C-H	0.408	1.054
r1811	OC−CH−CHOH*+*→C−CH−CHOH*+O*	C-O	1.743	2.545
r1812	OC−CH−CHOH*+*→OC−CH−CH*+OH*	C-OH	0.596	1.468
r1813	OC−CH−CHOH*+*→OC−CH*+CHOH*	C-C	1.190	1.289
r1814	OC−CH−CHOH*+*→CH−CHOH*+CO*	C-C	0.638	1.315
r1815	OC−CH−CH_2O*+*→OC−C−CH_2O*+H*	C-H	1.061	0.985
r1816	OC−CH−CH_2O*+*→OC−CH−CHO*+H*	C-H	0.379	1.059
r1817	OC−CH−CH_2O*+*→C−CH−CH_2O*+O*	C-O	1.539	2.625
r1818	OC−CH−CH_2O*+*→OC−CH−CH_2*+O*	C-O	1.433	2.664
r1819	OC−CH−CH_2O*+*→OC−CH*+CH_2O*	C-C	1.242	1.287
r1820	OC−CH−CH_2O*+*→CH−CH_2O*+CO*	C-C	1.020	1.297
r1821	OC−CH−CH_2*+*→OC−C−CH_2*+H*	C-H	1.282	1.000
r1822	OC−CH−CH_2*+*→OC−CH−CH*+H*	C-H	0.573	1.076
r1823	OC−CH−CH_2*+*→C−CH−CH_2*+O*	C-O	1.956	2.602
r1824	OC−CH−CH_2*+*→OC−CH*+CH_2*	C-C	1.155	1.308
r1825	OC−CH−CH_2*+*→CH−CH_2*+CO*	C-C	0.733	1.328
r1826	OC−CH−COH*+*→OC−CH−CO*+H*	O-H	0.932	1.509
r1827	OC−CH−COH*+*→OC−C−COH*+H*	C-H	1.258	1.032
r1828	OC−CH−COH*+*→C−CH−COH*+O*	C-O	1.871	2.735
r1829	OC−CH−COH*+*→C−CH−CO*+OH*	C-OH	1.646	1.491
r1830	OC−CH−COH*+*→OC−CH*+COH*	C-C	1.049	1.326
r1831	OC−CH−COH*+*→CH−COH*+CO*	C-C	0.975	1.329
r1832	OC−CH−CHO*+*→OC−C−CHO*+H*	C-H	1.105	1.054
r1833	OC−CH−CHO*+*→OC−CH−CO*+H*	C-H	0.582	1.110
r1834	OC−CH−CHO*+*→C−CH−CHO*+O*	C-O	1.612	2.848
r1835	OC−CH−CHO*+*→OC−CH−CH*+O*	C-O	1.745	2.799
r1836	OC−CH−CHO*+*→OC−CH*+CHO*	C-C	1.394	1.312
r1837	OC−CH−CHO*+*→CH−CHO*+CO*	C-C	0.821	1.339
r1838	OC−CH−CH*+*→OC−C−CH*+H*	C-H	1.433	1.037
r1839	OC−CH−CH*+*→C−CH−CO*+H*	C-H	1.419	1.039
r1840	OC−CH−CH*+*→C−CH−CH*+O*	C-O	2.272	2.671
r1841	OC−CH−CH*+*→OC−CH*+CH*	C-C	1.092	1.334
r1842	OC−CH−CH*+*→CH−CH*+CO*	C-C	0.635	1.356
r1843	OC−CH−CO*+*→OC−C−CO*+H*	C-H	1.556	1.062
r1844	OC−CH−CO*+*→C−CH−CO*+O*	C-O	2.552	2.698
r1845	OC−CH−CO*+*→OC−CH*+CO*	C-C	0.798	1.365
r1846	OC−CO−CH_2OH*+*→OC−CO−CH_2O*+H*	O-H	0.796	1.209
r1847	OC−CO−CH_2OH*+*→OC−CO−CHOH*+H*	C-H	0.443	1.017
r1848	OC−CO−CH_2OH*+*→C−CO−CH_2OH*+O*	C-O	1.716	2.443
r1849	OC−CO−CH_2OH*+*→OC−C−CH_2OH*+O*	C-O	1.575	2.495

Supplementary Table 16 – continues from previous page

Tag	Reaction	Type	E_a	E'_a
r1850	OC−CO−CH_2OH*+*→OC−CO−CH_2*+OH*	C-OH	0.693	1.491
r1851	OC−CO−CH_2OH*+*→OC−CO*+CH_2OH*	C-C	1.615	1.254
r1852	OC−CO−CH_2OH*+*→OC−CH_2OH*+CO*	C-C	0.466	1.308
r1853	OC−CO−CHOH*+*→OC−CO−CHO*+H*	O-H	0.878	1.268
r1854	OC−CO−CHOH*+*→OC−CO−COH*+H*	C-H	1.337	0.983
r1855	OC−CO−CHOH*+*→C−CO−CHOH*+O*	C-O	2.025	2.541
r1856	OC−CO−CHOH*+*→OC−C−CHOH*+O*	C-O	2.074	2.523
r1857	OC−CO−CHOH*+*→OC−CO−CH*+OH*	C-OH	1.653	1.514
r1858	OC−CO−CHOH*+*→OC−CO*+CHOH*	C-C	1.616	1.281
r1859	OC−CO−CHOH*+*→OC−CHOH*+CO*	C-C	0.648	1.327
r1860	OC−CO−CH_2O*+*→OC−CO−CHO*+H*	C-H	0.504	1.055
r1861	OC−CO−CH_2O*+*→C−CO−CH_2O*+O*	C-O	1.848	2.547
r1862	OC−CO−CH_2O*+*→OC−C−CH_2O*+O*	C-O	1.841	2.549
r1863	OC−CO−CH_2O*+*→OC−CO−CH_2*+O*	C-O	1.436	2.697
r1864	OC−CO−CH_2O*+*→OC−CO*+CH_2O*	C-C	1.493	1.280
r1865	OC−CO−CH_2O*+*→OC−CH_2O*+CO*	C-C	0.596	1.322
r1866	OC−CO−CH_2*+*→OC−CO−CH*+H*	C-H	1.366	1.004
r1867	OC−CO−CH_2*+*→C−CO−CH_2*+O*	C-O	1.899	2.668
r1868	OC−CO−CH_2*+*→OC−C−CH_2*+O*	C-O	2.118	2.588
r1869	OC−CO−CH_2*+*→OC−CO*+CH_2*	C-C	1.436	1.300
r1870	OC−CO−CH_2*+*→OC−CH_2*+CO*	C-C	0.543	1.342
r1871	OC−CO−COH*+*→OC−CO−CO*+H*	O-H	0.828	1.504
r1872	OC−CO−COH*+*→C−CO−COH*+O*	C-O	1.317	2.671
r1873	OC−CO−COH*+*→OC−C−COH*+O*	C-O	1.297	2.679
r1874	OC−CO−COH*+*→C−CO−CO*+OH*	C-OH	1.021	1.497
r1875	OC−CO−COH*+*→OC−CO*+COH*	C-C	0.476	1.319
r1876	OC−CO−COH*+*→OC−COH*+CO*	C-C	0.000	1.428
r1877	OC−CO−CHO*+*→OC−CO−CO*+H*	C-H	1.116	1.049
r1878	OC−CO−CHO*+*→C−CO−CHO*+O*	C-O	2.043	2.678
r1879	OC−CO−CHO*+*→OC−C−CHO*+O*	C-O	1.877	2.739
r1880	OC−CO−CHO*+*→OC−CO−CH*+O*	C-O	2.254	2.601
r1881	OC−CO−CHO*+*→OC−CO*+CHO*	C-C	1.516	1.305
r1882	OC−CO−CHO*+*→OC−CHO*+CO*	C-C	0.594	1.348
r1883	OC−CO−CH*+*→C−CO−CO*+H*	C-H	0.767	1.029
r1884	OC−CO−CH*+*→C−CO−CH*+O*	C-O	1.933	2.524
r1885	OC−CO−CH*+*→OC−C−CH*+O*	C-O	1.470	2.693
r1886	OC−CO−CH*+*→OC−CO*+CH*	C-C	0.507	1.327
r1887	OC−CO−CH*+*→OC−CH*+CO*	C-C	0.000	1.661
r1888	OC−C−CH_2OH*+*→OC−C−CH_2O*+H*	O-H	0.802	1.004
r1889	OC−C−CH_2OH*+*→OC−C−CHOH*+H*	C-H	0.872	0.976
r1890	OC−C−CH_2OH*+*→C−C−CH_2OH*+O*	C-O	1.635	2.488
r1891	OC−C−CH_2OH*+*→OC−C−CH_2*+OH*	C-OH	1.168	1.516
r1892	OC−C−CH_2OH*+*→C−CO*+CH_2OH*	C-C	1.239	1.274
r1893	OC−C−CH_2OH*+*→C−CH_2OH*+CO*	C-C	0.407	1.313
r1894	OC−C−CHOH*+*→OC−C−CHO*+H*	O-H	0.817	1.620
r1895	OC−C−CHOH*+*→OC−C−COH*+H*	C-H	0.453	1.032
r1896	OC−C−CHOH*+*→C−C−CHOH*+O*	C-O	1.542	2.561
r1897	OC−C−CHOH*+*→OC−C−CH*+OH*	C-OH	0.857	1.492
r1898	OC−C−CHOH*+*→C−CO*+CHOH*	C-C	0.769	1.301
r1899	OC−C−CHOH*+*→C−CHOH*+CO*	C-C	0.000	1.394
r1900	OC−C−CH_2O*+*→OC−C−CHO*+H*	C-H	0.349	1.054
r1901	OC−C−CH_2O*+*→C−C−CH_2O*+O*	C-O	1.624	2.567
r1902	OC−C−CH_2O*+*→OC−C−CH_2*+O*	C-O	1.565	2.588
r1903	OC−C−CH_2O*+*→C−CO*+CH_2O*	C-C	0.905	1.299
r1904	OC−C−CH_2O*+*→C−CH_2O*+CO*	C-C	0.417	1.322
r1905	OC−C−CH_2*+*→OC−C−CH*+H*	C-H	0.647	1.037
r1906	OC−C−CH_2*+*→C−C−CH_2*+O*	C-O	1.650	2.611
r1907	OC−C−CH_2*+*→C−CO*+CH_2*	C-C	0.611	1.320
r1908	OC−C−CH_2*+*→C−CH_2*+CO*	C-C	0.000	1.415
r1909	OC−C−COH*+*→OC−C−CO*+H*	O-H	0.900	1.208
r1910	OC−C−COH*+*→C−C−COH*+O*	C-O	1.956	2.622
r1911	OC−C−COH*+*→C−C−CO*+OH*	C-OH	1.313	1.485
r1912	OC−C−COH*+*→C−CO*+COH*	C-C	0.562	1.338

Supplementary Table 16 – continues from previous page

Tag	Reaction	Type	E_a	E'_a
r1913	OC−C−COH*+*→C−COH*+CO*	C-C	0.493	1.341
r1914	OC−C−CHO*+*→OC−C−CO*+H*	C-H	0.977	1.062
r1915	OC−C−CHO*+*→C−C−CHO*+O*	C-O	2.071	2.662
r1916	OC−C−CHO*+*→OC−C−CH*+O*	C-O	1.984	2.693
r1917	OC−C−CHO*+*→C−CO*+CHO*	C-C	1.081	1.324
r1918	OC−C−CHO*+*→C−CHO*+CO*	C-C	0.674	1.343
r1919	OC−C−CH*+*→C−C−CO*+H*	C-H	0.932	1.049
r1920	OC−C−CH*+*→C−C−CH*+O*	C-O	1.851	2.680
r1921	OC−C−CH*+*→C−CO*+CH*	C-C	0.434	1.347
r1922	OC−C−CH*+*→C−CH*+CO*	C-C	0.090	1.363
r1923	OC−C−CO*+*→C−C−CO*+O*	C-O	1.984	2.725
r1924	OC−C−CO*+*→C−CO*+CO*	C-C	0.042	1.377
r1925	C−CHOH−CH_2OH*+*→C−CHO−CH_2OH*+H*	O-H	0.688	0.718
r1926	C−CHOH−CH_2OH*+*→C−CHOH−CH_2O*+H*	O-H	0.688	1.141
r1927	C−CHOH−CH_2OH*+*→C−COH−CH_2OH*+H*	C-H	0.549	0.925
r1928	C−CHOH−CH_2OH*+*→C−CHOH−CHOH*+H*	C-H	0.776	0.901
r1929	C−CHOH−CH_2OH*+*→C−CH−CH_2OH*+OH*	C-OH	1.156	1.562
r1930	C−CHOH−CH_2OH*+*→C−CHOH−CH_2*+OH*	C-OH	1.201	1.565
r1931	C−CHOH−CH_2OH*+*→C−CHOH*+CH_2OH*	C-C	1.235	1.237
r1932	C−CHOH−CH_2OH*+*→HOCH−CH_2OH*+C*	C-C	1.592	1.220
r1933	C−CHOH−CHOH*+*→C−CHO−CHOH*+H*	O-H	0.706	0.882
r1934	C−CHOH−CHOH*+*→C−CHOH−CHO*+H*	O-H	0.706	1.055
r1935	C−CHOH−CHOH*+*→C−COH−CHOH*+H*	C-H	0.262	0.969
r1936	C−CHOH−CHOH*+*→C−CHOH−COH*+H*	C-H	0.748	0.917
r1937	C−CHOH−CHOH*+*→C−CH−CHOH*+OH*	C-OH	0.666	1.526
r1938	C−CHOH−CHOH*+*→C−CHOH−CH*+OH*	C-OH	1.338	1.566
r1939	C−CHOH−CHOH*+*→C−CHOH*+CHOH*	C-C	0.786	1.264
r1940	C−CHOH−CHOH*+*→HOCH−CHOH*+C*	C-C	1.260	1.241
r1941	C−CHOH−CH_2O*+*→C−CHO−CH_2O*+H*	O-H	0.753	1.075
r1942	C−CHOH−CH_2O*+*→C−COH−CH_2O*+H*	C-H	0.429	0.987
r1943	C−CHOH−CH_2O*+*→C−CHOH−CHO*+H*	C-H	0.914	0.934
r1944	C−CHOH−CH_2O*+*→C−CHOH−CH_2*+O*	C-O	1.591	2.379
r1945	C−CHOH−CH_2O*+*→C−CH−CH_2O*+OH*	C-OH	0.678	1.508
r1946	C−CHOH−CH_2O*+*→C−CHOH*+CH_2O*	C-C	1.152	1.262
r1947	C−CHOH−CH_2O*+*→OCH_2−CHOH*+C*	C-C	1.471	1.247
r1948	C−CHOH−CH_2*+*→C−CHO−CH_2*+H*	O-H	0.740	0.672
r1949	C−CHOH−CH_2*+*→C−COH−CH_2*+H*	C-H	0.143	1.008
r1950	C−CHOH−CH_2*+*→C−CHOH−CH*+H*	C-H	0.935	0.923
r1951	C−CHOH−CH_2*+*→C−CH−CH_2*+OH*	C-OH	0.680	1.513
r1952	C−CHOH−CH_2*+*→C−CHOH*+CH_2*	C-C	0.622	1.283
r1953	C−CHOH−CH_2*+*→CH_2−CHOH*+C*	C-C	1.018	1.264
r1954	C−CHOH−COH*+*→C−CHO−COH*+H*	O-H	0.730	0.709
r1955	C−CHOH−COH*+*→C−CHOH−CO*+H*	O-H	0.730	1.376
r1956	C−CHOH−COH*+*→C−COH−COH*+H*	C-H	0.000	1.162
r1957	C−CHOH−COH*+*→C−CH−COH*+OH*	C-OH	0.084	1.482
r1958	C−CHOH−COH*+*→C−CHOH−C*+OH*	C-OH	1.268	1.552
r1959	C−CHOH−COH*+*→C−CHOH*+COH*	C-C	0.167	1.301
r1960	C−CHOH−COH*+*→HOC−CHOH*+C*	C-C	0.907	1.266
r1961	C−CHOH−CHO*+*→C−CHO−CHO*+H*	O-H	0.756	0.951
r1962	C−CHOH−CHO*+*→C−COH−CHO*+H*	C-H	0.000	1.051
r1963	C−CHOH−CHO*+*→C−CHOH−CO*+H*	C-H	0.514	0.980
r1964	C−CHOH−CHO*+*→C−CHOH−CH*+O*	C-O	1.621	2.376
r1965	C−CHOH−CHO*+*→C−CH−CHO*+OH*	C-OH	0.000	1.639
r1966	C−CHOH−CHO*+*→C−CHOH*+CHO*	C-C	0.644	1.287
r1967	C−CHOH−CHO*+*→OCH−CHOH*+C*	C-C	0.849	1.277
r1968	C−CHOH−CH*+*→C−CHO−CH*+H*	O-H	0.738	0.871
r1969	C−CHOH−CH*+*→C−COH−CH*+H*	C-H	0.000	1.246
r1970	C−CHOH−CH*+*→C−CHOH−C*+H*	C-H	0.719	0.945
r1971	C−CHOH−CH*+*→C−CH−CH*+OH*	C-OH	0.396	1.497
r1972	C−CHOH−CH*+*→C−CHOH*+CH*	C-C	0.043	1.309
r1973	C−CHOH−CH*+*→CH−CHOH*+C*	C-C	0.522	1.287
r1974	C−CHOH−CO*+*→C−CHO−CO*+H*	O-H	0.823	0.895
r1975	C−CHOH−CO*+*→C−COH−CO*+H*	C-H	0.075	1.077

Supplementary Table 16 – continues from previous page

Tag	Reaction	Type	E_a	E'_a
r1976	C−CHOH−CO*+*→C−CHOH−C*+O*	C-O	1.922	2.436
r1977	C−CHOH−CO*+*→C−CH−CO*+OH*	C-OH	0.880	1.491
r1978	C−CHOH−CO*+*→C−CHOH*+CO*	C-C	0.000	1.354
r1979	C−CHOH−CO*+*→OC−CHOH*+C*	C-C	0.851	1.299
r1980	C−CHOH−C*+*→C−CHO−C*+H*	O-H	0.771	0.875
r1981	C−CHOH−C*+*→C−COH−C*+H*	C-H	0.044	1.041
r1982	C−CHOH−C*+*→C−CH−C*+OH*	C-OH	0.240	1.475
r1983	C−CHOH−C*+*→C−CHOH*+C*	C-C	0.215	1.312
r1984	C−COH−CH_2OH*+*→C−CO−CH_2OH*+H*	O-H	0.742	0.969
r1985	C−COH−CH_2OH*+*→C−COH−CH_2O*+H*	O-H	0.742	1.377
r1986	C−COH−CH_2OH*+*→C−COH−CHOH*+H*	C-H	0.514	0.969
r1987	C−COH−CH_2OH*+*→C−C−CH_2OH*+OH*	C-OH	1.143	1.540
r1988	C−COH−CH_2OH*+*→C−COH−CH_2*+OH*	C-OH	0.658	1.511
r1989	C−COH−CH_2OH*+*→C−COH*+CH_2OH*	C-C	1.578	1.238
r1990	C−COH−CH_2OH*+*→HOC−CH_2OH*+C*	C-C	1.454	1.244
r1991	C−COH−CHOH*+*→C−CO−CHOH*+H*	O-H	0.807	0.942
r1992	C−COH−CHOH*+*→C−COH−CHO*+H*	O-H	0.807	1.500
r1993	C−COH−CHOH*+*→C−COH−COH*+H*	C-H	0.405	1.030
r1994	C−COH−CHOH*+*→C−C−CHOH*+OH*	C-OH	1.312	1.523
r1995	C−COH−CHOH*+*→C−COH−CH*+OH*	C-OH	0.722	1.488
r1996	C−COH−CHOH*+*→C−COH*+CHOH*	C-C	1.460	1.265
r1997	C−COH−CHOH*+*→HOC−CHOH*+C*	C-C	1.444	1.266
r1998	C−COH−CH_2O*+*→C−CO−CH_2O*+H*	O-H	0.833	0.810
r1999	C−COH−CH_2O*+*→C−COH−CHO*+H*	C-H	0.523	1.036
r2000	C−COH−CH_2O*+*→C−COH−CH_2*+O*	C-O	1.516	2.611
r2001	C−COH−CH_2O*+*→C−C−CH_2O*+OH*	C-OH	1.468	1.521
r2002	C−COH−CH_2O*+*→C−COH*+CH_2O*	C-C	1.678	1.263
r2003	C−COH−CH_2O*+*→HOC−CH_2O*+C*	C-C	1.528	1.271
r2004	C−COH−CH_2*+*→C−CO−CH_2*+H*	O-H	0.864	1.078
r2005	C−COH−CH_2*+*→C−COH−CH*+H*	C-H	0.675	1.044
r2006	C−COH−CH_2*+*→C−C−CH_2*+OH*	C-OH	1.511	1.511
r2007	C−COH−CH_2*+*→C−COH*+CH_2*	C-C	1.454	1.284
r2008	C−COH−CH_2*+*→HOC−CH_2*+C*	C-C	1.441	1.285
r2009	C−COH−COH*+*→C−CO−COH*+H*	O-H	0.897	0.892
r2010	C−COH−COH*+*→C−COH−CO*+H*	O-H	0.897	1.382
r2011	C−COH−COH*+*→C−C−COH*+OH*	C-OH	1.695	1.509
r2012	C−COH−COH*+*→C−COH−C*+OH*	C-OH	1.370	1.490
r2013	C−COH−COH*+*→C−COH*+COH*	C-C	1.297	1.302
r2014	C−COH−COH*+*→HOC−COH*+C*	C-C	1.431	1.296
r2015	C−COH−CHO*+*→C−CO−CHO*+H*	O-H	0.906	0.857
r2016	C−COH−CHO*+*→C−COH−CO*+H*	C-H	0.660	1.077
r2017	C−COH−CHO*+*→C−COH−CH*+O*	C-O	1.760	2.710
r2018	C−COH−CHO*+*→C−C−CHO*+OH*	C-OH	1.607	1.500
r2019	C−COH−CHO*+*→C−COH*+CHO*	C-C	1.662	1.288
r2020	C−COH−CHO*+*→OCH−COH*+C*	C-C	1.546	1.294
r2021	C−COH−CH*+*→C−CO−CH*+H*	O-H	0.917	0.221
r2022	C−COH−CH*+*→C−COH−C*+H*	C-H	1.064	1.041
r2023	C−COH−CH*+*→C−C−CH*+OH*	C-OH	1.605	1.495
r2024	C−COH−CH*+*→C−COH*+CH*	C-C	1.256	1.311
r2025	C−COH−CH*+*→CH−COH*+C*	C-C	1.459	1.301
r2026	C−COH−CO*+*→C−CO−CO*+H*	O-H	0.966	0.475
r2027	C−COH−CO*+*→C−COH−C*+O*	C-O	2.193	2.704
r2028	C−COH−CO*+*→C−C−CO*+OH*	C-OH	1.774	1.485
r2029	C−COH−CO*+*→C−COH*+CO*	C-C	0.955	1.341
r2030	C−COH−CO*+*→OC−COH*+C*	C-C	1.475	1.317
r2031	C−COH−C*+*→C−CO−C*+H*	O-H	0.914	0.842
r2032	C−COH−C*+*→C−C−C*+OH*	C-OH	2.047	1.522
r2033	C−COH−C*+*→C−COH*+C*	C-C	1.180	1.314
r2034	C−CHO−CH_2OH*+*→C−CHO−CH_2O*+H*	O-H	0.692	1.438
r2035	C−CHO−CH_2OH*+*→C−CO−CH_2OH*+H*	C-H	0.373	0.947
r2036	C−CHO−CH_2OH*+*→C−CHO−CHOH*+H*	C-H	0.647	0.918
r2037	C−CHO−CH_2OH*+*→C−CH−CH_2OH*+O*	C-O	1.137	2.390
r2038	C−CHO−CH_2OH*+*→C−CHO−CH_2*+OH*	C-OH	1.302	1.569

Supplementary Table 16 – continues from previous page

Tag	Reaction	Type	E_a	E'_a
r2039	C−CHO−CH_2OH*+*→C−CHO*+CH_2OH*	C-C	1.190	1.240
r2040	C−CHO−CH_2OH*+*→OCH−CH_2OH*+C*	C-C	0.941	1.252
r2041	C−CHO−CHOH*+*→C−CHO−CHO*+H*	O-H	0.731	1.099
r2042	C−CHO−CHOH*+*→C−CO−CHOH*+H*	C-H	0.317	0.982
r2043	C−CHO−CHOH*+*→C−CHO−COH*+H*	C-H	0.944	0.915
r2044	C−CHO−CHOH*+*→C−CH−CHOH*+O*	C-O	0.985	2.545
r2045	C−CHO−CHOH*+*→C−CHO−CH*+OH*	C-OH	1.373	1.557
r2046	C−CHO−CHOH*+*→C−CHO*+CHOH*	C-C	0.887	1.267
r2047	C−CHO−CHOH*+*→OCH−CHOH*+C*	C-C	0.676	1.277
r2048	C−CHO−CH_2O*+*→C−CO−CH_2O*+H*	C-H	0.772	0.984
r2049	C−CHO−CH_2O*+*→C−CHO−CHO*+H*	C-H	1.061	0.953
r2050	C−CHO−CH_2O*+*→C−CH−CH_2O*+O*	C-O	1.241	2.625
r2051	C−CHO−CH_2O*+*→C−CHO−CH_2*+O*	C-O	1.963	2.361
r2052	C−CHO−CH_2O*+*→C−CHO*+CH_2O*	C-C	1.400	1.265
r2053	C−CHO−CH_2O*+*→OCH−CH_2O*+C*	C-C	1.314	1.269
r2054	C−CHO−CH_2*+*→C−CO−CH_2*+H*	C-H	0.000	1.147
r2055	C−CHO−CH_2*+*→C−CHO−CH*+H*	C-H	0.747	0.936
r2056	C−CHO−CH_2*+*→C−CH−CH_2*+O*	C-O	0.825	2.602
r2057	C−CHO−CH_2*+*→C−CHO*+CH_2*	C-C	0.479	1.286
r2058	C−CHO−CH_2*+*→OCH−CH_2*+C*	C-C	0.272	1.296
r2059	C−CHO−COH*+*→C−CHO−CO*+H*	O-H	0.727	1.467
r2060	C−CHO−COH*+*→C−CO−COH*+H*	C-H	0.000	1.179
r2061	C−CHO−COH*+*→C−CH−COH*+O*	C-O	0.438	2.735
r2062	C−CHO−COH*+*→C−CHO−C*+OH*	C-OH	1.135	1.545
r2063	C−CHO−COH*+*→C−CHO*+COH*	C-C	0.071	1.304
r2064	C−CHO−COH*+*→OCH−COH*+C*	C-C	0.294	1.294
r2065	C−CHO−CHO*+*→C−CO−CHO*+H*	C-H	0.225	1.032
r2066	C−CHO−CHO*+*→C−CHO−CO*+H*	C-H	0.644	0.987
r2067	C−CHO−CHO*+*→C−CH−CHO*+O*	C-O	0.527	2.848
r2068	C−CHO−CHO*+*→C−CHO−CH*+O*	C-O	1.718	2.412
r2069	C−CHO−CHO*+*→C−CHO*+CHO*	C-C	0.764	1.290
r2070	C−CHO−CHO*+*→OCH−CHO*+C*	C-C	0.434	1.306
r2071	C−CHO−CH*+*→C−CO−CH*+H*	C-H	0.559	0.976
r2072	C−CHO−CH*+*→C−CHO−C*+H*	C-H	0.758	0.955
r2073	C−CHO−CH*+*→C−CH−CH*+O*	C-O	0.826	2.671
r2074	C−CHO−CH*+*→C−CHO*+CH*	C-C	0.102	1.313
r2075	C−CHO−CH*+*→CH−CHO*+C*	C-C	0.144	1.311
r2076	C−CHO−CO*+*→C−CO−CO*+H*	C-H	0.591	1.029
r2077	C−CHO−CO*+*→C−CH−CO*+O*	C-O	1.282	2.698
r2078	C−CHO−CO*+*→C−CHO−C*+O*	C-O	1.918	2.465
r2079	C−CHO−CO*+*→C−CHO*+CO*	C-C	0.000	1.360
r2080	C−CHO−CO*+*→OC−CHO*+C*	C-C	0.480	1.320
r2081	C−CHO−C*+*→C−CO−C*+H*	C-H	0.213	1.035
r2082	C−CHO−C*+*→C−CH−C*+O*	C-O	0.760	2.768
r2083	C−CHO−C*+*→C−CHO*+C*	C-C	0.245	1.316
r2084	C−CH−CH_2OH*+*→C−CH−CH_2O*+H*	O-H	0.746	1.623
r2085	C−CH−CH_2OH*+*→C−C−CH_2OH*+H*	C-H	0.597	0.963
r2086	C−CH−CH_2OH*+*→C−CH−CHOH*+H*	C-H	0.405	0.984
r2087	C−CH−CH_2OH*+*→C−CH−CH_2*+OH*	C-OH	0.722	1.513
r2088	C−CH−CH_2OH*+*→C−CH*+CH_2OH*	C-C	1.150	1.260
r2089	C−CH−CH_2OH*+*→CH−CH_2OH*+C*	C-C	1.182	1.258
r2090	C−CH−CHOH*+*→C−CH−CHO*+H*	O-H	0.829	1.956
r2091	C−CH−CHOH*+*→C−C−CHOH*+H*	C-H	0.932	0.990
r2092	C−CH−CHOH*+*→C−CH−COH*+H*	C-H	0.345	1.053
r2093	C−CH−CHOH*+*→C−CH−CH*+OH*	C-OH	1.029	1.497
r2094	C−CH−CHOH*+*→C−CH*+CHOH*	C-C	1.155	1.287
r2095	C−CH−CHOH*+*→CH−CHOH*+C*	C-C	1.155	1.287
r2096	C−CH−CH_2O*+*→C−C−CH_2O*+H*	C-H	1.211	0.992
r2097	C−CH−CH_2O*+*→C−CH−CHO*+H*	C-H	0.264	1.093
r2098	C−CH−CH_2O*+*→C−CH−CH_2*+O*	C-O	1.811	2.602
r2099	C−CH−CH_2O*+*→C−CH*+CH_2O*	C-C	1.491	1.285
r2100	C−CH−CH_2O*+*→CH−CH_2O*+C*	C-C	1.821	1.269
r2101	C−CH−CH_2*+*→C−C−CH_2*+H*	C-H	0.976	1.008

Supplementary Table 16 – continues from previous page

Tag	Reaction	Type	E_a	E'_a
r2102	C−CH−CH_2*+*→C−CH−CH*+H*	C-H	0.774	1.029
r2103	C−CH−CH_2*+*→C−CH*+CH_2*	C-C	0.964	1.306
r2104	C−CH−CH_2*+*→CH−CH_2*+C*	C-C	1.094	1.300
r2105	C−CH−COH*+*→C−CH−CO*+H*	O-H	0.930	0.788
r2106	C−CH−COH*+*→C−C−COH*+H*	C-H	1.435	1.012
r2107	C−CH−COH*+*→C−CH−C*+OH*	C-OH	1.355	1.475
r2108	C−CH−COH*+*→C−CH*+COH*	C-C	1.075	1.324
r2109	C−CH−COH*+*→CH−COH*+C*	C-C	1.554	1.301
r2110	C−CH−CHO*+*→C−C−CHO*+H*	C-H	1.721	1.026
r2111	C−CH−CHO*+*→C−CH−CO*+H*	C-H	1.601	1.039
r2112	C−CH−CHO*+*→C−CH−CH*+O*	C-O	2.453	2.671
r2113	C−CH−CHO*+*→C−CH*+CHO*	C-C	1.792	1.310
r2114	C−CH−CHO*+*→CH−CHO*+C*	C-C	1.771	1.311
r2115	C−CH−CH*+*→C−C−CH*+H*	C-H	0.998	1.033
r2116	C−CH−CH*+*→C−CH−C*+H*	C-H	0.705	1.064
r2117	C−CH−CH*+*→C−CH*+CH*	C-C	0.653	1.333
r2118	C−CH−CH*+*→CH−CH*+C*	C-C	0.749	1.328
r2119	C−CH−CO*+*→C−C−CO*+H*	C-H	0.948	1.049
r2120	C−CH−CO*+*→C−CH−C*+O*	C-O	1.629	2.768
r2121	C−CH−CO*+*→C−CH*+CO*	C-C	0.106	1.363
r2122	C−CH−CO*+*→OC−CH*+C*	C-C	0.658	1.337
r2123	C−CH−C*+*→C−C−C*+H*	C-H	1.753	0.991
r2124	C−CH−C*+*→C−CH*+C*	C-C	0.960	1.335
r2125	C−CO−CH_2OH*+*→C−CO−CH_2O*+H*	O-H	0.775	1.159
r2126	C−CO−CH_2OH*+*→C−CO−CHOH*+H*	C-H	0.620	0.982
r2127	C−CO−CH_2OH*+*→C−C−CH_2OH*+O*	C-O	1.443	2.488
r2128	C−CO−CH_2OH*+*→C−CO−CH_2*+OH*	C-OH	0.658	1.498
r2129	C−CO−CH_2OH*+*→C−CO*+CH_2OH*	C-C	1.047	1.274
r2130	C−CO−CH_2OH*+*→OC−CH_2OH*+C*	C-C	0.909	1.280
r2131	C−CO−CHOH*+*→C−CO−CHO*+H*	O-H	0.827	1.336
r2132	C−CO−CHOH*+*→C−CO−COH*+H*	C-H	0.544	1.029
r2133	C−CO−CHOH*+*→C−C−CHOH*+O*	C-O	1.608	2.561
r2134	C−CO−CHOH*+*→C−CO−CH*+OH*	C-OH	1.595	1.531
r2135	C−CO−CHOH*+*→C−CO*+CHOH*	C-C	0.835	1.301
r2136	C−CO−CHOH*+*→OC−CHOH*+C*	C-C	0.878	1.299
r2137	C−CO−CH_2O*+*→C−CO−CHO*+H*	C-H	0.545	1.032
r2138	C−CO−CH_2O*+*→C−C−CH_2O*+O*	C-O	1.615	2.567
r2139	C−CO−CH_2O*+*→C−CO−CH_2*+O*	C-O	1.337	2.668
r2140	C−CO−CH_2O*+*→C−CO*+CH_2O*	C-C	0.896	1.299
r2141	C−CO−CH_2O*+*→OC−CH_2O*+C*	C-C	1.010	1.294
r2142	C−CO−CH_2*+*→C−CO−CH*+H*	C-H	1.517	0.976
r2143	C−CO−CH_2*+*→C−C−CH_2*+O*	C-O	1.948	2.611
r2144	C−CO−CH_2*+*→C−CO*+CH_2*	C-C	0.909	1.320
r2145	C−CO−CH_2*+*→OC−CH_2*+C*	C-C	1.027	1.314
r2146	C−CO−COH*+*→C−CO−CO*+H*	O-H	0.896	0.895
r2147	C−CO−COH*+*→C−C−COH*+O*	C-O	1.927	2.622
r2148	C−CO−COH*+*→C−CO−C*+OH*	C-OH	1.442	1.494
r2149	C−CO−COH*+*→C−CO*+COH*	C-C	0.533	1.338
r2150	C−CO−COH*+*→OC−COH*+C*	C-C	0.985	1.317
r2151	C−CO−CHO*+*→C−CO−CO*+H*	C-H	1.054	1.029
r2152	C−CO−CHO*+*→C−C−CHO*+O*	C-O	1.843	2.662
r2153	C−CO−CHO*+*→C−CO−CH*+O*	C-O	2.220	2.524
r2154	C−CO−CHO*+*→C−CO*+CHO*	C-C	0.854	1.324
r2155	C−CO−CHO*+*→OC−CHO*+C*	C-C	0.943	1.320
r2156	C−CO−CH*+*→C−CO−C*+H*	C-H	0.434	1.035
r2157	C−CO−CH*+*→C−C−CH*+O*	C-O	1.219	2.680
r2158	C−CO−CH*+*→C−CO*+CH*	C-C	0.000	1.545
r2159	C−CO−CH*+*→OC−CH*+C*	C-C	0.010	1.337
r2160	C−CO−CO*+*→C−C−CO*+O*	C-O	1.647	2.725
r2161	C−CO−CO*+*→C−CO−C*+O*	C-O	1.756	2.685
r2162	C−CO−CO*+*→C−CO*+CO*	C-C	0.000	1.672
r2163	C−CO−CO*+*→OC−CO*+C*	C-C	0.716	1.330
r2164	C−CO−C*+*→C−C−C*+O*	C-O	2.139	2.564

Supplementary Table 16 – continues from previous page

Tag	Reaction	Type	E_a	E'_a
r2165	C−CO−C*+*→C−CO*+C*	C-C	0.349	1.349
r2166	C−C−CH_2OH*+*→C−C−CH_2O*+H*	O-H	0.799	1.090
r2167	C−C−CH_2OH*+*→C−C−CHOH*+H*	C-H	0.719	0.990
r2168	C−C−CH_2OH*+*→C−C−CH_2*+OH*	C-OH	1.055	1.511
r2169	C−C−CH_2OH*+*→C−C*+CH_2OH*	C-C	1.859	1.244
r2170	C−C−CH_2OH*+*→C−CH_2OH*+C*	C-C	0.974	1.285
r2171	C−C−CHOH*+*→C−C−CHO*+H*	O-H	0.837	1.212
r2172	C−C−CHOH*+*→C−C−COH*+H*	C-H	0.785	1.012
r2173	C−C−CHOH*+*→C−C−CH*+OH*	C-OH	1.050	1.495
r2174	C−C−CHOH*+*→C−C*+CHOH*	C-C	1.556	1.271
r2175	C−C−CHOH*+*→C−CHOH*+C*	C-C	0.680	1.312
r2176	C−C−CH_2O*+*→C−C−CHO*+H*	C-H	0.673	1.026
r2177	C−C−CH_2O*+*→C−C−CH_2*+O*	C-O	1.569	2.611
r2178	C−C−CH_2O*+*→C−C*+CH_2O*	C-C	1.615	1.269
r2179	C−C−CH_2O*+*→C−CH_2O*+C*	C-C	1.075	1.294
r2180	C−C−CH_2*+*→C−C−CH*+H*	C-H	0.774	1.033
r2181	C−C−CH_2*+*→C−C*+CH_2*	C-C	1.339	1.290
r2182	C−C−CH_2*+*→C−CH_2*+C*	C-C	0.613	1.324
r2183	C−C−COH*+*→C−C−CO*+H*	O-H	0.870	1.252
r2184	C−C−COH*+*→C−C−C*+OH*	C-OH	1.741	1.522
r2185	C−C−COH*+*→C−C*+COH*	C-C	0.995	1.308
r2186	C−C−COH*+*→C−COH*+C*	C-C	0.875	1.314
r2187	C−C−CHO*+*→C−C−CO*+H*	C-H	0.814	1.049
r2188	C−C−CHO*+*→C−C−CH*+O*	C-O	1.733	2.680
r2189	C−C−CHO*+*→C−C*+CHO*	C-C	1.439	1.294
r2190	C−C−CHO*+*→C−CHO*+C*	C-C	0.980	1.316
r2191	C−C−CH*+*→C−C−C*+H*	C-H	1.427	0.991
r2192	C−C−CH*+*→C−C*+CH*	C-C	1.031	1.316
r2193	C−C−CH*+*→C−CH*+C*	C-C	0.634	1.335
r2194	C−C−CO*+*→C−C−C*+O*	C-O	2.288	2.564
r2195	C−C−CO*+*→C−C*+CO*	C-C	0.550	1.347
r2196	C−C−CO*+*→C−CO*+C*	C-C	0.498	1.349
r2197	C−C−C*+*→C−C*+C*	C-C	0.541	1.319

Supplementary Table 17: **Glycerol tags**. Tags used for the reaction network of glycerol and other C_3 derivatives in the ioChem-BD database.

Tag	Formula
38301	$HOCH_2-CHOH-CH_2OH$
37301	$HOCH_2-COH-CH_2OH$
37302	$HOCH_2-CHO-CH_2OH$
37303	$HOCH-CHOH-CH_2OH$
37304	$OCH_2-CHOH-CH_2OH$
37201	$HOCH_2-CH-CH_2OH$
37202	$CH_2-CHOH-CH_2OH$
36301	$HOCH_2-CO-CH_2OH$
36302	$HOCH-CHOH-CHOH$
36303	$HOCH-COH-CH_2OH$
36304	$HOCH-CHO-CH_2OH$
36305	$OCH_2-CHOH-CHOH$
36306	$OCH_2-CHOH-CH_2O$
36307	$OCH_2-COH-CH_2OH$
36308	$OCH_2-CHO-CH_2OH$
36309	$HOC-CHOH-CH_2OH$
36310	$OCH-CHOH-CH_2OH$
36201	$HOCH_2-C-CH_2OH$
36202	$HOCH-CH-CH_2OH$
36203	$OCH_2-CH-CH_2OH$
36204	$CH_2-CHOH-CHOH$
36205	$CH_2-CHOH-CH_2O$
36206	$CH_2-COH-CH_2OH$
36207	$CH_2-CHO-CH_2OH$
36208	$CH-CHOH-CH_2OH$
36101	$CH_2-CHOH-CH_2$
36102	$CH_2-CH-CH_2OH$
35301	$HOCH-COH-CHOH$
35302	$HOCH-CHO-CHOH$
35303	$HOCH-CO-CH_2OH$
35304	$OCH_2-COH-CHOH$
35305	$OCH_2-COH-CH_2O$
35306	$OCH_2-CHO-CHOH$
35307	$OCH_2-CHO-CH_2O$
35308	$OCH_2-CO-CH_2OH$
35309	$HOC-CHOH-CHOH$
35310	$HOC-CHOH-CH_2O$
35311	$HOC-COH-CH_2OH$
35312	$HOC-CHO-CH_2OH$
35313	$OCH-CHOH-CHOH$
35314	$OCH-CHOH-CH_2O$
35315	$OCH-COH-CH_2OH$
35316	$OCH-CHO-CH_2OH$
35317	$OC-CHOH-CH_2OH$
35201	$HOCH-CH-CHOH$
35202	$HOCH-C-CH_2OH$
35203	$OCH_2-CH-CHOH$
35204	$OCH_2-CH-CH_2O$
35205	OCH_2-C-CH_2OH
35206	$CH_2-COH-CHOH$
35207	$CH_2-COH-CH_2O$
35208	$CH_2-CHO-CHOH$
35209	$CH_2-CHO-CH_2O$
35210	$CH_2-CO-CH_2OH$

Supplementary Table 17 – continued

Tag	Formula
35211	$HOC{-}CHOH{-}CH_2$
35212	$HOC{-}CH{-}CH_2OH$
35213	$OCH{-}CHOH{-}CH_2$
35214	$OCH{-}CH{-}CH_2OH$
35215	$CH{-}CHOH{-}CHOH$
35216	$CH{-}CHOH{-}CH_2O$
35217	$CH{-}COH{-}CH_2OH$
35218	$CH{-}CHO{-}CH_2OH$
35219	$C{-}CHOH{-}CH_2OH$
35101	$CH_2{-}COH{-}CH_2$
35102	$CH_2{-}CHO{-}CH_2$
35103	$CH_2{-}CH{-}CHOH$
35104	$CH_2{-}CH{-}CH_2O$
35105	$CH_2{-}C{-}CH_2OH$
35106	$CH{-}CHOH{-}CH_2$
35107	$CH{-}CH{-}CH_2OH$
35001	$CH_2{-}CH{-}CH_2$
34301	$HOCH{-}CO{-}CHOH$
34302	$OCH_2{-}CO{-}CHOH$
34303	$OCH_2{-}CO{-}CH_2O$
34304	$HOC{-}CHOH{-}COH$
34305	$HOC{-}COH{-}CHOH$
34306	$HOC{-}COH{-}CH_2O$
34307	$HOC{-}CHO{-}CHOH$
34308	$HOC{-}CHO{-}CH_2O$
34309	$HOC{-}CO{-}CH_2OH$
34310	$OCH{-}CHOH{-}COH$
34311	$OCH{-}CHOH{-}CHO$
34312	$OCH{-}COH{-}CHOH$
34313	$OCH{-}COH{-}CH_2O$
34314	$OCH{-}CHO{-}CHOH$
34315	$OCH{-}CHO{-}CH_2O$
34316	$OCH{-}CO{-}CH_2OH$
34317	$OC{-}CHOH{-}CHOH$
34318	$OC{-}CHOH{-}CH_2O$
34319	$OC{-}COH{-}CH_2OH$
34320	$OC{-}CHO{-}CH_2OH$
34201	$HOCH{-}C{-}CHOH$
34202	$OCH_2{-}C{-}CHOH$
34203	$OCH_2{-}C{-}CH_2O$
34204	$CH_2{-}CO{-}CHOH$
34205	$CH_2{-}CO{-}CH_2O$
34206	$HOC{-}COH{-}CH_2$
34207	$HOC{-}CHO{-}CH_2$
34208	$HOC{-}CH{-}CHOH$
34209	$HOC{-}CH{-}CH_2O$
34210	$HOC{-}C{-}CH_2OH$
34211	$OCH{-}COH{-}CH_2$
34212	$OCH{-}CHO{-}CH_2$
34213	$OCH{-}CH{-}CHOH$
34214	$OCH{-}CH{-}CH_2O$
34215	$OCH{-}C{-}CH_2OH$
34216	$CH{-}CHOH{-}COH$
34217	$CH{-}CHOH{-}CHO$
34218	$CH{-}COH{-}CHOH$
34219	$CH{-}COH{-}CH_2O$
34220	$CH{-}CHO{-}CHOH$
34221	$CH{-}CHO{-}CH_2O$
34222	$CH{-}CO{-}CH_2OH$

Supplementary Table 17 – continued

Tag	Formula
34223	$OC-CHOH-CH_2$
34224	$OC-CH-CH_2OH$
34225	$C-CHOH-CHOH$
34226	$C-CHOH-CH_2O$
34227	$C-COH-CH_2OH$
34228	$C-CHO-CH_2OH$
34101	$CH_2-CO-CH_2$
34102	$CH_2-C-CHOH$
34103	CH_2-C-CH_2O
34104	$HOC-CH-CH_2$
34105	$OCH-CH-CH_2$
34106	$CH-CHOH-CH$
34107	$CH-COH-CH_2$
34108	$CH-CHO-CH_2$
34109	$CH-CH-CHOH$
34110	$CH-CH-CH_2O$
34111	$CH-C-CH_2OH$
34112	$C-CHOH-CH_2$
34113	$C-CH-CH_2OH$
34001	CH_2-C-CH_2
34002	$CH-CH-CH_2$
33301	$HOC-COH-COH$
33302	$HOC-CHO-COH$
33303	$HOC-CO-CHOH$
33304	$HOC-CO-CH_2O$
33305	$OCH-COH-COH$
33306	$OCH-COH-CHO$
33307	$OCH-CHO-COH$
33308	$OCH-CHO-CHO$
33309	$OCH-CO-CHOH$
33310	$OCH-CO-CH_2O$
33311	$OC-CHOH-COH$
33312	$OC-CHOH-CHO$
33313	$OC-COH-CHOH$
33314	$OC-COH-CH_2O$
33315	$OC-CHO-CHOH$
33316	$OC-CHO-CH_2O$
33317	$OC-CO-CH_2OH$
33201	$HOC-CH-COH$
33202	$HOC-CO-CH_2$
33203	$HOC-C-CHOH$
33204	$HOC-C-CH_2O$
33205	$OCH-CH-COH$
33206	$OCH-CH-CHO$
33207	$OCH-CO-CH_2$
33208	$OCH-C-CHOH$
33209	$OCH-C-CH_2O$
33210	$CH-COH-COH$
33211	$CH-COH-CHO$
33212	$CH-CHO-COH$
33213	$CH-CHO-CHO$
33214	$CH-CO-CHOH$
33215	$CH-CO-CH_2O$
33216	$OC-CHOH-CH$
33217	$OC-COH-CH_2$
33218	$OC-CHO-CH_2$
33219	$OC-CH-CHOH$
33220	$OC-CH-CH_2O$
33221	$OC-C-CH_2OH$

Supplementary Table 17 – continued

Tag	Formula
33222	C−CHOH−COH
33223	C−CHOH−CHO
33224	C−COH−CHOH
33225	C−COH−CH_2O
33226	C−CHO−CHOH
33227	C−CHO−CH_2O
33228	C−CO−CH_2OH
33101	HOC−C−CH_2
33102	OCH−C−CH_2
33103	CH−COH−CH
33104	CH−CHO−CH
33105	CH−CH−COH
33106	CH−CH−CHO
33107	CH−CO−CH_2
33108	CH−C−CHOH
33109	CH−C−CH_2O
33110	OC−CH−CH_2
33111	C−CHOH−CH
33112	C−COH−CH_2
33113	C−CHO−CH_2
33114	C−CH−CHOH
33115	C−CH−CH_2O
33116	C−C−CH_2OH
33001	CH−CH−CH
33002	CH−C−CH_2
33003	C−CH−CH_2
32301	HOC−CO−COH
32302	OCH−CO−COH
32303	OCH−CO−CHO
32304	OC−CHOH−CO
32305	OC−COH−COH
32306	OC−COH−CHO
32307	OC−CHO−COH
32308	OC−CHO−CHO
32309	OC−CO−CHOH
32310	OC−CO−CH_2O
32201	HOC−C−COH
32202	OCH−C−COH
32203	OCH−C−CHO
32204	CH−CO−COH
32205	CH−CO−CHO
32206	OC−COH−CH
32207	OC−CHO−CH
32208	OC−CH−COH
32209	OC−CH−CHO
32210	OC−CO−CH_2
32211	OC−C−CHOH
32212	OC−C−CH_2O
32213	C−CHOH−CO
32214	C−COH−COH
32215	C−COH−CHO
32216	C−CHO−COH
32217	C−CHO−CHO
32218	C−CO−CHOH
32219	C−CO−CH_2O
32101	CH−CO−CH
32102	CH−C−COH
32103	CH−C−CHO
32104	OC−CH−CH

Supplementary Table 17 – continued

Tag	Formula
32105	OC−C−CH_2
32106	C−CHOH−C
32107	C−COH−CH
32108	C−CHO−CH
32109	C−CH−COH
32110	C−CH−CHO
32111	C−CO−CH_2
32112	C−C−CHOH
32113	C−C−CH_2O
32001	CH−C−CH
32002	C−CH−CH
32003	C−C−CH_2
31301	OC−COH−CO
31302	OC−CHO−CO
31303	OC−CO−COH
31304	OC−CO−CHO
31201	OC−CH−CO
31202	OC−CO−CH
31203	OC−C−COH
31204	OC−C−CHO
31205	C−COH−CO
31206	C−CHO−CO
31207	C−CO−COH
31208	C−CO−CHO
31101	OC−C−CH
31102	C−COH−C
31103	C−CHO−C
31104	C−CH−CO
31105	C−CO−CH
31106	C−C−COH
31107	C−C−CHO
31001	C−CH−C
31002	C−C−CH
30301	OC−CO−CO
30201	OC−C−CO
30202	C−CO−CO
30101	C−CO−C
30102	C−C−CO
30001	C−C−C

Supplementary References

[1] Thorne, L. R. An innovative approach to balancing chemical-reaction equations: A simplified matrix-inversion technique for determining the matrix null space. *Chem. Educator* **15**, 304–308 (2010).

[2] Zaffran, J., Michel, C., Auneau, F., Delbecq, F. & Sautet, P. Linear energy relations as predictive tools for polyalcohol catalytic reactivity. *ACS Catal.* **4**, 464–468 (2014).

[3] Garcia-Ratés, M., García-Muelas, R. & López, N. Solvation effects on methanol decomposition on Pd(111), Pt(111) and Ru(0001). *J. Phys. Chem. C* **121**, 13803–13809 (2017).

[4] Davda, R. R., Shabaker, J. W., Huber, G. W., Cortright, R. D. & Dumesic, J. A. Aqueous-phase reforming of ethylene glycol on silica-supported metal catalysts. *Appl. Catal. B* **43**, 13–26 (2003).

[5] Salciccioli, M., Chen, Y. & Vlachos, D. G. Microkinetic modeling and reduced rate expressions of ethylene hydrogenation and ethane hydrogenolysis on platinum. *Ind. Eng. Chem. Res.* **50**, 28–40 (2011).

[6] Lausche, A. C. *et al.* On the effect of coverage-dependent adsorbate–adsorbate interactions for CO methanation on transition metal surfaces. *J. Catal.* **307**, 275–282 (2013).

[7] García-Muelas, R., Li, Q. & López, N. Density functional theory comparison of methanol decomposition and reverse reactions on metal surfaces. *ACS Catal.* **5**, 1027–1036 (2015).

[8] Álvarez-Moreno, M. *et al.* Managing the computational chemistry big data problem: The ioChem-BD platform. *J. Chem. Inf. Model.* **55**, 95–103 (2014).

[9] Wang, S. *et al.* Universal transition state scaling relations for (de) hydrogenation over transition metals. *Phys. Chem. Chem. Phys.* **13**, 20760–20765 (2011).

[10] Liu, B. & Greeley, J. A density functional theory analysis of trends in glycerol decomposition on close-packed transition metal surfaces. *Phys. Chem. Chem. Phys.* **15**, 6475–6485 (2013).

[11] Wang, S. *et al.* Universal Brønsted-Evans-Polanyi relations for C–C, C–O, C–N, N–O, N–N, and O–O dissociation reactions. *Catal. Lett.* **141**, 370–373 (2011).

[12] Ferrin, P. *et al.* Modeling ethanol decomposition on transition metals: A combined application of scaling and Brønsted-Evans-Polanyi relations. *J. Am. Chem. Soc.* **131**, 5809–5815 (2009).

[13] Li, Q., García-Muelas, R. & López, N. Microkinetics of alcohol reforming for H_2 production from a FAIR density functional theory database. *https://doi.org/10.19061/iochem-bd-1-37*.

[14] Li, R., Zhang, M. & Yu, Y. A DFT study on the Cu(111) surface for ethyl acetate synthesis from ethanol dehydrogenation. *Appl. Surf. Sci.* **258**, 6777–6784 (2012).

[15] Michel, C., Auneau, F., Delbecq, F. & Sautet, P. C–H versus O–H Bond dissociation for alcohols on a Rh(111) surface: A strong assistance from hydrogen bonded neighbors. *ACS Catal.* **1**, 1430–1440 (2011).

[16] Chiu, C.-C., Genest, A. & Rösch, N. Decomposition of ethanol over Ru(0001): A DFT study. *Top. Catal.* **56**, 874–884 (2013).

[17] Engelbach, H. & Sprague, M. J. Preparation of glyoxal from ethylene glycol (1981). US Patent 4282374.

[18] Salciccioli, M., Yu, W., Barteau, M. A., Chen, J. G. & Vlachos, D. G. Differentiation of O–H and C–H bond scission mechanisms of ethylene glycol on Pt and Ni/Pt using theory and isotopic labeling experiments. *J. Am. Chem. Soc.* **133**, 7996–8004 (2011).

[19] Yu, W. *et al.* Theoretical and experimental studies of C–C versus C–O bond scission of ethylene glycol reaction pathways via metal-modified molybdenum carbides. *ACS Catal.* **4**, 1409–1418 (2014).

[20] Gokhale, A. A., Dumesic, J. A. & Mavrikakis, M. On the mechanism of low-temperature water gas shift reaction on copper. *J. Am. Chem. Soc.* **130**, 1402–1414 (2008).

[21] Grabow, L. C., Gokhale, A. A., Evans, S. T., Dumesic, J. A. & Mavrikakis, M. Mechanism of the water gas shift reaction on Pt: First principles, experiments, and microkinetic modeling. *J. Phys. Chem. C* **112**, 4608–4617 (2008).

[22] Hrbek, J., DePaola, R. A. & Hoffmann, F. M. The interaction of methanol with Ru(001). *J. Chem. Phys.* **81**, 2818–2827 (1984).

[23] Gates, J. A. & Kesmodel, L. L. Methanol adsorption and decomposition on clean and oxygen precovered palladium(111). *J. Catal.* **83**, 437–445 (1983).

[24] Kok, G. A., Noordermeer, A. & Nieuwenhuys, B. E. Decomposition of methanol and the interaction of coadsorbed hydrogen and carbon monoxide on a Pd(111) surface. *Surf. Sci.* **135**, 65–80 (1983).

[25] Gong, J., Flaherty, D. W., Ojifinni, R. A., White, J. M. & Mullins, C. B. Surface chemistry of methanol on clean and atomic oxygen pre-covered Au(111). *J. Phys. Chem. C* **112**, 5501–5509 (2008).

[26] Ehlers, D. H., Spitzer, A. & Lüth, H. The adsorption of methanol on Pt(111), an IR reflection and UV photoemission study. *Surf. Sci.* **160**, 57–69 (1985).

[27] Solymosi, F., Berkó, A. & Tarnóczi, T. I. Adsorption and decomposition of methanol on Rh(111) studied by electron energy loss and thermal desorption spectroscopy. *Surf. Sci.* **141**, 533–548 (1984).

[28] Chen, J.-J., Jiang, Z.-C., Zhou, Y., Chakraborty, B. R. & Winograd, N. Spectroscopic studies of methanol decomposition on Pd(111). *Surf. Sci.* **328**, 248–262 (1995).

[29] Rebholz, M. & Kruse, N. Mechanisms of methanol decomposition on Pd(111). *J. Chem. Phys.* **95**, 7745–7759 (1991).

[30] Gazdzicki, P. & Jakob, P. Reactions of methanol on Ru(0001). *J. Phys. Chem. C* **114**, 2655–2663 (2010).

[31] Sexton, B. A. Methanol decomposition on platinum(111). *Surf. Sci.* **102**, 271–281 (1981).

[32] Sexton, B. A., Rendulic, K. D. & Huges, A. E. Decomposition pathways of C_1-C_4 alcohols adsorbed on platinum(111). *Surf. Sci.* **121**, 181–198 (1982).

[33] Williams, R. M., Pang, S. H. & Medlin, J. W. O–H versus C–H bond scission sequence in ethanol decomposition on Pd(111). *Surf. Sci.* **619**, 114–118 (2014).

[34] Lee, A. F., Gawthrope, D. E., Hart, N. J. & Wilson, K. A Fast XPS study of the surface chemistry of ethanol over Pt{111}. *Surf. Sci.* **548**, 200–208 (2004).

[35] Griffin, M. B., Jorgensen, E. L. & Medlin, J. W. The adsorption and reaction of ethylene glycol and 1,2-propanediol on Pd(111): A TPD and HREELS study. *Surf. Sci.* **604**, 1558–1564 (2010).

[36] Skoplyak, O., Barteau, M. A. & Chen, J. G. Reforming of oxygenates for H_2 production: Correlating reactivity of ethylene glycol and ethanol on Pt(111) and Ni/Pt(111) with surface *d*-band center. *J. Phys. Chem. B* **110**, 1686–1694 (2006).

[37] Davis, J. L. & Barteau, M. A. Decarbonylation and decomposition pathways of alcohols on Pd(111). *Surf. Sci.* **187**, 387–406 (1987).

[38] Jenkins, J. W. & Shutt, E. The HotSpot^{TM} Reactor. *Platin. Met. Rev* **33**, 118–127 (1989).

[39] Edwards, N. *et al.* On-board hydrogen generation for transport applications: The HotSpot^{TM} methanol processor. *J. Power Sources* **71**, 123–128 (1998).

[40] Geissler, K. *et al.* Autothermal methanol reforming for hydrogen production in fuel cell applications. *Phys. Chem. Chem. Phys.* **3**, 289–293 (2001).

[41] Raimondi, F., Geissler, K., Wambach, J. & Wokaun, A. Hydrogen production by methanol reforming: Post-reaction characterisation of a Cu/ZnO/Al_2O_3 catalyst by XPS and TPD. *Appl. Surf. Sci.* **189**, 59–71 (2002).

[42] Dauenhauer, P. J., Salge, J. R. & Schmidt, L. D. Renewable hydrogen by autothermal steam reforming of volatile carbohydrates. *J. Catal.* **244**, 238–247 (2006).

[43] Deluga, G. A., Salge, J. R., Schmidt, L. D. & Verykios, X. E. Renewable hydrogen from ethanol by autothermal reforming. *Science* **303**, 993–997 (2004).

[44] Gutierrez, A., Karinen, R., Airaksinen, S., Kaila, R. & Krause, A. Autothermal reforming of ethanol on noble metal catalysts. *Int. J. Hydrog. Energy* **36**, 8967–8977 (2011).

[45] Hung, C.-C., Chen, S.-L., Liao, Y.-K., Chen, C.-H. & Wang, J.-H. Oxidative steam reforming of ethanol for hydrogen production on M/Al_2O_3. *Int. J. Hydrog. Energy* **37**, 4955–4966 (2012).

[46] Cai, W., Ramírez de la Piscina, P. & Homs, N. Oxidative steam reforming of bio-butanol for hydrogen production: Effects of noble metals on bimetallic CoM/ZnO catalysts (M=Ru, Rh, Ir, Pd). *Appl. Catal., B* **145**, 56–62 (2014).

[47] Harju, H., Lehtonen, J. & Lefferts, L. Steam and autothermal reforming of *n*-butanol over Rh/ZrO_2 catalyst. *Catal. Today* **244**, 47–57 (2015).

[48] Iwasa, N., Masuda, S., Ogawa, N. & Takezawa, N. Steam reforming of methanol over Pd/ZnO: Effect of the formation of PdZn alloys upon the reaction. *Appl. Catal., A.* **125**, 145–157 (1995).

[49] Shishido, T., Yamamoto, Y., Morioka, H., Takaki, K. & Takehira, K. Active Cu/ZnO and Cu/ZnO/Al_2O_3 catalysts prepared by homogeneous precipitation method in steam reforming of methanol. *Appl. Catal., A.* **263**, 249–253 (2004).

[50] Yao, C.-Z. *et al.* Effect of preparation method on the hydrogen production from methanol steam reforming over binary Cu/ZrO_2 catalysts. *Appl. Catal., A.* **297**, 151–158 (2006).

[51] Cavallaro, S. Ethanol steam reforming on Rh/Al_2O_3 catalysts. *Energ. Fuel.* **14**, 1195–1199 (2000).

[52] Llorca, J., Homs, N., Sales, J. & Ramírez de la Piscina, P. Efficient production of hydrogen over supported cobalt catalysts from ethanol steam reforming. *J. Catal.* **209**, 306–317 (2002).

[53] Vaidya, P. D. & Rodrigues, A. E. Kinetics of steam reforming of ethanol over a Ru/Al_2O_3 catalyst. *Ind. Eng. Chem. Res.* **45**, 6614–6618 (2006).

[54] Vizcaíno, A., Carrero, A. & Calles, J. Hydrogen production by ethanol steam reforming over Cu–Ni supported catalysts. *Int. J. Hydrog. Energy* **32**, 1450–1461 (2007).

[55] Basagiannis, A. C., Panagiotopoulou, P. & Verykios, X. E. Low temperature steam reforming of ethanol over supported noble metal catalysts. *Top. Catal.* **51**, 2–12 (2008).

[56] Ciambelli, P., Palma, V. & Ruggiero, A. Low temperature catalytic steam reforming of ethanol. 2. Preliminary kinetic investigation of Pt/CeO_2 catalysts. *Appl. Catal., B* **96**, 190–197 (2010).

[57] Bayram, B., Soykal, I. I., von Deak, D., Miller, J. T. & Ozkan, U. S. Ethanol steam reforming over Co-based catalysts: Investigation of cobalt coordination environment under reaction conditions. *J. Catal.* **284**, 77–89 (2011).

[58] Da Silva, A. M. *et al.* Steam and CO_2 reforming of ethanol over Rh/CeO_2 catalyst. *Appl. Catal., B* **102**, 94–109 (2011).

[59] Ramos, I. A. C. *et al.* Hydrogen production from ethanol steam reforming on M/CeO_2/YSZ (M=Ru, Pd, Ag) nanocomposites. *Catal. Today* **180**, 96–104 (2012).

[60] Rossetti, I. *et al.* Ni/SiO_2 and Ni/ZrO_2 catalysts for the steam reforming of ethanol. *Appl. Catal., B* **117**, 384–396 (2012).

[61] Cobo, M. *et al.* Steam reforming of ethanol over bimetallic RhPt/La_2O_3: Long-term stability under favorable reaction conditions. *Int. J. Hydrog. Energy* **38**, 5580–5593 (2013).

[62] Sutton, J. E., Panagiotopoulou, P., Verykios, X. E. & Vlachos, D. G. Combined DFT, microkinetic, and experimental study of ethanol steam reforming on Pt. *J. Phys. Chem. C* **117**, 4691–4706 (2013).

[63] Hirai, T., Ikenaga, N.-O., Miyake, T. & Suzuki, T. Production of hydrogen by steam reforming of glycerin on ruthenium catalyst. *Energy & Fuels* **19**, 1761–1762 (2005).

[64] Adhikari, S., Fernando, S. & Haryanto, A. Production of hydrogen by steam reforming of glycerin over alumina-supported metal catalysts. *Catal. Today* **129**, 355–364 (2007).

[65] Harju, H., Lehtonen, J. & Lefferts, L. Steam reforming of n-butanol over Rh/ZrO_2 catalyst: Role of 1-butene and butyraldehyde. *Appl. Catal., B* **182**, 33–46 (2016).

[66] Lin, L. *et al.* Low-temperature hydrogen production from water and methanol using Pt/α-MoC catalysts. *Nature* **544**, 80–83 (2017).

[67] Shabaker, J. W., Davda, R. R., Huber, G. W., Cortright, R. D. & Dumesic, J. A. Aqueous-phase reforming of methanol and ethylene glycol over alumina-supported platinum catalysts. *J. Catal.* **215**, 344–352 (2003).

[68] Cruz, I. O., Ribeiro, N. F. P., Aranda, D. A. G. & Souza, M. M. V. M. Hydrogen production by aqueous-phase reforming of ethanol over nickel catalysts prepared from hydrotalcite precursors. *Catal. Commun.* **9**, 2606–2611 (2008).

[69] Huber, G. W., Shabaker, J. W., Evans, S. T. & Dumesic, J. A. Aqueous-phase reforming of ethylene glycol over supported Pt and Pd bimetallic catalysts. *Appl. Catal. B* **62**, 226–235 (2006).

[70] Liu, J. *et al.* Aqueous-phase reforming of ethylene glycol to hydrogen on Pd/Fe_3O_4 catalyst prepared by co-precipitation: Metal–support interaction and excellent intrinsic activity. *J. Catal.* **274**, 287–295 (2010).

[71] De Vlieger, D., Mojet, B., Lefferts, L. & Seshan, K. Aqueous phase reforming of ethylene glycol – role of intermediates in catalyst performance. *J. Catal.* **292**, 239–245 (2012).

[72] Kim, H.-D. *et al.* Hydrogen production through the aqueous phase reforming of ethylene glycol over supported Pt-based bimetallic catalysts. *Int. J. Hydrog. Energy* **37**, 8310–8317 (2012).

[73] Huber, G. W., Shabaker, J. W. & Dumesic, J. A. Raney Ni-Sn catalyst for H_2 production from biomass-derived hydrocarbons. *Science* **300**, 2075–2077 (2003).

[74] Huber, G. W., Cortright, R. D. & Dumesic, J. A. Renewable alkanes by aqueous-phase reforming of biomass-derived oxygenates. *Angew. Chem. Int. Ed.* **43**, 1549–1551 (2004).

[75] Shabaker, J. W., Huber, G. W. & Dumesic, J. A. Aqueous-phase reforming of oxygenated hydrocarbons over Sn-modified Ni catalysts. *J. Catal.* **222**, 180–191 (2004).

[76] King, D. L. *et al.* Aqueous phase reforming of glycerol for hydrogen production over Pt–Re supported on carbon. *Appl. Catal., B* **99**, 206–213 (2010).

[77] Godina, L. I., Kirilin, A. V., Tokarev, A. V. & Murzin, D. Y. Aqueous phase reforming of industrially relevant sugar alcohols with different chiralities. *ACS Catal.* **5**, 2989–3005 (2015).

[78] Gomes, J. R. B. & Gomes, J. A. N. F. A DFT study of the methanol oxidation catalyzed by a copper surface. *Surf. Sci.* **471**, 59–70 (2001).

[79] Greeley, J. & Mavrikakis, M. Methanol decomposition on Cu(111): A DFT study. *J. Catal.* **208**, 291–300 (2002).

[80] Zhao, Y.-F. *et al.* Insight into methanol synthesis from CO_2 hydrogenation on Cu(111): Complex reaction network and the effects of H_2O. *J. Catal.* **281**, 199–211 (2011).

[81] Lawton, T. J., Carrasco, J., Baber, A. E., Michaelides, A. & Sykes, E. C. H. Hydrogen-bonded assembly of methanol on Cu(111). *Phys. Chem. Chem. Phys.* **14**, 11846–11852 (2012).

[82] Zuo, Z.-J., Wang, L., Han, P.-D. & Huang, W. Insights into the reaction mechanisms of methanol decomposition, methanol oxidation and steam reforming of methanol on Cu(111): A density functional theory study. *Int. J. Hydrog. Energy* **39**, 1664–1679 (2014).

[83] Lin, S., Johnson, R. S., Smith, G. K., Xie, D. & Guo, H. Pathways for methanol steam reforming involving adsorbed formaldehyde and hydroxyl intermediates on Cu(111): Density functional theory studies. *Phys. Chem. Chem. Phys.* **13**, 9622–9631 (2011).

[84] Jiang, Z., Guo, S. & Fang, T. Theoretical investigation on the dehydrogenation mechanism of CH_3OH on Cu(100) surface. *J. Alloys Compd.* **698**, 617–625 (2017).

[85] Zhang, Y.-C., Zuo, Z.-J., Ren, R.-P. & Lv, Y.-K. Insights into the effect of Pt doping of Cu(110)/H_2O for methanol decomposition: A density functional theory study. *RSC Adv.* **6**, 109124–109131 (2016).

[86] Ren, R.-P., Zhang, Y.-C., Liu, S., Zuo, Z.-J. & Lv, Y.-K. DFT studies of the methanol decomposition mechanism on the H_2O/Cu(110) and OH pre-adsorbed H_2O/Cu(110) interfaces: Comparison with the clean Cu(110) surface. *Int. J. Hydrog. Energy* **41**, 2411–2423 (2016).

[87] Singnurkar, P. *et al.* DFT and RAIRS investigations of methanol on Cu(110) and on oxygen-modified Cu(110). *J. Phys. Chem. C* **112**, 14034–14040 (2008).

[88] Sakong, S. & Groß, A. Density functional theory study of the partial oxidation of methanol on copper surfaces. *J. Catal.* **231**, 420–429 (2005).

[89] Sakong, S., Sendner, C. & Groß, A. Partial oxidation of methanol on Cu(110): Energetics and kinetics. *THEOCHEM* **771**, 117–122 (2006).

[90] Sakong, S. & Gross, A. Total oxidation of methanol on Cu(110): A density functional theory study. *J. Phys. Chem. A* **111**, 8814–8822 (2007).

[91] Li, X. & Lim, K. H. DFT study of steam reforming of formaldehyde on Cu, PdZn, and Ir. *ChemCatChem* **4**, 1311–1320 (2012).

[92] Li, J. & Zhou, G. Density functional theory study of O–H and C–H bond scission of methanol catalyzed by a chemisorbed oxygen layer on Cu(111). *Surf. Sci.* **646**, 288–297 (2016).

[93] Gu, X.-K. & Li, W.-X. First-principles study on the origin of the different selectivities for methanol steam reforming on Cu(111) and Pd(111). *J. Phys. Chem. C* **114**, 21539–21547 (2010).

[94] Murphy, C. J. *et al.* Structure and energetics of hydrogen-bonded networks of methanol on close packed transition metal surfaces. *J. Chem. Phys.* **141**, 014701 (2014).

[95] Wang, J., Kawazoe, Y., Sun, Q., Chan, S. & Su, H. The selectivity and activity of catalyst for CO hydrogenation to methanol and hydrocarbon: A comparative study on Cu, Co and Ni surfaces. *Surf. Sci.* **645**, 30–40 (2016).

[96] Chen, Z.-X., Neyman, K. M., Lim, K. H. & Rösch, N. CH_3O decomposition on PdZn(111), Pd(111), and Cu(111). A theoretical study. *Langmuir* **20**, 8068–8077 (2004).

[97] Yang, Y., White, M. G. & Liu, P. Theoretical study of methanol synthesis from CO_2 hydrogenation on metal-doped Cu(111) surfaces. *J. Phys. Chem. C* **116**, 248–256 (2012).

[98] Zuo, Z.-J., Gao, X.-Y., Han, P.-D., Liu, S.-Z. & Huang, W. Density Functional Theory (DFT) and Kinetic Monte Carlo (KMC) study of the reaction mechanism of hydrogen production from methanol on ZnCu(111). *J. Phys. Chem. C* **120**, 27500–27508 (2016).

[99] Schennach, R., Eichler, A. & Rendulic, K. D. Adsorption and desorption of methanol on Pd(111) and on a Pd/V surface alloy. *J. Phys. Chem. B* **107**, 2552–2558 (2003).

[100] Yudanov, I. V., Matveev, A. V., Neyman, K. M. & Rösch, N. How the C–O bond breaks during methanol decomposition on nanocrystallites of palladium catalysts. *J. Am. Chem. Soc.* **130**, 9342–9352 (2008).

[101] Jiang, R., Guo, W., Li, M., Fu, D. & Shan, H. Density functional investigation of methanol dehydrogenation on Pd(111). *J. Phys. Chem. C* **113**, 4188–4197 (2009).

[102] Huang, Y. & Chen, Z.-X. Density functional investigations of methanol dehydrogenation on Pd-Zn surface alloy. *Langmuir* **26**, 10796–10802 (2010).

[103] Huang, Y., He, X. & Chen, Z.-X. First-principles study towards the reactivity of the Pd(111) surface with low Zn deposition. *J. Chem. Phys.* **134**, 184702 (2011).

[104] Lin, S., Ma, J., Ye, X., Xie, D. & Guo, H. CO hydrogenation on Pd(111): Competition between Fischer–Tropsch and oxygenate synthesis pathways. *J. Phys. Chem. C* **117**, 14667–14676 (2013).

[105] Jiang, Z., Wang, B. & Fang, T. A theoretical study on the complete dehydrogenation of methanol on Pd(100) surface. *Appl. Surf. Sci.* **364**, 613–619 (2016).

[106] Lin, S. *et al.* Influence of step defects on methanol decomposition: Periodic density functional studies on Pd(211) and kinetic Monte Carlo simulations. *J. Phys. Chem. C* **117**, 451–459 (2013).

[107] Ye, J., Liu, C. & Ge, Q. A DFT study of methanol dehydrogenation on the PdIn(110) surface. *Phys. Chem. Chem. Phys.* **14**, 16660–16667 (2012).

[108] Smith, G. K. *et al.* Initial steps in methanol steam reforming on PdZn and ZnO surfaces: Density functional theory studies. *Surf. Sci.* **605**, 750–759 (2011).

[109] Lin, S., Xie, D. & Guo, H. First-principles study of the methyl formate pathway of methanol steam reforming on PdZn(111) with comparison to Cu(111). *J. Mol. Catal. A: Chem.* **356**, 165–170 (2012).

[110] Lin, S., Xie, D. & Guo, H. Pathways of methanol steam reforming on PdZn and comparison with Cu. *J. Phys. Chem. C* **115**, 20583–20589 (2011).

[111] Huang, Y., He, X. & Chen, Z.-X. Density functional study of methanol decomposition on clean and O or OH adsorbed PdZn(111). *J. Chem.Phys.* **138**, 184701 (2013).

[112] Huang, Z.-Q., Long, B. & Chang, C.-R. A theoretical study on the catalytic role of water in methanol steam reforming on PdZn(111). *Catal. Sci. Tech* **5**, 2935–2944 (2015).

[113] Cheng, F. & Chen, Z.-X. Where does methanol lose hydrogen to trigger steam reforming? A revisit of methanol dehydrogenation on the PdZn alloy model obtained from kinetic Monte Carlo simulations. *Phys. Chem. Chem. Phys.* **18**, 3936–3943 (2016).

[114] Greeley, J. & Mavrikakis, M. A first-principles study of methanol decomposition on Pt(111). *J. Am. Chem. Soc.* **124**, 7193–7201 (2002).

[115] Greeley, J. & Mavrikakis, M. Competitive paths for methanol decomposition on Pt(111). *J. Am. Chem. Soc.* **126**, 3910–3919 (2004).

[116] Zhu, H. *et al.* Decomposition of methanthiol on Pt(111): A density functional investigation. *Langmuir* **26**, 12017–12025 (2010).

[117] Zhao, L. *et al.* The oxidation of methanol on PtRu(111): A periodic density functional theory investigation. *J. Phys. Chem. C* **119**, 20389–20400 (2015).

[118] Yuan, D., Gong, X. & Wu, R. Decomposition pathways of methanol on the PtAu(111) bimetallic surface: A first-principles study. *J. Chem.Phys.* **128**, 064706 (2008).

[119] Zhong, W., Liu, Y. & Zhang, D. Theoretical study of methanol oxidation on the PtAu(111) bimetallic surface: CO pathway vs non-CO pathway. *J. Phys. Chem. C* **116**, 2994–3000 (2012).

[120] Ding, Q. *et al.* Insight into the reaction mechanisms of methanol on PtRu/Pt(111): A density functional study. *Appl. Surf. Sci.* **369**, 257–266 (2016).

[121] Lu, X. *et al.* Methanol oxidation on Pt_3Sn(111) for direct methanol fuel cells: Methanol decomposition. *ACS Appl. Mater. Interfaces* **8**, 12194–12204 (2016).

[122] Du, P., Wu, P. & Cai, C. Mechanistic insight into the facet-dependent adsorption of methanol on a Pt_3Ni nanocatalyst. *J. Phys. Chem. C* **119**, 18352–18363 (2015).

[123] Wang, X., Chen, L. & Li, B. A density functional theory study of methanol dehydrogenation on the $PtPd_3$(111) surface. *Int. J. Hydrog. Energy* **40**, 9656–9669 (2015).

[124] Kramer, Z. C., Gu, X.-K., Zhou, D. D. Y., Li, W.-X. & Skodje, R. T. Following molecules through reactive networks: Surface catalyzed decomposition of methanol on Pd(111), Pt(111), and Ni(111). *J. Phys. Chem. C* **118**, 12364–12383 (2014).

[125] Krajčí, M., Tsai, A.-P. & Hafner, J. Understanding the selectivity of methanol steam reforming on the (111) surfaces of NiZn, PdZn and PtZn: Insights from DFT. *J. Catal.* **330**, 6–18 (2015).

[126] Ferrin, P., Nilekar, A. U., Greeley, J., Mavrikakis, M. & Rossmeisl, J. Reactivity descriptors for direct methanol fuel cell anode catalysts. *Surf. Sci.* **602**, 3424–3431 (2008).

[127] Zhang, M., Yao, R., Jiang, H., Li, G. & Chen, Y. Catalytic activity of transition metal doped Cu(111) surfaces for ethanol synthesis from acetic acid hydrogenation: A DFT study. *RSC Adv.* **7**, 1443–1452 (2017).

[128] Zhou, Y.-H., Lv, P.-H. & Wang, G.-C. DFT studies of methanol decomposition on Ni(100) surface: Compared with Ni(111) surface. *J. Mol. Catal. A: Chem.* **258**, 203–215 (2006).

[129] Wang, G.-C. *et al.* Kinetic mechanism of methanol decomposition on Ni(111) surface: A theoretical study. *J. Phys. Chem. B* **109**, 12431–12442 (2005).

[130] Remediakis, I. N., Abild-Pedersen, F. & Nørskov, J. K. DFT study of formaldehyde and methanol synthesis from CO and H_2 on Ni(111). *J. Phys. Chem. B* **108**, 14535–14540 (2004).

[131] Chen, W.-K., Liu, S.-H., Cao, M.-J., Yan, Q.-G. & Lu, C.-H. Adsorption and dissociation of methanol on Au(111) surface: A first-principles periodic density functional study. *THEOCHEM* **770**, 87–91 (2006).

[132] Wang, L., He, C., Zhang, W., Li, Z. & Yang, J. Methanol-selective oxidation pathways on Au surfaces: A first-principles study. *J. Phys. Chem. C* **118**, 17511–17520 (2014).

[133] Moura, A. S., Fajín, J. L. C., Pinto, A. S. S., Mandado, M. & D S Cordeiro, M. N. Competitive paths for methanol decomposition on ruthenium: A DFT study. *J. Phys. Chem. C* **119**, 27382–27391 (2015).

[134] Lu, X. *et al.* Methanol oxidation on Ru(0001) for direct methanol fuel cells: Analysis of the competitive reaction mechanism. *RSC Adv.* **6**, 1729–1737 (2016).

[135] Wang, H., He, C.-Z., Huai, L.-Y., Tao, F.-M. & Liu, J.-Y. Decomposition of methanol on clean and oxygen-predosed V(100): A first-principles study. *J. Phys. Chem. C* **116**, 25344–25353 (2012).

[136] Wang, H., He, C.-z., Huai, L.-y. & Liu, J.-y. Decomposition and oxidation of methanol on Ir(111): A first-principles study. *J. Phys. Chem. C* **117**, 4574–4584 (2013).

[137] Luo, W. & Asthagiri, A. Density functional theory study of methanol steam reforming on Co(0001) and Co(111) surfaces. *J. Phys. Chem. C* **118**, 15274–15285 (2014).

[138] Halevi, B. *et al.* High CO_2 selectivity of ZnO powder catalysts for methanol steam reforming. *J. Phys. Chem. C* **117**, 6493–6503 (2013).

[139] Lin, S., Huang, J., Gao, X., Ye, X. & Guo, H. Theoretical insight into the reaction mechanism of ethanol steam reforming on Co(0001). *J. Phys. Chem. C* **119**, 2680–2691 (2015).

[140] Li, M.-R., Chen, J. & Wang, G.-C. Reaction mechanism of ethanol on model cobalt catalysts: DFT calculations. *J. Phys. Chem. C* **120**, 14198–14208 (2016).

[141] Zheng, H., Zhang, R., Li, Z. & Wang, B. Insight into the mechanism and possibility of ethanol formation from syngas on Cu(100) surface. *J. Mol. Catal. A: Chem.* **404**, 115–130 (2015).

[142] Sun, K., Zhang, M. & Wang, L. Effects of catalyst surface and hydrogen bond on ethanol dehydrogenation to ethoxy on Cu catalysts. *Chem. Phys. Lett.* **585**, 89–94 (2013).

[143] Alcalá, R., Mavrikakis, M. & Dumesic, J. A. DFT studies for cleavage of C-C and C-O bonds in surface species derived from ethanol on Pt(111). *J. Catal.* **218**, 178–190 (2003).

[144] Wang, H.-F. & Liu, Z.-P. Comprehensive mechanism and structure-sensitivity of ethanol oxidation on platinum: New transition-state searching method for resolving the complex reaction network. *J. Am. Chem. Soc.* **130**, 10996–11004 (2008).

[145] Sutton, J. E., Guo, W., Katsoulakis, M. A. & Vlachos, D. G. Effects of correlated parameters and uncertainty in electronic-structure-based chemical kinetic modelling. *Nat. Chem.* **8**, 331–337 (2016).

[146] Pereira, A. O. & Miranda, C. R. Atomic scale insights into ethanol oxidation on Pt, Pd and Au metallic nanofilms: A DFT with van der Waals interactions. *Appl. Surf. Sci.* **288**, 564–571 (2014).

[147] Li, M., Guo, W., Jiang, R., Zhao, L. & Shan, H. Decomposition of ethanol on Pd(111): A density functional theory study. *Langmuir* **26**, 1879–1888 (2009).

[148] Sheng, T., Lin, W.-F., Hardacre, C. & Hu, P. Role of water and adsorbed hydroxyls on ethanol electrochemistry on Pd: New mechanism, active centers, and energetics for direct ethanol fuel cell running in alkaline medium. *J. Phys. Chem. C* **118**, 5762–5772 (2014).

[149] Guo, W. *et al.* Ethanol decomposition on a Pd(110) surface: A density functional theory investigation. *Dalton Trans.* **42**, 2309–2318 (2013).

[150] Li, M. *et al.* Density functional study of ethanol decomposition on Rh(111). *J. Phys. Chem. C* **114**, 21493–21503 (2010).

[151] Syu, C.-Y. & Wang, J.-H. Mechanistic study of the oxidative steam reforming of EtOH on Rh(111): The importance of the oxygen effect. *ChemCatChem* **5**, 3164–3174 (2013).

[152] Zhang, J. *et al.* Density functional theory studies of ethanol decomposition on Rh(211). *J. Phys. Chem. C* **115**, 22429–22437 (2011).

[153] Zhang, R., Wang, G. & Wang, B. Insights into the mechanism of ethanol formation from syngas on Cu and an expanded prediction of improved Cu-based catalyst. *J. Catal.* **305**, 238–255 (2013).

[154] Zhang, J. *et al.* Ethanol steam reforming on Rh catalysts: Theoretical and experimental understanding. *ACS Catal.* **4**, 448–456 (2014).

[155] Yang, M.-M., Bao, X.-H. & Li, W.-X. First principle study of ethanol adsorption and formation of hydrogen bond on Rh(111) surface. *J. Phys. Chem. C* **111**, 7403–7410 (2007).

[156] Zhang, R., Wang, G., Wang, B. & Ling, L. Insight into the effect of promoter Mn on ethanol formation from syngas on a Mn-promoted MnCu(211) surface: A comparison with a Cu(211) Surface. *J. Phys. Chem. C* **118**, 5243–5254 (2014).

[157] Tereshchuk, P. & Da Silva, J. L. F. Density functional investigation of the adsorption of ethanol–water mixture on the Pt(111) surface. *J. Phys. Chem. C* **117**, 16942–16952 (2013).

[158] Xing, S.-K. & Wang, G.-C. Reaction mechanism of ethanol decomposition on $Mo_2C(100)$ investigated by the first principles study. *J. Mol. Catal. A: Chem.* **377**, 180–189 (2013).

[159] Choi, Y. & Liu, P. Mechanism of ethanol synthesis from syngas on Rh(111). *J. Am. Chem. Soc.* **131**, 13054–13061 (2009).

[160] Choi, Y. & Liu, P. Understanding of ethanol decomposition on Rh(111) from density functional theory and kinetic Monte Carlo simulations. *Catal. Today* **165**, 64–70 (2011).

[161] Kapur, N., Hyun, J., Shan, B., Nicholas, J. B. & Cho, K. *Ab initio* study of CO hydrogenation to oxygenates on reduced Rh terraces and stepped surfaces. *J. Phys. Chem. C* **114**, 10171–10182 (2010).

[162] Resta, A. *et al.* Step enhanced dehydrogenation of ethanol on Rh. *Surf. Sci.* **602**, 3057–3063 (2008).

[163] Wang, J., Liu, Z., Zhang, R. & Wang, B. Ethanol synthesis from syngas on the stepped Rh(211) surface: Effect of surface structure and composition. *J. Phys. Chem. C* **118**, 22691–22701 (2014).

[164] Chiang, H.-N., Nachimuthu, S., Cheng, Y.-C., Damayanti, N. P. & Jiang, J.-C. A DFT study of ethanol adsorption and decomposition on α-$Al_2O_3(0001)$ surface. *Appl. Surf. Sci.* **363**, 636–643 (2016).

[165] Ma, Y., Hernández, L., Guadarrama-Pérez, C. & Balbuena, P. B. Ethanol reforming on Co(0001) surfaces: A Density Functional Theory study. *J. Phys. Chem. A* **116**, 1409–1416 (2012).

[166] Sutton, J. E. & Vlachos, D. G. Ethanol activation on closed-packed surfaces. *Ind. Eng. Chem. Res.* **54**, 4213–4225 (2015).

[167] Sutton, J. E. & Vlachos, D. G. Effect of errors in linear scaling relations and Brønsted–Evans–Polanyi relations on activity and selectivity maps. *J. Catal.* **338**, 273–283 (2016).

[168] Wang, J.-H., Lee, C. S. & Lin, M. C. Mechanism of ethanol reforming: Theoretical foundations. *J. Phys. Chem. C* **113**, 6681–6688 (2009).

[169] Mei, D., Lebarbier Dagle, V., Xing, R., Albrecht, K. O. & Dagle, R. A. Steam reforming of ethylene glycol over $MgAl_2O_4$ supported Rh, Ni, and Co catalysts. *ACS Catal.* **6**, 315–325 (2016).

[170] Salciccioli, M. & Vlachos, D. G. Kinetic modeling of Pt catalyzed and computation-driven catalyst discovery for ethylene glycol decomposition. *ACS Catal.* **1**, 1246–1256 (2011).

[171] Kandoi, S. *et al.* Reaction kinetics of ethylene glycol reforming over platinum in the vapor versus aqueous phases. *J. Phys. Chem. C* **115**, 961–971 (2011).

[172] Gu, X.-K., Liu, B. & Greeley, J. First-principles study of structure sensitivity of ethylene glycol conversion on platinum. *ACS Catal.* **5**, 2623–2631 (2015).

[173] Auneau, F., Michel, C., Delbecq, F., Pinel, C. & Sautet, P. Unravelling the mechanism of glycerol hydrogenolysis over rhodium catalyst through combined experimental–theoretical investigations. *Chem. Eur. J.* **17**, 14288–14299 (2011).

[174] Tereshchuk, P., Chaves, A. S. & Da Silva, J. L. F. Glycerol adsorption on platinum surfaces: A density functional theory investigation with van der Waals corrections. *J. Phys. Chem. C* **118**, 15251–15259 (2014).

[175] Liu, B. & Greeley, J. Decomposition pathways of glycerol via C–H, O–H, and C–C bond scission on Pt(111): A density functional theory study. *J. Phys. Chem. C* **115**, 19702–19709 (2011).

[176] Liu, B. & Greeley, J. Density functional theory study of selectivity considerations for C–C versus C–O bond scission in glycerol decomposition on Pt(111). *Top. Catal.* **55**, 280–289 (2012).

[177] Chen, Y., Salciccioli, M. & Vlachos, D. G. An efficient reaction pathway search method applied to the decomposition of glycerol on platinum. *J. Phys. Chem. C* **115**, 18707–18720 (2011).

[178] García-Ratés, M. & López, N. Multigrid-based methodology for implicit solvation models in periodic DFT. *J. Chem. Theory Comput.* **12**, 1331–1341 (2016).
